# Genome sequencing of the staple food crop white Guinea yam enables the development of a molecular marker for sex determination

**DOI:** 10.1186/s12915-017-0419-x

**Published:** 2017-09-19

**Authors:** Muluneh Tamiru, Satoshi Natsume, Hiroki Takagi, Benjamen White, Hiroki Yaegashi, Motoki Shimizu, Kentaro Yoshida, Aiko Uemura, Kaori Oikawa, Akira Abe, Naoya Urasaki, Hideo Matsumura, Pachakkil Babil, Shinsuke Yamanaka, Ryo Matsumoto, Satoru Muranaka, Gezahegn Girma, Antonio Lopez-Montes, Melaku Gedil, Ranjana Bhattacharjee, Michael Abberton, P. Lava Kumar, Ismail Rabbi, Mai Tsujimura, Toru Terachi, Wilfried Haerty, Manuel Corpas, Sophien Kamoun, Günter Kahl, Hiroko Takagi, Robert Asiedu, Ryohei Terauchi

**Affiliations:** 1 Iwate Biotechnology Research Center, Kitakami, Japan; 2 The Earlham Institute, Norwich, UK; 3 Kobe University, Kobe, Japan; 4 Okinawa Agricultural Research Center, Naha, Japan; 5 Shinshu University, Nagano, Japan; 6 Tokyo University of Agriculture, Tokyo, Japan; 7 Japan International Research Center for Agricultural Sciences, Tsukuba, Japan; 8 International Institute of Tropical Agriculture, Ibadan, Nigeria; 9 Kyoto Sangyo University, Kyoto, Japan; 10 The Sainsbury Laboratory, Norwich, UK; 11 University of Frankfurt, Frankfurt, Germany; 12 Kyoto University, Kyoto, Japan

**Keywords:** Yam, *Dioscorea*, Whole-genome sequence, Dioecy, Sex determination

## Abstract

**Background:**

Root and tuber crops are a major food source in tropical Africa. Among these crops are several species in the monocotyledonous genus *Dioscorea* collectively known as yam, a staple tuber crop that contributes enormously to the subsistence and socio-cultural lives of millions of people, principally in West and Central Africa. Yam cultivation is constrained by several factors, and yam can be considered a neglected “orphan” crop that would benefit from crop improvement efforts. However, the lack of genetic and genomic tools has impeded the improvement of this staple crop.

**Results:**

To accelerate marker-assisted breeding of yam, we performed genome analysis of white Guinea yam (*Dioscorea rotundata*) and assembled a 594-Mb genome, 76.4% of which was distributed among 21 linkage groups. In total, we predicted 26,198 genes. Phylogenetic analyses with 2381 conserved genes revealed that *Dioscorea* is a unique lineage of monocotyledons distinct from the Poales (rice), Arecales (palm), and Zingiberales (banana). The entire *Dioscorea* genus is characterized by the occurrence of separate male and female plants (dioecy), a feature that has limited efficient yam breeding. To infer the genetics of sex determination, we performed whole-genome resequencing of bulked segregants (quantitative trait locus sequencing [QTL-seq]) in F1 progeny segregating for male and female plants and identified a genomic region associated with female heterogametic (male = ZZ, female = ZW) sex determination. We further delineated the W locus and used it to develop a molecular marker for sex identification of Guinea yam plants at the seedling stage.

**Conclusions:**

Guinea yam belongs to a unique and highly differentiated clade of monocotyledons. The genome analyses and sex-linked marker development performed in this study should greatly accelerate marker-assisted breeding of Guinea yam. In addition, our QTL-seq approach can be utilized in genetic studies of other outcrossing crops and organisms with highly heterozygous genomes. Genomic analysis of orphan crops such as yam promotes efforts to improve food security and the sustainability of tropical agriculture.

**Electronic supplementary material:**

The online version of this article (doi:10.1186/s12915-017-0419-x) contains supplementary material, which is available to authorized users.

## Background

Yam is a collective name for tuber-bearing crops belonging to the monocotyledonous *Dioscorea* genus in the family Dioscoreaceae of the order Dioscoreales. This genus contains approximately 450 species which are primarily distributed in tropical and subtropical regions worldwide [1]. Among the Dioscoreaceae, three minor genera are monoecious (having male and female flowers on a plant), but the entire genus *Dioscorea* is characterized by dioecy (the presence of separate male and female plants), a feature shared by only 5–6% of angiosperms [2]. The origin of *Dioscorea* is supposed to be in the Late Cretaceous (~80 Mya [3]), suggesting that the origin of dioecy dates back to this time. Approximately 10 *Dioscorea* species have been independently domesticated in West Africa, Southeast Asia, and the Pacific and Caribbean islands [4]. *D. rotundata* is the most popular species in West and Central Africa, the main region for yam production worldwide, which contributed approximately 96% of the 63 million tons of yam produced globally in 2013 (Additional file 1: Table S1 and Additional file 2: Figure S1). *D. rotundata* (white Guinea yam) and *D. cayenensis* (yellow Guinea yam) represent a major source of food and income in this region, as well as an integral part of the socio-cultural life. This geographical region is often referred to as the “civilization of the yam,” reflecting the West African societies that are tightly linked to yam cultivation [5, 6].

Despite its considerable regional importance, Guinea yam has long been regarded as an “orphan” crop, as it is not traded around the world, and it has attracted little attention from researchers and little investment. Guinea yam cultivation is constrained by several factors. Seeds are seldom used as starting materials; instead, yams are commonly propagated clonally using small whole tubers (referred to as “seed yams”) or tuber pieces. Yam is an annual climber that requires stakes for support and is highly vulnerable to a plethora of pests and diseases. Therefore, an understanding of yam genetics and a systematic improvement of yam based on crossbreeding for traits associated with tuber yield and quality, a reduced requirement for staking, and resistance/tolerance to disease and nematodes are urgently needed. Genetic analysis of *Dioscorea* has been constrained by the small number of available genetic markers. Furthermore, *Dioscorea* cultivars are highly heterozygous due to their obligate outcrossing. This heterozygosity renders genetic analysis approaches commonly used in inbreeding species, e.g., linkage analysis using the segregating progeny of an F2 generation and recombinant inbred lines (RILs), inapplicable to yam.

The International Institute of Tropical Agriculture (IITA) has a global mandate for yam research and development within the CGIAR Consortium [7]. We initiated a yam genomics program several years ago as part of an IITA-coordinated international collaboration. To generate genetic and genomic tools for yam breeding, we sequenced and assembled a highly heterozygous diploid genome of *D. rotundata*. We used this genome sequence and genetic resources to identify a locus associated with sex determination, which we used to develop a diagnostic marker for sex identification at the seedling stage. These genomic resources broaden our knowledge of Guinea yam genetics and provide a platform for implementing genomics-assisted breeding by marker-assisted selection (MAS) in this important staple crop.

## Results

### Whole-genome sequencing (WGS) and assembly

**Fig. 1:**
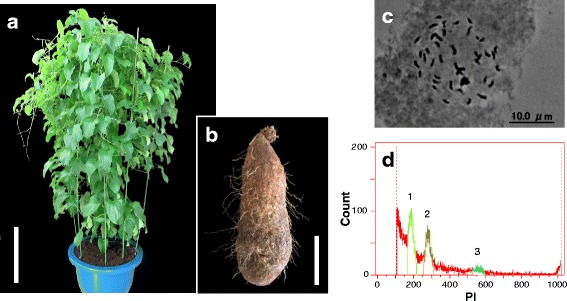
Determination of ploidy level and genome size in *Dioscorea rotundata* plant TDr96_F1. **a** TDr96_F1 plant grown in a greenhouse at Iwate Biotechnology Research Center (IBRC), Japan. Bar = 50 cm. **b** TDr96_F1 tuber. Bar = 10 cm. **c** Diploid somatic chromosomes at metaphase stage obtained from TDr96_F1 root tips (2*n* = 2× = 40). **d** FCM histogram of propidium iodide (PI)-stained nuclei from *D. rotundata* (TDr96_F1) and rice (*Oryza sativa* L.). Rice (genome size = 380 Mb) served as an internal reference standard. 1 = G1 (*O. sativa*), 2 = G1 (*D. rotundata*), and 3 = G2 (*D. rotundata*), where G1 and G2 represent the Gap 1 and Gap 2 phases of the cell cycle, respectively

To generate a *D. rotundata* genome sequence, an individual plant, TDr96_F1, was selected from the progeny in the open-pollinated *D. rotundata* breeding line TDr96/00629 (Fig. 1a, b). As TDr96_F1 never flowered during the current study period, we could not determine its sex. While *D. rotundata* is characterized by different ploidy levels (2× and 3×) with a basic chromosome number of 20 [8, 9], we found TDr96_F1 to be diploid (2*n* = 2× = 40) based on the mitotic chromosome number within root meristem cells (Fig. 1c). We estimated the genome size of TDr96_F1 to be 570 Mb by flow cytometry (FCM) analysis (Fig. 1d).

We used total DNA from fresh leaf samples to prepare a paired-end (PE) library and eight types of mate-pair (MP) jump libraries with insert sizes of 2, 3, 4, 5, 6, 8, 20, and 40 kb and sequenced the PE and MP jump libraries on Illumina sequencers. We also generated a 100-kb jump bacterial artificial chromosome (BAC) library, from which 9984 clones were subjected to BAC-end Sanger sequencing, resulting in PE reads corresponding to a 0.46-Gb sequence with ~ 0.8× genome coverage (Additional file 1: Table S2 and Additional file 2: Figure S2). In total, we generated 85.14 Gb of sequencing reads, representing ~ 149.4× coverage of the estimated 570-Mb genome (Additional file 1: Table S2). Using *k*-mer analysis-based genome size estimation [10] of TDr96_F1 PE reads with ALLPATHS-LG [11] (see below), we found that the genome size was roughly 579 Mb, which is similar to the size estimated by FCM (Additional file 2: Figure S3). The PE and MP jump reads were used for de novo assembly with the ALLPATHS-LG assembler [11], which provides good performance even for highly heterozygous genomes [12]. Further scaffolding with SSPACE software using the 100-kb jump reads [13] (Additional file 2: Figure S4) generated 4723 scaffolds with a total length of 594 Mb, i.e., 2.6% and 4.2% longer than the genome size estimated by *k*-mer (579 Mb) and FCM (570 Mb) analyses, respectively. We estimated the scaffold N50 to be 2.12 Mb (longest scaffold: 13.6 Mb), with approximately 93.9% of the assembly represented by 586 scaffolds longer than 100 kb (Additional file 1: Table S3). From ALLPATHS-LG output, we judged that more than 1.4 million sites were potentially heterozygous (Table 1). This assembly is hereafter referred to as the “TDr96_F1 reference genome.”

**Table 1:** Characteristics of nuclear genome sequence in *Dioscorea rotundata* and other angiosperms

Feature	Value			
	*D. rotundata* (v0.1)	*A. thaliana* (TAIR10)	*B. distachyon* (v3.1)	*O. sativa* (v7_JGI 323)
Total length (Mbp)	594.23	119.67	271.16	374.47
GC (%)	35.83	36.06	46.40	43.57
Number of scaffolds (≥ 0 bp)	4723	7	10	14
Number of scaffolds (≥ 1000 bp)	4704	7	10	14
Largest scaffold (Mbp)	13.61	30.43	75.07	43.27
N50 (Mbp)	2.12	23.46	59.13	29.96
N75 (Mbp)	0.77	19.70	48.59	28.44
Number of Ns per 100 kb	282.45^a^	155.60	155.85	44.13
Ambiguous bases	1,413,029	–	–	–
Number of genes	26,198	27,416	34,310	42,189
Exons				
Number	158,059	141,044	154,104	178,353
Average number per gene	6.03	5.14	4.49	4.25
Total length (Mbp)	42.43	33.49	39.01	46.85
Average size (bp)	268.43	237.46	253.15	262.70
Average GC (%)	44.08	43.70	51.02	51.12
Introns				
Number	105,663	86,212	85,484	94,345
Average number per gene	4.03	3.14	2.49	2.25
Total length (Mbp)	83.12	17.87	47.70	53.34
Average size (bp)	630.33	157.25	398.18	391.23
Average GC (%)	32.37	32.45	38.29	37.20
Transposable elements^b^				
% Total interspersed	46.07	13.32	37.39	44.40
Total interspersed total length (Mbp)	274.51	15.94	101.39	166.27
% Short interspersed nuclear elements (SINEs)	0.02	0.17	0.38	0.88
SINEs total length (Mbp)	0.13	0.20	1.02	3.31
% Long interspersed nuclear elements (LINEs)	2.43	1.07	2.91	1.29
LINEs total length (Mbp)	14.46	1.29	7.90	4.83
% Long terminal repeat (LTR) elements	22.82	6.35	19.31	21.09
LTR elements total length (Mbp)	135.71	7.61	52.36	78.98
% DNA elements	6.70	3.08	7.11	16.7
DNA elements total length (Mbp)	39.83	3.69	19.27	62.82
% Unclassified	14.20	2.64	7.68	4.36
Unclassified total length (Mbp)	84.38	3.16	20.84	16.32

^a^Number of Ns per 100 kb using the *D. rotundata* broken scaffolds

^b^Transposable elements were identified by masking the genomes using RepeatModeler and RepeatMasker, with the same parameters across all species

We assessed the quality of our assembly by investigating the presence of 248 highly conserved core eukaryotic genes with the Core Eukaryotic Genes Mapping Approach (CEGMA) [14] and confirmed the presence of 243 (98%) of those genes (Additional file 1: Table S4). Similarly, 94% of 956 Benchmarking Universal Single-Copy Orthologs (BUSCOs) [15] were present in at least one complete single copy in the assembly (Additional file 1: Table S5). Since the TDr96_F1 reference genome was generated from total genomic DNA, it also contained organelle-derived sequences. Alignment of the TDr96_F1 PE reads to the published *D. rotundata* chloroplast genome sequence [16] showed that 14.7% of the total PE reads were derived from the chloroplast genome (Additional file 1: Table S6). We also isolated mitochondrial DNA from TDr96_F1 leaves, sequenced this DNA using PE reads with Illumina MiSeq, and generated a 564-kb de novo assembly comprising 76 scaffolds (Additional file 2: Figure S5). Among PE reads, 1.25% represented mitochondrial sequences.

### Generation of pseudo-chromosomes by anchoring scaffolds onto a linkage map

**Fig. 2:**
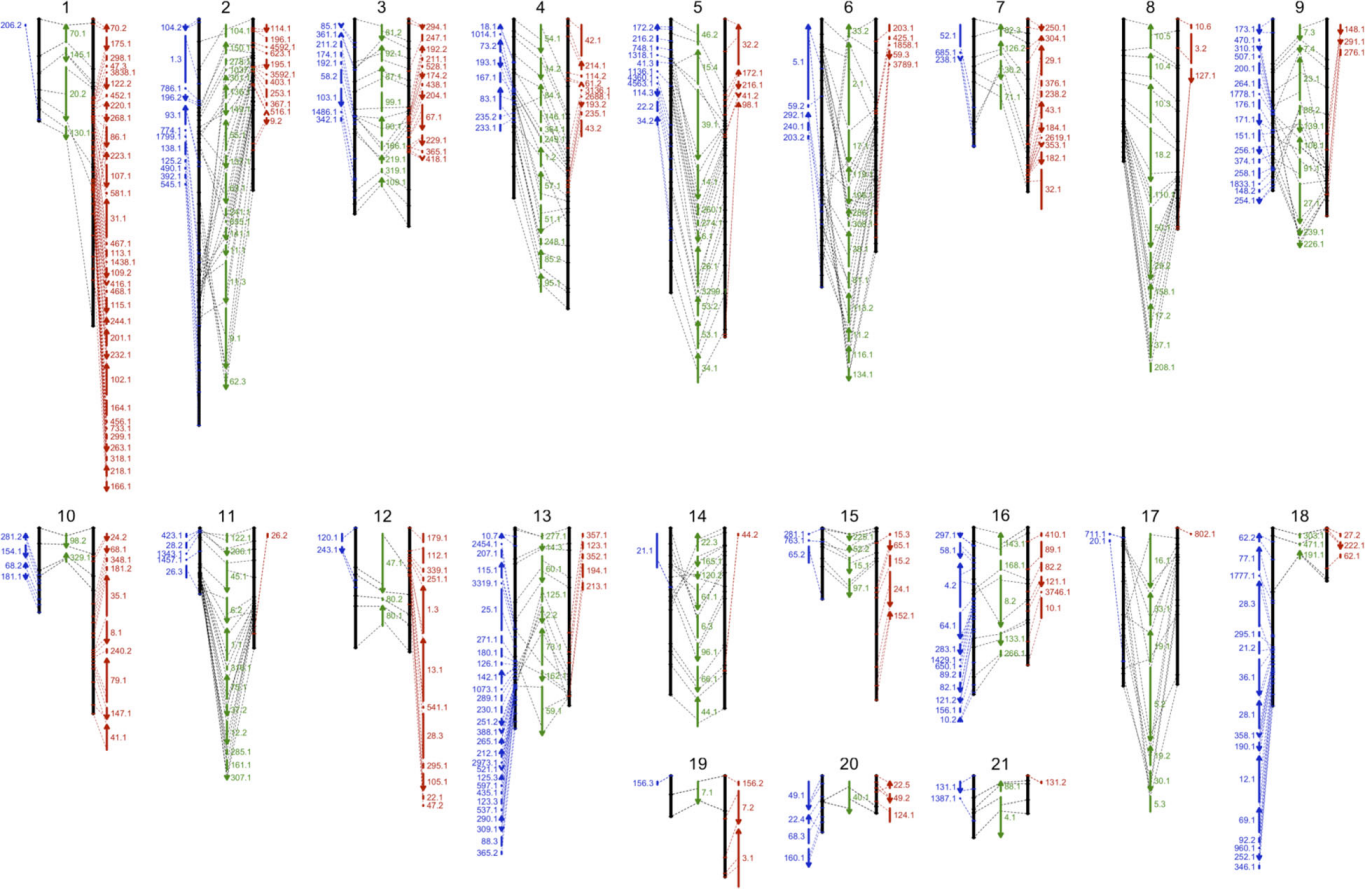
Integrated genetic and physical map of *D. rotundata*. Approximately 76.4% of *D. rotundata* scaffold sequences were anchored using a RAD-based genetic map generated with 150 F1 individuals obtained from a cross between TDr97/00917 (P1, female) and TDr99/02627 (P2, male). The 21 chromosome-scale pseudo-molecules are numbered from 1 to 21. Markers are located according to genetic distance (cM). *Black lines* represent the 21 P1 and P2 linkage groups (LGs), and scaffolds anchored to P1 and P2 LGs are shown in *red* and *blue*, respectively. Scaffolds shared between the P1 and P2 LGs are shown in *green. Numbers* and *arrows* indicate scaffolds and their orientation, respectively

We developed a genetic map of *D. rotundata* using 150 F1 individuals obtained from a cross between two heterozygous breeding lines, TDr97/00917 (P1, female) and TDr99/02627 (P2, male), using restriction site associated DNA (RAD)-tags as DNA markers [17] (Additional file 2: Figures S6, S7) and the pseudo-testcross method [18, 19]. We aligned RAD-tags to TDr96_F1 scaffold sequences and selected DNA markers heterozygous in P1 and homozygous in P2, as well as markers heterozygous in P2 and homozygous in P1, resulting in 1326 and 1272 markers for P1 and P2 heterozygous sites, respectively (Additional file 1: Table S7 and Additional file 2: Figure S8). We then calculated the recombination fraction (*rf*) between the RAD markers to generate linkage maps. If the pairwise *rf* value of two RAD markers on the same scaffold exceeded 0.25, the scaffold was divided halfway between the markers because they were likely misassembled (see explanation in Additional file 2: Figure S9 and Additional file 1: Table S8). Two linkage maps, P1-Map and P2-Map, were generated based on the segregation pattern of the selected markers in the F1 progeny (Additional file 2: Figure S10), to which *D. rotundata* scaffolds were anchored using the 100-bp DNA sequences of RAD-tags. We combined the two maps using shared scaffolds (Additional file 2: Figures S11, S12), which allowed the ~ 454-Mb sequence (representing 76.4% of the assembly) to be anchored onto 21 linkage groups (LGs) to construct chromosome-scale pseudo-molecules (Fig. 2 and Additional file 1: Table S9). Smaller LGs could not be unequivocally mapped; hence, 21 LGs were obtained, whereas 20 LGs are expected based on the basic chromosome number. We validated the quality of our assembly by comparing the pseudo-molecule sequence with newly sequenced PE sequence reads having an average insert size of ~ 100 kb obtained from the BACs. Among the 315 BAC clones for which sequences of both ends could be mapped onto the assembly, 265 (84.1%) had both pairs in the same scaffold in the correct orientation, with an average distance of 116 kb (Additional file 1: Table S10 and Additional file 2: Figure S13), confirming the quality of our assembly. We compared the de novo assembled scaffolds to linkage information about the RAD markers, finding that 75% of RAD markers on the same scaffolds had *rf* < 0.25, and 73.5% of the scaffolds were retained without the need for splitting (Additional file 1: Table S11). The remaining 26.5% of the scaffolds had an *rf* > 0.25 and were divided into two or more scaffolds to solve the inconsistency between assembly and linkage information.

### Guinea yam gene prediction and comparative genomics

We predicted genes and transposons using the TDr96_F1 reference genome sequence. To construct reliable gene models, we followed the MAKER pipeline using RNA-seq data from 18 samples representing various *D. rotundata* tissues (Additional file 1: Tables S12, S13) and combined the data with publicly available expressed sequence tags (ESTs) and homologous protein sequences from related angiosperm species (Additional file 2: Figure S14). This resulted in the prediction of 26,198 genes (Table 1 and Additional file 3), 22,477 (85.8%) of which are supported by RNA-seq data.

We compared Guinea yam genome sequence metrics with those of *Arabidopsis thaliana* (dicot), *Brachypodium distachyon* (monocot), and *Oryza sativa* (monocot) (Table 1). Interestingly, the GC contents of the total genome and exons of protein-coding genes in Guinea yam were 35.8% and 44.1%, respectively; these values are close to those of *Arabidopsis* and much lower than those of the Poales species *Brachypodium* and *Oryza* (Table 1). We annotated an average of 6.03 exons and 4.03 introns per gene. Roughly half of the genome was represented by an interspersed sequence (274.5 Mb), a major component of which was long terminal repeat (LTR) sequences (135.7 Mb) (Table 1).

**Fig. 3:**
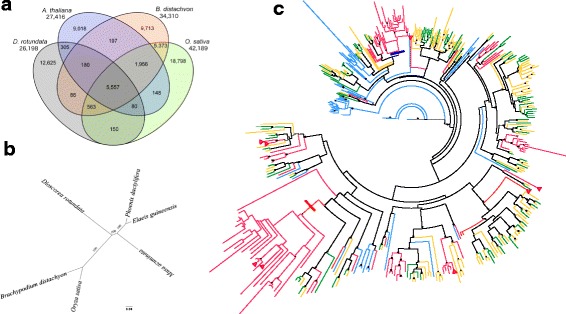
Comparative genomics of *Dioscorea rotundata* and other angiosperm species. **a** Venn diagram showing conserved and unique genes at 1:1 correspondence among *D. rotundata*, *Arabidopsis thaliana*, *Brachypodium distachyon*, and *Oryza sativa.* Total gene counts in each genome are given below the species name. **b** Maximum likelihood tree of *D. rotundata*, *B. distachyon*, *O. sativa*, *Elaeis guineensis*, *Musa acuminata*, and *Phoenix dactylifera* based on 2381 orthologous protein-coding genes. The bootstrap values across 1000 resamplings are shown. The scale bar represents the mean number of substitutions per site. **c** Phylogenetic analysis of the relationships of mannose-specific bulb-type lectin proteins in *D. rotundata* (*red*), *A. thaliana* (*blue*), *B. distachyon* (*green*), and *O. sativa* (*orange*). *Arrowheads* represent bulb-type lectins observed to have enriched expression in tubers. High confidence bootstrap values (1000 replicates) are represented at the nodes of the tree as *dots. Thick red* and *blue lines* show two root branches of *D. rotundata*-specific expanded genes

We identified 5557 *D. rotundata* genes with a 1:1:1:1 orthologous relationship to the high-quality *B. distachyon*, *O. sativa*, and *A. thaliana* gene models (Fig. 3a and Additional files 4, 5). This number was reduced to 2795 genes when we included Arecales (*Elaeis guineensis*, *Phoenix dactylifera*) and Zingiberales (*Musa acuminata*) in our analysis (Additional files 6, 7). We constructed a phylogenetic tree based on the alignment of 2381 orthologous protein-coding genes in the five monocotyledonous species (Fig. 3b). *D. rotundata* did not group with any species in the tree, including *Musa* of Zingiberales, *Phoenix* and *Elaeis* of Arecales, and *Oryza* and *Brachypodium* of Poales, suggesting that *Dioscorea* diversified independently from these taxa in monocotyledons.

For 12,625 *D. rotundata* genes, no orthologs or paralogs were found in *B. distachyon*, *O. sativa*, or *A. thaliana*, and 11,348 *D. rotundata* genes had no clear homologs in any of the six species shown in Fig. 3a and Additional file 8. Of these 11,348 genes without homologs, 3422 were expressed in tuber tissues, a tissue type not shared with the other species examined.

Non-redundant Gene Ontology (GO) terms “intracellular organelle”, “protein binding”, and “ion binding” were significantly enriched among *D. rotundata* genes that showed no orthology to the other species, but not among the conserved genes (Additional files 9, 10). *D. rotundata* genes without orthologs in the other species included 68 genes encoding proteins with lectin domains that are involved in defense against microbial pathogens, nematodes, and insects, accounting for 31% of the 216 lectin-coding genes functionally annotated in *D. rotundata*. Among the 12 subfamilies of lectins [20], the bulb-type lectin (snowdrop lectin; B-lectin) family contributed the largest share (110) of genes in *D. rotundata* (Additional file 1: Table S14). Phylogenetic analysis of the B-lectin genes in *D. rotundata* (110 genes; 51 unique), *B. distachyon*, *O. sativa*, and *A. thaliana* revealed two expansions of B-lectin genes in *Dioscorea* (Fig. 3c). The first expansion (blue band) consisted of 22 receptor-like serine/threonine-protein kinases, which are thought to play a role in signaling and the activation of plant defense mechanisms [21]. The second expansion (red band) consisted of 28 mannose-binding lectins sharing high similarity with *Dioscorea batatas* tuber lectin DB1 (accession number AB178475). DB1 has insecticidal properties against cotton bollworm (*Helicoverpa armigera*), and studies in transgenic tobacco and rice plants expressing *DB1* demonstrated that it also confers resistance against green peach aphid and brown plant hopper, respectively [22–24]. Of these mannose-binding lectin genes in Guinea yam, 16 did not have orthologs in any of the six other species examined, and two showed enriched expression (Benjamini–Hochberg [25] adjusted *P* value [padj] < 0.05) in tubers.

RNA-seq analysis comparing three tuber tissues to all other nine tissues (Additional file 1: Table S12) revealed that 2023 genes were enriched in tubers. The top 50 highly expressed (padj < 0.05) genes included genes encoding starch synthases and branching enzymes, as well as three carbonic anhydrase-encoding genes. Basic Local Alignment Search Tool (BLASTP) (https://blast.ncbi.nlm.nih.gov) analysis showed that these carbonic anhydrase-encoding genes shared high identity (average 76%) with genes encoding *Dioscorea japonica* precursors of dioscorin, a tuber storage protein that has carbonic anhydrase activity and exists in multiple isoforms [26] (Additional file 11).

To infer the past genome duplication in *D. rotundata*, we performed genome-wide dot plot analysis of *D. rotundata* against itself (Additional file 2: Figure S15), which revealed no indication of genome duplication. Nevertheless, we observed 946 paralogous gene clusters composed of duplicated genes in *D. rotundata*. Of these, 145 duplicate clusters of paralogous genes were observed only in *D. rotundata*. To investigate macrosynteny between *D. rotundata* and related species, we carried out whole-genome syntenic dot plot analysis against the genomes of *Oryza sativa*, *Spirodela polyrhiza*, and *Phoenix dactylifera*. At the chromosomal level, it was difficult to observe synteny conservation between these species. To assess microsynteny conservation, we performed a syntenic path assembly [27] of the scaffolds from these species against *D. rotundata-*masked pseudo-chromosomes (see Methods). The reordering and reorientation of the scaffolds relative to *D. rotundata* pseudo-molecules identified large proportions of the genomes to be conserved at the microsyntenic level (Additional file 2: Figure S16). This suggested that the *D. rotundata* genome has undergone many recombination events after its divergence from the other species.

### Whole-genome resequencing of F1 bulk segregants identifies a genomic region associated with sex determination in *D. rotundata*

**Fig. 4:**
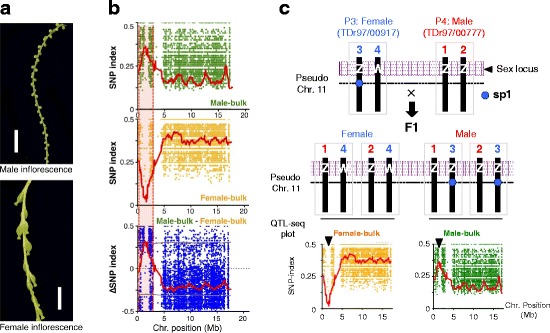
QTL-seq-based analysis of sex determination in *D. rotundata*. **a** Male and female inflorescences of *D. rotundata*. Bars = 10 mm. **b** SNP-index and ∆SNP-index plots generated for pseudo-chromosome 11 (see Fig. 2). DNA samples from 50 male and 50 female F1 individuals were pooled to prepare the male and female bulks, respectively. *Green*, *yellow*, and *blue dots* represent SNP-index values at all SNP positions, and *red lines* denote the sliding window average SNP-index values at 1-Mb intervals with 50-kb increments. *Horizontal brown lines* in the ∆SNP-index plot represent the 95% confidence limit. The candidate genomic region presumably associated with sex determination is indicated by a *pink background*. **c** Schematic diagram showing the possible genotypes of female (P3, TDr97/00917) and male (P4, TDr97/00777) parents as well as their F1 progeny segregating for female and male. Genotypes of sex-determination locus are indicated as ZW or ZZ. The position of the cleaved amplified polymorphic sequence (CAPS) marker, sp1, is indicated by a *dashed line*. Sister chromatids are indicated by numbers

We previously developed a next generation sequencing (NGS)-based method for bulked segregant analysis (BSA) for quantitative trait locus (QTL) mapping in rice, named QTL-seq [28]. To our knowledge, this method has not been applied in species with highly heterozygous genomes. The majority of *Dioscorea* species, including *D. rotundata*, are mostly dioecious, with separate male and female plants (Fig. 4a), making it interesting to understand the genetic mechanism of sex determination in this genus. From a cross between two *D. rotundata* breeding accessions, TDr97/00917 (P3, female) and TDr97/00777 (P4, male), we generated an F1 population of 253 individuals in 2014 that segregated for male, female, monoecious (male and female flowers on the same plant), and non-flowering types (Additional file 1: Table S15). For QTL-seq analysis (see Additional file 2: Figure S17 for details), we sequenced two DNA bulks representing male and female plants, each from 50 individuals, generating 7.9- and 7.3-Gb sequences, which provided 13.9× and 12.7× coverage of the predicted *D. rotundata* genome, respectively (Additional file 1: Table S16). We also resequenced the genome of the female parent (P3) and generated a P3 reference sequence (P3-Ref) by replacing TDr96_F1 nucleotides with P3 nucleotides at all different sites between the two genotypes. Likewise, we generated the male parent (P4) reference sequence (P4-Ref) by aligning P4 sequence reads to TDr96_F1 and replacing TDr96_F1 nucleotides with those of P4 at all different sites. We then separately aligned sequence reads obtained from F1 male-bulk and female-bulk DNA to the P3- and P4-Ref sequences. To identify single-nucleotide polymorphism (SNP) markers associated with the F1 gender phenotype, thus potentially suggesting candidate sex-determining gene(s), we focused on SNPs that segregated in the F1 progeny either as SNPs homozygous in the female parent (P3) but heterozygous in the male parent (P4), or vice versa. We could then identify genomic regions with SNPs heterozygous in one parent whose alleles were differentially transmitted to the two sexes in the F1, suggesting Y or W linkage, respectively. This is similar to mapping by backcrossing, but does not require a BC1 generation using inbred lines. Scanning the entire genome identified a single region, from 0.65 Mb to 2.35 Mb on pseudo-chromosome 11, whose SNP-index values (the frequency of short reads aligned to a particular position of the genome with SNPs different from the reference sequence [28]) differed for male and female bulks in the second category of SNPs just described (Fig. 4b and Additional file 2: Figure S18).

**Fig. 5:**
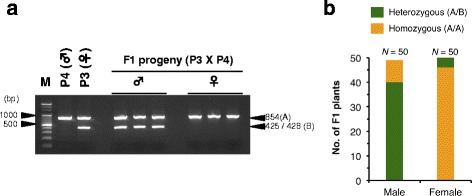
A CAPS marker developed on pseudo-chromosome 11 co-segregates with sex in F1 progeny derived from a cross between female (P3) and male (P4) parents. **a** Agarose gel electrophoresis of the CAPS marker, sp1, for the parents and F1 progeny segregating for male and female phenotypes. This marker segregates for a non-cleaved band (854 bp) indicated as (A) and cleaved bands (425 bp + 428 bp) indicated as (B). **b** Frequency of the sp1 genotypes (A/B or A/A) among the F1 progeny segregating for male (50 plants) and female (50 plants). There is a statistically significant association between A/B sp1 genotype and male and between A/A sp1 genotype and female (Fisher’s exact test: *P* = 1.913e-14)

We identified a sex-linked region with category 2 SNP markers that are heterozygous in the female parent (P3) but homozygous in the male parent (P4) (Additional file 2: Figure S18), suggesting that the male sex is determined by the homozygous (designated ZZ) state of the locus responsible for sex determination, whereas that of the female sex is determined by the heterozygous (ZW) (or hemizygous: Z-) state of this locus (Fig. 4c). Genotyping of the F1 individuals used for bulk sequencing using the cleaved amplified polymorphic sequence (CAPS) marker sp1 developed within the candidate genomic region revealed significant co-segregation between the sp1 marker and the sex of the individual (*P* = 1.913e-14, Fisher’s exact test). This analysis confirmed that the genomic region identified by QTL-seq is indeed associated with sex determination (Fig. 5a, b and Additional file 2: Figure S19). The switch of sp1 male and female marker genotypes in the F1 progeny occurred because the marker genotype was heterozygous in the female parent (Fig. 4c).

**Fig. 6:**
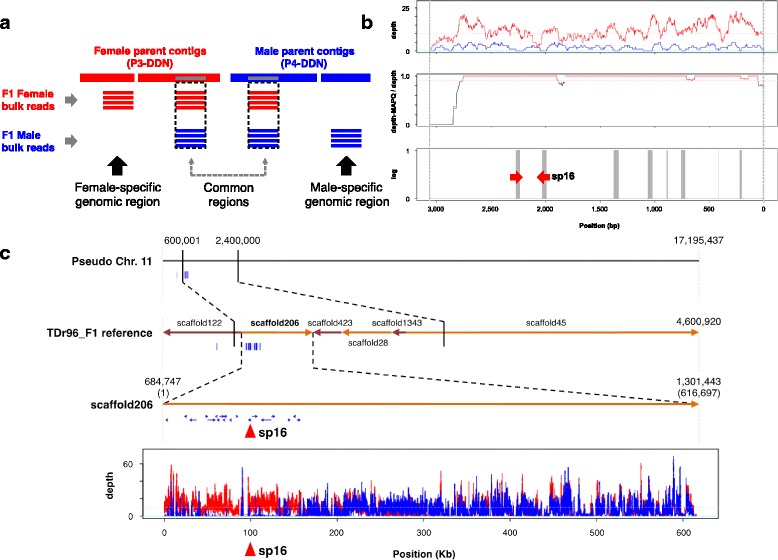
Identification of female-specific putative W-linked genomic region. **a** Schematic diagram of the method used to identify the female-specific putative W-linked genomic region. De novo assembled genome sequences of female (P3-DDN) and male (P4-DDN) parents were combined to serve as a reference sequence. Short reads of bulked DNA from F1 female and F1 male progeny were separately mapped onto this combined reference sequence. The majority of reads mapped to two duplicated homologous locations in the reference genome (indicated as “common regions”), which gave low MAPQ scores (<60) in the BWA alignment. Female parental contigs that were mapped only with reads belonging to the F1 female bulk corresponded to female-specific genomic regions. Sequence reads mapped to such positions were identified by their high MAPQ scores (=60). **b** An example of a female-specific contig (contig Female917_flattened_line_87512_3057). Alignment depths of F1 female bulk (*red*) and F1 male bulk (*blue*) are shown (*top*). Frequency of reads mapped with MAPQ score = 60. The *red line* corresponds to genomic regions that were covered by short reads, > 90% of which had a MAPQ score of 60 (middle). A genomic region that is covered only by female reads (not by male reads) and > 90% of mapped reads had MAPQ score = 60 (indicated by *gray bars*) (*bottom*). *Red arrowheads* indicate the positions of PCR primers for the DNA marker sp16. **c** Location of the female-specific genomic region. *Thick gray horizontal line* denotes pseudo-chromosome 11 (*top*), scaffolds on chromosome 11 (*middle*), and scaffold206 (*bottom*). The *thin blue lines* shown under the *first*, *second*, and *third horizontal lines* indicate the positions of female contigs (P3-DDN) specifically mapped by F1 female bulk reads. The *square box* at the bottom indicates alignment depth of reads of F1 female bulk (*red*) and F1 bulk of progeny in which sp16 amplification was not observed (sp16-minus) (*blue*) to scaffold206. *Red triangles* indicate the position of DNA marker sp16

**Fig. 7:**
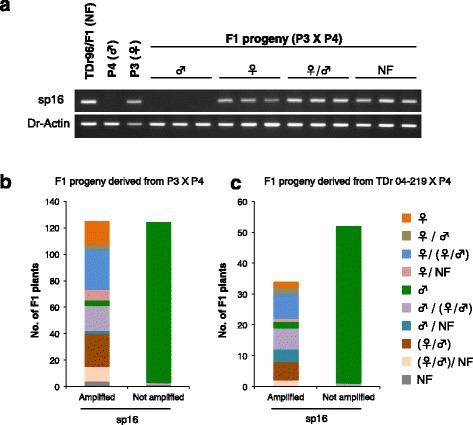
DNA marker sp16 is located in a W-linked region. **a** Results of agarose gel electrophoresis of PCR products amplified by DNA marker sp16 (sp16). *Actin* from *D. rotundata* (Dr-Actin) served as a control to show that template DNA was present for all samples. *NF* non-flowering. **b** Bar graphs showing the correspondence of sp16 genotypes (sp16 PCR product Amplified or Not amplified) with the sex of F1 progeny derived from a cross between P3 and P4 and phenotyped over 2 years (2014 and 2015). Color codes indicate sex manifestation of the plants during the 2-year period, disregarding the yearly order (i.e., plants showing sex changes from male [2014] to female [2015] and female [2014] to male [2015] were combined and are indicated by ♀/♂). Monoecy is indicated by (♀/♂). *NF* non-flowering. **c** The same as **b** but for F1 progeny obtained from a separate cross involving TDr04-219 and P4

**Fig. 8:**
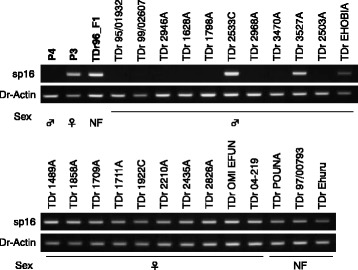
Test of correspondence between sp16 genotypes and sex in 24 *D. rotundata* breeding accessions. Results of agarose gel electrophoresis of PCR products amplified using sp16 DNA markers are shown. Dr-Actin is a control indicating the presence of template DNA for all lines. *NF* non-flowering

As the TDr96_F1 plant never flowered, we were unable to determine its sex based on flower phenotype and therefore could not directly characterize its genotype (ZZ or ZW) at the candidate sex locus. To identify the genomic regions linked to Z and W, we assembled the P3 (female) and P4 (male) genomes de novo using their PE reads with the DISCOVAR De Novo assembler [29], generating P3-DDN (female, N50 = 3.3 kb) and P4-DDN (male, N50 = 2.7 kb) reference sequences (Fig. 6a, Additional file 1: Table S17 and Additional file 2: Figure S20). We separately mapped short reads derived from bulked DNA from 50 male and 50 female F1 progeny (P3 × P4) to P3-DDN and P4-DDN and looked for unique P3-DDN (female) genomic regions (presumably corresponding to the W-linked region) that were specifically mapped by F1 female-bulk reads but not by F1 male-bulk reads. The 1345 regions (sizes from 1 to 129 bp) totaling 15,390 bp conformed to this pattern (Additional file 2: Figure S21). We ordered these fragments by size and found that the N20 value was 42 bp. Conversely, we found only 435 regions (total size 3775 bp) of P4-DDN (male) mapped by the F1-male bulk but not by the F1-female bulk (Additional file 2: Figure S21). The large size difference between female-specific P3-DDN regions (total 15,390 bp) and male-specific P4-DDN regions (total 3775 bp) suggested that the ZW female genome has additional DNA sequences not present in the ZZ male. We hypothesize that the recovery of small male-specific P4-DDN regions may have occurred by chance. We focused on 36 female-specific contigs of P3-DDN that contained DNA fragments larger than 42 bp (Fig. 6b and Additional file 2: Figure S21). When we used the 36 contigs as BLASTN queries against the TDr96_F1 reference genome, 20 were located on scaffold206 (667.8 kb) on pseudo-chromosome 11 (Fig. 6c, Additional file 1: Table S18), suggesting that P3-DDN contigs with female-specific regions were indeed located within the sex-linked region identified by QTL-seq (Fig. 4b). We developed a PCR primer pair for one such P3-DDN contig (Fig. 6b; Female917_flattened_line_87512_3057) harboring female-specific regions; we named this DNA marker sp16. sp16 amplified a PCR fragment in the P3 female parent but not in the P4 male parent (Fig. 7a), demonstrating that this fragment was located in the female-specific region. An sp16 PCR fragment was amplified in TDr96_F1, our reference genome plant (Fig. 7a), suggesting that this individual likely had the ZW genotype. In F1 progeny derived from a P3 × P4 cross, the sp16 fragment was amplified in all female plants, but it failed to be amplified in the majority of male individuals. Furthermore, sp16 fragments were amplified in monoecious as well as non-flowering progeny (Fig. 7a). We monitored flowering in all 249 F1 individuals in two consecutive seasons (2014 and 2015) and found that 194 plants showed consistent sex phenotypes. However, the remaining 55 plants showed changes in sex among male, female, and monoecious (Fig. 7b). Genotyping of all F1 individuals using sp16 revealed a striking pattern: 121 of the 125 plants that were consistent for male over the 2 years showed no PCR amplification of sp16, whereas all plants with the remaining phenotypes showed amplification of sp16 (Fig. 7b). A similar pattern was observed in another F1 family (TDr04-219 × P4) involving the same male parent, P4 (Fig. 7c). We also assayed 24 Guinea yam breeding accessions of known sex using the same marker (Fig. 8). All 10 female accessions, as well as three accessions that did not flower, showed amplification of sp16. Of the 11 male accessions genotyped, eight did not show amplification of sp16, whereas the remaining three did.

We hypothesized that the ZZ genotype stably gives rise to the male phenotype, whereas the ZW genotype results in unstable sex phenotypes; ZW mainly generates the female phenotype, but sometimes monoecious or male phenotypes depending on the environments. Therefore, some individuals of the F1 progeny derived from a cross between P3 and P4 might have been scored as male despite their genotype being ZW, which may have obscured our analysis, resulting in non-zero depth of male DNA bulk within the putative W-region (Fig. 6b). To address this possibility, we selected 50 ZZ plants from the F1 progeny based on their sp16 genotype and bulked and sequenced the DNA (sp16-minus bulk). The sp16-minus bulk reads, as well as female bulk reads, were separately mapped to the combined sequence of the TDr96_F1 reference genome and P4-DDN to identify the female-specific TDr96_F1 genomic region, as described in Fig. 6a. As shown in Fig. 6c and Additional file 2: Figure S20c, d, we successfully delineated the putative W-linked region mapped predominantly with female-only bulk DNA, representing an approximately 161-kb region of scaffold206 on pseudo-chromosome 11. This putative female-specific W-linked region contains ~ 10 predicted genes (Additional file 12).

## Discussion

Molecular markers, such as simple sequence repeats (SSRs), indels, and SNPs, can, for the first time, be developed for various applications in Guinea yam, including linkage mapping, genome-wide association analysis, genomic selection, and MAS. We have already analyzed sequences containing SSR motifs in the genome and identified more than 22,000 candidates that can be used to design primers (Additional file 1: Table S19). We designed primer pairs for 1000 of these sequences and obtained the information necessary for their immediate use in genetic analyses (Additional file 13). SSR markers isolated from one *Dioscorea* species can be transferred to other species [30]. From a practical plant breeding point of view, the sp16 sex-linked marker should prove useful for selecting plantlets for crossing, substantially saving the space and labor required to grow plants and accelerating breeding programs. However, the sex-determination system may vary among *Dioscorea* species (see below), so the transferability of sex-linked DNA markers from *D. rotundata* to other species should be addressed in future studies.

Our identification of the locus underpinning an important trait by QTL-seq, using F1 progeny derived from highly heterozygous parents, opens up new avenues to WGS-based mapping of important traits in crops and tree species for which inbred lines are difficult to obtain and/or generation times are too long, impeding the use of conventional linkage analysis approaches.

Development of DNA markers linked to agronomically important traits and their use for MAS increase the role yam plays in ensuring food security for resource-poor households in Africa and beyond. The *D. rotundata* genome sequences reported here should also contribute to understanding the origin of Guinea yam and its domestication from its wild progenitor species, which are widely distributed in West and Central Africa.

Our results suggest that the Guinea yam sex-determination system involves female heterogamy (male = ZZ, female = ZW). We identified two DNA markers, sp1 (linked to the putative Z-linked region) and sp16 (presumably located within the putative W-linked region, which in TDr96_F1 is presumed to be ZW, and spans only 161 kb). The chromosomes carrying the Z- and W-linked regions are probably not strongly differentiated, and diverged sequences corresponding to Z and W chromosomes were not recovered in our reference genome. Future work should test for structural differences, such as inversions, between the Z- and W-linked regions. Guinea yam sex determination is not, however, a simple genetic system. The consistent maleness of individuals with the ZZ genotype, based on the sp16 sequence, versus occasional maleness of ZW individuals, suggests that maleness is the default phenotype and that the W allele is dominant over Z and can, but does not always, suppress male organ development and feminize the flower. If the feminizing function of the W allele fails in a subset of flowers, the individual will be monoecious. ZW individuals can change sex over time (Fig. 7), indicating that the Z-suppressing function can be affected by the environment. Self-pollination between male and female flowers of ZW monoecious plants could become possible, which may allow inbred lines to be generated, allowing fixation of desired alleles of agronomically important traits. To make it practical, though, we may have to carefully monitor the level of inbreeding depression in *D. rotundata*. Dioecy is the norm in *Dioscorea* species, and previous reports suggest that males are usually the heterogametic sex (XY) in the genus [31, 32]. A genetic study of *D. tokoro* also confirmed an XY male system [19]. *D. tokoro* belongs to the section Stenophora, which is distantly related to the section Enantiophyllum, which contains *D. rotundata* [3]. Our data suggest that the sex-determination system has changed within the genus during the evolution, which could be an interesting topic for future studies. Once the *D. rotundata* sex-determination gene has been isolated, its comparison with another dioecious monocot species such as *Asparagus*, for which the sex-determination gene has been recently isolated [33], would be interesting.

## Conclusions

Here, we sequenced the whole genome (594 Mb) of the dioecious tuber crop Guinea yam (*Dioscorea rotundata*) using a heterozygous individual and anchored the scaffolds to 21 linkage groups to generate pseudo-chromosomes. We exploited the genome sequence to map the sex-determination locus by QTL-seq using BSA of F1 progeny. This analysis revealed a genomic region on pseudo-chromosome 11 tightly linked to femaleness within a female heterogametic (ZZ = male, ZW = female) sex-determination system. This genome sequence will serve as a springboard towards gene mapping and discovery in yam (*Dioscorea* spp.) and genetic improvement of these important yet neglected staple crops.

## Methods

### Plant materials

The TDr96_F1 line used for WGS was selected from F1 progeny obtained from an open-pollinated *D. rotundata* breeding line (TDr96/00629) grown under field conditions in the experimental fields of the International Institute of Tropical Agriculture (IITA) in Nigeria. F1 seeds from TDr96/00629 and those obtained from the cross between the parental lines TDr97/00917 and TDr99/02627 used for RAD-seq were germinated on wet paper towels in darkness at 28 °C. After germination, the seeds were transferred to soil (Sakata Supermix A [34]) and grown at 30 °C with a 16-h/8-h photoperiod in a greenhouse at Iwate Biotechnology Research Institute (IBRC) in Japan. Fresh leaf samples were collected for DNA extraction. Additionally, to resequence the F1 progeny used for QTL-seq analysis, lyophilized leaf samples obtained from plants that were grown and phenotyped under field conditions at IITA were used for DNA extraction.

### Determination of chromosome number and ploidy level

For chromosome observation, root tips of TDr96_F1 plants generated by in vitro propagation of nodal explants were sampled and fixed in acetic acid-alcohol (1:3 ratio) for 24 h without pretreatment. Fixed root tips were stained with a 1% aceto-carmine solution for 24 h. Samples were prepared by the squash method and analyzed under an Olympus BX50 optical microscope (Olympus Optical Co, Ltd., Tokyo, Japan [35]) at 400× magnification.

### Estimation of *D. rotundata* genome size

The genome size of TDr96_F1 (*D. rotundata*) was estimated both by FCM and *k*-mer analyses. FCM analysis was carried out using nuclei prepared from fresh leaf samples of TDr96_F1 and a *japonica* rice (*Oryza sativa* L.) cultivar of known genome size (~380 Mb [36]), which served as an internal reference standard. Nuclei were isolated and stained with propidium iodide (PI) simultaneously and analyzed using a Cell Lab Quanta™ SC Flow Cytometer (Beckman Coulter, CA [37]) following the manufacturer’s protocol. The ratio of G1 peak means [yam (281.7):rice (188.7) = 1.493] was used to estimate the genome size of *D. rotundata* to be ~ 570 Mb (380 Mb × 1.5). *k*-mer analysis-based genome size estimation [10] was performed with TDr96_F1 PE reads with an average size of ~ 230 bp and a total length of 16.77 Gb (16,771,579,510 bp) using ALLPATHS-LG [11]. *k*-mer frequency analysis, with the *k-*mer size set to 25, generated values for *k*-mer coverage (Kc = 25.66) and mean read length (Rl = 228.8), which were used to estimate the genome size of TDr96_F1 to 579 Mb as follows:

$$ \mathrm{Genome}\kern0.17em \mathrm{Size}=\mathrm{Total}\;\mathrm{PE}\;\mathrm{read}\kern0.17em \mathrm{length}\;\left(\mathrm{bp}\right)\div \mathrm{Read}\kern0.17em \mathrm{coverage}\;\left(\mathrm{Rc}\right) $$

$$ \mathrm{Read}\kern0.17em \mathrm{coverage}\;\left(\mathrm{Rc}\right)=\left[k- mer\;\mathrm{coverage}\;\left(\mathrm{Kc}\right)\times \mathrm{Rl}\left]\div \right[\mathrm{Rl}\hbox{--} k\hbox{--} mer\;\mathrm{length}\;\left(\mathrm{k}\right)+1\right] $$

### Whole-genome sequencing

For WGS, genomic DNA was extracted from fresh TDr96_F1 leaf samples using a NucleoSpin Plant II Kit according to the manufacturer’s protocol (Macherey-Nagel GmbH & Co. KG [38]) with slight modifications. Homogenized samples were washed with 0.1 M 4-(2-hydroxyethyl)-1-piperazineethanesulfonic acid (HEPES) buffer to remove contaminating polysaccharides. Just before use, 120 mg polyvinylpyrrolidone (PVP), 90 mg l-ascorbic acid, and 200 μl 2-mercaptoethanol (ME) were added to 10 ml HEPES buffer, and 1 ml of the mixture was used to wash each sample; washing was repeated three times. Additionally, 10 μl 2-ME and 5 μl of 30% polyethylene glycol (PEG)-20000 were added to 1 ml of PL1 buffer (provided with the NucleoSpin Plant II Kit), and twice the recommended volume of buffer (800 μl) was used for cell lysis. Libraries for PE short reads and MP jump reads of various insert sizes including 2, 3, 4, 5, 6, and 8 kb were constructed using an Illumina TruSeq DNA LT Sample Prep Kit and a Nextera Mate Pair Sample Prep Kit, respectively. The PE library was sequenced on the Illumina MiSeq platform, while the MP libraries were sequenced on the HiSeq 2500 platform. Library construction and sequencing of the 20- and 40-kb MP jump sequences were carried out by Eurofins Genomics (Operon [39]) and Lucigen [40], respectively. The 20-kb and 40-kb jump libraries were sequenced on the MiSeq and HiSeq 2500 platforms, respectively. BAC libraries were constructed by Lucigen, and BAC-end sequencing was carried out by Genaris [41] using Sanger sequencing. A total of 30,750 clones corresponding to 3072 Mb of sequence and 5.4× genome coverage were constructed. Of these, 9984 clones were used for BAC-end sequencing, generating a 13.6-Mb sequence in PE fasta format, which was converted to 50-bp PE short reads corresponding to a 0.46-Gb sequence and ~ 0.8× coverage of the estimated 470-Mb *D. rotundata* genome (Additional file 1: Table S2 and Additional file 2: Figure S2).

### De novo assembly

All TDr96_F1 sequence reads in fastq format were filtered for quality using the FASTX-Toolkit version 0.0.13 [42]. For PE reads and MP short jump reads with insert sizes ranging from 2 to 8 kb, only those having sequence reads with a Phred quality score of ≥ 30 (i.e., ≥ 90% of the reads) were retained. Adapter trimming and removal of MP reads with the wrong insert sizes were performed using an in-house pipeline of scripts written in Perl and C++. Quality filtering of the long jump sequences (20-, 40-, and 100-kb insert sizes) was carried out by the suppliers. For the initial de novo assembly, short PE reads and MP jump reads with 2- to 40-kb insert sizes (Additional file 1: Table S2) were assembled using ALLPATHS-LG assembler version R49856 [11]. Further scaffolding of the assembly generated by ALLPATHS-LG was performed using the 100-kb jump MP fastq reads obtained by BAC-end sequencing and the SSPACE PREMIUM 2.3 scaffolding tool with default parameters [13].

### Constructing organelle genome sequences

De novo assembly of the *D. rotundata* mitochondrial genome sequence was performed using mitochondrial DNA isolated from TDr96_F1 leaf samples according to the method of Terachi and Tsunewaki [43] with the following minor modifications. Fresh green leaves (ca. 150 g) were homogenized in 1.5 L of homogenization buffer containing 0.44 M mannitol, 50 mM Tris-HCl (pH 8.0), 3 mM ethylenediaminetetraacetic acid (EDTA), 5 mM 2-ME, 0.1% (w/v) bovine serum albumin, and 0.1% (w/v) PVP. Following DNaseI treatment, the mitochondrial fraction was collected from the interface between 1.30 M and 1.45 M of a sucrose gradient. Mitochondrial DNA was purified by EtBr/CsCl centrifugation at 80,000 rpm for 6 h at 20 °C in a Beckman TLA 100.3 rotor. The DNA band was collected and purified by ethanol precipitation. The resulting mitochondrial DNA (15 ng) was amplified using a REPLI-g Mini Kit (Qiagen, Cat. no. 150023) and used for library construction. The library was sequenced on an Illumina MiSeq sequencer, and the resulting PE reads were assembled de novo using DISCOVAR De Novo [29], generating *D. rotundata* mitochondria contigs. For scaffolding, MP reads with insert sizes of 2, 3, 4, 5, 6, 8, and 20 kb obtained from *D. rotundata* genomic DNA (gDNA) were aligned to the *D. rotundata* mitochondrial contigs. MP reads showing 100% alignment were selected and used for scaffolding of *D. rotundata* mitochondrial contigs by SSPACE [13] (Additional file 2: Figure S5). To reconstruct the *D. rotundata* chloroplast genome sequence, the PE reads of TDr96_F1 were aligned to the recently published *D. rotundata* chloroplast genome sequence [16] (GenBank ID = NC_024170.1) by Burrows-Wheeler alignment (BWA) [44], and chloroplast-derived sequences were identified, amounting to 5,403,420 reads (14.74% of the total size of PE reads generated for TDr96_F1 [Table 1]) matching the assembled 155.4-kb chloroplast genome of *D. rotundata*.

### Evaluation of the completeness of the genomic assembly

To evaluate the completeness of the *D. rotundata* genome assembly, the assembly was checked for the presence of 248 highly conserved core eukaryotic genes [45] using CEGMA version 2.4 with default parameters [14] (Additional file 1: Table S4). To further assess the completeness of the genome, the successor to CEGMA, Benchmarking Universal Single-Copy Orthologs (BUSCO), was used to check for the presence of 956 BUSCOs with version 1.1.b1 [15] using the early access plant dataset (Additional file 1: Table S5).

### Annotation of transposable elements (TEs)

Legacy repetitive sequences, including transposons, were predicted using CENSOR 4.2.29 [46] with the following options: show_simple, nofilter, and mode rough using the Munich Information Center for Protein Sequences (MIPS) Repeat Element Database [47]. Following identification, the repeat elements were classified using mips-REcat [47]. Repetitive sequences were later improved by remodeling using RepeatModeler 1.0.8 [48] and masked with RepeatMasker 4.0.5 [49]. Using the National Center for Biotechnology Information (NCBI) database, one of three other options was used to generate interspersed RepeatModeler-based, interspersed Rebase-based, and Low complexity repeats: “nolow”, “nolow, species Viridiplantae”, and “noint”, respectively. Repeat element content and other statistics were compared between the *D. rotundata* and *A. thaliana* TAIR10 [50], *B. distachyon* v3.1 [51], and *O. sativa* v7_JGI 323 [52] genomes using the RepeatModeled and RepeatMasked references (Table 1).

### RNA-seq

Total RNA was extracted using leaf, stem, flower, and tuber samples collected from a greenhouse-grown TDr96_F1 plant using a Plant RNeasy Kit (Qiagen [53]) with slight modifications. RLC buffer was used for lysis after the addition of 5 μl 30% PEG-20000 and 10 μl 2-ME to 1 ml of buffer. The RNA samples were treated with DNase (Qiagen) to remove contaminating genomic DNA. Two micrograms of total RNA was used to construct complementary DNA (cDNA) libraries using a TruSeq RNA Sample Prep Kit V2 (Illumina) according to the manufacturer’s instructions. The libraries were used for PE sequencing using 2× 100 cycles on the HiSeq 2500 platform in high-output mode. Illumina sequencing reads were filtered by Phred quality score, and reads with a quality score of ≥ 30 (≥90% of reads) were retained (Additional file 1: Table S12). Only one RNA-seq experiment was carried out per tissue/organ (indicated as sample in Additional file 1: Table S12).

### Prediction of protein-coding genes

The legacy gene models were generated previously using the legacy repeat-masked reference genome and three approaches: ab initio, ab initio supported by evidence-based prediction, and evidence-based prediction. The ab initio prediction was carried out with FGENESH 3.1.1 [54]. The ab initio supported by evidence-based prediction was performed with AUGUSTUS 3.0.3 [55] using the maize5 training set and a hint file as the gene model support information. To construct the hint file, TopHat 2.0.11 [56] was used to align RNA-seq reads from tuber, flower (young), leaf (young), stem, leaf (old), and flower (old) samples to the *D. rotundata* reference genome, and Cufflinks 2.2.1 [57] was used to generate gene models from these data. The evidence-based predictions using the Program to Assemble Spliced Alignments (PASA) [58] were generated in a Trinity [59] assembled transcriptome from the RNA-seq data. JIGSAW 3.2.9 [60] was used to select and combine the gene models obtained using the three approaches with the weighting values assigned to the results from FGENESH, AUGUSTUS, and PASA of 10, 3, and 3, respectively. In total, 21,882 consensus gene models were predicted. These gene models were further improved upon using the MAKER [61] pipeline (Additional file 2: Figure S14). Publicly available ESTs and protein sequences from related plant species were aligned to the genome using GMAP [62] and Exonerate 2.2.0 [63], respectively. De novo and reference-guided transcripts were assembled from RNA-seq data from all 18 tissues using Bowtie 1.1.1 [64], Trinity 2.0.6 and SAMtools 1.2.0 [65], and Trinity 2.0.6 and TopHat 2.1.0, respectively. Both sets of assembled transcripts were used to build a comprehensive transcript database using PASA (Additional file 1: Table S13). High-quality non-redundant transcripts from PASA were used to generate a training set for AUGUSTUS 3.1. Gene models were predicted twice using the genome, improved repeat sequences, assembled transcripts, EST and protein alignments, the AUGUSTUS training set, and a legacy set of 21,882 gene models obtained previously using MAKER 2.31.6 [61], retaining all legacy gene models or querying them with new evidence and discarding those that could not be validated. From both MAKER runs, 21,894 and 76,449 gene models were predicted, respectively. A consensus set of gene models from both MAKER outputs was obtained using JIGSAW 3.2.9 [60] at a 1:1 ratio. In total, 26,198 consensus gene models were predicted in the *D. rotundata* genome. The corresponding amino acid sequences were also predicted for these gene models. To confirm these gene models, the RNA-seq reads were aligned to the CDSs (coding sequences) of the predicted genes using BWA [44] with default parameters. Accordingly, 85.8% of the gene models could be aligned by at least a single RNA-seq read. Functional annotation of the amino acid sequences was performed using the in-house pipeline, AnnotF, which compares Blast2GO [66] and InterProScan [67] functional terms.

### Comparative genomics

Pairwise orthology relationships were determined with Inparanoid [68–70] using the longest protein-coding isoform for each gene in *Arabidopsis thaliana* (TAIR10) [50], *Oryza sativa japonica* (v7.0) [52], *Brachypodium distachyon* (v3.1) [71], *Musa acuminata* (v2) [72], *Elaeis guineensis* (EG5) [73], and *Phoenix dactylifera* (DPV01) [74]. Orthology clusters across all seven species were determined using Multiparanoid [75]. Sequences for the 12 classes of lectins were obtained from UniProt [76] for the proteomes of *A. thaliana* (up000006548), *B. distachyon* (up000008810), and *O. sativa* (up000059680). Protein alignments for B-lectin class protein sequences from all three of these species and *D. rotundata* were generated using the program Multiple Alignment using Fast Fourier Transform (MAFFT) [77]. Maximum likelihood trees were constructed based on the concatenated alignments of all 378 B-lectin proteins using RAxML [78] 8.0.2 with 1000 bootstraps. Enrichment of tuber-specific genes was detected using TopHat 2.1.0 to align RNA-seq data from each of the 12 tissues to the genome, with one biological replicate for each tissue. HTSeq 0.6.1 [79] was used to generate raw counts. Then the Bioconductor package DESeq2 1.14.1 [80] was used to compare raw counts of the three tuber tissues against all the other nine tissues (Additional file 1: Table S12) to determine tuber-enriched gene expression based on a log2 fold change > 0 and Benjamini–Hochberg [25] adjusted *P* value < 0.05.

Gene enrichment analysis of orthology clusters was performed with GOATOOLS [81], using the Holm significance test, and the false discovery rate was adjusted using the Benjamini–Hochberg procedure [25]. The list of enriched genes was filtered for redundant Gene Ontology (GO) terms using REVIGO [82]. For the species phylogeny, protein alignments for each gene with a 1:1 orthologous relationship across all monocot species were generated with MAFFT using the longest protein isoform. Maximum likelihood trees were constructed based on the concatenated alignments of 2381 orthologous protein-coding genes using RAxML 8.2.8 [78] with a JTT + Γ model and 1000 bootstraps.

SynMAP [83] using BLASTZ [84] alignments, DAGchainer [85] (options -D 30 and -A 2), and no merging of syntenic blocks were used as part of the CoGe platform [86] to identify syntenic blocks between the hard-masked pseudo-chromosomes of *D. rotundata* and scaffolds/contigs of *Oryza sativa japonica* (A123v1.0), *Spirodela polyrhiza* (v0.01), and *Phoenix dactylifera* L. (v3). A syntenic path assembly was then carried out on each of the same three species in SynMap using synteny between the scaffolds/contigs against *D. rotundata* pseudo-molecules. The syntenic path assembly is a reference-guided assembly that uses the synteny between two species to order and orientate contigs. This approach highlights regions of conservation that were otherwise too shuffled to be clearly observed. Self-self synteny analysis of *D. rotundata* pseudo-chromosomes was carried out using SynMap Last alignments with default parameters and syntenic gene pair synonymous rate change calculated by CodeML [87].

### RAD-based linkage mapping and scaffold anchoring

RAD-seq was performed as previously described [88] with a minor modification. Genomic DNA was digested with the restriction enzymes *Pac*I and *Nla*III to prepare libraries used to generate PE reads by Illumina HiSeq 2500 (Additional file 2: Figure S6). Approximately 822.7-Mb and 250.4-Mb sequence reads covering 22.9% and 5.3% of the estimated 504-Mb *D. rotundata* genome sequence, excluding gap regions, at average depths of 7.2× and 9.8× were generated for the parental lines and F1 individuals, respectively (Additional file 2: Figure S7).

#### Library preparation and sequencing

For library construction, 1 μg DNA obtained from the two parental lines (TDr97/00917-P1 and TDr99/02627-P2) and the 150 F1 individuals was digested with *Pac*I, which recognizes 5’-TTAATTAA-3’, and a biotinylated adapter-1 was ligated to the digested DNA fragments. The adapter-1-ligated DNA fragments were digested with a second enzyme, *Nla*III (5’-CATG-3’). After collecting the biotinylated fragments using streptavidin-coated magnetic beads, adapter-2 was ligated to the *Nla*III-digested ends. The adapter-ligated DNA was amplified using primers containing sample-specific index sequences, adapter-1 (F) and adapter-2 (R) sequences, and sequences corresponding to the P7 and P5 primers for Illumina sequencing library preparation (Additional file 2: Figure S6). The PCR products were pooled in equal proportions, purified, and subjected to PE sequencing on the Illumina HiSeq 2500 platform. Detailed information about the primers (P7 and P5) used for Illumina library preparation are given in Additional file 1: Table S20.

#### Identification of parental line-specific heterozygous markers

RAD-tags were aligned to the *D. rotundata* reference genome using BWA [44]. The aligned data were converted to SAM/BAM files using SAMtools [65], and the RAD-tags with mapping quality < 60 or containing insertions/deletions in the alignment data were excluded from analysis. Low mapping positions including those with only a single RAD-tag and a mapping quality score of < 30 were also excluded. SNP-index values [28] were calculated at all SNP positions. For linkage mapping, two types of heterozygous markers (SNP-type and presence/absence-type) were identified (Additional file 2: Figure S8). The SNP-type heterozygous markers were defined based on SNP-index patterns of the parental line RAD-tags. For example, positions with SNP-index values ranging from 0.2 to 0.8 in P1 but homozygous in P2 with SNP-index values of either 0 or 1 were defined as P1-specific heterozygous SNPs. A similar procedure was followed to identify P2-specific heterozygous SNP markers. The selected markers were filtered using depth information at all positions. To increase the accuracy of the selected markers, their segregation (1:1 ratio) was confirmed in 150 F1 individuals obtained from a cross between P1 and P2. If the segregation ratio was out of the confidence interval (*P* < 0.05) hypothesized by the binomial distribution, *B*(*n* = number of individuals, *P* = 0.5), the markers were excluded from further analysis. Only one marker was selected per 10-kb interval based on the number of F1 individuals represented and tag coverage. A total of 1105 and 990 P1- and P2-heterozygous SNP markers were selected, respectively (Additional file 1: Table S7).

The presence/absence-type markers were defined based on the alignment depth of parental line RAD-tags. First, genomic positions that could be aligned by RAD-tags from only one of the parental lines were identified. Additionally, aligned tags should be heterozygous for that particular region. Similar to the SNP-type markers, the segregation patterns of candidate presence/absence-type markers in the F1 progeny were confirmed, and only those that segregated at a 1:1 ratio (as confirmed by binomial distribution filter) were retained. In the F1 progeny, positions with sequencing depths of ≥ 3 and 0 were defined as heterozygous and homozygous, respectively. For a given candidate position/marker, if the number of F1 individuals defined as homozygous or heterozygous was less than 120, the marker was excluded from further analysis. Only one heterozygous position was selected as a marker within a given 10-kb interval. In total, 221 and 282 positions were selected as P1- and P2-specific presence/absence-type heterozygous markers, respectively (Additional file 1: Table S7).

#### Linkage mapping

To developing parental line-specific linkage maps, P1-Map and P2-Map, recombination fraction (*rf*) values between all pairs of markers on a given scaffold were calculated for both parents using the recombination pattern of the 150 F1 individuals. To minimize incorrect mapping, scaffolds were divided at positions where *rf* values exceeded 0.25 from the initial marker position (Additional file 2: Figure S9). Only two flanking (distal) markers per scaffold were selected, corresponding to 477 and 493 P1- and P2-specific markers, respectively. These markers were used to develop P1 and P2 linkage maps according to the pseudo-testcross method [18] using the backcross model of R/qtl [89]. Due to the use of the pseudo-testcross method, the initial maps contained both the coupling and repulsion-type markers. Consequently, the genetic distance in linkage groups was larger than expected. To avoid the effect of repulsion-type markers when calculating genetic distances, these markers were converted to coupling-type markers. If a marker showed a high logarithm of odds (LOD) score and an *rf* value > 0.5, it was defined as repulsion type and was therefore converted to the coupling-type genotype. This conversion was carried out gradually by changing the threshold of the LOD score from 10 to 5, and then to 3. After converting all repulsion markers to coupling markers, linkage maps were developed using markers showing LOD score > 3 and *rf* value < 0.25. Accordingly, a total of 21 and 23 linkage groups, each with a minimum of three markers, were generated for P1- and P2-Maps, respectively (Additional file 1: Table S8 and Additional file 2: Figure S10).

#### Anchoring scaffolds

To develop chromosome-scale pseudo-molecules, TDr96_F1 scaffold sequences were anchored onto the two parental-specific linkage maps using the selected RAD markers. To combine the two maps, the number of scaffolds shared between all possible linkage group (LG) pairs corresponding to the two maps was determined (Additional file 2: Figures S11, S12). LG pairs that shared the largest number of scaffolds were combined using the same scaffolds. Each combined LG represented a pseudo-chromosome, which was designated/numbered according to the P1-Map LG designation (see Fig. 2 and Additional file 2: Figure S11). After combining the two maps to construct the pseudo-chromosomes, P1- and P2-specifc scaffolds were ordered according to their original order in their respective LGs. If the order of scaffolds could not be decided because the order was similar in both the P1- and P2-Maps, the order in P1-LG was adopted (Fig. 2). Finally, the ordered scaffolds were connected by 1000 nucleotides of “N” into a single fasta file for each pseudo-chromosome (Additional file 2: Figure S12).

### QTL-seq analysis

DNA samples obtained from the two parental lines, TDr97/00917 (P3, female) and TDr97/00777 (P4, male), as well as samples pooled in equal amounts from 50 male (male-bulk) and 50 female (female-bulk) F1 individuals obtained from the cross between P3 and P4 were subjected to WGS. Libraries for sequencing were constructed from 1-μg DNA samples with a TruSeq DNA PCR-Free LT Sample Preparation Kit (Illumina) and were sequenced via 76 cycles on the Illumina NextSeq 500 platform. Short reads in which more than 20% of sequenced nucleotides exhibited a Phred quality score of < 20 were excluded from further analysis. To perform QTL-seq analysis of F1 progeny, two types of analyses are required. In the first analysis, the SNP index and ΔSNP index are calculated at P4-specific heterozygous positions. The second analysis is performed using P3-specific heterozygous positions. To identify P4-specific heterozygous positions, the P3 “reference sequence” was first developed by aligning P3 reads to the reference genome sequence of *D. rotundata* and replacing nucleotides of the *D. rotundata* reference genome sequence with nucleotides of P3 at all SNP positions showing an SNP index of 1 (Additional file 2: Figure S17c). SNP detection, calculation of SNP index, and replacement of SNPs were carried out via step 2 of QTL-seq pipeline version 1.4.4 [90]. Short reads obtained from both the male and female parents were then aligned to the “reference sequence” and heterozygous SNP positions between the two were extracted. A SNP was defined as heterozygous if the same position showed an SNP-index value ranging from 0.4 to 0.6 in one parent and a value of 0 in the second parent. Of the selected markers/positions, only those having enough depth in both parents (>15) were used for analysis of SNP-index values in the bulk-sequenced samples. P3-specific heterozygous positions were identified similarly using the P4 “reference sequence.”

After identifying P4- and P3-specific heterozygous positions, the Illumina reads from the two bulk-sequenced samples (male and female bulks) were aligned to the reference sequences using BWA [44] and subjected to Coval filtering [91] as previously described. When the P3 reference sequence was used for alignment, the SNP-index values were calculated only at all of the P4-specific heterozygous positions. By contrast, when the P4 reference sequence was used for alignment, the SNP-index values were calculated only at the P3-specific heterozygous positions. In both cases, positions with shallow depth (< 6) in either of the two samples were excluded from analysis. The ∆SNP index was calculated by subtracting the SNP-index values of the male bulk from those of the female bulk. To generate confidence intervals of the SNP-index value, an in silico test simulating the application of QTL-seq to DNA bulked from 50 randomly selected F1 individuals was performed as described previously [28] (Additional file 2: Figure S22). The simulation test was repeated 10,000 times depending on the alignment depth of short reads to generate confidence intervals. These intervals were plotted for all SNP positions analyzed. Finally, sliding window analysis was applied to SNP-index, ∆SNP-index, and confidence interval plots with a 1-Mb window size and a 50-kb increment to generate SNP-index graphs (Additional file 2: Figure S18).

### Identification of putative W-region by de novo assembly of female and male parental genomes and mapping of bulked DNA from female and male F1 progeny

DNA samples obtained from the two parental lines, TDr97/00917 (P3, female) and TDr97/00777 (P4, male), were separately subjected to de novo assembly. Libraries for sequencing were prepared with a TruSeq DNA PCR-Free LT Sample Preparation Kit (Illumina) and were sequenced for 251 cycles on the Illumina MiSeq platform. Contigs were generated using the DISCOVAR De Novo assembler [29], resulting in P3-DDN and P4-DDN, respectively. Separately, whole-genome resequencing of bulked DNA was performed on bulked DNA samples obtained from 50 female F1 (Female-bulk.fastq) and 50 male F1 (Male-bulk.fastq) progeny, all derived from a cross between P3 and P4. Two reference sequences, P3-DDN and P4-DDN, were combined to generate P3-DDN/P4-DDN. Short reads from the female and male bulks were separately mapped to P3-DDN/P4-DDN using the alignment software BWA [44]. After mapping, the MAPQ scores of the aligned reads were obtained. Under our conditions, if a short read was mapped to a unique position of the reference sequence, the MAPQ score was 60, whereas if the read was mapped to multiple positions, MAPQ was < 60. Since two reference sequences (P3-DDN and P4-DDN) were fused to generate P3-DDN/P4-DDN, most genomic regions were represented twice. Therefore, most short reads mapped to two or more positions, leading to a MAPQ score < 60. The reads that mapped to the P3-DDN/P4-DDN with MAPQ = 60 were judged to be located in either P3- or P4-specific genomic regions. After finding these P3- or P4-specific genomic regions, the depth of short reads that covered the regions for Female-bulk.fastq and Male-bulk.fastq, respectively, was evaluated. If the depth of Female-bulk.fastq was high and the depth of Male-bulk.fastq was 0 or close to 0, such genomic regions were retained as putative W-regions (Fig. 6 and Additional file 2: Figure S20).

### DNA markers linked to sex

The primer sequences used for amplification of sex-linked markers sp1 and sp16, as well as the control *Actin* gene fragment (Dr-Actin), were as follows:

PCR primers for sp1 fragment:

- sp1-F; 5’-GATCTGGCTTCCTCCATCTTG-3’
- sp1-R; 5’-GCTTGGGTGGTTAGTTTATTGTTTG-3’
- PCR primers for sp16 fragment:
- sp16-F; 5’-AATGTGTTTAACAGGGTGAATTC-3’
- sp16-R; 5’-GAATTCAGCCGAATATACTTATTC-3’
- PCR primers for Dr-Actin gene fragment:
- Dr-Actin-F; 5’-CAGGGAAAAGATGACCCAAATC-3’
- Dr-Actin-R; 5’-CCATCACCAGAATCCAGCAC-3’

PCR was performed using the following conditions: 30 cycles of 98 °C for 10 s, 55 °C for 30 s, and 72 °C for 1 min. For CAPS analysis of the sp1 marker, the amplified DNA was digested with *Eco*RI. All PCR products were electrophoresed on 1.5% agarose gels.

### Identification of SSR markers

Approximately 4,932,582 bp simple sequence repeat (SSR) motif-containing sequences were predicted in the *D. rotundata* genome. Within this region, SSR sequences with enough flanking regions were identified and evaluated for use in primer design. Accordingly, 134,101 SSR-containing sequences, excluding those with single base repeats, were identified. The SSR information for these sequences was analyzed using GMATo [92] version 1.2 Build 20130106 with the following parameters: m (minimum motif length) = 2, x (maximum motif length) = 10, and r (minimum repeat number) = 10. The necessary information was obtained for 22,164 SSR-containing sequences in the assembled genome, 12,724 (57.4%) of which were anchored to the genetic map (Additional file 1: Table S19). Primer pairs were designed for 1000 of these sequences using Primer3 [93] software release 2.3.6 with the following parameters: product size = 100–500, primer length = 18–22 bp (optimum 20 bp), GC content = 40–60% (optimum 50%), and Tm = 57–63 (optimum = 60.0).

## Additional files

Additional file 1: Tables S1–S20. Supplementary data including world yam production statistics [94] (**Table S1**), summary of genome sequence reads (**Table S2**), summary of genome assembly (**Table S3**), CEGMA result [95] (**Table S4**), BUSCO result (**Table S5**), summary of chloroplast genome assembly (**Table S6**), data on RAD-based linkage analysis and anchoring of scaffolds (**Tables S7–S9**), validation of genome assembly (**Tables S10, S11**), summary of RNA-seq data (**Table S12**), summary of assembly of transcripts (**Table S13**), number of lectin genes in the genomes of *D. rotundata* and three species (**Table S14**), segregation of sex in F1 derived from a cross between two accessions (**Table S15**), summary statistics of bulk DNA sequencing and its analysis (**Tables S16, S17**), BLAST result of female-specific region against TDr96_F1 reference genome (**Table S18**), summary of simple sequence repeats (**Table S19**), sequences of primers used for RAD-seq (**Table S20**). (PPTX 137 kb)

Additional file 2: Figures S1–S22. Supplementary figures including a summary of world yam production and photos of yam markets in West Africa (**Figure S1**), summary of BAC-end sequencing used for genome scaffolding (**Figure S2**), summary of k-mer analysis of Guinea yam genome (**Figure S3**), flowchart of Guinea yam genome assembly (**Figure S4**), summary of Guinea yam mitochondrial genome (**Figure S5**), flowchart of RAD-seq for linkage analysis (**Figure S6**), summary of RAD-seq analysis (**Figure S7**), summary of RAD-seq DNA markers used for linkage mapping and anchoring of scaffolds (**Figure S8**), procedure of linkage analysis and split of scaffolds depending on recombination fraction between RAD markers (**Figure S9**), RAD-seq-based linkage maps of *D. rotundata* generated by pseudo-testcross method (**Figure S10**), a matrix showing scaffolds shared between two linkage groups generated for two parents (**Figure S11**), schematic diagram for developing physical map of *D. rotundata* (**Figure S12**), frequency of distances of BAC-end sequences in the genome (**Figure S13**), scheme showing pipeline of genome annotation of *D. rotundata* (**Figure S14**), self-self syntenic dot plot of *D. rotundata* pseudo-chromosomes (**Figure S15**), SyMAP dot plot analysis of whole genome synteny between three monocot species (**Figure S16**), explanation of QTL-seq analysis to identify sex-linked genome regions in *D. rotundata* (**Figure S17**), QTL-seq results (**Figure S18**), sp1 DNA marker genotypes of F1 progeny and their association with sex (**Figure S19**), explanation of method for identification of putative W-region of D. rotundata genome (**Figure S20**), identification of female- and male-specific genomic regions (**Figure S21**), method of calculation of confidence interval of QTL-seq analysis (**Figure S22**). (PPTX 15700 kb)

Additional file 3: Supplemental dataset S1. List of the 26,198 protein coding genes predicted in the *D. rotundata* genome. (XLSX 1480 kb)

Additional file 4: Supplemental dataset S2. Gene orthology between four angiosperms showing presence, absence, and duplication between species. (XLSX 1250 kb)

Additional file 5: Supplemental dataset S3. Functional annotation of *D. rotundata* genes conserved between *D. rotundata* and at least one other angiosperm (*A. thaliana*, *B. distachyon*, and *O. sativa*). (XLSX 639 kb)

Additional file 6: Supplemental dataset S4. Gene orthology between seven angiosperms showing presence, absence, and duplication between species. (XLSX 2360 kb)

Additional file 7: Supplemental dataset S5. Functional annotation of *D. rotundata* genes conserved between *D. rotundata* and at least one other angiosperm (*A. thaliana*, *B. distachyon*, *O. sativa*, *E. guineensis*, *P. dactylifera*, and *M. acuminata*). (XLSX 356 kb)

Additional file 8: Supplemental dataset S6. Functional annotation of *D. rotundata* genes with no orthologous genes found in *A. thaliana*, *B. distachyon*, *O. sativa*, *E. guineensis*, *P. dactylifera*, and *M. acuminata*. (XLSX 720 kb)

Additional file 9: Supplemental dataset S7. Non-redundant Gene Ontology terms for 2795 genes significantly (after FDR correction) enriched in *D. rotundata* with orthologous genes identified in *A. thaliana*, *B. distachyon*, *O. sativa*, *E. guineensis*, *P. dactylifera*, and *M. acuminata*. (XLSX 35 kb)

Additional file 10: Supplemental dataset S8. Non-redundant Gene Ontology terms for 11,348 genes significantly (after FDR correction) enriched in *D. rotundata* with no orthologous genes identfied in *A. thaliana*, *B. distachyon*, *O. sativa*, *E. guineensis*, *P. dactylifera*, and *M. acuminata*. (XLSX 9 kb)

Additional file 11: Supplemental dataset S9. Top 50 highest expressed genes observed to be enriched in tuber. (XLSX 59 kb)

Additional file 12: Supplemental dataset S10. List of genes predicted within the female-specific (W-linked) region on pseudo-chromosome 11 identified by QTL-seq. (XLSX 464 kb)

Additional file 13: Supplemental dataset S11. New SSR markers developed from D. rotundata genome sequence. (XLSX 126 kb)
