# Supplementary material for: Genome sequencing of the staple food crop white Guinea yam enables the development of a molecular marker for sex determination
Source: BMC Biol. 2017 Sep 19;15:86. doi: 10.1186/s12915-017-0419-x (PMC5604175; doi:10.1186/s12915-017-0419-x)
Supplement: Supplementary file 1 — Supplementary data including world yam production statistics [94] (Table S1), summary of genome sequence reads (Table S2), summary of genome assembly (Table S3), CEGMA result [95] (Table S4), BUSCO result (Table S5), summary of chloroplast genome assembly (Table S6), data on RAD-based linkage analysis and anchoring of scaffolds (Tables S7–S9), validation of genome assembly (Tables S10, S11), summary of RNA-seq data (Table S12), summary of assembly of transcripts (Table S13), number of lectin genes in the genomes of D. rotundata and three species (Table S14), segregation of sex in F1 derived from a cross between two accessions (Table S15), summary statistics of bulk DNA sequencing and its analysis (Tables S16, S17), BLAST result of female-specific region against TDr96_F1 reference genome (Table S18), summary of simple sequence repeats (Table S19), sequences of primers used for RAD-seq (Table S20). (PPTX 137 kb) [file 12915_2017_419_MOESM1_ESM.pptx]

## Slide 1
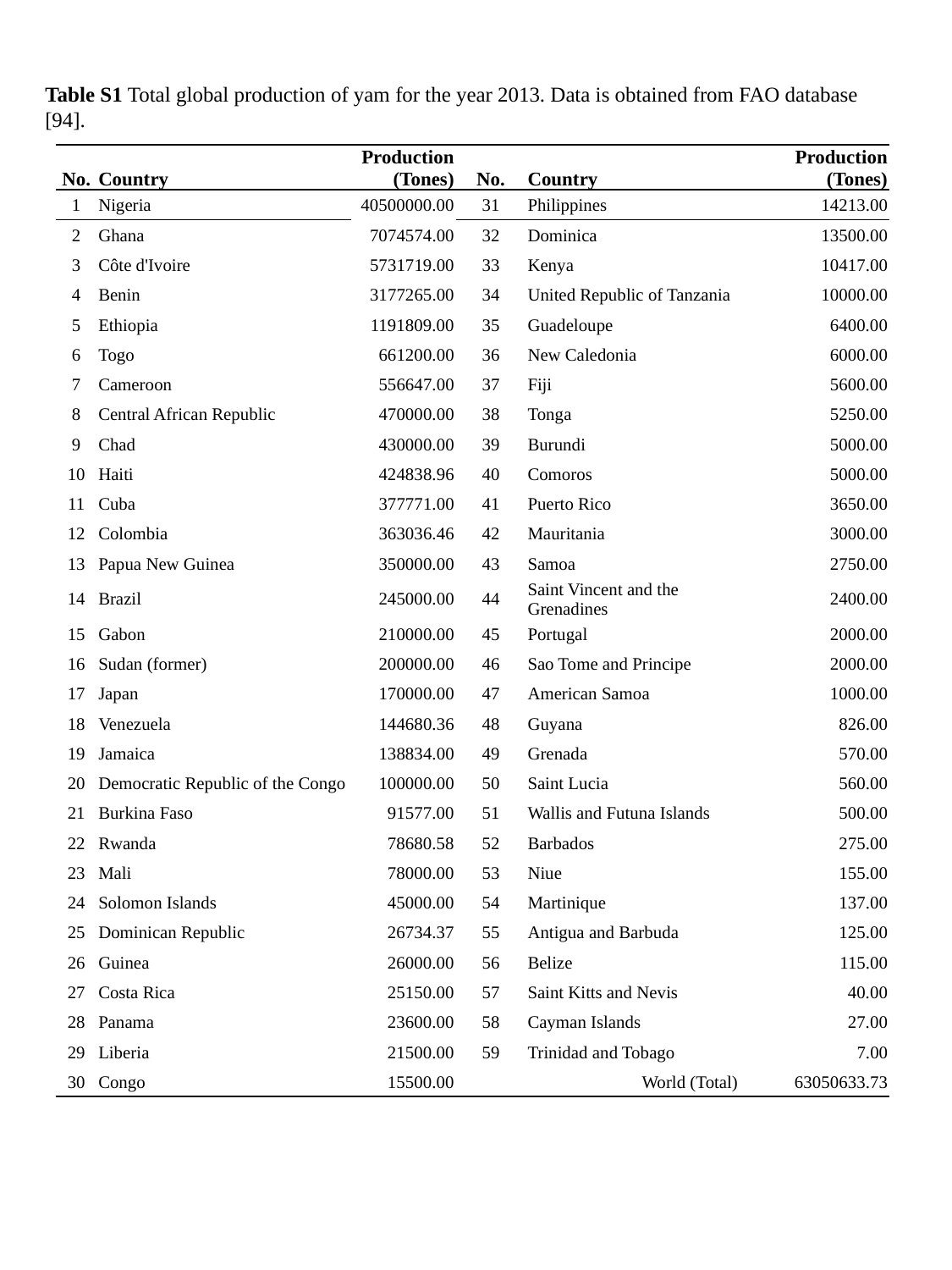

Table S1 Total global production of yam for the year 2013. Data is obtained from FAO database [94].
| No. | Country | Production (Tones) | No. | Country | Production (Tones) |
| --- | --- | --- | --- | --- | --- |
| 1 | Nigeria | 40500000.00 | 31 | Philippines | 14213.00 |
| 2 | Ghana | 7074574.00 | 32 | Dominica | 13500.00 |
| 3 | Côte d'Ivoire | 5731719.00 | 33 | Kenya | 10417.00 |
| 4 | Benin | 3177265.00 | 34 | United Republic of Tanzania | 10000.00 |
| 5 | Ethiopia | 1191809.00 | 35 | Guadeloupe | 6400.00 |
| 6 | Togo | 661200.00 | 36 | New Caledonia | 6000.00 |
| 7 | Cameroon | 556647.00 | 37 | Fiji | 5600.00 |
| 8 | Central African Republic | 470000.00 | 38 | Tonga | 5250.00 |
| 9 | Chad | 430000.00 | 39 | Burundi | 5000.00 |
| 10 | Haiti | 424838.96 | 40 | Comoros | 5000.00 |
| 11 | Cuba | 377771.00 | 41 | Puerto Rico | 3650.00 |
| 12 | Colombia | 363036.46 | 42 | Mauritania | 3000.00 |
| 13 | Papua New Guinea | 350000.00 | 43 | Samoa | 2750.00 |
| 14 | Brazil | 245000.00 | 44 | Saint Vincent and the Grenadines | 2400.00 |
| 15 | Gabon | 210000.00 | 45 | Portugal | 2000.00 |
| 16 | Sudan (former) | 200000.00 | 46 | Sao Tome and Principe | 2000.00 |
| 17 | Japan | 170000.00 | 47 | American Samoa | 1000.00 |
| 18 | Venezuela | 144680.36 | 48 | Guyana | 826.00 |
| 19 | Jamaica | 138834.00 | 49 | Grenada | 570.00 |
| 20 | Democratic Republic of the Congo | 100000.00 | 50 | Saint Lucia | 560.00 |
| 21 | Burkina Faso | 91577.00 | 51 | Wallis and Futuna Islands | 500.00 |
| 22 | Rwanda | 78680.58 | 52 | Barbados | 275.00 |
| 23 | Mali | 78000.00 | 53 | Niue | 155.00 |
| 24 | Solomon Islands | 45000.00 | 54 | Martinique | 137.00 |
| 25 | Dominican Republic | 26734.37 | 55 | Antigua and Barbuda | 125.00 |
| 26 | Guinea | 26000.00 | 56 | Belize | 115.00 |
| 27 | Costa Rica | 25150.00 | 57 | Saint Kitts and Nevis | 40.00 |
| 28 | Panama | 23600.00 | 58 | Cayman Islands | 27.00 |
| 29 | Liberia | 21500.00 | 59 | Trinidad and Tobago | 7.00 |
| 30 | Congo | 15500.00 | | World (Total) | 63050633.73 |

## Slide 2
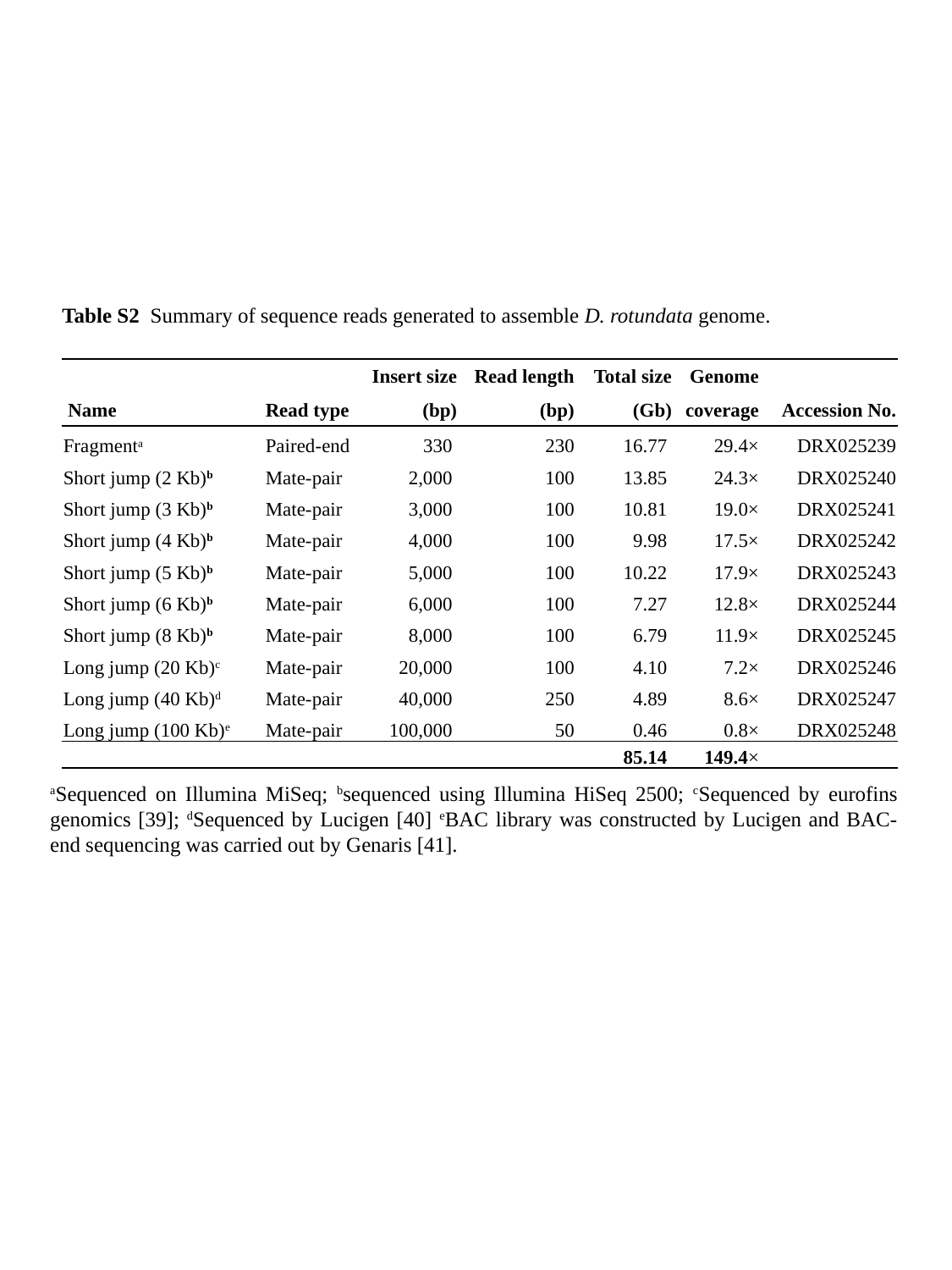

Table S2 Summary of sequence reads generated to assemble D. rotundata genome.
| | | Insert size | Read length | Total size | Genome | |
| --- | --- | --- | --- | --- | --- | --- |
| Name | Read type | (bp) | (bp) | (Gb) | coverage | Accession No. |
| Fragmenta | Paired-end | 330 | 230 | 16.77 | 29.4× | DRX025239 |
| Short jump (2 Kb)b | Mate-pair | 2,000 | 100 | 13.85 | 24.3× | DRX025240 |
| Short jump (3 Kb)b | Mate-pair | 3,000 | 100 | 10.81 | 19.0× | DRX025241 |
| Short jump (4 Kb)b | Mate-pair | 4,000 | 100 | 9.98 | 17.5× | DRX025242 |
| Short jump (5 Kb)b | Mate-pair | 5,000 | 100 | 10.22 | 17.9× | DRX025243 |
| Short jump (6 Kb)b | Mate-pair | 6,000 | 100 | 7.27 | 12.8× | DRX025244 |
| Short jump (8 Kb)b | Mate-pair | 8,000 | 100 | 6.79 | 11.9× | DRX025245 |
| Long jump (20 Kb)c | Mate-pair | 20,000 | 100 | 4.10 | 7.2× | DRX025246 |
| Long jump (40 Kb)d | Mate-pair | 40,000 | 250 | 4.89 | 8.6× | DRX025247 |
| Long jump (100 Kb)e | Mate-pair | 100,000 | 50 | 0.46 | 0.8× | DRX025248 |
| | | | | 85.14 | 149.4× | |
aSequenced on Illumina MiSeq; bsequenced using Illumina HiSeq 2500; cSequenced by eurofins genomics [39]; dSequenced by Lucigen [40] eBAC library was constructed by Lucigen and BAC-end sequencing was carried out by Genaris [41].

## Slide 3
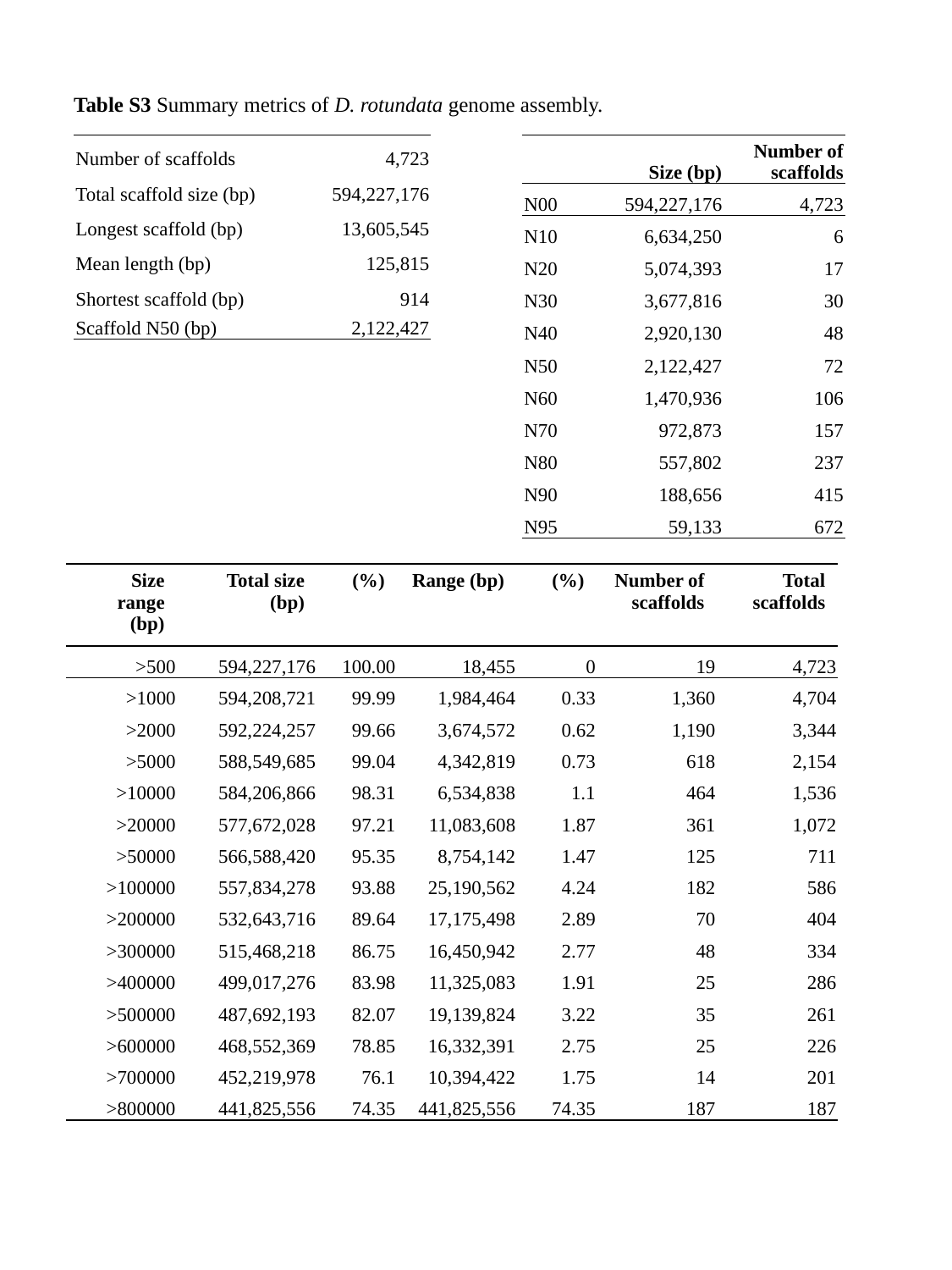

Table S3 Summary metrics of D. rotundata genome assembly.
| Number of scaffolds | 4,723 |
| --- | --- |
| Total scaffold size (bp) | 594,227,176 |
| Longest scaffold (bp) | 13,605,545 |
| Mean length (bp) | 125,815 |
| Shortest scaffold (bp) | 914 |
| Scaffold N50 (bp) | 2,122,427 |
| | Size (bp) | Number of scaffolds |
| --- | --- | --- |
| N00 | 594,227,176 | 4,723 |
| N10 | 6,634,250 | 6 |
| N20 | 5,074,393 | 17 |
| N30 | 3,677,816 | 30 |
| N40 | 2,920,130 | 48 |
| N50 | 2,122,427 | 72 |
| N60 | 1,470,936 | 106 |
| N70 | 972,873 | 157 |
| N80 | 557,802 | 237 |
| N90 | 188,656 | 415 |
| N95 | 59,133 | 672 |
| Size range (bp) | Total size (bp) | (%) | Range (bp) | (%) | Number of scaffolds | Total scaffolds |
| --- | --- | --- | --- | --- | --- | --- |
| >500 | 594,227,176 | 100.00 | 18,455 | 0 | 19 | 4,723 |
| >1000 | 594,208,721 | 99.99 | 1,984,464 | 0.33 | 1,360 | 4,704 |
| >2000 | 592,224,257 | 99.66 | 3,674,572 | 0.62 | 1,190 | 3,344 |
| >5000 | 588,549,685 | 99.04 | 4,342,819 | 0.73 | 618 | 2,154 |
| >10000 | 584,206,866 | 98.31 | 6,534,838 | 1.1 | 464 | 1,536 |
| >20000 | 577,672,028 | 97.21 | 11,083,608 | 1.87 | 361 | 1,072 |
| >50000 | 566,588,420 | 95.35 | 8,754,142 | 1.47 | 125 | 711 |
| >100000 | 557,834,278 | 93.88 | 25,190,562 | 4.24 | 182 | 586 |
| >200000 | 532,643,716 | 89.64 | 17,175,498 | 2.89 | 70 | 404 |
| >300000 | 515,468,218 | 86.75 | 16,450,942 | 2.77 | 48 | 334 |
| >400000 | 499,017,276 | 83.98 | 11,325,083 | 1.91 | 25 | 286 |
| >500000 | 487,692,193 | 82.07 | 19,139,824 | 3.22 | 35 | 261 |
| >600000 | 468,552,369 | 78.85 | 16,332,391 | 2.75 | 25 | 226 |
| >700000 | 452,219,978 | 76.1 | 10,394,422 | 1.75 | 14 | 201 |
| >800000 | 441,825,556 | 74.35 | 441,825,556 | 74.35 | 187 | 187 |

## Slide 4
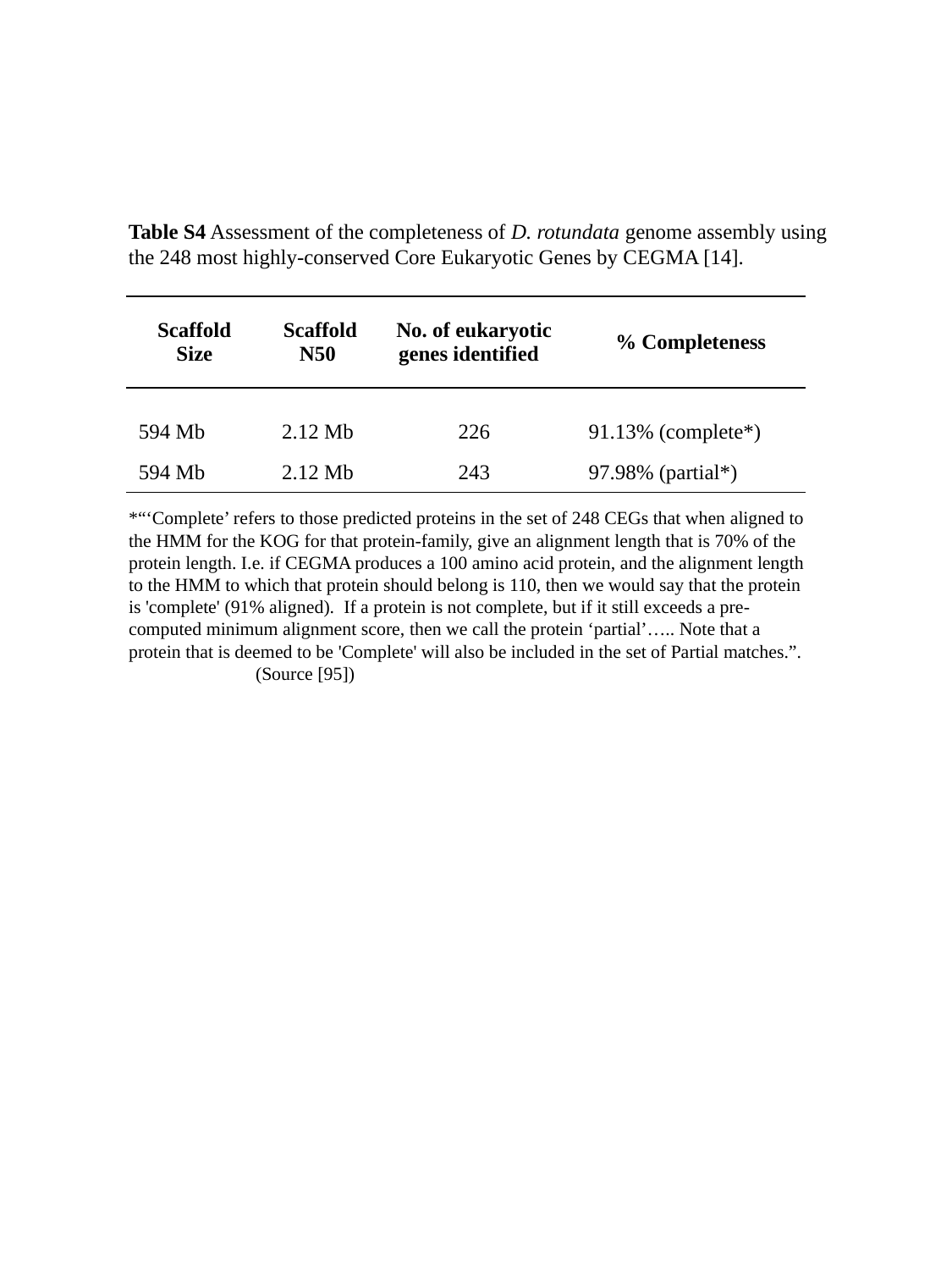

Table S4 Assessment of the completeness of D. rotundata genome assembly using the 248 most highly-conserved Core Eukaryotic Genes by CEGMA [14].
| Scaffold Size | Scaffold N50 | No. of eukaryotic genes identified | % Completeness |
| --- | --- | --- | --- |
| 594 Mb | 2.12 Mb | 226 | 91.13% (complete\*) |
| 594 Mb | 2.12 Mb | 243 | 97.98% (partial\*) |
*“‘Complete’ refers to those predicted proteins in the set of 248 CEGs that when aligned to the HMM for the KOG for that protein-family, give an alignment length that is 70% of the protein length. I.e. if CEGMA produces a 100 amino acid protein, and the alignment length to the HMM to which that protein should belong is 110, then we would say that the protein is 'complete' (91% aligned). If a protein is not complete, but if it still exceeds a pre-computed minimum alignment score, then we call the protein ‘partial’….. Note that a protein that is deemed to be 'Complete' will also be included in the set of Partial matches.”.
	(Source [95])

## Slide 5
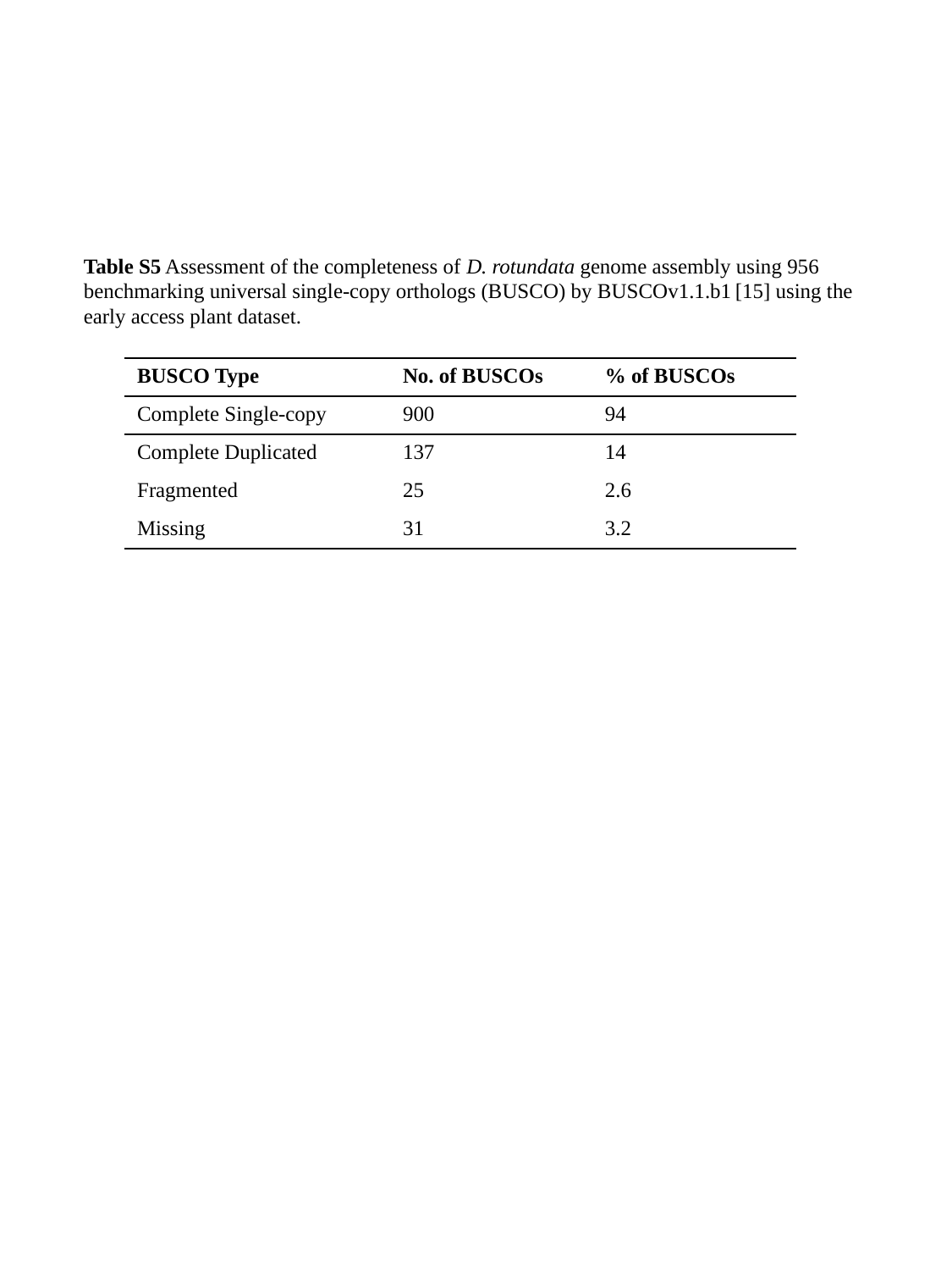

Table S5 Assessment of the completeness of D. rotundata genome assembly using 956 benchmarking universal single-copy orthologs (BUSCO) by BUSCOv1.1.b1 [15] using the early access plant dataset.
| BUSCO Type | No. of BUSCOs | % of BUSCOs |
| --- | --- | --- |
| Complete Single-copy | 900 | 94 |
| Complete Duplicated | 137 | 14 |
| Fragmented | 25 | 2.6 |
| Missing | 31 | 3.2 |

## Slide 6
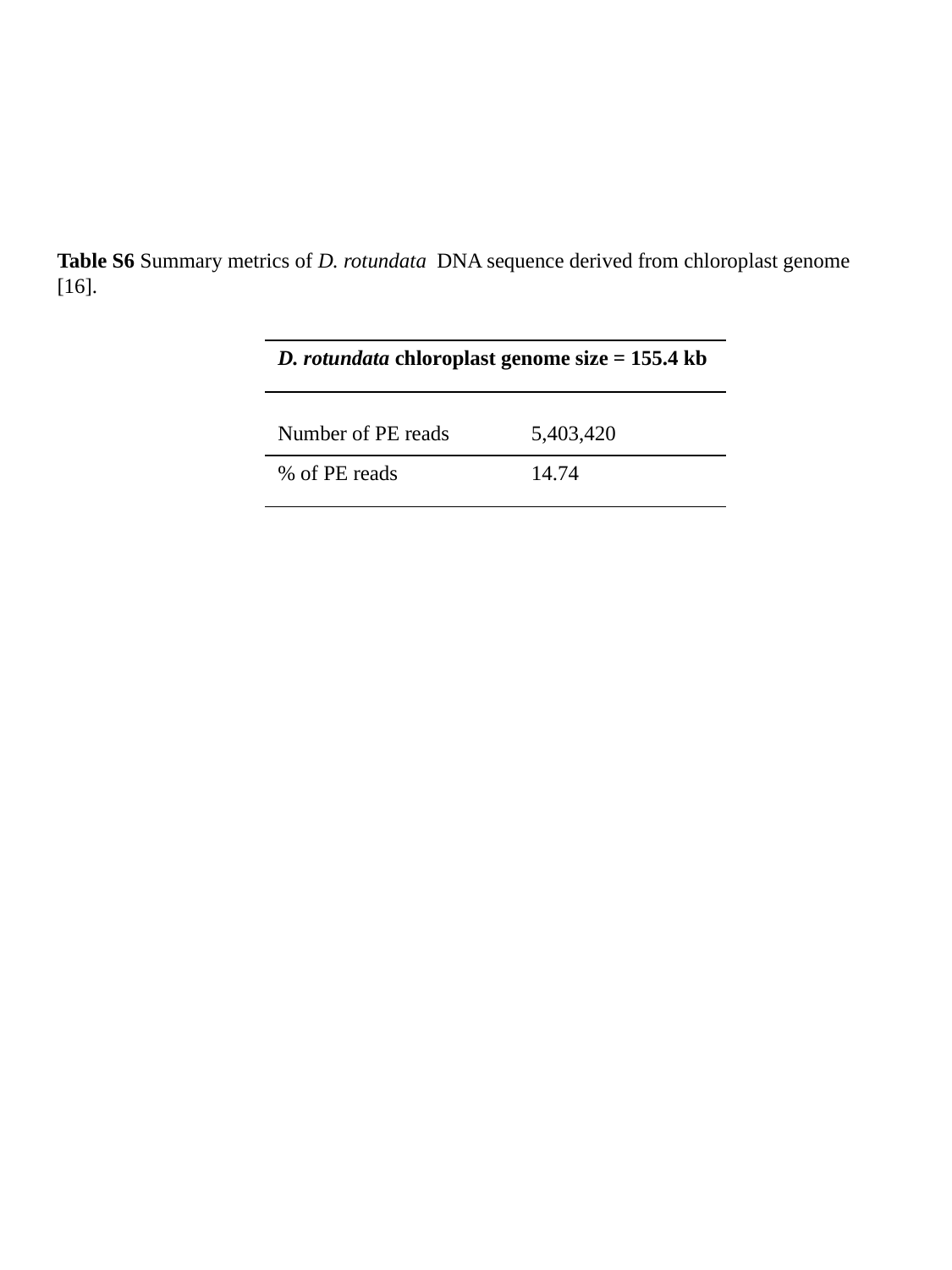

Table S6 Summary metrics of D. rotundata DNA sequence derived from chloroplast genome [16].
| D. rotundata chloroplast genome size = 155.4 kb | |
| --- | --- |
| Number of PE reads | 5,403,420 |
| % of PE reads | 14.74 |

## Slide 7
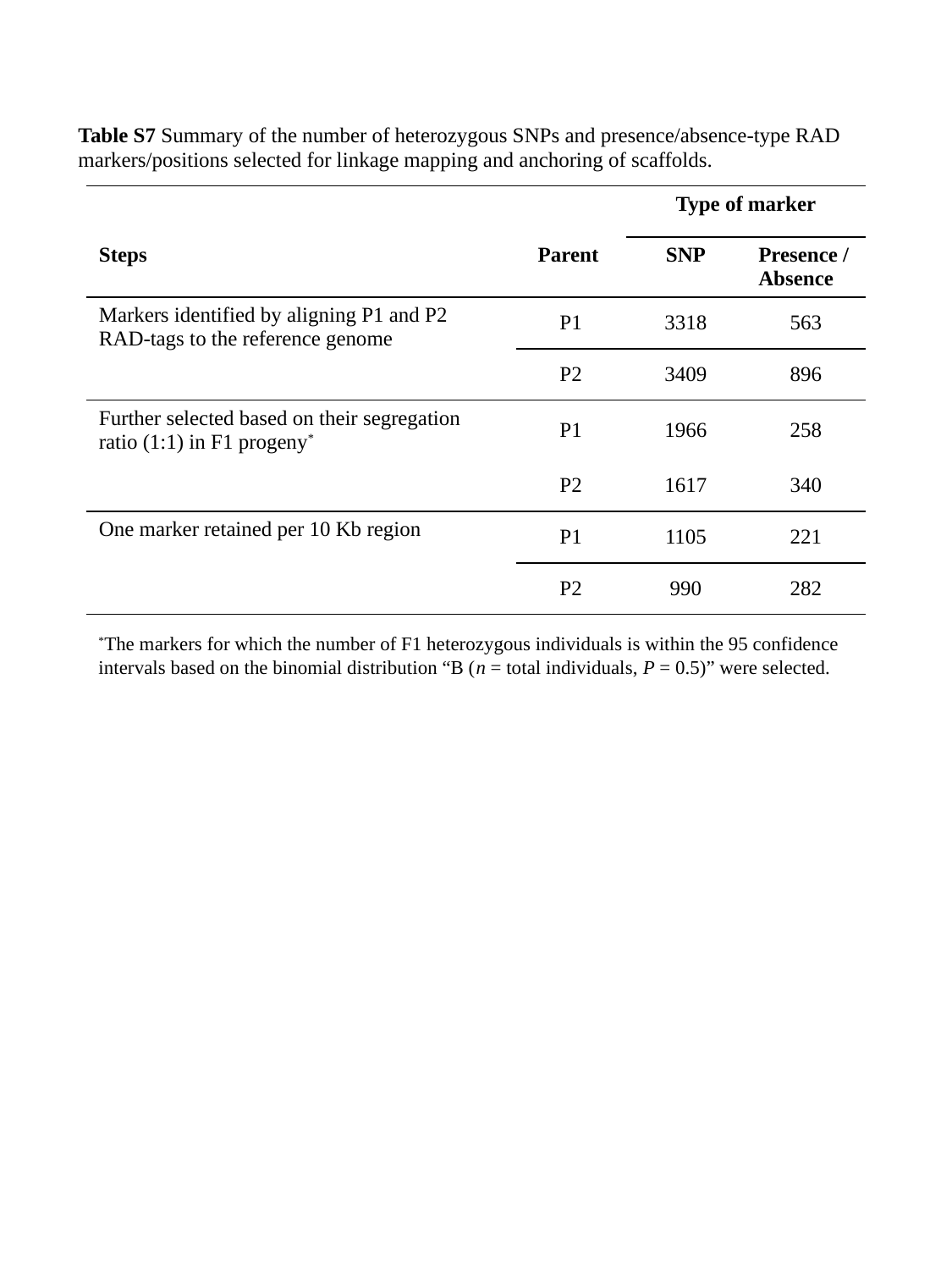

Table S7 Summary of the number of heterozygous SNPs and presence/absence-type RAD markers/positions selected for linkage mapping and anchoring of scaffolds.
| | | Type of marker | |
| --- | --- | --- | --- |
| Steps | Parent | SNP | Presence /Absence |
| Markers identified by aligning P1 and P2 RAD-tags to the reference genome | P1 | 3318 | 563 |
| | P2 | 3409 | 896 |
| Further selected based on their segregation ratio (1:1) in F1 progeny\* | P1 | 1966 | 258 |
| | P2 | 1617 | 340 |
| One marker retained per 10 Kb region | P1 | 1105 | 221 |
| | P2 | 990 | 282 |
*The markers for which the number of F1 heterozygous individuals is within the 95 confidence intervals based on the binomial distribution “B (n = total individuals, P = 0.5)” were selected.

## Slide 8
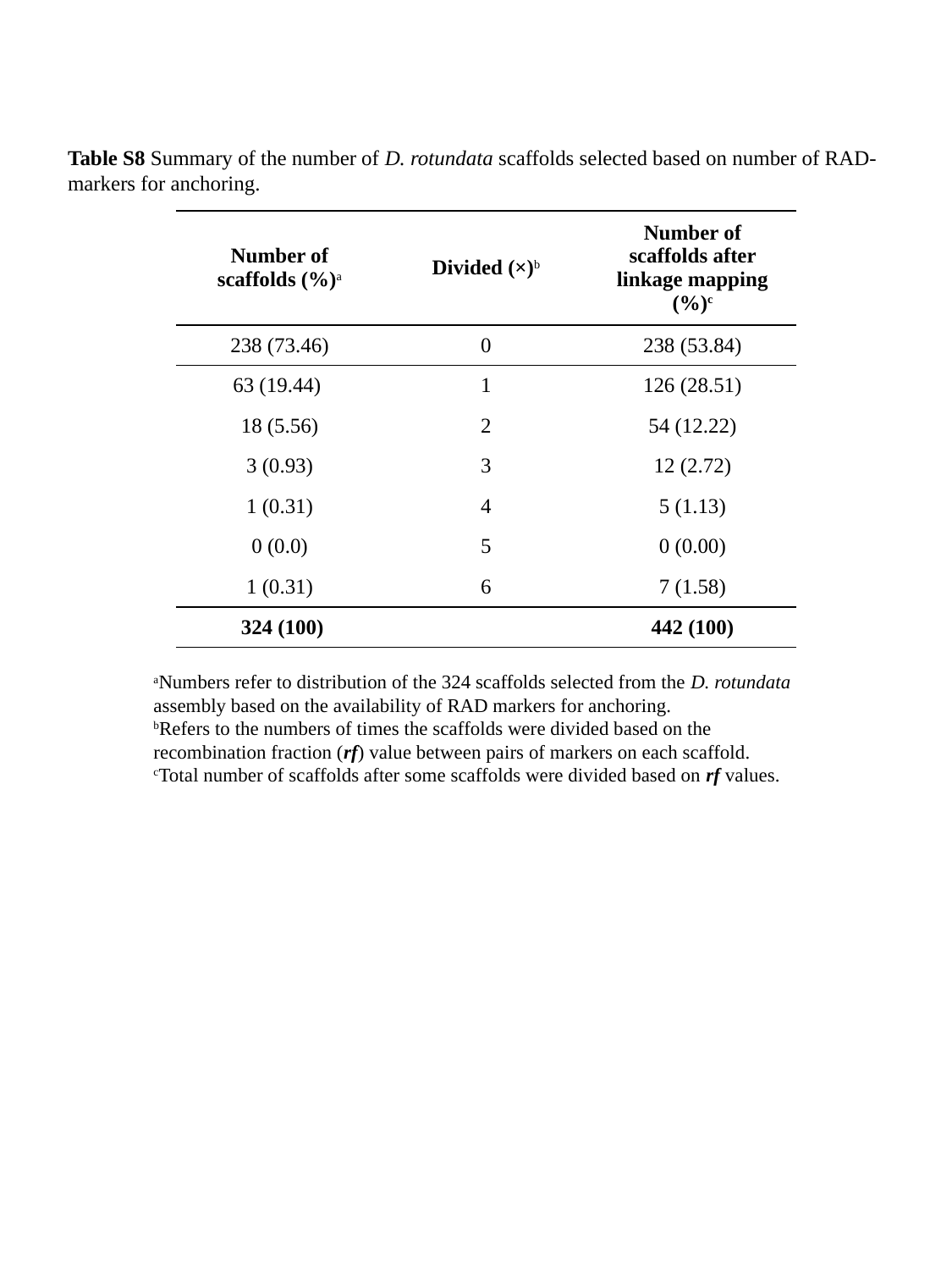

Table S8 Summary of the number of D. rotundata scaffolds selected based on number of RAD-markers for anchoring.
| Number of scaffolds (%)a | Divided (×)b | Number of scaffolds after linkage mapping (%)c |
| --- | --- | --- |
| 238 (73.46) | 0 | 238 (53.84) |
| 63 (19.44) | 1 | 126 (28.51) |
| 18 (5.56) | 2 | 54 (12.22) |
| 3 (0.93) | 3 | 12 (2.72) |
| 1 (0.31) | 4 | 5 (1.13) |
| 0 (0.0) | 5 | 0 (0.00) |
| 1 (0.31) | 6 | 7 (1.58) |
| 324 (100) | | 442 (100) |
aNumbers refer to distribution of the 324 scaffolds selected from the D. rotundata assembly based on the availability of RAD markers for anchoring.
bRefers to the numbers of times the scaffolds were divided based on the recombination fraction (rf) value between pairs of markers on each scaffold.
cTotal number of scaffolds after some scaffolds were divided based on rf values.

## Slide 9
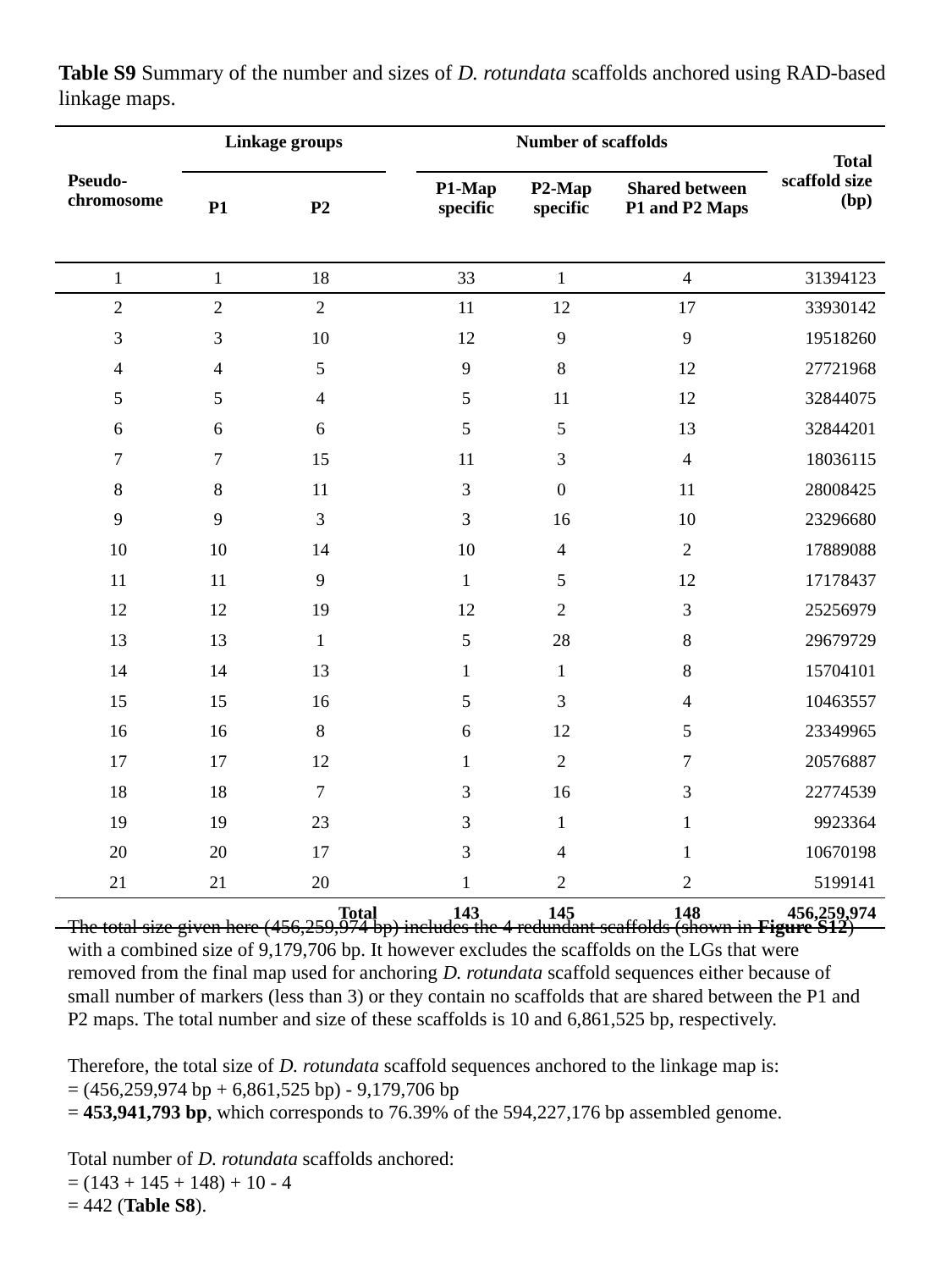

Table S9 Summary of the number and sizes of D. rotundata scaffolds anchored using RAD-based linkage maps.
| Pseudo-chromosome | Linkage groups | | | Number of scaffolds | | | Total scaffold size (bp) |
| --- | --- | --- | --- | --- | --- | --- | --- |
| | P1 | P2 | | P1-Map specific | P2-Map specific | Shared between P1 and P2 Maps | |
| 1 | 1 | 18 | | 33 | 1 | 4 | 31394123 |
| 2 | 2 | 2 | | 11 | 12 | 17 | 33930142 |
| 3 | 3 | 10 | | 12 | 9 | 9 | 19518260 |
| 4 | 4 | 5 | | 9 | 8 | 12 | 27721968 |
| 5 | 5 | 4 | | 5 | 11 | 12 | 32844075 |
| 6 | 6 | 6 | | 5 | 5 | 13 | 32844201 |
| 7 | 7 | 15 | | 11 | 3 | 4 | 18036115 |
| 8 | 8 | 11 | | 3 | 0 | 11 | 28008425 |
| 9 | 9 | 3 | | 3 | 16 | 10 | 23296680 |
| 10 | 10 | 14 | | 10 | 4 | 2 | 17889088 |
| 11 | 11 | 9 | | 1 | 5 | 12 | 17178437 |
| 12 | 12 | 19 | | 12 | 2 | 3 | 25256979 |
| 13 | 13 | 1 | | 5 | 28 | 8 | 29679729 |
| 14 | 14 | 13 | | 1 | 1 | 8 | 15704101 |
| 15 | 15 | 16 | | 5 | 3 | 4 | 10463557 |
| 16 | 16 | 8 | | 6 | 12 | 5 | 23349965 |
| 17 | 17 | 12 | | 1 | 2 | 7 | 20576887 |
| 18 | 18 | 7 | | 3 | 16 | 3 | 22774539 |
| 19 | 19 | 23 | | 3 | 1 | 1 | 9923364 |
| 20 | 20 | 17 | | 3 | 4 | 1 | 10670198 |
| 21 | 21 | 20 | | 1 | 2 | 2 | 5199141 |
| | Total | | | 143 | 145 | 148 | 456,259,974 |
The total size given here (456,259,974 bp) includes the 4 redundant scaffolds (shown in Figure S12) with a combined size of 9,179,706 bp. It however excludes the scaffolds on the LGs that were removed from the final map used for anchoring D. rotundata scaffold sequences either because of small number of markers (less than 3) or they contain no scaffolds that are shared between the P1 and P2 maps. The total number and size of these scaffolds is 10 and 6,861,525 bp, respectively.
Therefore, the total size of D. rotundata scaffold sequences anchored to the linkage map is:
= (456,259,974 bp + 6,861,525 bp) - 9,179,706 bp
= 453,941,793 bp, which corresponds to 76.39% of the 594,227,176 bp assembled genome.
Total number of D. rotundata scaffolds anchored:
= (143 + 145 + 148) + 10 - 4
= 442 (Table S8).

## Slide 10
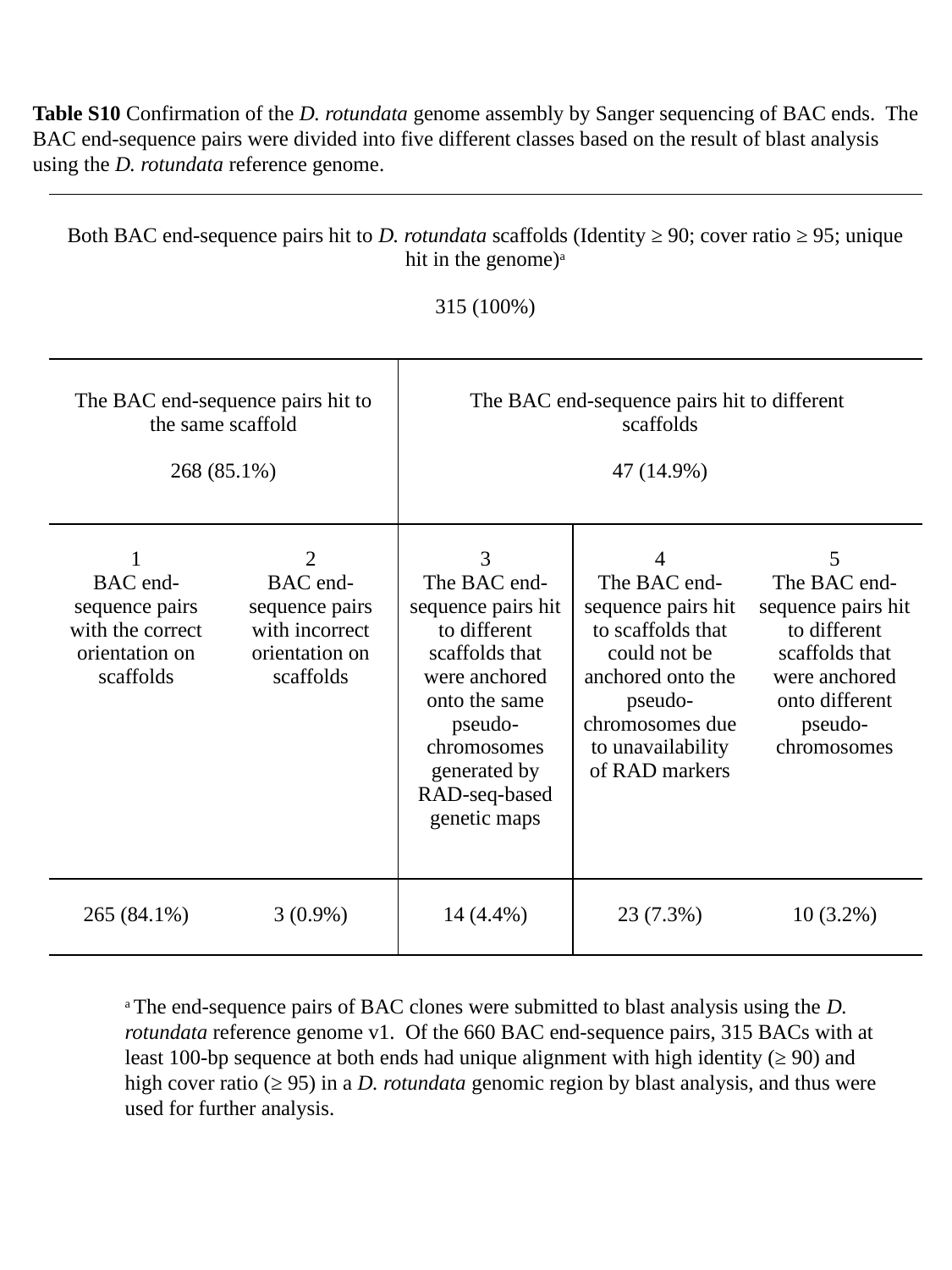

Table S10 Confirmation of the D. rotundata genome assembly by Sanger sequencing of BAC ends. The BAC end-sequence pairs were divided into five different classes based on the result of blast analysis using the D. rotundata reference genome.
| Both BAC end-sequence pairs hit to D. rotundata scaffolds (Identity ≥ 90; cover ratio ≥ 95; unique hit in the genome)a   315 (100%) | | | | |
| --- | --- | --- | --- | --- |
| The BAC end-sequence pairs hit to the same scaffold   268 (85.1%) | | The BAC end-sequence pairs hit to different scaffolds   47 (14.9%) | | |
| 1 BAC end-sequence pairs with the correct orientation on scaffolds | 2 BAC end-sequence pairs with incorrect orientation on scaffolds | 3 The BAC end-sequence pairs hit to different scaffolds that were anchored onto the same pseudo-chromosomes generated by RAD-seq-based genetic maps | 4 The BAC end-sequence pairs hit to scaffolds that could not be anchored onto the pseudo-chromosomes due to unavailability of RAD markers | 5 The BAC end-sequence pairs hit to different scaffolds that were anchored onto different pseudo-chromosomes |
| 265 (84.1%) | 3 (0.9%) | 14 (4.4%) | 23 (7.3%) | 10 (3.2%) |
a The end-sequence pairs of BAC clones were submitted to blast analysis using the D. rotundata reference genome v1. Of the 660 BAC end-sequence pairs, 315 BACs with at least 100-bp sequence at both ends had unique alignment with high identity (≥ 90) and high cover ratio (≥ 95) in a D. rotundata genomic region by blast analysis, and thus were used for further analysis.

## Slide 11
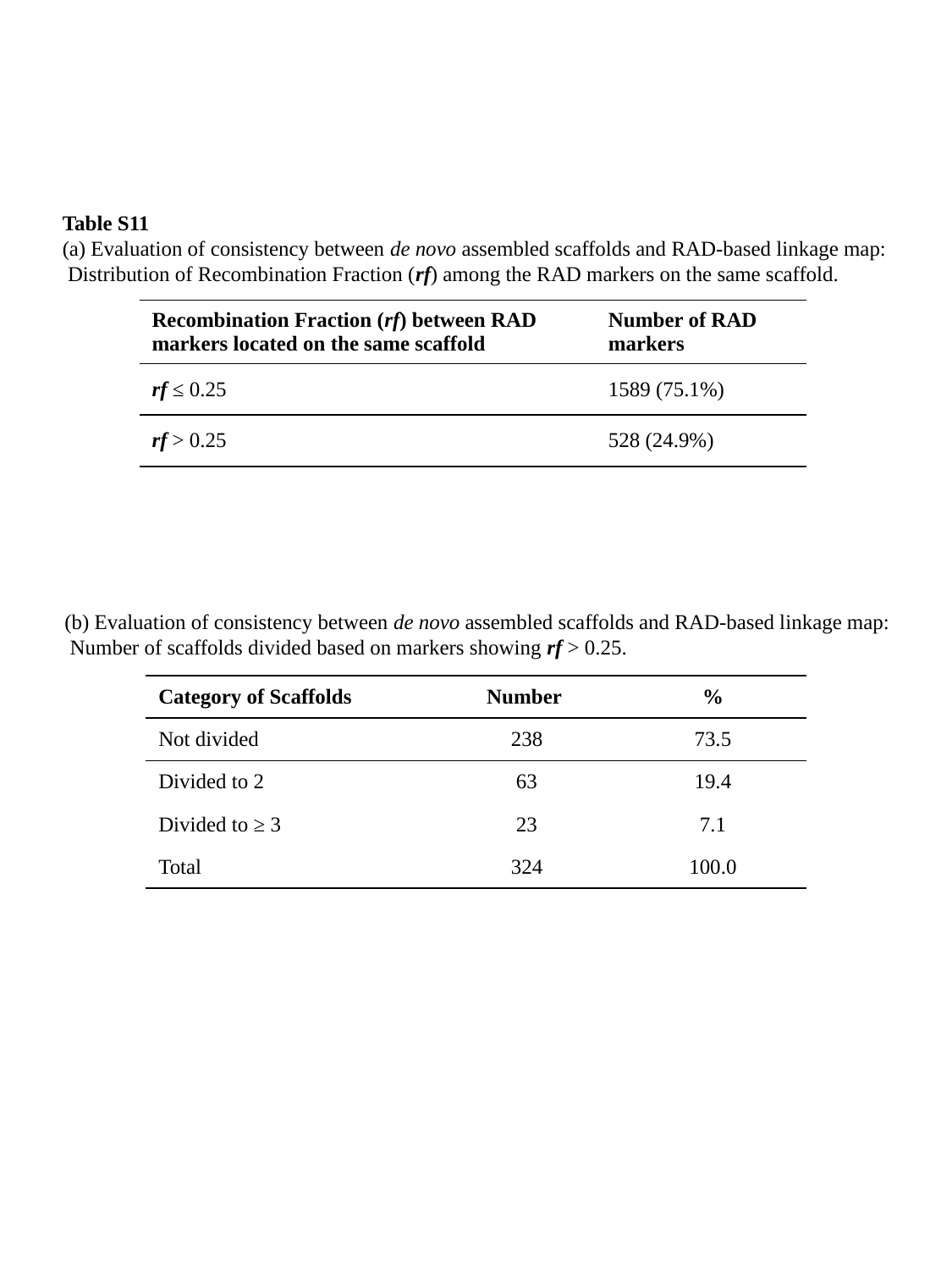

Table S11
(a) Evaluation of consistency between de novo assembled scaffolds and RAD-based linkage map: Distribution of Recombination Fraction (rf) among the RAD markers on the same scaffold.
| Recombination Fraction (rf) between RAD markers located on the same scaffold | Number of RAD markers |
| --- | --- |
| rf ≤ 0.25 | 1589 (75.1%) |
| rf > 0.25 | 528 (24.9%) |
(b) Evaluation of consistency between de novo assembled scaffolds and RAD-based linkage map: Number of scaffolds divided based on markers showing rf > 0.25.
| Category of Scaffolds | Number | % |
| --- | --- | --- |
| Not divided | 238 | 73.5 |
| Divided to 2 | 63 | 19.4 |
| Divided to ≥ 3 | 23 | 7.1 |
| Total | 324 | 100.0 |

## Slide 12
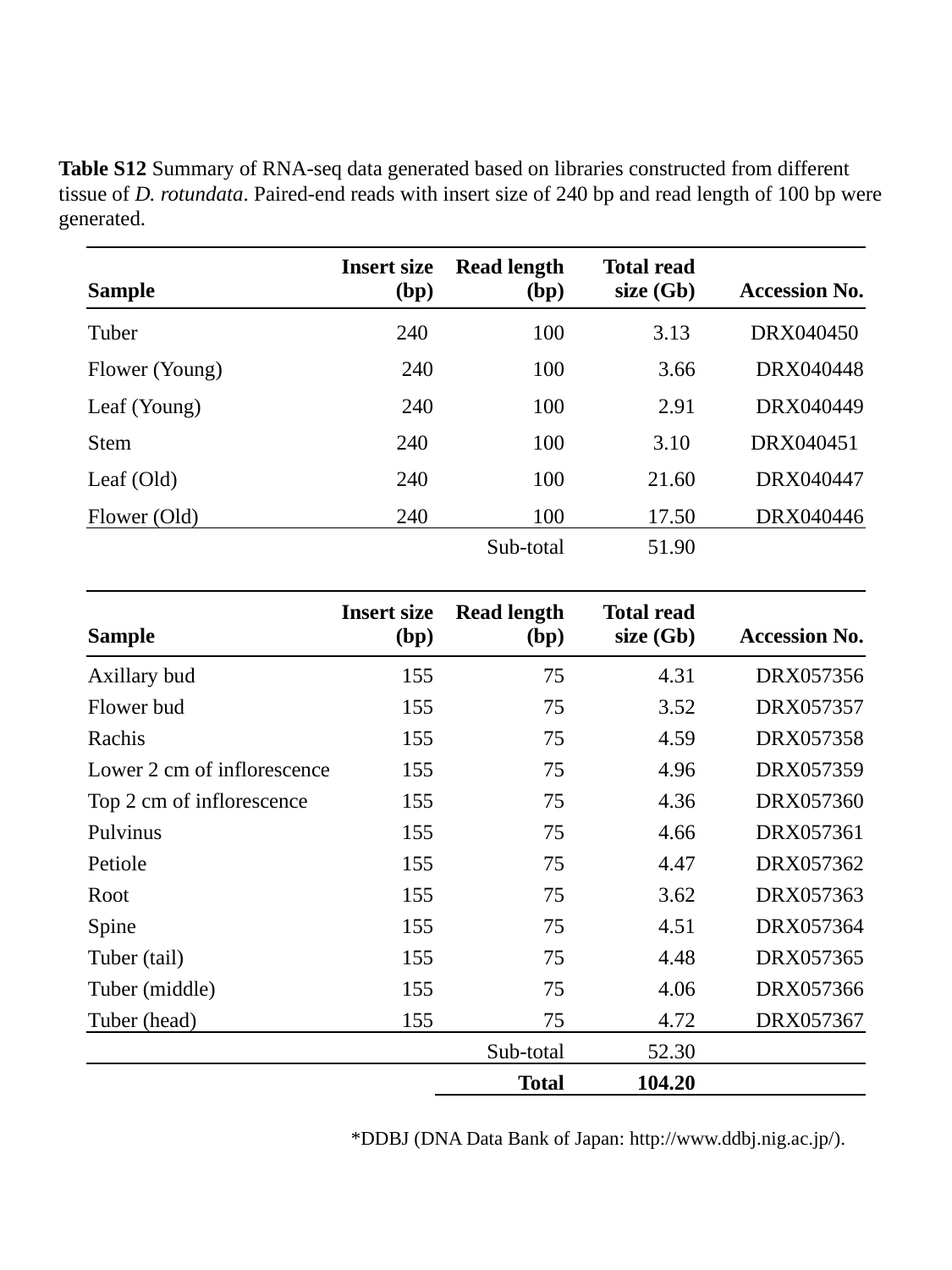

Table S12 Summary of RNA-seq data generated based on libraries constructed from different tissue of D. rotundata. Paired-end reads with insert size of 240 bp and read length of 100 bp were generated.
| Sample | Insert size (bp) | Read length (bp) | Total read size (Gb) | Accession No. |
| --- | --- | --- | --- | --- |
| Tuber | 240 | 100 | 3.13 | DRX040450 |
| Flower (Young) | 240 | 100 | 3.66 | DRX040448 |
| Leaf (Young) | 240 | 100 | 2.91 | DRX040449 |
| Stem | 240 | 100 | 3.10 | DRX040451 |
| Leaf (Old) | 240 | 100 | 21.60 | DRX040447 |
| Flower (Old) | 240 | 100 | 17.50 | DRX040446 |
| | | Sub-total | 51.90 | |
| | | | | |
| Sample | Insert size (bp) | Read length (bp) | Total read size (Gb) | Accession No. |
| Axillary bud | 155 | 75 | 4.31 | DRX057356 |
| Flower bud | 155 | 75 | 3.52 | DRX057357 |
| Rachis | 155 | 75 | 4.59 | DRX057358 |
| Lower 2 cm of inflorescence | 155 | 75 | 4.96 | DRX057359 |
| Top 2 cm of inflorescence | 155 | 75 | 4.36 | DRX057360 |
| Pulvinus | 155 | 75 | 4.66 | DRX057361 |
| Petiole | 155 | 75 | 4.47 | DRX057362 |
| Root | 155 | 75 | 3.62 | DRX057363 |
| Spine | 155 | 75 | 4.51 | DRX057364 |
| Tuber (tail) | 155 | 75 | 4.48 | DRX057365 |
| Tuber (middle) | 155 | 75 | 4.06 | DRX057366 |
| Tuber (head) | 155 | 75 | 4.72 | DRX057367 |
| | | Sub-total | 52.30 | |
| | | Total | 104.20 | |
*DDBJ (DNA Data Bank of Japan: http://www.ddbj.nig.ac.jp/).

## Slide 13
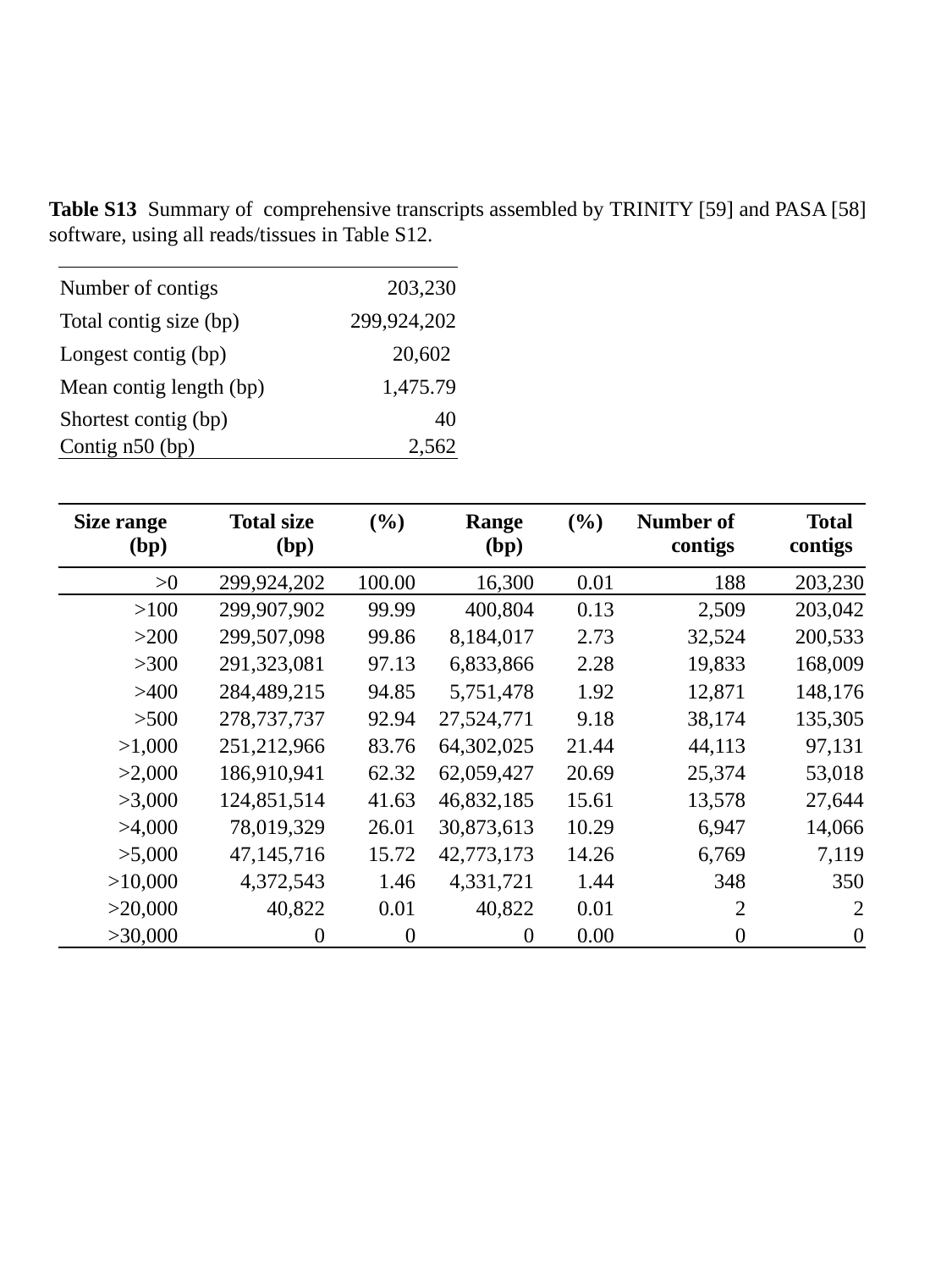

Table S13 Summary of comprehensive transcripts assembled by trinity [59] and PASA [58] software, using all reads/tissues in Table S12.
| Number of contigs | 203,230 |
| --- | --- |
| Total contig size (bp) | 299,924,202 |
| Longest contig (bp) | 20,602 |
| Mean contig length (bp) | 1,475.79 |
| Shortest contig (bp) | 40 |
| Contig n50 (bp) | 2,562 |
| Size range (bp) | Total size (bp) | (%) | Range (bp) | (%) | Number of contigs | Total contigs |
| --- | --- | --- | --- | --- | --- | --- |
| >0 | 299,924,202 | 100.00 | 16,300 | 0.01 | 188 | 203,230 |
| >100 | 299,907,902 | 99.99 | 400,804 | 0.13 | 2,509 | 203,042 |
| >200 | 299,507,098 | 99.86 | 8,184,017 | 2.73 | 32,524 | 200,533 |
| >300 | 291,323,081 | 97.13 | 6,833,866 | 2.28 | 19,833 | 168,009 |
| >400 | 284,489,215 | 94.85 | 5,751,478 | 1.92 | 12,871 | 148,176 |
| >500 | 278,737,737 | 92.94 | 27,524,771 | 9.18 | 38,174 | 135,305 |
| >1,000 | 251,212,966 | 83.76 | 64,302,025 | 21.44 | 44,113 | 97,131 |
| >2,000 | 186,910,941 | 62.32 | 62,059,427 | 20.69 | 25,374 | 53,018 |
| >3,000 | 124,851,514 | 41.63 | 46,832,185 | 15.61 | 13,578 | 27,644 |
| >4,000 | 78,019,329 | 26.01 | 30,873,613 | 10.29 | 6,947 | 14,066 |
| >5,000 | 47,145,716 | 15.72 | 42,773,173 | 14.26 | 6,769 | 7,119 |
| >10,000 | 4,372,543 | 1.46 | 4,331,721 | 1.44 | 348 | 350 |
| >20,000 | 40,822 | 0.01 | 40,822 | 0.01 | 2 | 2 |
| >30,000 | 0 | 0 | 0 | 0.00 | 0 | 0 |

## Slide 14
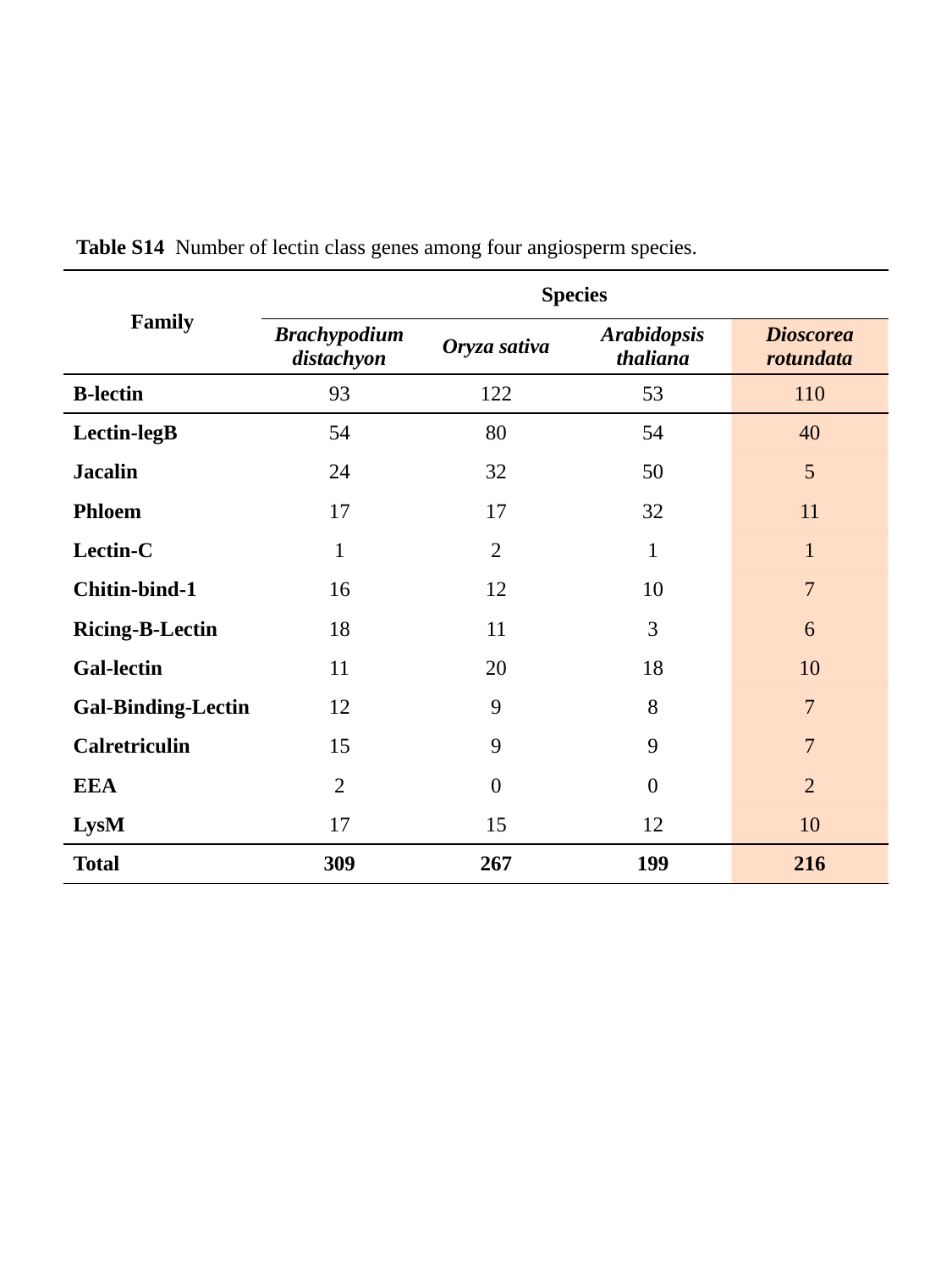

Table S14 Number of lectin class genes among four angiosperm species.
| Family | Species | | | |
| --- | --- | --- | --- | --- |
| | Brachypodium distachyon | Oryza sativa | Arabidopsis thaliana | Dioscorea rotundata |
| B-lectin | 93 | 122 | 53 | 110 |
| Lectin-legB | 54 | 80 | 54 | 40 |
| Jacalin | 24 | 32 | 50 | 5 |
| Phloem | 17 | 17 | 32 | 11 |
| Lectin-C | 1 | 2 | 1 | 1 |
| Chitin-bind-1 | 16 | 12 | 10 | 7 |
| Ricing-B-Lectin | 18 | 11 | 3 | 6 |
| Gal-lectin | 11 | 20 | 18 | 10 |
| Gal-Binding-Lectin | 12 | 9 | 8 | 7 |
| Calretriculin | 15 | 9 | 9 | 7 |
| EEA | 2 | 0 | 0 | 2 |
| LysM | 17 | 15 | 12 | 10 |
| Total | 309 | 267 | 199 | 216 |

## Slide 15
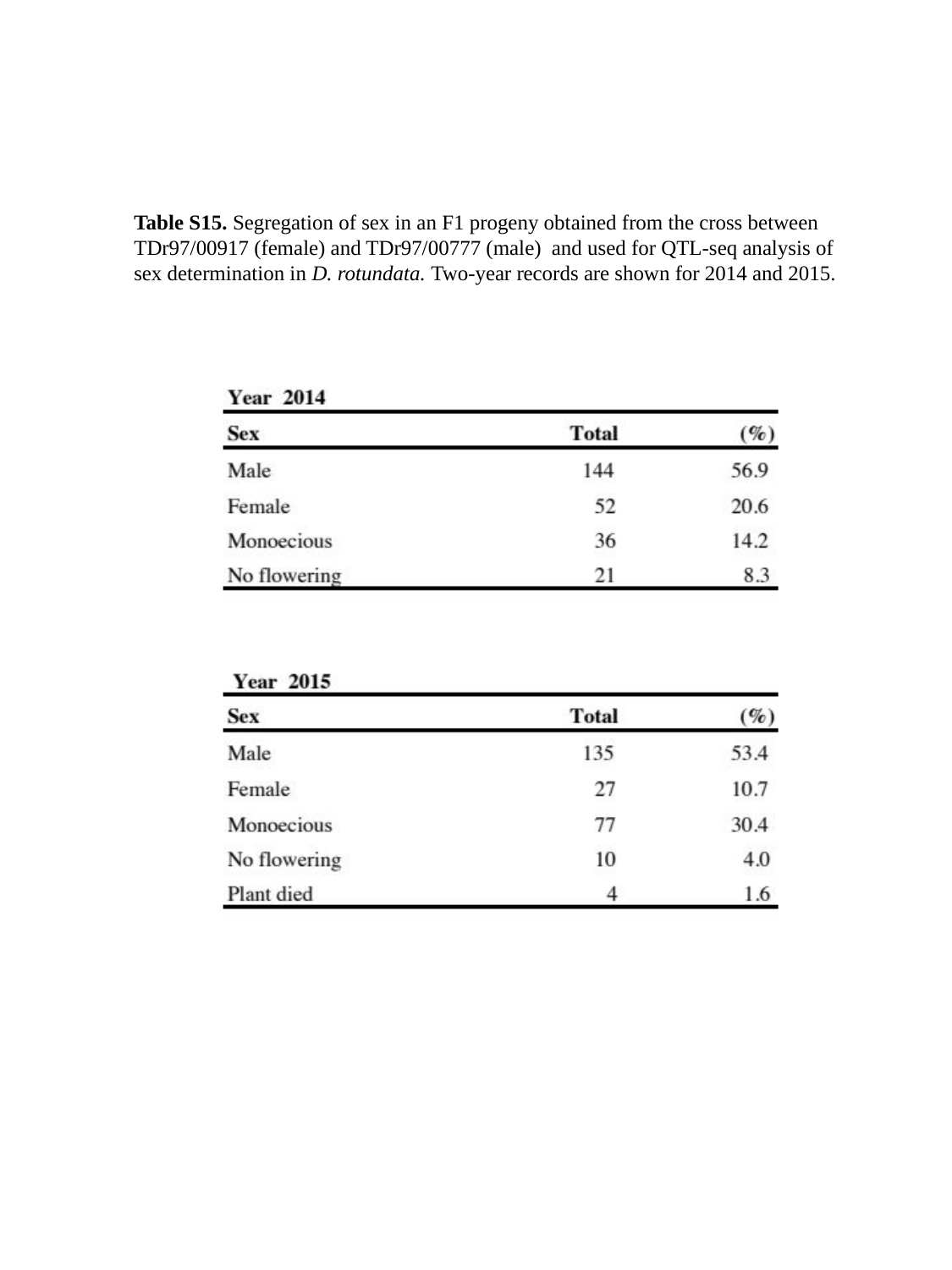

Table S15. Segregation of sex in an F1 progeny obtained from the cross between TDr97/00917 (female) and TDr97/00777 (male) and used for QTL-seq analysis of sex determination in D. rotundata. Two-year records are shown for 2014 and 2015.

## Slide 16
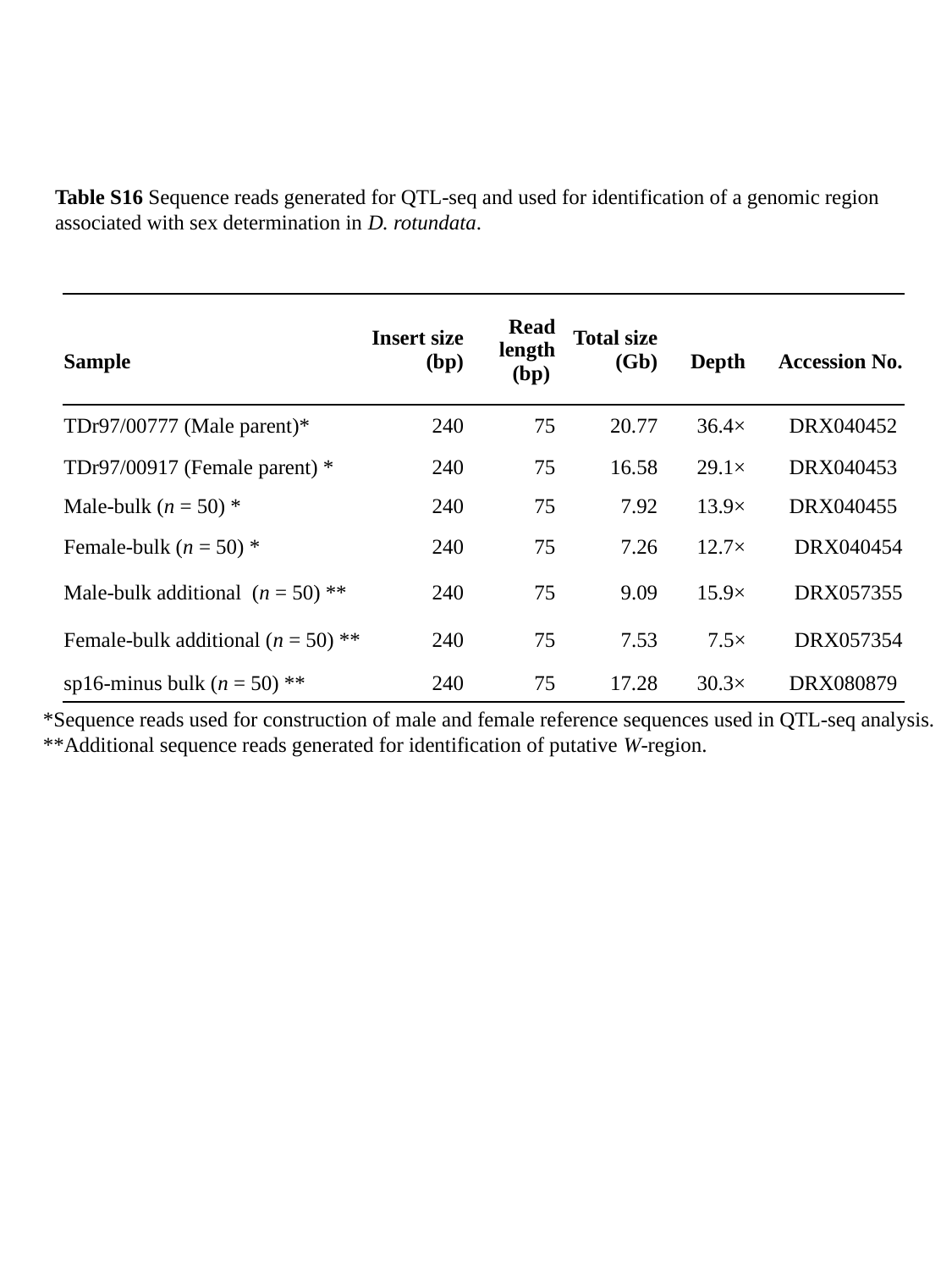

Table S16 Sequence reads generated for QTL-seq and used for identification of a genomic region associated with sex determination in D. rotundata.
| Sample | Insert size (bp) | Read length (bp) | Total size (Gb) | Depth | Accession No. |
| --- | --- | --- | --- | --- | --- |
| TDr97/00777 (Male parent)\* | 240 | 75 | 20.77 | 36.4× | DRX040452 |
| TDr97/00917 (Female parent) \* | 240 | 75 | 16.58 | 29.1× | DRX040453 |
| Male-bulk (n = 50) \* | 240 | 75 | 7.92 | 13.9× | DRX040455 |
| Female-bulk (n = 50) \* | 240 | 75 | 7.26 | 12.7× | DRX040454 |
| Male-bulk additional (n = 50) \*\* | 240 | 75 | 9.09 | 15.9× | DRX057355 |
| Female-bulk additional (n = 50) \*\* | 240 | 75 | 7.53 | 7.5× | DRX057354 |
| sp16-minus bulk (n = 50) \*\* | 240 | 75 | 17.28 | 30.3× | DRX080879 |
*Sequence reads used for construction of male and female reference sequences used in QTL-seq analysis.
**Additional sequence reads generated for identification of putative W-region.

## Slide 17
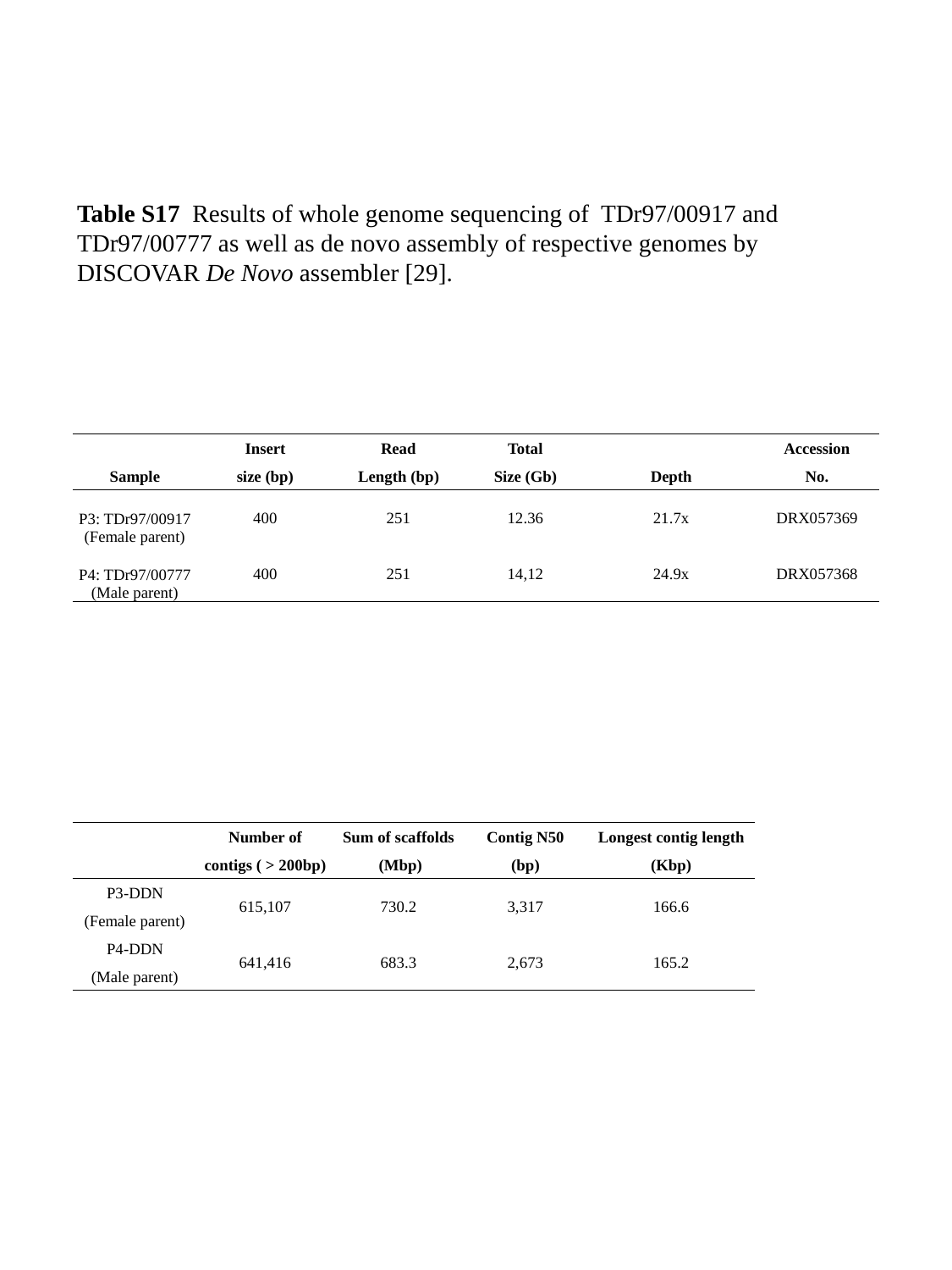

Table S17 Results of whole genome sequencing of TDr97/00917 and TDr97/00777 as well as de novo assembly of respective genomes by DISCOVAR De Novo assembler [29].
| | | | | | | | |
| --- | --- | --- | --- | --- | --- | --- | --- |
| | | Insert | Read | Total | | Accession | |
| | Sample | size (bp) | Length (bp) | Size (Gb) | Depth | No. | |
| | P3: TDr97/00917 (Female parent) | 400 | 251 | 12.36 | 21.7x | DRX057369 | |
| | P4: TDr97/00777 (Male parent) | 400 | 251 | 14,12 | 24.9x | DRX057368 | |
| | | | | | | | |
| | | | | | | | |
| | | | | | | | |
| | | | | | | | |
| | | | | | | | |
| | | | | | | | |
| | | | | | | | |
| | | | | | | | |
| | | | | | | | |
| | | | | | | | |
| | | Number of | Sum of scaffolds | Contig N50 | Longest contig length | | |
| | | contigs ( > 200bp) | (Mbp) | (bp) | (Kbp) | | |
| | P3-DDN | 615,107 | 730.2 | 3,317 | 166.6 | | |
| | (Female parent) | | | | | | |
| | P4-DDN | 641,416 | 683.3 | 2,673 | 165.2 | | |
| | (Male parent) | | | | | | |
| | | | | | | | |

## Slide 18
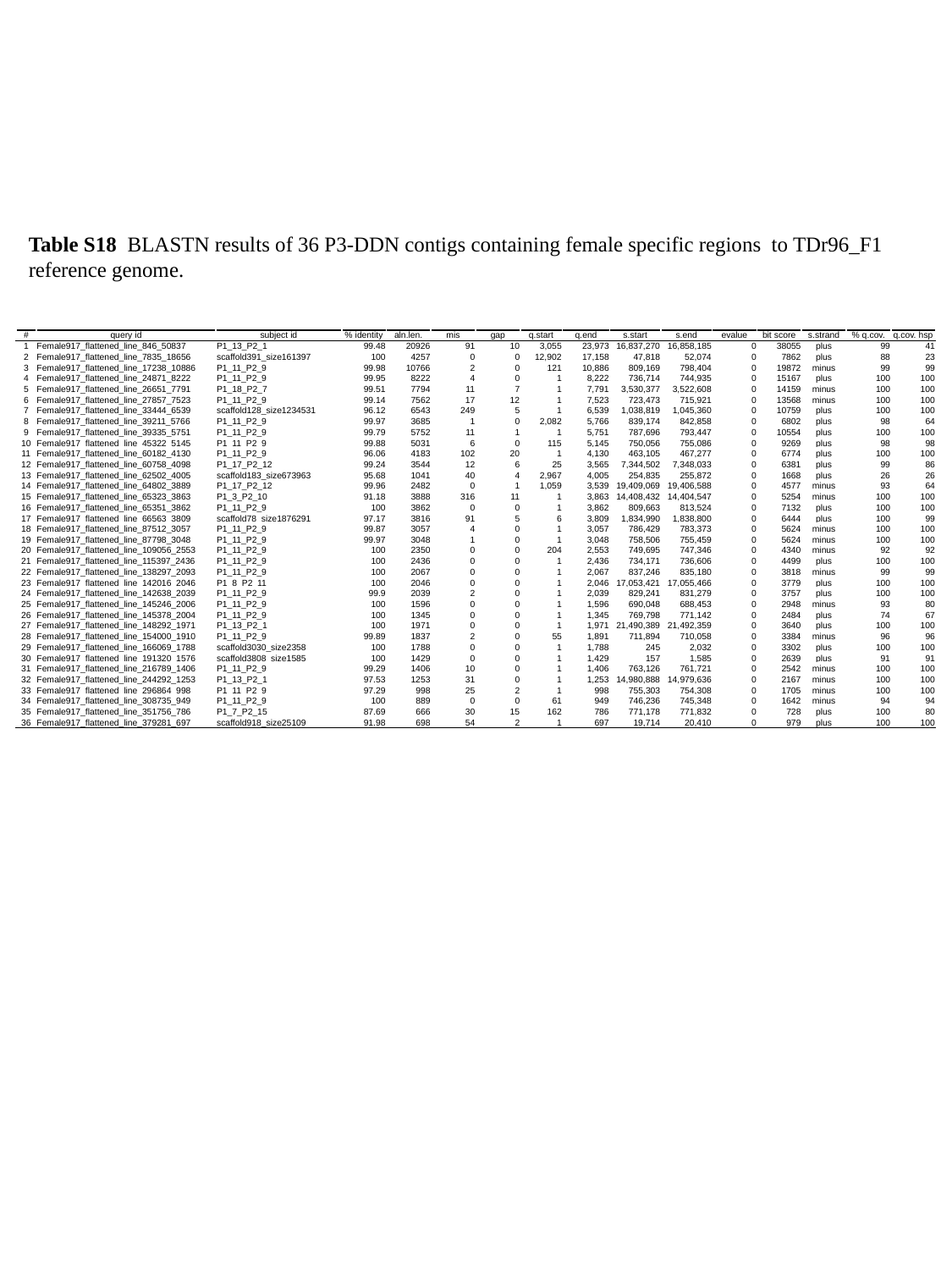

Table S18 BLASTN results of 36 P3-DDN contigs containing female specific regions to TDr96_F1 reference genome.
| # | query id | subject id | % identity | aln.len. | mis | gap | q.start | q.end | s.start | s.end | evalue | bit score | s.strand | % q.cov. | q.cov. hsp |
| --- | --- | --- | --- | --- | --- | --- | --- | --- | --- | --- | --- | --- | --- | --- | --- |
| 1 | Female917\_flattened\_line\_846\_50837 | P1\_13\_P2\_1 | 99.48 | 20926 | 91 | 10 | 3,055 | 23,973 | 16,837,270 | 16,858,185 | 0 | 38055 | plus | 99 | 41 |
| 2 | Female917\_flattened\_line\_7835\_18656 | scaffold391\_size161397 | 100 | 4257 | 0 | 0 | 12,902 | 17,158 | 47,818 | 52,074 | 0 | 7862 | plus | 88 | 23 |
| 3 | Female917\_flattened\_line\_17238\_10886 | P1\_11\_P2\_9 | 99.98 | 10766 | 2 | 0 | 121 | 10,886 | 809,169 | 798,404 | 0 | 19872 | minus | 99 | 99 |
| 4 | Female917\_flattened\_line\_24871\_8222 | P1\_11\_P2\_9 | 99.95 | 8222 | 4 | 0 | 1 | 8,222 | 736,714 | 744,935 | 0 | 15167 | plus | 100 | 100 |
| 5 | Female917\_flattened\_line\_26651\_7791 | P1\_18\_P2\_7 | 99.51 | 7794 | 11 | 7 | 1 | 7,791 | 3,530,377 | 3,522,608 | 0 | 14159 | minus | 100 | 100 |
| 6 | Female917\_flattened\_line\_27857\_7523 | P1\_11\_P2\_9 | 99.14 | 7562 | 17 | 12 | 1 | 7,523 | 723,473 | 715,921 | 0 | 13568 | minus | 100 | 100 |
| 7 | Female917\_flattened\_line\_33444\_6539 | scaffold128\_size1234531 | 96.12 | 6543 | 249 | 5 | 1 | 6,539 | 1,038,819 | 1,045,360 | 0 | 10759 | plus | 100 | 100 |
| 8 | Female917\_flattened\_line\_39211\_5766 | P1\_11\_P2\_9 | 99.97 | 3685 | 1 | 0 | 2,082 | 5,766 | 839,174 | 842,858 | 0 | 6802 | plus | 98 | 64 |
| 9 | Female917\_flattened\_line\_39335\_5751 | P1\_11\_P2\_9 | 99.79 | 5752 | 11 | 1 | 1 | 5,751 | 787,696 | 793,447 | 0 | 10554 | plus | 100 | 100 |
| 10 | Female917\_flattened\_line\_45322\_5145 | P1\_11\_P2\_9 | 99.88 | 5031 | 6 | 0 | 115 | 5,145 | 750,056 | 755,086 | 0 | 9269 | plus | 98 | 98 |
| 11 | Female917\_flattened\_line\_60182\_4130 | P1\_11\_P2\_9 | 96.06 | 4183 | 102 | 20 | 1 | 4,130 | 463,105 | 467,277 | 0 | 6774 | plus | 100 | 100 |
| 12 | Female917\_flattened\_line\_60758\_4098 | P1\_17\_P2\_12 | 99.24 | 3544 | 12 | 6 | 25 | 3,565 | 7,344,502 | 7,348,033 | 0 | 6381 | plus | 99 | 86 |
| 13 | Female917\_flattened\_line\_62502\_4005 | scaffold183\_size673963 | 95.68 | 1041 | 40 | 4 | 2,967 | 4,005 | 254,835 | 255,872 | 0 | 1668 | plus | 26 | 26 |
| 14 | Female917\_flattened\_line\_64802\_3889 | P1\_17\_P2\_12 | 99.96 | 2482 | 0 | 1 | 1,059 | 3,539 | 19,409,069 | 19,406,588 | 0 | 4577 | minus | 93 | 64 |
| 15 | Female917\_flattened\_line\_65323\_3863 | P1\_3\_P2\_10 | 91.18 | 3888 | 316 | 11 | 1 | 3,863 | 14,408,432 | 14,404,547 | 0 | 5254 | minus | 100 | 100 |
| 16 | Female917\_flattened\_line\_65351\_3862 | P1\_11\_P2\_9 | 100 | 3862 | 0 | 0 | 1 | 3,862 | 809,663 | 813,524 | 0 | 7132 | plus | 100 | 100 |
| 17 | Female917\_flattened\_line\_66563\_3809 | scaffold78\_size1876291 | 97.17 | 3816 | 91 | 5 | 6 | 3,809 | 1,834,990 | 1,838,800 | 0 | 6444 | plus | 100 | 99 |
| 18 | Female917\_flattened\_line\_87512\_3057 | P1\_11\_P2\_9 | 99.87 | 3057 | 4 | 0 | 1 | 3,057 | 786,429 | 783,373 | 0 | 5624 | minus | 100 | 100 |
| 19 | Female917\_flattened\_line\_87798\_3048 | P1\_11\_P2\_9 | 99.97 | 3048 | 1 | 0 | 1 | 3,048 | 758,506 | 755,459 | 0 | 5624 | minus | 100 | 100 |
| 20 | Female917\_flattened\_line\_109056\_2553 | P1\_11\_P2\_9 | 100 | 2350 | 0 | 0 | 204 | 2,553 | 749,695 | 747,346 | 0 | 4340 | minus | 92 | 92 |
| 21 | Female917\_flattened\_line\_115397\_2436 | P1\_11\_P2\_9 | 100 | 2436 | 0 | 0 | 1 | 2,436 | 734,171 | 736,606 | 0 | 4499 | plus | 100 | 100 |
| 22 | Female917\_flattened\_line\_138297\_2093 | P1\_11\_P2\_9 | 100 | 2067 | 0 | 0 | 1 | 2,067 | 837,246 | 835,180 | 0 | 3818 | minus | 99 | 99 |
| 23 | Female917\_flattened\_line\_142016\_2046 | P1\_8\_P2\_11 | 100 | 2046 | 0 | 0 | 1 | 2,046 | 17,053,421 | 17,055,466 | 0 | 3779 | plus | 100 | 100 |
| 24 | Female917\_flattened\_line\_142638\_2039 | P1\_11\_P2\_9 | 99.9 | 2039 | 2 | 0 | 1 | 2,039 | 829,241 | 831,279 | 0 | 3757 | plus | 100 | 100 |
| 25 | Female917\_flattened\_line\_145246\_2006 | P1\_11\_P2\_9 | 100 | 1596 | 0 | 0 | 1 | 1,596 | 690,048 | 688,453 | 0 | 2948 | minus | 93 | 80 |
| 26 | Female917\_flattened\_line\_145378\_2004 | P1\_11\_P2\_9 | 100 | 1345 | 0 | 0 | 1 | 1,345 | 769,798 | 771,142 | 0 | 2484 | plus | 74 | 67 |
| 27 | Female917\_flattened\_line\_148292\_1971 | P1\_13\_P2\_1 | 100 | 1971 | 0 | 0 | 1 | 1,971 | 21,490,389 | 21,492,359 | 0 | 3640 | plus | 100 | 100 |
| 28 | Female917\_flattened\_line\_154000\_1910 | P1\_11\_P2\_9 | 99.89 | 1837 | 2 | 0 | 55 | 1,891 | 711,894 | 710,058 | 0 | 3384 | minus | 96 | 96 |
| 29 | Female917\_flattened\_line\_166069\_1788 | scaffold3030\_size2358 | 100 | 1788 | 0 | 0 | 1 | 1,788 | 245 | 2,032 | 0 | 3302 | plus | 100 | 100 |
| 30 | Female917\_flattened\_line\_191320\_1576 | scaffold3808\_size1585 | 100 | 1429 | 0 | 0 | 1 | 1,429 | 157 | 1,585 | 0 | 2639 | plus | 91 | 91 |
| 31 | Female917\_flattened\_line\_216789\_1406 | P1\_11\_P2\_9 | 99.29 | 1406 | 10 | 0 | 1 | 1,406 | 763,126 | 761,721 | 0 | 2542 | minus | 100 | 100 |
| 32 | Female917\_flattened\_line\_244292\_1253 | P1\_13\_P2\_1 | 97.53 | 1253 | 31 | 0 | 1 | 1,253 | 14,980,888 | 14,979,636 | 0 | 2167 | minus | 100 | 100 |
| 33 | Female917\_flattened\_line\_296864\_998 | P1\_11\_P2\_9 | 97.29 | 998 | 25 | 2 | 1 | 998 | 755,303 | 754,308 | 0 | 1705 | minus | 100 | 100 |
| 34 | Female917\_flattened\_line\_308735\_949 | P1\_11\_P2\_9 | 100 | 889 | 0 | 0 | 61 | 949 | 746,236 | 745,348 | 0 | 1642 | minus | 94 | 94 |
| 35 | Female917\_flattened\_line\_351756\_786 | P1\_7\_P2\_15 | 87.69 | 666 | 30 | 15 | 162 | 786 | 771,178 | 771,832 | 0 | 728 | plus | 100 | 80 |
| 36 | Female917\_flattened\_line\_379281\_697 | scaffold918\_size25109 | 91.98 | 698 | 54 | 2 | 1 | 697 | 19,714 | 20,410 | 0 | 979 | plus | 100 | 100 |

## Slide 19
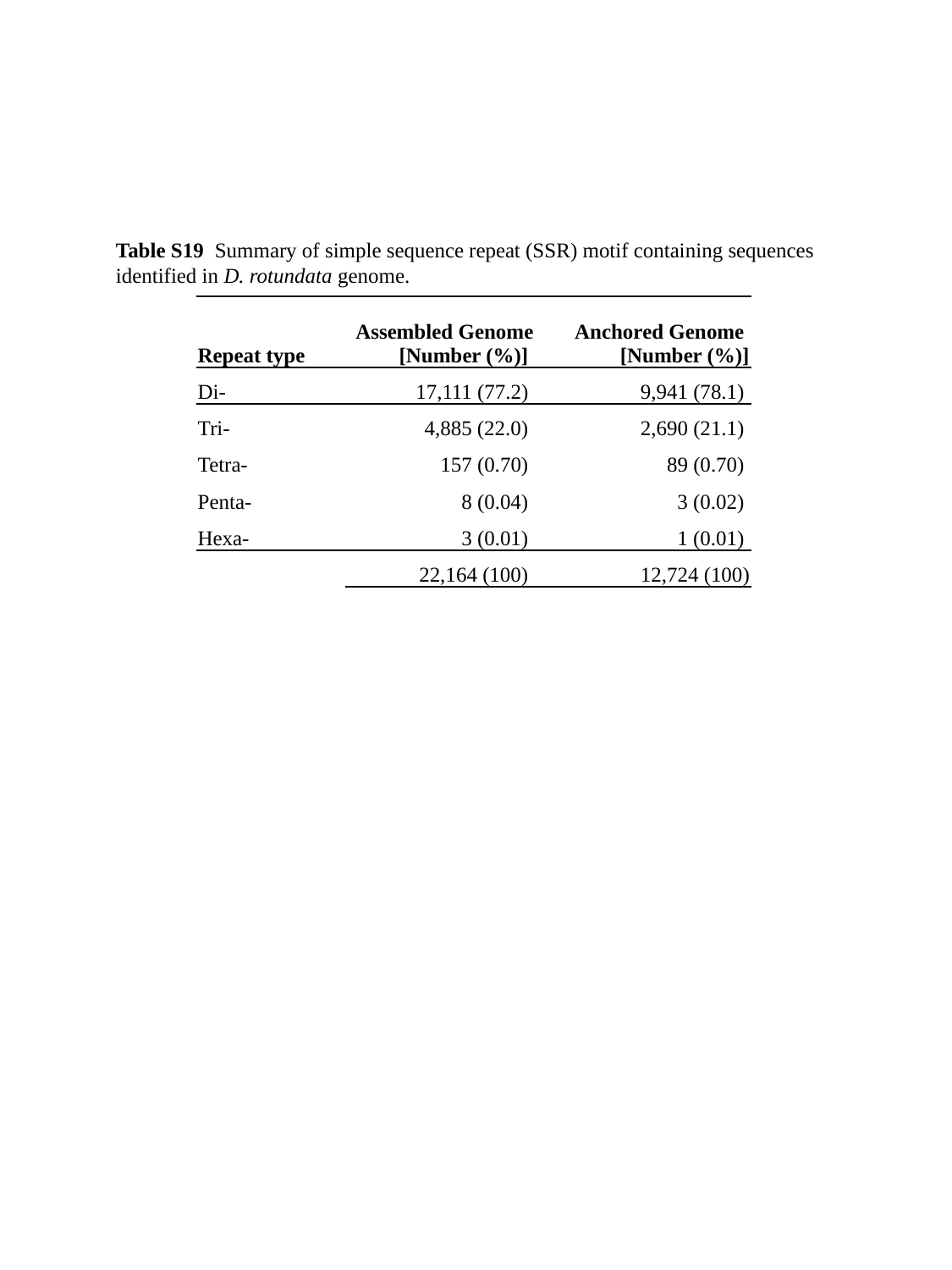

Table S19 Summary of simple sequence repeat (SSR) motif containing sequences identified in D. rotundata genome.
| Repeat type | Assembled Genome [Number (%)] | Anchored Genome [Number (%)] |
| --- | --- | --- |
| Di- | 17,111 (77.2) | 9,941 (78.1) |
| Tri- | 4,885 (22.0) | 2,690 (21.1) |
| Tetra- | 157 (0.70) | 89 (0.70) |
| Penta- | 8 (0.04) | 3 (0.02) |
| Hexa- | 3 (0.01) | 1 (0.01) |
| | 22,164 (100) | 12,724 (100) |

## Slide 20
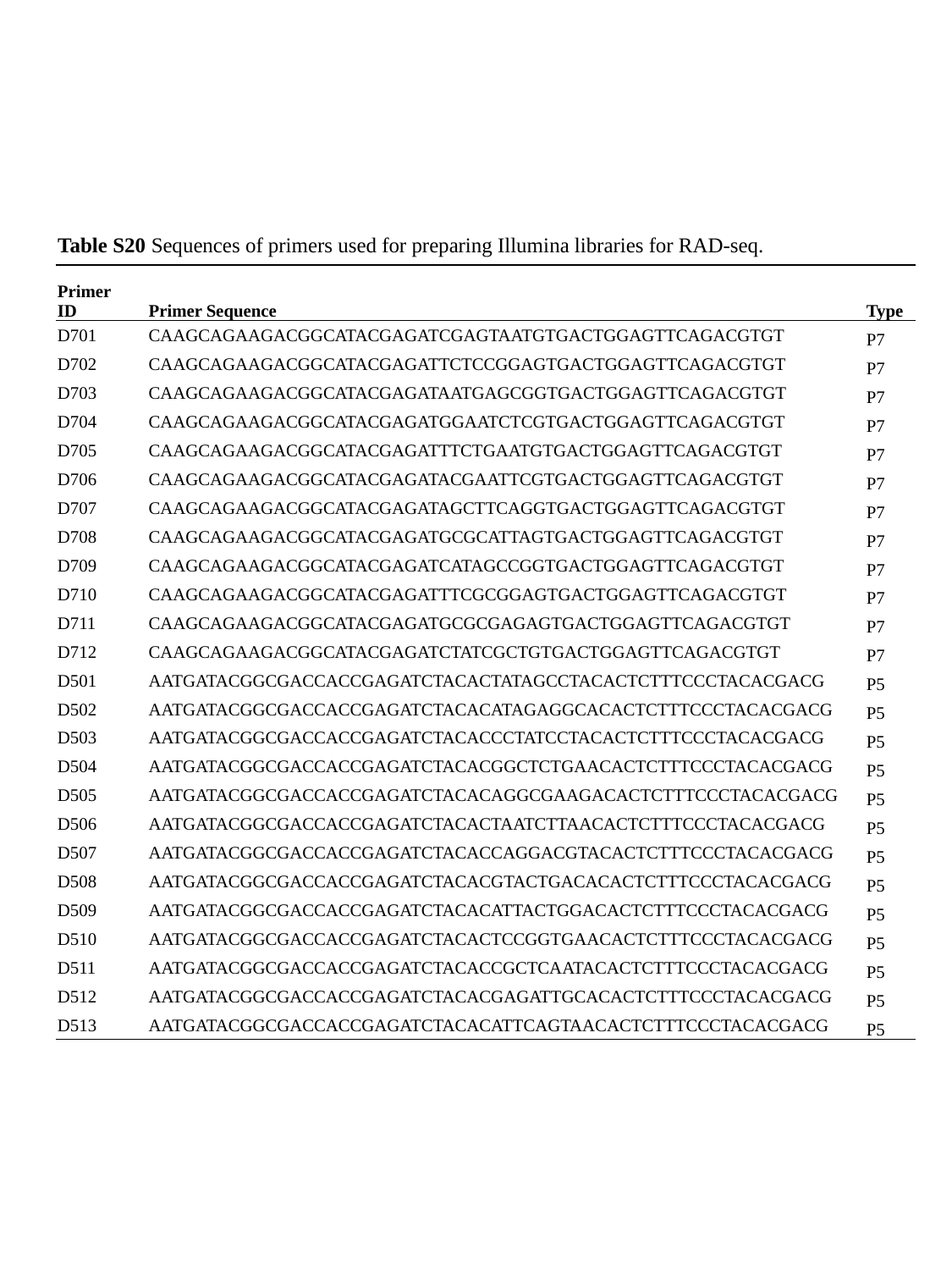

Table S20 Sequences of primers used for preparing Illumina libraries for RAD-seq.
| Primer ID | Primer Sequence | Type |
| --- | --- | --- |
| D701 | CAAGCAGAAGACGGCATACGAGATCGAGTAATGTGACTGGAGTTCAGACGTGT | P7 |
| D702 | CAAGCAGAAGACGGCATACGAGATTCTCCGGAGTGACTGGAGTTCAGACGTGT | P7 |
| D703 | CAAGCAGAAGACGGCATACGAGATAATGAGCGGTGACTGGAGTTCAGACGTGT | P7 |
| D704 | CAAGCAGAAGACGGCATACGAGATGGAATCTCGTGACTGGAGTTCAGACGTGT | P7 |
| D705 | CAAGCAGAAGACGGCATACGAGATTTCTGAATGTGACTGGAGTTCAGACGTGT | P7 |
| D706 | CAAGCAGAAGACGGCATACGAGATACGAATTCGTGACTGGAGTTCAGACGTGT | P7 |
| D707 | CAAGCAGAAGACGGCATACGAGATAGCTTCAGGTGACTGGAGTTCAGACGTGT | P7 |
| D708 | CAAGCAGAAGACGGCATACGAGATGCGCATTAGTGACTGGAGTTCAGACGTGT | P7 |
| D709 | CAAGCAGAAGACGGCATACGAGATCATAGCCGGTGACTGGAGTTCAGACGTGT | P7 |
| D710 | CAAGCAGAAGACGGCATACGAGATTTCGCGGAGTGACTGGAGTTCAGACGTGT | P7 |
| D711 | CAAGCAGAAGACGGCATACGAGATGCGCGAGAGTGACTGGAGTTCAGACGTGT | P7 |
| D712 | CAAGCAGAAGACGGCATACGAGATCTATCGCTGTGACTGGAGTTCAGACGTGT | P7 |
| D501 | AATGATACGGCGACCACCGAGATCTACACTATAGCCTACACTCTTTCCCTACACGACG | P5 |
| D502 | AATGATACGGCGACCACCGAGATCTACACATAGAGGCACACTCTTTCCCTACACGACG | P5 |
| D503 | AATGATACGGCGACCACCGAGATCTACACCCTATCCTACACTCTTTCCCTACACGACG | P5 |
| D504 | AATGATACGGCGACCACCGAGATCTACACGGCTCTGAACACTCTTTCCCTACACGACG | P5 |
| D505 | AATGATACGGCGACCACCGAGATCTACACAGGCGAAGACACTCTTTCCCTACACGACG | P5 |
| D506 | AATGATACGGCGACCACCGAGATCTACACTAATCTTAACACTCTTTCCCTACACGACG | P5 |
| D507 | AATGATACGGCGACCACCGAGATCTACACCAGGACGTACACTCTTTCCCTACACGACG | P5 |
| D508 | AATGATACGGCGACCACCGAGATCTACACGTACTGACACACTCTTTCCCTACACGACG | P5 |
| D509 | AATGATACGGCGACCACCGAGATCTACACATTACTGGACACTCTTTCCCTACACGACG | P5 |
| D510 | AATGATACGGCGACCACCGAGATCTACACTCCGGTGAACACTCTTTCCCTACACGACG | P5 |
| D511 | AATGATACGGCGACCACCGAGATCTACACCGCTCAATACACTCTTTCCCTACACGACG | P5 |
| D512 | AATGATACGGCGACCACCGAGATCTACACGAGATTGCACACTCTTTCCCTACACGACG | P5 |
| D513 | AATGATACGGCGACCACCGAGATCTACACATTCAGTAACACTCTTTCCCTACACGACG | P5 |
