# Supplementary material for: Genome sequencing of the staple food crop white Guinea yam enables the development of a molecular marker for sex determination
Source: BMC Biol. 2017 Sep 19;15:86. doi: 10.1186/s12915-017-0419-x (PMC5604175; doi:10.1186/s12915-017-0419-x)
Supplement: Supplementary file 2 — Supplementary figures including a summary of world yam production and photos of yam markets in West Africa (Figure S1), summary of BAC-end sequencing used for genome scaffolding (Figure S2), summary of k-mer analysis of Guinea yam genome (Figure S3), flowchart of Guinea yam genome assembly (Figure S4), summary of Guinea yam mitochondrial genome (Figure S5), flowchart of RAD-seq for linkage analysis (Figure S6), summary of RAD-seq analysis (Figure S7), summary of RAD-seq DNA markers used for linkage mapping and anchoring of scaffolds (Figure S8), procedure of linkage analysis and split of scaffolds depending on recombination fraction between RAD markers (Figure S9), RAD-seq-based linkage maps of D. rotundata generated by pseudo-testcross method (Figure S10), a matrix showing scaffolds shared between two linkage groups generated for two parents (Figure S11), schematic diagram for developing physical map of D. rotundata (Figure S12), frequency of distances of BAC-end sequences in the genome (Figure S13), scheme showing pipeline of genome annotation of D. rotundata (Figure S14), self-self syntenic dot plot of D. rotundata pseudo-chromosomes (Figure S15), SyMAP dot plot analysis of whole genome synteny between three monocot species (Figure S16), explanation of QTL-seq analysis to identify sex-linked genome regions in D. rotundata (Figure S17), QTL-seq results (Figure S18), sp1 DNA marker genotypes of F1 progeny and their association with sex (Figure S19), explanation of method for identification of putative W-region of D. rotundata genome (Figure S20), identification of female- and male-specific genomic regions (Figure S21), method of calculation of confidence interval of QTL-seq analysis (Figure S22). (PPTX 15700 kb) [file 12915_2017_419_MOESM2_ESM.pptx]

## Slide 1
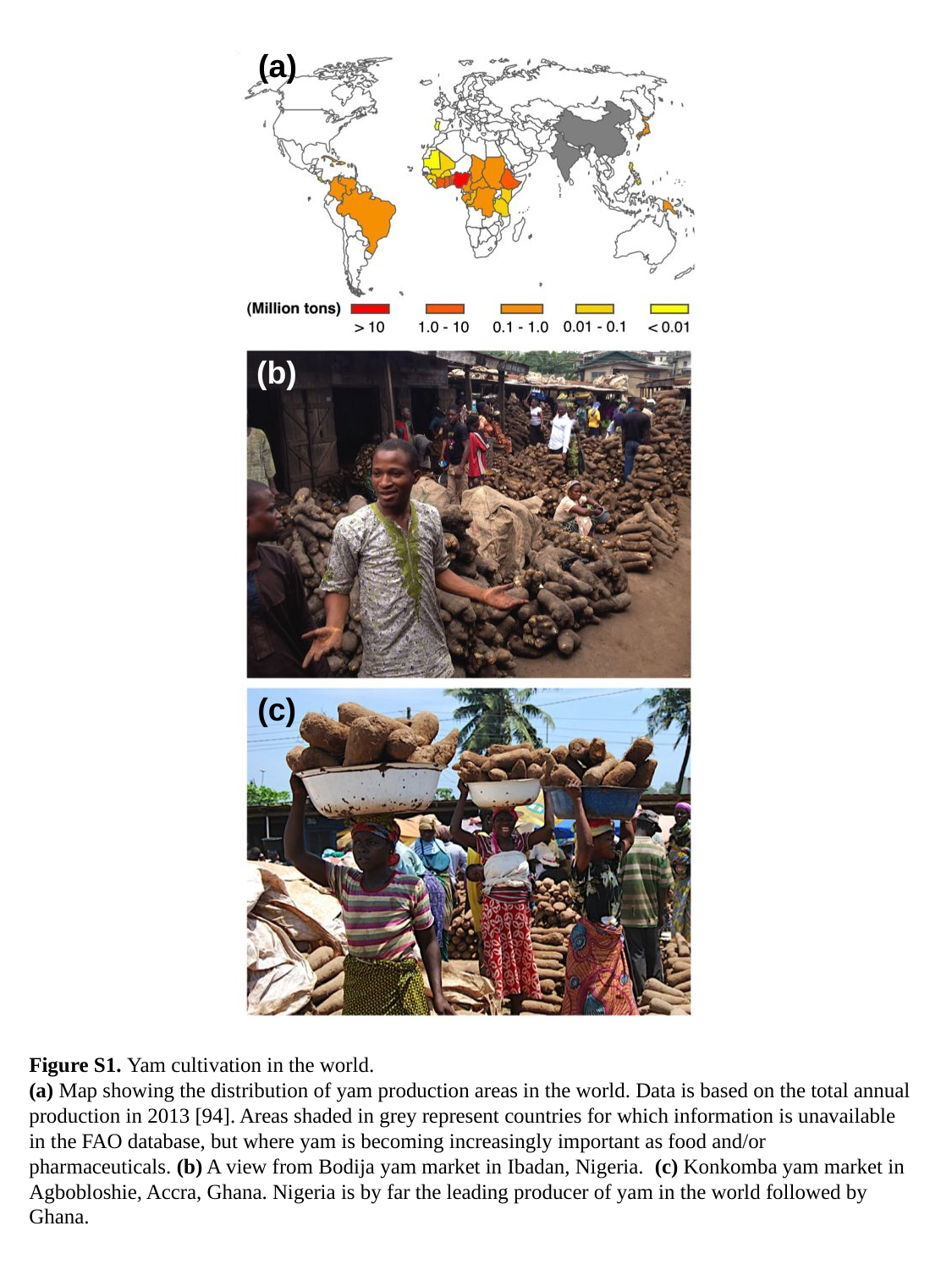

(a)
(b)
(c)
Figure S1. Yam cultivation in the world.
(a) Map showing the distribution of yam production areas in the world. Data is based on the total annual production in 2013 [94]. Areas shaded in grey represent countries for which information is unavailable in the FAO database, but where yam is becoming increasingly important as food and/or pharmaceuticals. (b) A view from Bodija yam market in Ibadan, Nigeria. (c) Konkomba yam market in Agbobloshie, Accra, Ghana. Nigeria is by far the leading producer of yam in the world followed by Ghana.

## Slide 2
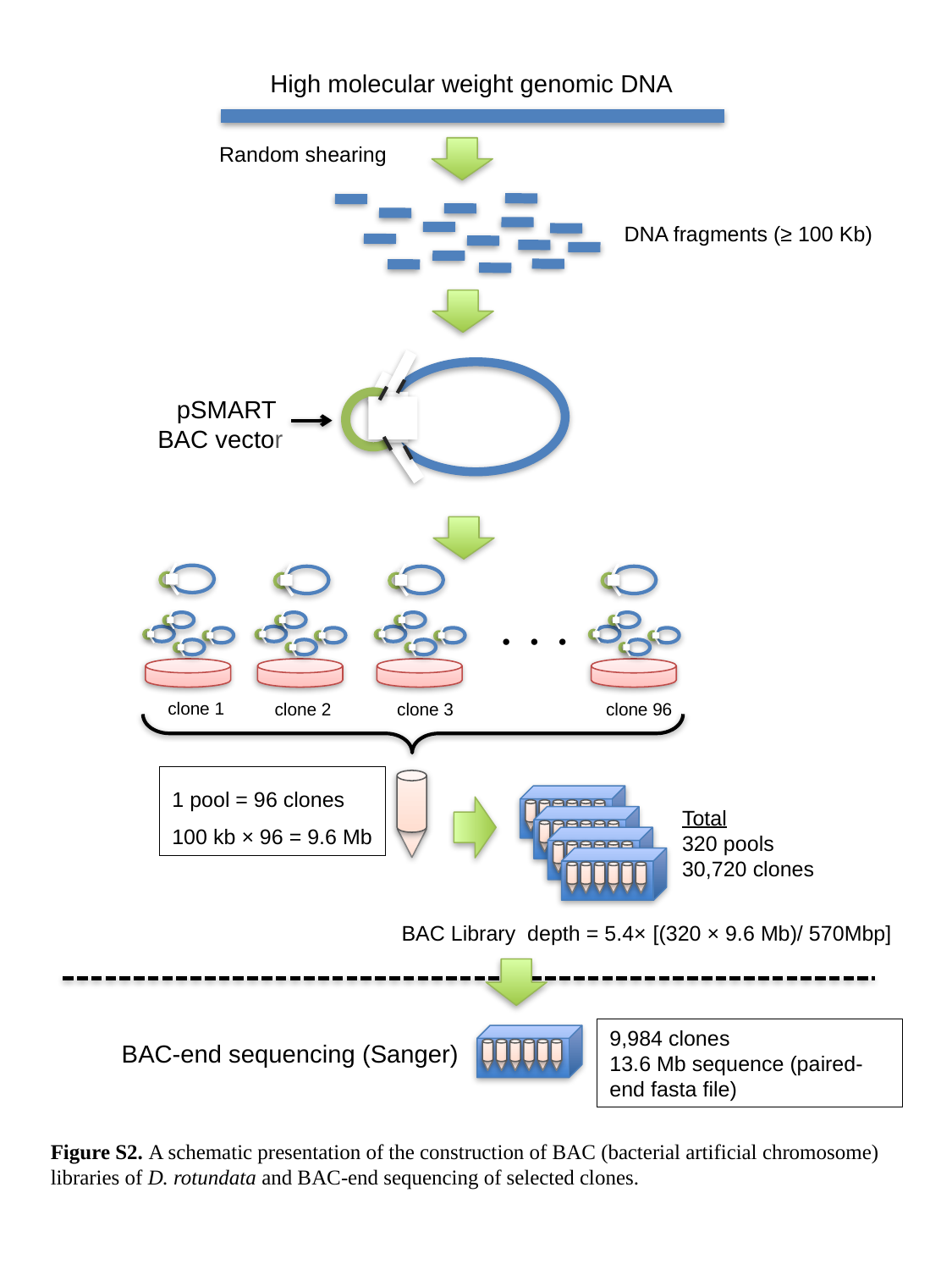

High molecular weight genomic DNA
Random shearing
DNA fragments (≥ 100 Kb)
pSMART
BAC vector
・・・
clone 1
clone 3
clone 96
clone 2
1 pool = 96 clones
100 kb × 96 = 9.6 Mb
Total
320 pools
30,720 clones
BAC Library depth = 5.4× [(320 × 9.6 Mb)/ 570Mbp]
9,984 clones
13.6 Mb sequence (paired-end fasta file)
BAC-end sequencing (Sanger)
Figure S2. A schematic presentation of the construction of BAC (bacterial artificial chromosome) libraries of D. rotundata and BAC-end sequencing of selected clones.

## Slide 3
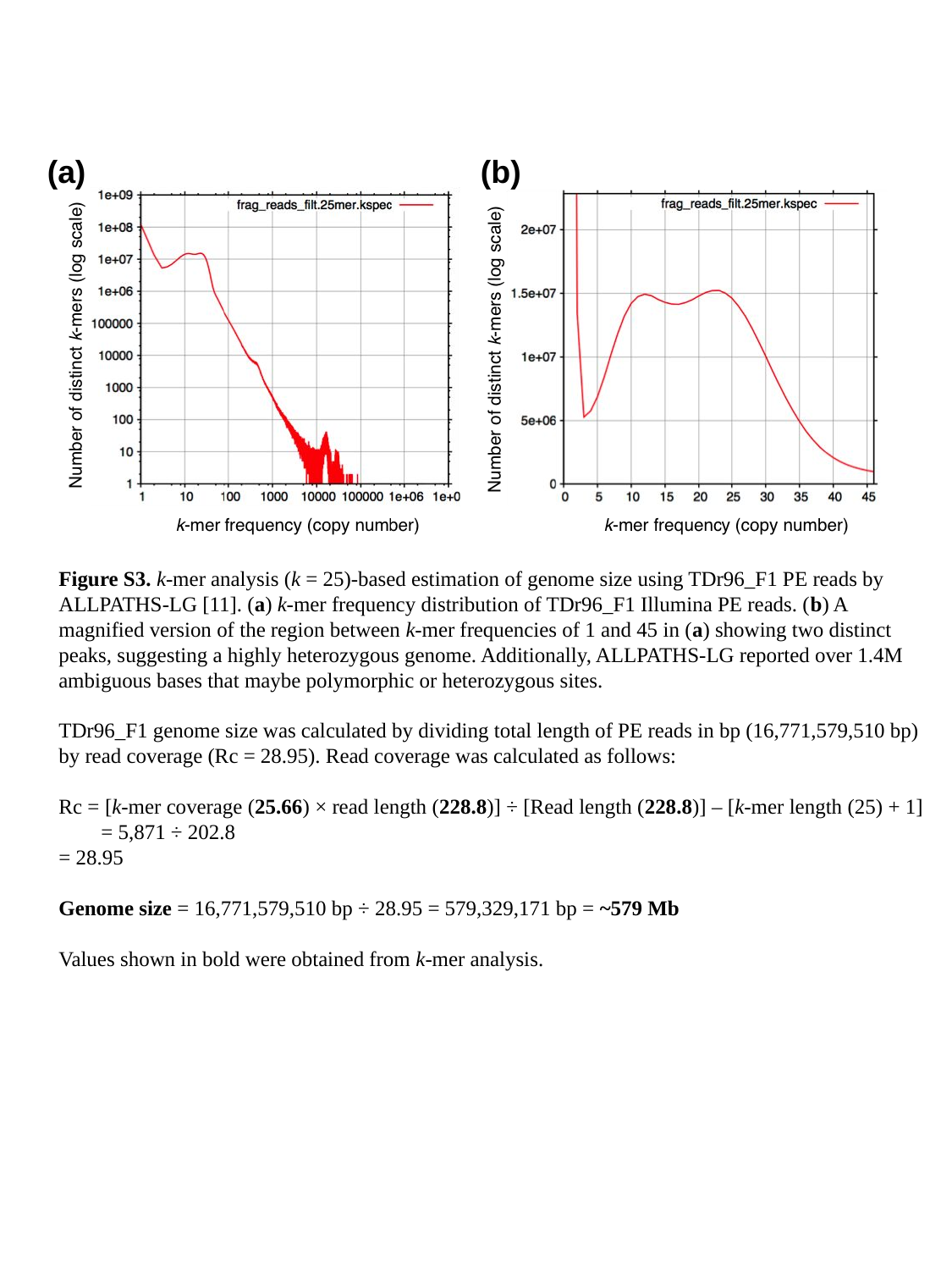

(a)
(b)
Figure S3. k-mer analysis (k = 25)-based estimation of genome size using TDr96_F1 PE reads by ALLPATHS-LG [11]. (a) k-mer frequency distribution of TDr96_F1 Illumina PE reads. (b) A magnified version of the region between k-mer frequencies of 1 and 45 in (a) showing two distinct peaks, suggesting a highly heterozygous genome. Additionally, ALLPATHS-LG reported over 1.4M ambiguous bases that maybe polymorphic or heterozygous sites.
TDr96_F1 genome size was calculated by dividing total length of PE reads in bp (16,771,579,510 bp) by read coverage (Rc = 28.95). Read coverage was calculated as follows:
Rc = [k-mer coverage (25.66) × read length (228.8)] ÷ [Read length (228.8)] – [k-mer length (25) + 1] = 5,871 ÷ 202.8
= 28.95
Genome size = 16,771,579,510 bp ÷ 28.95 = 579,329,171 bp = ~579 Mb
Values shown in bold were obtained from k-mer analysis.

## Slide 4
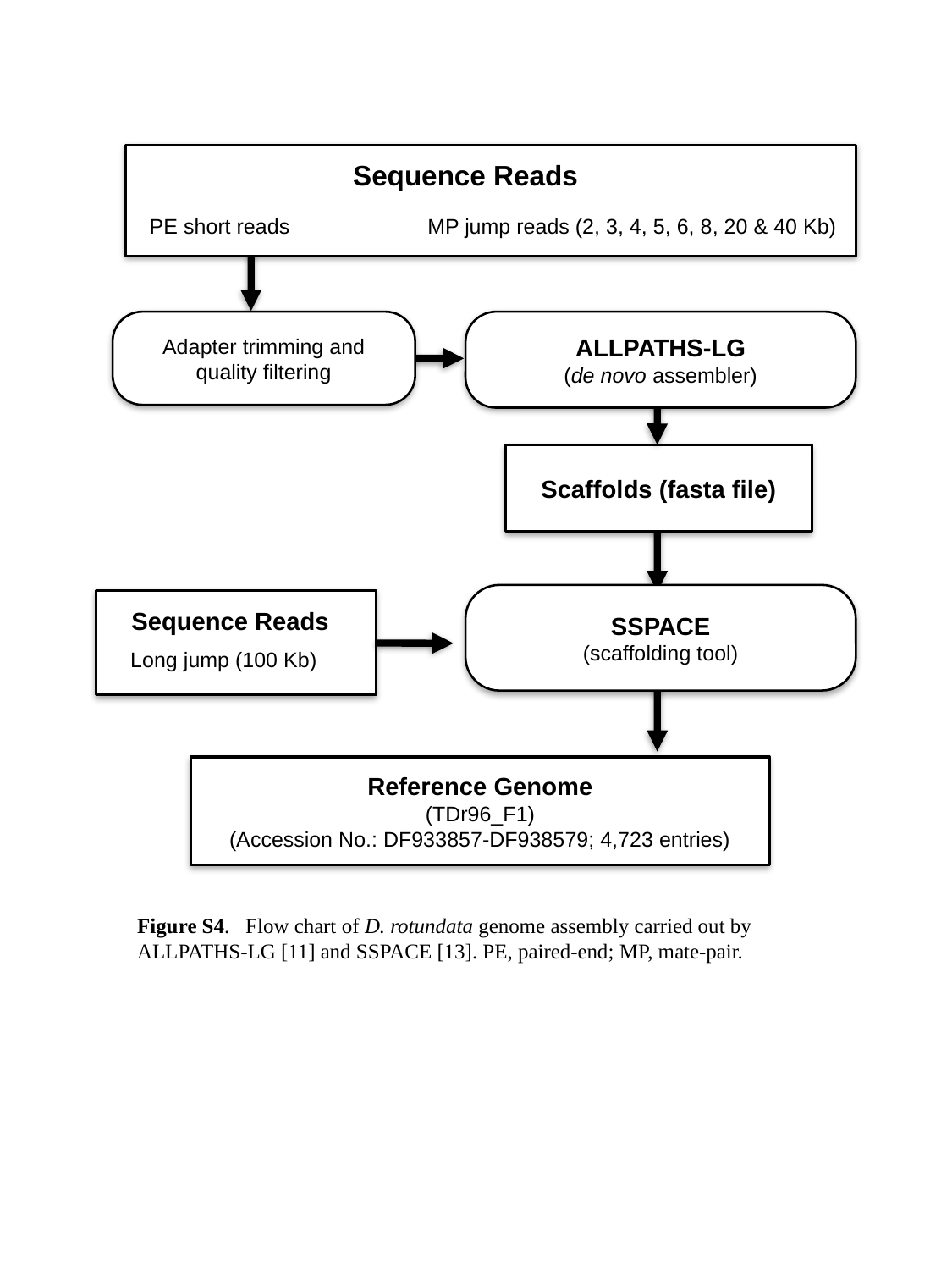

Sequence Reads
PE short reads
MP jump reads (2, 3, 4, 5, 6, 8, 20 & 40 Kb)
Adapter trimming and
quality filtering
ALLPATHS-LG
(de novo assembler)
Scaffolds (fasta file)
SSPACE
(scaffolding tool)
Sequence Reads
Long jump (100 Kb)
Reference Genome
(TDr96_F1)
(Accession No.: DF933857-DF938579; 4,723 entries)
Figure S4. Flow chart of D. rotundata genome assembly carried out by
ALLPATHS-LG [11] and SSPACE [13]. PE, paired-end; MP, mate-pair.

## Slide 5
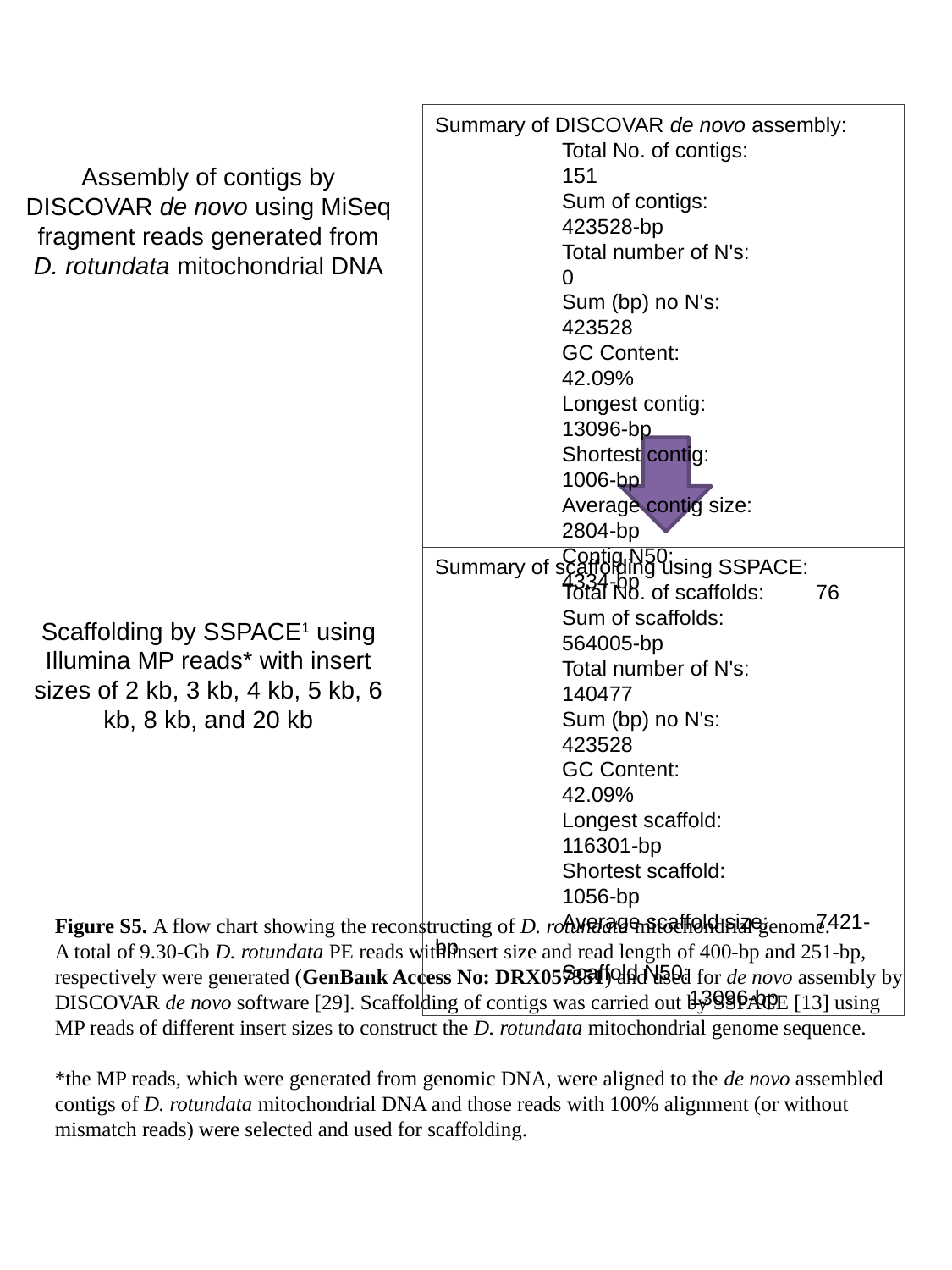

Summary of DISCOVAR de novo assembly:
	Total No. of contigs:		151
	Sum of contigs:		423528-bp
 	Total number of N's:		0
 	Sum (bp) no N's:		423528
	GC Content:			42.09%
	Longest contig:		13096-bp
	Shortest contig:		1006-bp
	Average contig size:		2804-bp
	Contig N50:			4334-bp
Assembly of contigs by DISCOVAR de novo using MiSeq fragment reads generated from D. rotundata mitochondrial DNA
Summary of scaffolding using SSPACE:
	Total No. of scaffolds:	76
	Sum of scaffolds:		564005-bp
 	Total number of N's:		140477
 	Sum (bp) no N's:		423528
	GC Content:			42.09%
	Longest scaffold:		116301-bp
	Shortest scaffold:		1056-bp
	Average scaffold size:	7421-bp
	Scaffold N50:			13096-bp
Scaffolding by SSPACE1 using Illumina MP reads* with insert sizes of 2 kb, 3 kb, 4 kb, 5 kb, 6 kb, 8 kb, and 20 kb
Figure S5. A flow chart showing the reconstructing of D. rotundata mitochondrial genome.
A total of 9.30-Gb D. rotundata PE reads with insert size and read length of 400-bp and 251-bp, respectively were generated (GenBank Access No: DRX057351) and used for de novo assembly by DISCOVAR de novo software [29]. Scaffolding of contigs was carried out by SSPACE [13] using MP reads of different insert sizes to construct the D. rotundata mitochondrial genome sequence.
*the MP reads, which were generated from genomic DNA, were aligned to the de novo assembled contigs of D. rotundata mitochondrial DNA and those reads with 100% alignment (or without mismatch reads) were selected and used for scaffolding.

## Slide 6
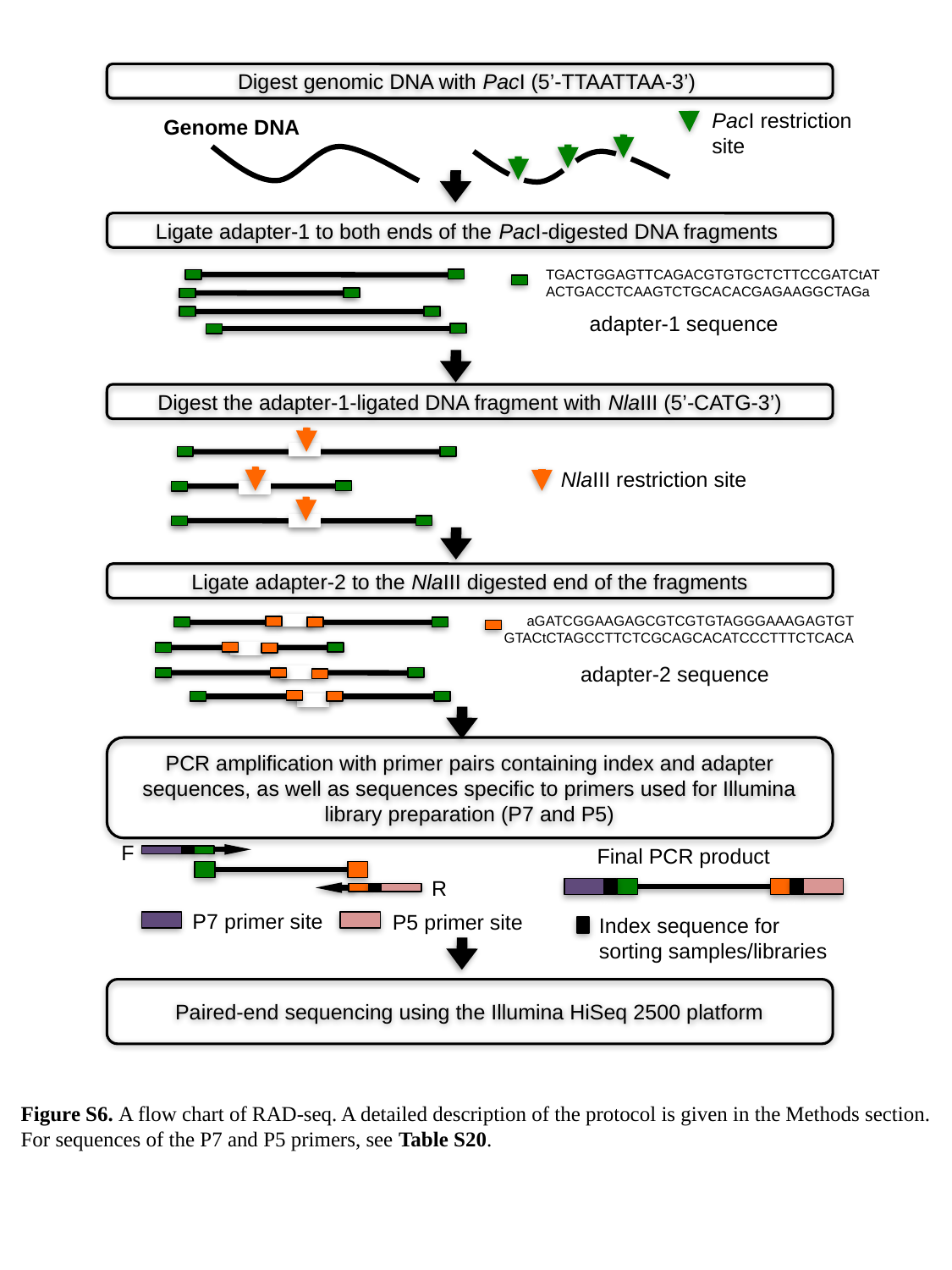

Digest genomic DNA with PacI (5’-TTAATTAA-3’)
PacI restriction site
Genome DNA
Ligate adapter-1 to both ends of the PacI-digested DNA fragments
TGACTGGAGTTCAGACGTGTGCTCTTCCGATCtAT
ACTGACCTCAAGTCTGCACACGAGAAGGCTAGa
adapter-1 sequence
Digest the adapter-1-ligated DNA fragment with NlaIII (5’-CATG-3’)
NlaIII restriction site
Ligate adapter-2 to the NlaIII digested end of the fragments
aGATCGGAAGAGCGTCGTGTAGGGAAAGAGTGT
GTACtCTAGCCTTCTCGCAGCACATCCCTTTCTCACA
PCR amplification with primer pairs containing index and adapter sequences, as well as sequences specific to primers used for Illumina library preparation (P7 and P5)
F
Final PCR product
R
P7 primer site
P5 primer site
Index sequence for sorting samples/libraries
Paired-end sequencing using the Illumina HiSeq 2500 platform
adapter-2 sequence
Figure S6. A flow chart of RAD-seq. A detailed description of the protocol is given in the Methods section. For sequences of the P7 and P5 primers, see Table S20.

## Slide 7
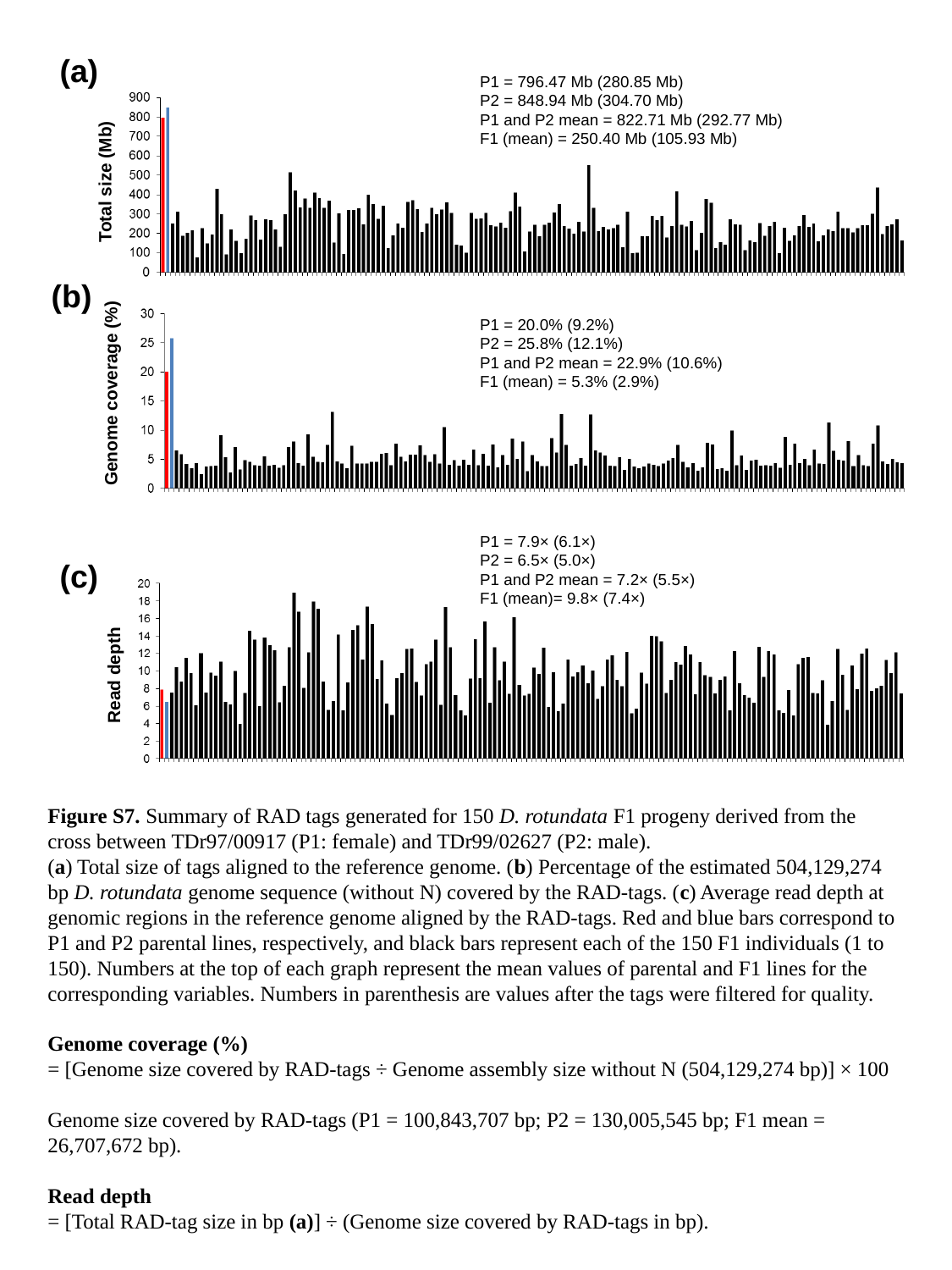

(a)
P1 = 796.47 Mb (280.85 Mb)
P2 = 848.94 Mb (304.70 Mb)
P1 and P2 mean = 822.71 Mb (292.77 Mb)
F1 (mean) = 250.40 Mb (105.93 Mb)
Total size (Mb)
(b)
P1 = 20.0% (9.2%)
P2 = 25.8% (12.1%)
P1 and P2 mean = 22.9% (10.6%)
F1 (mean) = 5.3% (2.9%)
Genome coverage (%)
P1 = 7.9× (6.1×)
P2 = 6.5× (5.0×)
P1 and P2 mean = 7.2× (5.5×)
F1 (mean)= 9.8× (7.4×)
(c)
Read depth
Figure S7. Summary of RAD tags generated for 150 D. rotundata F1 progeny derived from the cross between TDr97/00917 (P1: female) and TDr99/02627 (P2: male).
(a) Total size of tags aligned to the reference genome. (b) Percentage of the estimated 504,129,274 bp D. rotundata genome sequence (without N) covered by the RAD-tags. (c) Average read depth at genomic regions in the reference genome aligned by the RAD-tags. Red and blue bars correspond to P1 and P2 parental lines, respectively, and black bars represent each of the 150 F1 individuals (1 to 150). Numbers at the top of each graph represent the mean values of parental and F1 lines for the corresponding variables. Numbers in parenthesis are values after the tags were filtered for quality.
Genome coverage (%)
= [Genome size covered by RAD-tags ÷ Genome assembly size without N (504,129,274 bp)] × 100
Genome size covered by RAD-tags (P1 = 100,843,707 bp; P2 = 130,005,545 bp; F1 mean = 26,707,672 bp).
Read depth
= [Total RAD-tag size in bp (a)] ÷ (Genome size covered by RAD-tags in bp).

## Slide 8
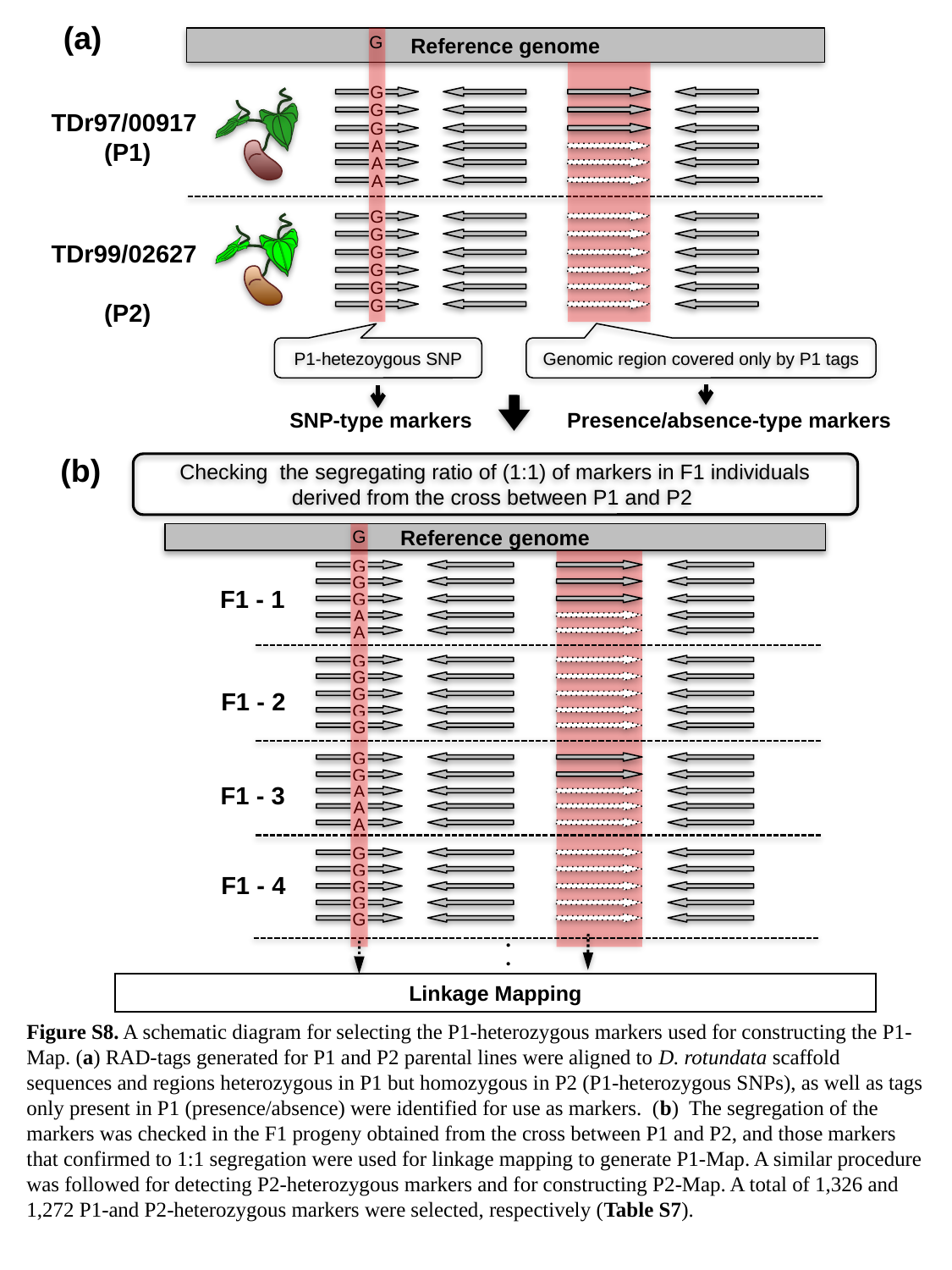

(a)
G
Reference genome
G
G
TDr97/00917
(P1)
G
A
A
A
G
G
TDr99/02627
(P2)
G
G
G
G
P1-hetezoygous SNP
Genomic region covered only by P1 tags
SNP-type markers
Presence/absence-type markers
(b)
Checking the segregating ratio of (1:1) of markers in F1 individuals derived from the cross between P1 and P2
G
G
G
F1 - 1
G
A
A
G
G
F1 - 2
G
G
G
G
G
F1 - 3
A
A
A
G
G
F1 - 4
G
G
G
Reference genome
・・
Linkage Mapping
Figure S8. A schematic diagram for selecting the P1-heterozygous markers used for constructing the P1-Map. (a) RAD-tags generated for P1 and P2 parental lines were aligned to D. rotundata scaffold sequences and regions heterozygous in P1 but homozygous in P2 (P1-heterozygous SNPs), as well as tags only present in P1 (presence/absence) were identified for use as markers. (b) The segregation of the markers was checked in the F1 progeny obtained from the cross between P1 and P2, and those markers that confirmed to 1:1 segregation were used for linkage mapping to generate P1-Map. A similar procedure was followed for detecting P2-heterozygous markers and for constructing P2-Map. A total of 1,326 and 1,272 P1-and P2-heterozygous markers were selected, respectively (Table S7).

## Slide 9
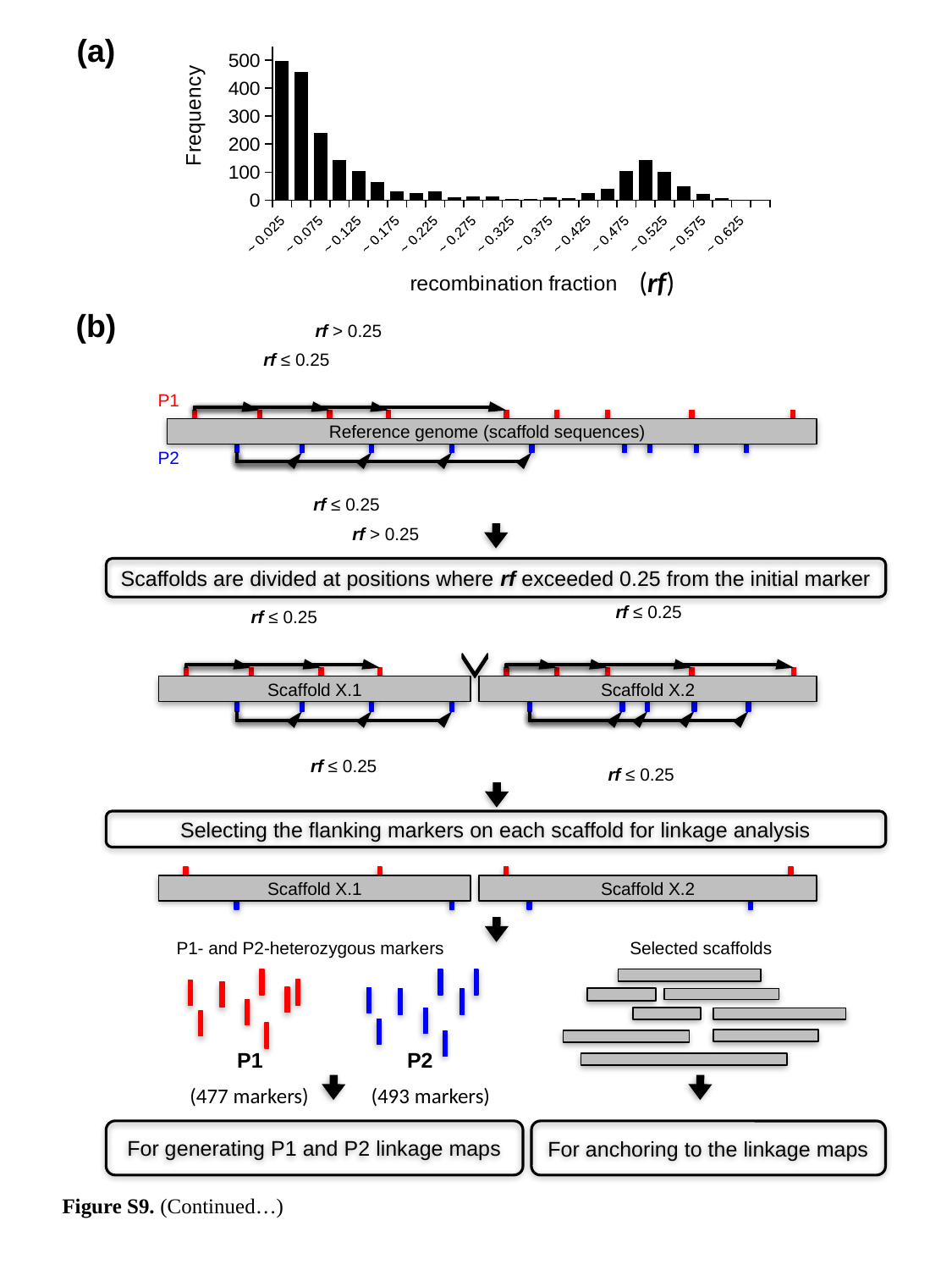

(a)
### Chart
| Category | | |
|---|---|---|
| ~ 0.025 | 243.0 | 254.0 |
| ~ 0.050 | 265.0 | 192.0 |
| ~ 0.075 | 105.0 | 133.0 |
| ~ 0.100 | 62.0 | 79.0 |
| ~ 0.125 | 57.0 | 44.0 |
| ~ 0.150 | 45.0 | 18.0 |
| ~ 0.175 | 16.0 | 13.0 |
| ~ 0.200 | 8.0 | 17.0 |
| ~ 0.225 | 21.0 | 9.0 |
| ~ 0.250 | 4.0 | 4.0 |
| ~ 0.275 | 5.0 | 6.0 |
| ~ 0.300 | 5.0 | 6.0 |
| ~ 0.325 | 3.0 | 1.0 |
| ~ 0.350 | 3.0 | 0.0 |
| ~ 0.375 | 8.0 | 1.0 |
| ~ 0.400 | 6.0 | 0.0 |
| ~ 0.425 | 19.0 | 5.0 |
| ~ 0.450 | 30.0 | 10.0 |
| ~ 0.475 | 46.0 | 58.0 |
| ~ 0.500 | 66.0 | 75.0 |
| ~ 0.525 | 42.0 | 58.0 |
| ~ 0.550 | 19.0 | 28.0 |
| ~ 0.575 | 8.0 | 14.0 |
| ~ 0.600 | 1.0 | 4.0 |
| ~ 0.625 | 0.0 | 1.0 |
| ~ 0.650 | 0.0 | 0.0 |(rf)
(b)
rf > 0.25
rf ≤ 0.25
P1
Reference genome (scaffold sequences)
P2
rf ≤ 0.25
rf > 0.25
Scaffolds are divided at positions where rf exceeded 0.25 from the initial marker
rf ≤ 0.25
rf ≤ 0.25
Scaffold X.1
Scaffold X.2
rf ≤ 0.25
rf ≤ 0.25
Selecting the flanking markers on each scaffold for linkage analysis
Scaffold X.1
Scaffold X.2
P1- and P2-heterozygous markers
Selected scaffolds
P2
P1
(477 markers)
(493 markers)
For generating P1 and P2 linkage maps
For anchoring to the linkage maps
Figure S9. (Continued…)

## Slide 10
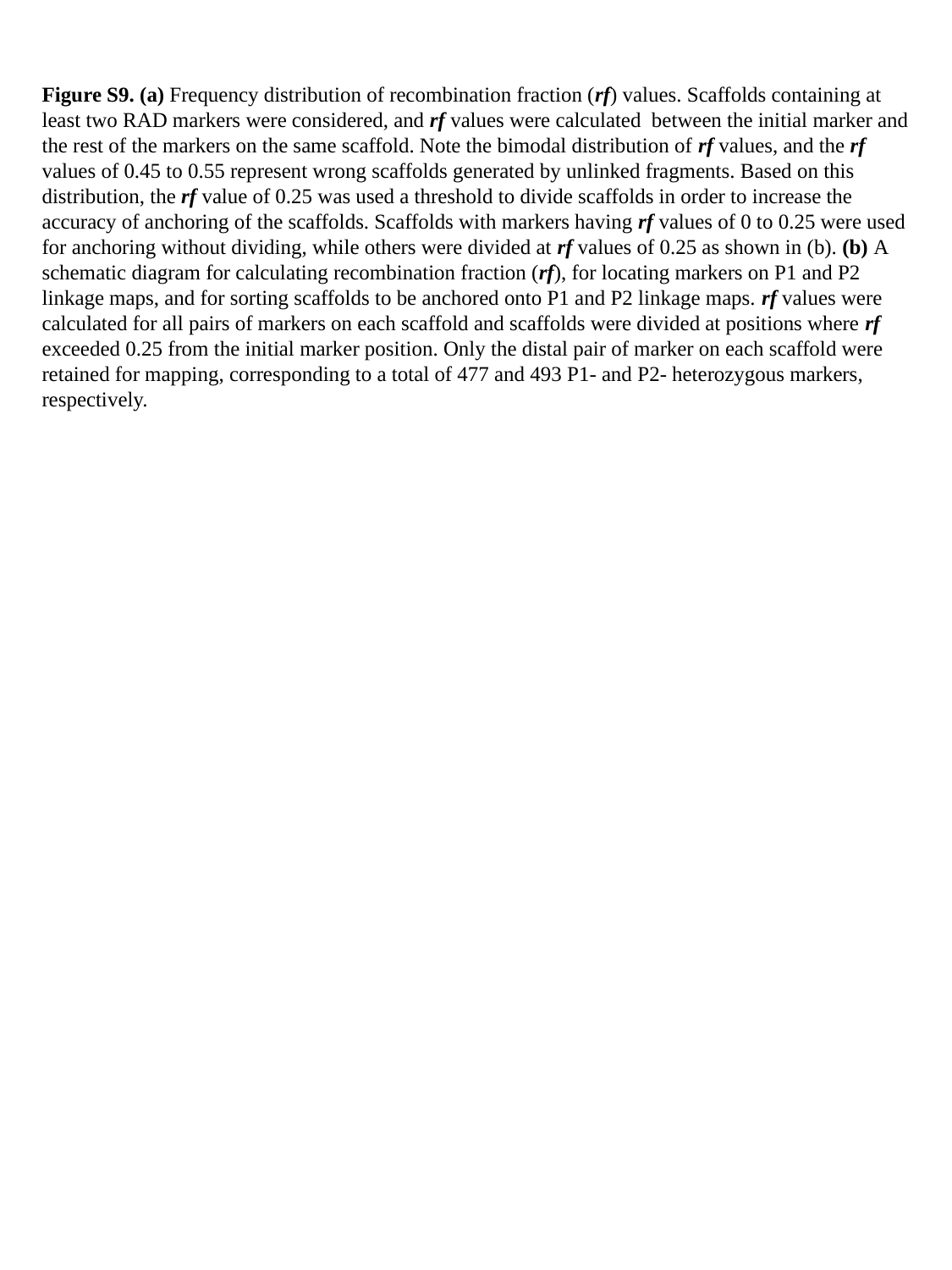

Figure S9. (a) Frequency distribution of recombination fraction (rf) values. Scaffolds containing at least two RAD markers were considered, and rf values were calculated between the initial marker and the rest of the markers on the same scaffold. Note the bimodal distribution of rf values, and the rf values of 0.45 to 0.55 represent wrong scaffolds generated by unlinked fragments. Based on this distribution, the rf value of 0.25 was used a threshold to divide scaffolds in order to increase the accuracy of anchoring of the scaffolds. Scaffolds with markers having rf values of 0 to 0.25 were used for anchoring without dividing, while others were divided at rf values of 0.25 as shown in (b). (b) A schematic diagram for calculating recombination fraction (rf), for locating markers on P1 and P2 linkage maps, and for sorting scaffolds to be anchored onto P1 and P2 linkage maps. rf values were calculated for all pairs of markers on each scaffold and scaffolds were divided at positions where rf exceeded 0.25 from the initial marker position. Only the distal pair of marker on each scaffold were retained for mapping, corresponding to a total of 477 and 493 P1- and P2- heterozygous markers, respectively.

## Slide 11
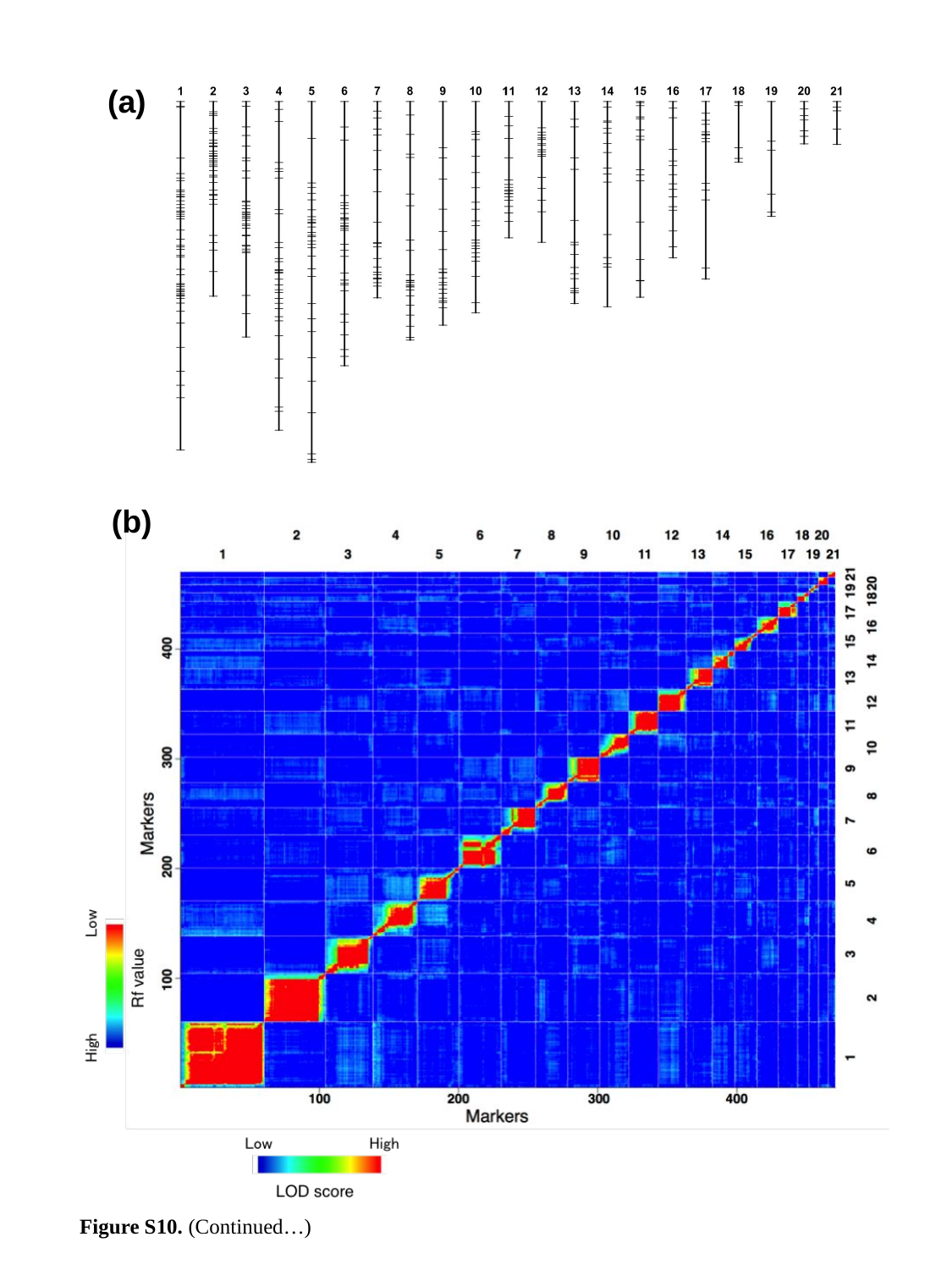

(a)
(b)
Figure S10. (Continued…)

## Slide 12
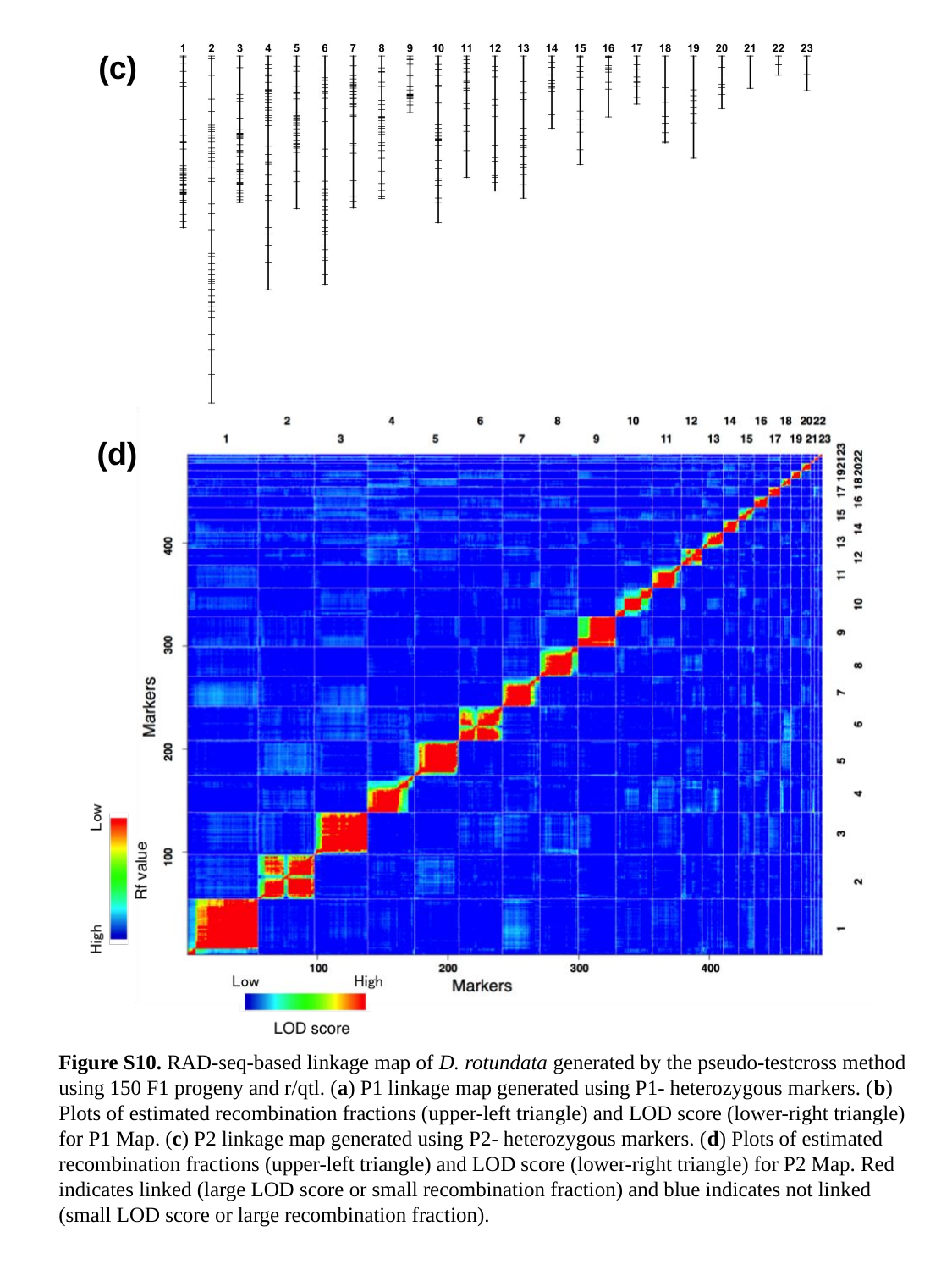

(c)
(d)
Figure S10. RAD-seq-based linkage map of D. rotundata generated by the pseudo-testcross method using 150 F1 progeny and r/qtl. (a) P1 linkage map generated using P1- heterozygous markers. (b) Plots of estimated recombination fractions (upper-left triangle) and LOD score (lower-right triangle) for P1 Map. (c) P2 linkage map generated using P2- heterozygous markers. (d) Plots of estimated recombination fractions (upper-left triangle) and LOD score (lower-right triangle) for P2 Map. Red indicates linked (large LOD score or small recombination fraction) and blue indicates not linked (small LOD score or large recombination fraction).

## Slide 13
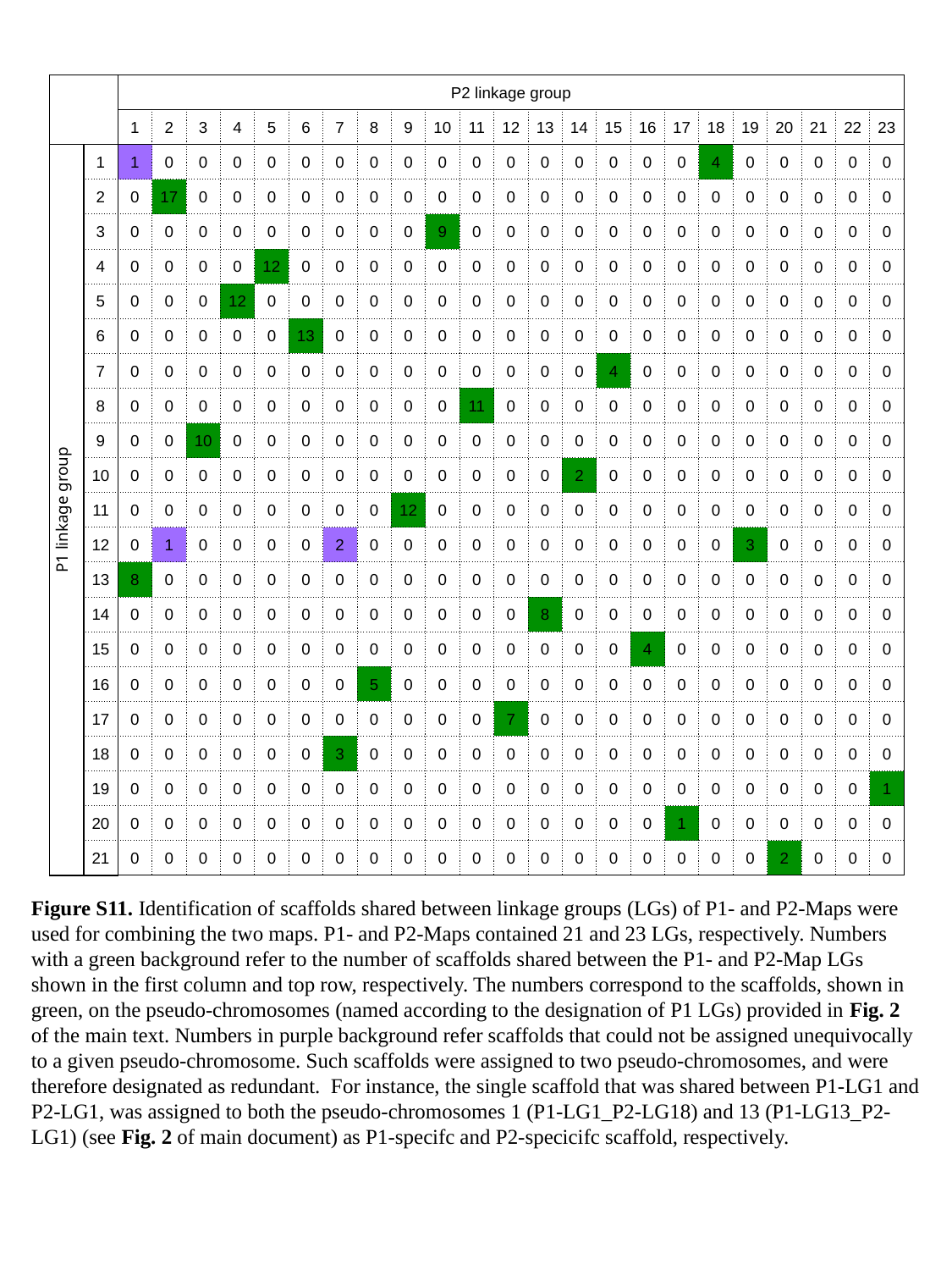

| | | P2 linkage group | | | | | | | | | | | | | | | | | | | | | | |
| --- | --- | --- | --- | --- | --- | --- | --- | --- | --- | --- | --- | --- | --- | --- | --- | --- | --- | --- | --- | --- | --- | --- | --- | --- |
| | | 1 | 2 | 3 | 4 | 5 | 6 | 7 | 8 | 9 | 10 | 11 | 12 | 13 | 14 | 15 | 16 | 17 | 18 | 19 | 20 | 21 | 22 | 23 |
| P1 linkage group | 1 | 1 | 0 | 0 | 0 | 0 | 0 | 0 | 0 | 0 | 0 | 0 | 0 | 0 | 0 | 0 | 0 | 0 | 4 | 0 | 0 | 0 | 0 | 0 |
| | 2 | 0 | 17 | 0 | 0 | 0 | 0 | 0 | 0 | 0 | 0 | 0 | 0 | 0 | 0 | 0 | 0 | 0 | 0 | 0 | 0 | 0 | 0 | 0 |
| | 3 | 0 | 0 | 0 | 0 | 0 | 0 | 0 | 0 | 0 | 9 | 0 | 0 | 0 | 0 | 0 | 0 | 0 | 0 | 0 | 0 | 0 | 0 | 0 |
| | 4 | 0 | 0 | 0 | 0 | 12 | 0 | 0 | 0 | 0 | 0 | 0 | 0 | 0 | 0 | 0 | 0 | 0 | 0 | 0 | 0 | 0 | 0 | 0 |
| | 5 | 0 | 0 | 0 | 12 | 0 | 0 | 0 | 0 | 0 | 0 | 0 | 0 | 0 | 0 | 0 | 0 | 0 | 0 | 0 | 0 | 0 | 0 | 0 |
| | 6 | 0 | 0 | 0 | 0 | 0 | 13 | 0 | 0 | 0 | 0 | 0 | 0 | 0 | 0 | 0 | 0 | 0 | 0 | 0 | 0 | 0 | 0 | 0 |
| | 7 | 0 | 0 | 0 | 0 | 0 | 0 | 0 | 0 | 0 | 0 | 0 | 0 | 0 | 0 | 4 | 0 | 0 | 0 | 0 | 0 | 0 | 0 | 0 |
| | 8 | 0 | 0 | 0 | 0 | 0 | 0 | 0 | 0 | 0 | 0 | 11 | 0 | 0 | 0 | 0 | 0 | 0 | 0 | 0 | 0 | 0 | 0 | 0 |
| | 9 | 0 | 0 | 10 | 0 | 0 | 0 | 0 | 0 | 0 | 0 | 0 | 0 | 0 | 0 | 0 | 0 | 0 | 0 | 0 | 0 | 0 | 0 | 0 |
| | 10 | 0 | 0 | 0 | 0 | 0 | 0 | 0 | 0 | 0 | 0 | 0 | 0 | 0 | 2 | 0 | 0 | 0 | 0 | 0 | 0 | 0 | 0 | 0 |
| | 11 | 0 | 0 | 0 | 0 | 0 | 0 | 0 | 0 | 12 | 0 | 0 | 0 | 0 | 0 | 0 | 0 | 0 | 0 | 0 | 0 | 0 | 0 | 0 |
| | 12 | 0 | 1 | 0 | 0 | 0 | 0 | 2 | 0 | 0 | 0 | 0 | 0 | 0 | 0 | 0 | 0 | 0 | 0 | 3 | 0 | 0 | 0 | 0 |
| | 13 | 8 | 0 | 0 | 0 | 0 | 0 | 0 | 0 | 0 | 0 | 0 | 0 | 0 | 0 | 0 | 0 | 0 | 0 | 0 | 0 | 0 | 0 | 0 |
| | 14 | 0 | 0 | 0 | 0 | 0 | 0 | 0 | 0 | 0 | 0 | 0 | 0 | 8 | 0 | 0 | 0 | 0 | 0 | 0 | 0 | 0 | 0 | 0 |
| | 15 | 0 | 0 | 0 | 0 | 0 | 0 | 0 | 0 | 0 | 0 | 0 | 0 | 0 | 0 | 0 | 4 | 0 | 0 | 0 | 0 | 0 | 0 | 0 |
| | 16 | 0 | 0 | 0 | 0 | 0 | 0 | 0 | 5 | 0 | 0 | 0 | 0 | 0 | 0 | 0 | 0 | 0 | 0 | 0 | 0 | 0 | 0 | 0 |
| | 17 | 0 | 0 | 0 | 0 | 0 | 0 | 0 | 0 | 0 | 0 | 0 | 7 | 0 | 0 | 0 | 0 | 0 | 0 | 0 | 0 | 0 | 0 | 0 |
| | 18 | 0 | 0 | 0 | 0 | 0 | 0 | 3 | 0 | 0 | 0 | 0 | 0 | 0 | 0 | 0 | 0 | 0 | 0 | 0 | 0 | 0 | 0 | 0 |
| | 19 | 0 | 0 | 0 | 0 | 0 | 0 | 0 | 0 | 0 | 0 | 0 | 0 | 0 | 0 | 0 | 0 | 0 | 0 | 0 | 0 | 0 | 0 | 1 |
| | 20 | 0 | 0 | 0 | 0 | 0 | 0 | 0 | 0 | 0 | 0 | 0 | 0 | 0 | 0 | 0 | 0 | 1 | 0 | 0 | 0 | 0 | 0 | 0 |
| | 21 | 0 | 0 | 0 | 0 | 0 | 0 | 0 | 0 | 0 | 0 | 0 | 0 | 0 | 0 | 0 | 0 | 0 | 0 | 0 | 2 | 0 | 0 | 0 |
Figure S11. Identification of scaffolds shared between linkage groups (LGs) of P1- and P2-Maps were used for combining the two maps. P1- and P2-Maps contained 21 and 23 LGs, respectively. Numbers with a green background refer to the number of scaffolds shared between the P1- and P2-Map LGs shown in the first column and top row, respectively. The numbers correspond to the scaffolds, shown in green, on the pseudo-chromosomes (named according to the designation of P1 LGs) provided in Fig. 2 of the main text. Numbers in purple background refer scaffolds that could not be assigned unequivocally to a given pseudo-chromosome. Such scaffolds were assigned to two pseudo-chromosomes, and were therefore designated as redundant. For instance, the single scaffold that was shared between P1-LG1 and P2-LG1, was assigned to both the pseudo-chromosomes 1 (P1-LG1_P2-LG18) and 13 (P1-LG13_P2-LG1) (see Fig. 2 of main document) as P1-specifc and P2-specicifc scaffold, respectively.

## Slide 14
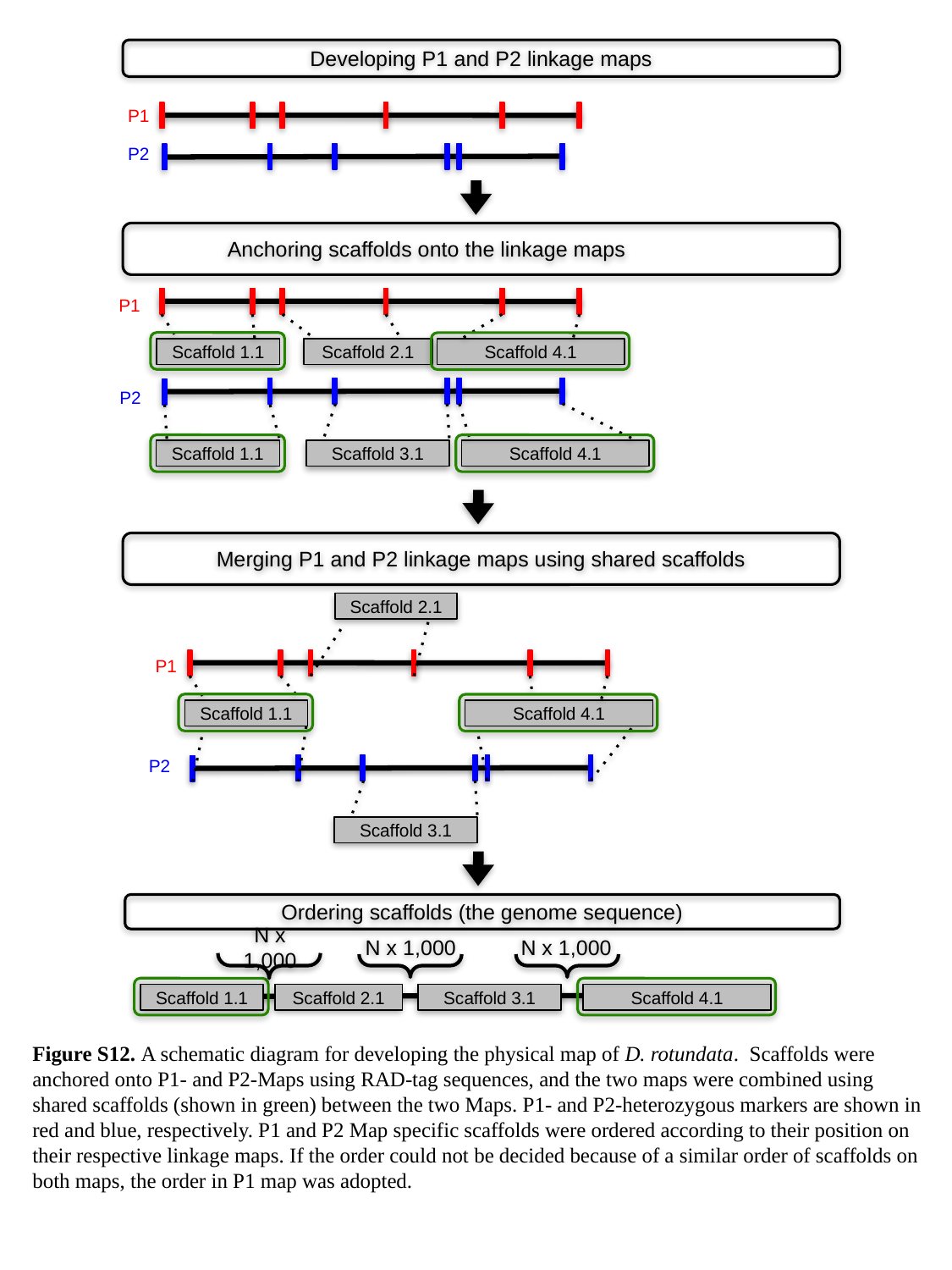

Developing P1 and P2 linkage maps
P1
P2
Anchoring scaffolds onto the linkage maps
P1
Scaffold 1.1
Scaffold 2.1
Scaffold 4.1
P2
Scaffold 1.1
Scaffold 3.1
Scaffold 4.1
Merging P1 and P2 linkage maps using shared scaffolds
Scaffold 2.1
P1
Scaffold 1.1
Scaffold 4.1
P2
Scaffold 3.1
Ordering scaffolds (the genome sequence)
N x 1,000
N x 1,000
N x 1,000
Scaffold 4.1
Scaffold 1.1
Scaffold 2.1
Scaffold 3.1
Figure S12. A schematic diagram for developing the physical map of D. rotundata. Scaffolds were anchored onto P1- and P2-Maps using RAD-tag sequences, and the two maps were combined using shared scaffolds (shown in green) between the two Maps. P1- and P2-heterozygous markers are shown in red and blue, respectively. P1 and P2 Map specific scaffolds were ordered according to their position on their respective linkage maps. If the order could not be decided because of a similar order of scaffolds on both maps, the order in P1 map was adopted.

## Slide 15
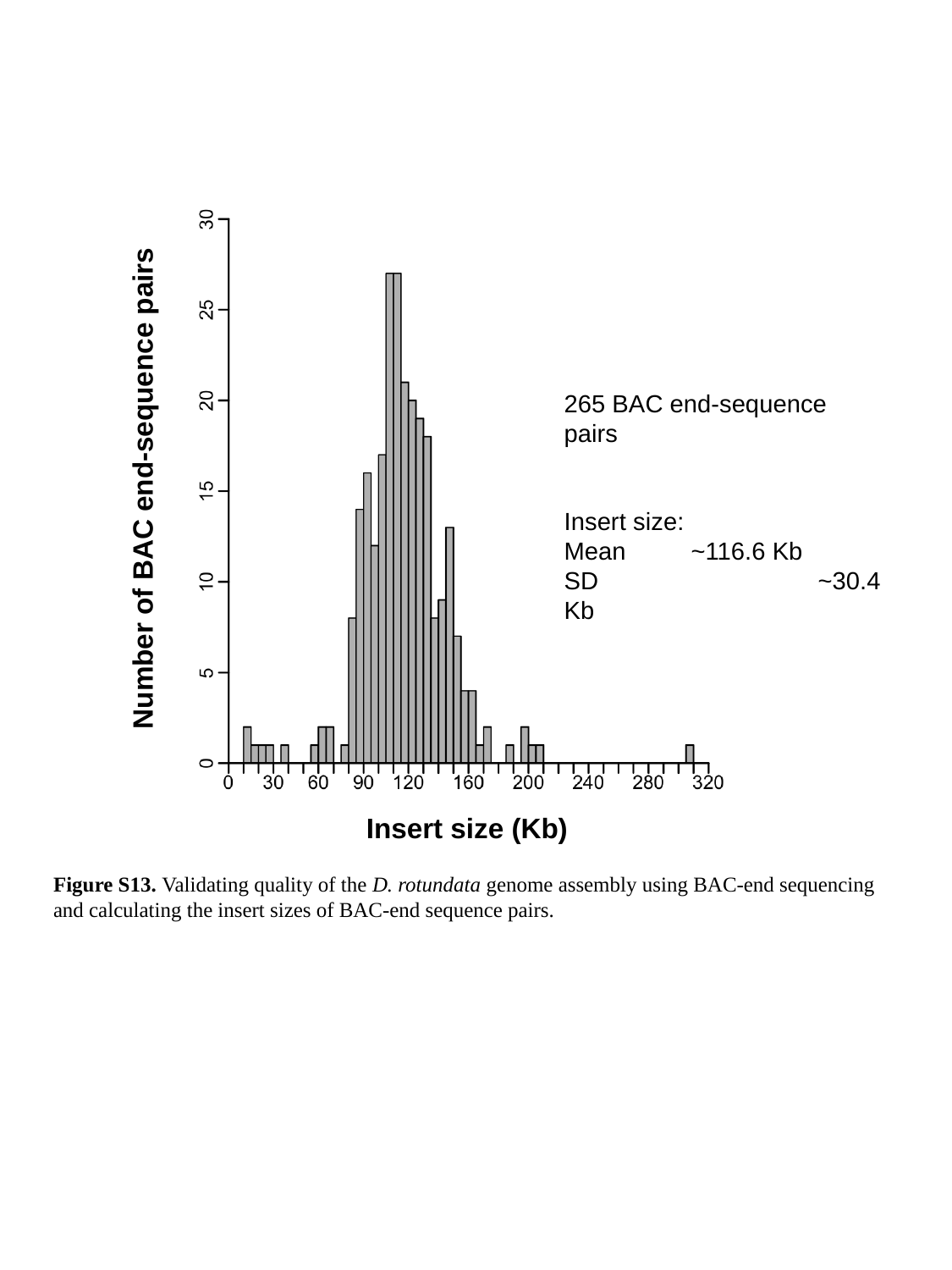

Number of BAC end-sequence pairs
Insert size (Kb)
265 BAC end-sequence pairs
Insert size:
Mean	~116.6 Kb
SD		~30.4 Kb
Figure S13. Validating quality of the D. rotundata genome assembly using BAC-end sequencing and calculating the insert sizes of BAC-end sequence pairs.

## Slide 16
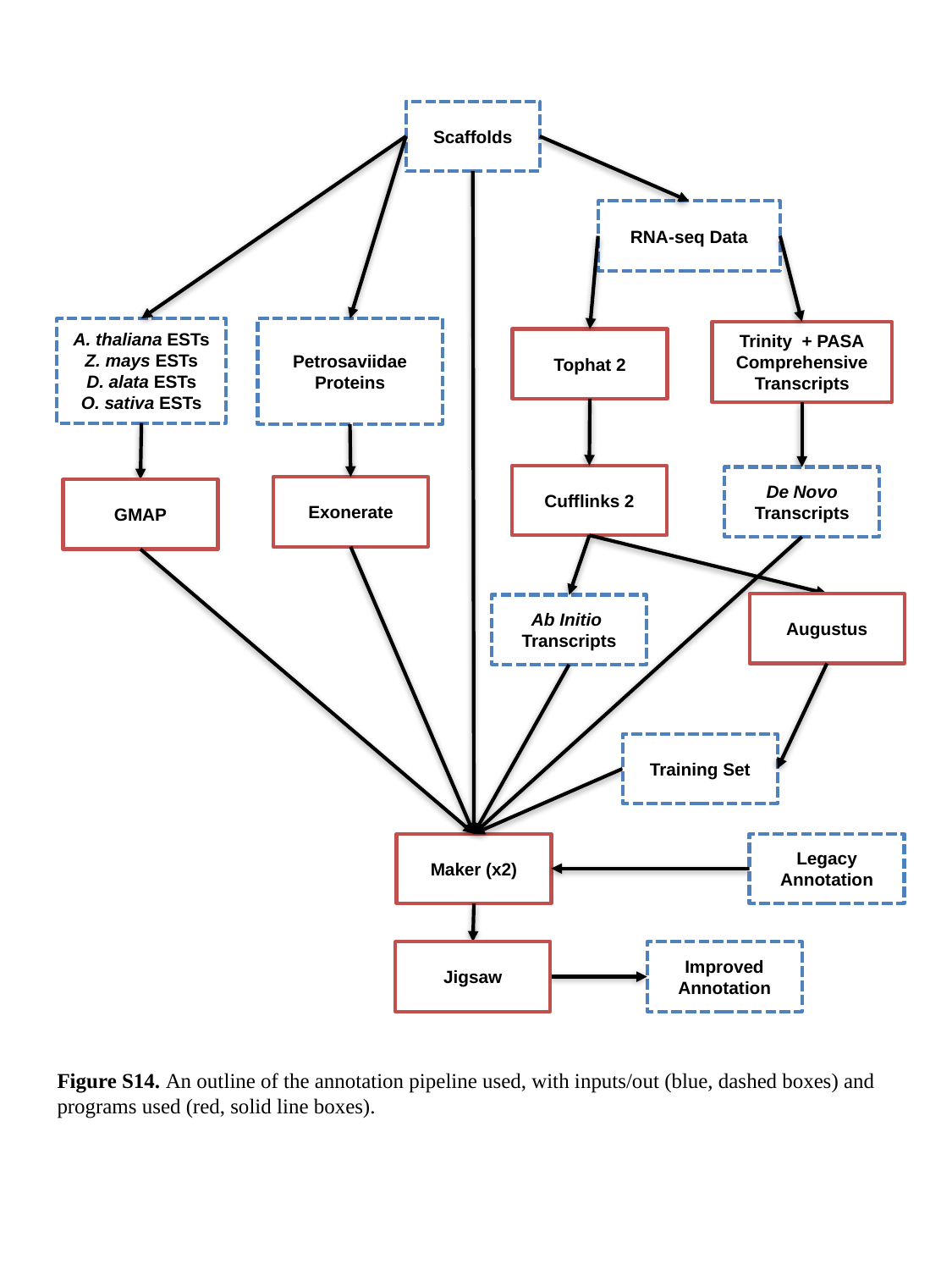

Scaffolds
RNA-seq Data
A. thaliana ESTs
Z. mays ESTs
D. alata ESTs
O. sativa ESTs
Petrosaviidae Proteins
Trinity + PASA
Comprehensive Transcripts
Tophat 2
Cufflinks 2
De Novo Transcripts
Exonerate
GMAP
Augustus
Ab Initio Transcripts
Training Set
Maker (x2)
Legacy Annotation
Jigsaw
Improved Annotation
Figure S14. An outline of the annotation pipeline used, with inputs/out (blue, dashed boxes) and programs used (red, solid line boxes).

## Slide 17
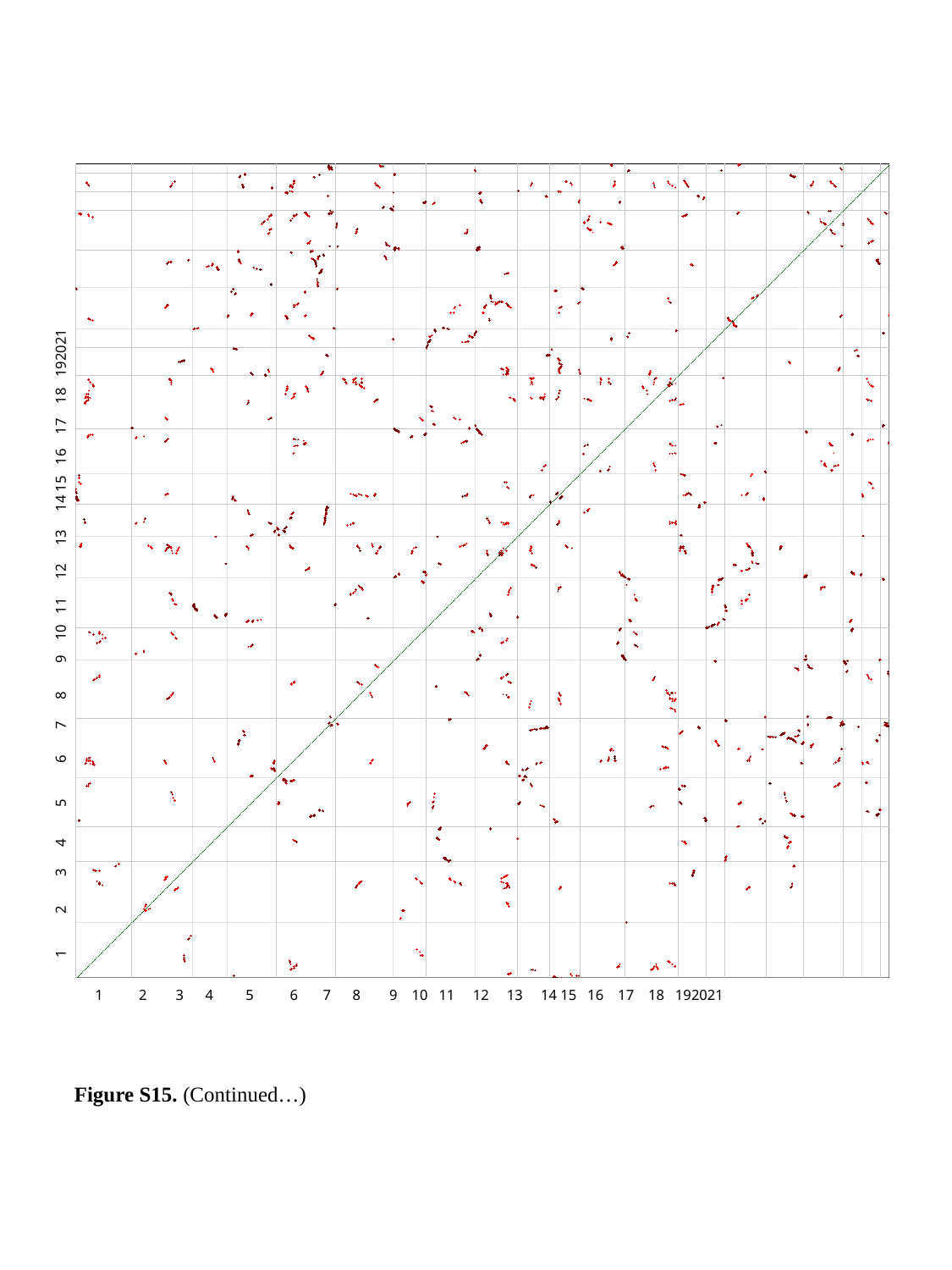

1 2 3 4 5 6 7 8 9 10 11 12 13 14 15 16 17 18 192021
 1 2 3 4 5 6 7 8 9 10 11 12 13 14 15 16 17 18 192021
Figure S15. (Continued…)

## Slide 18
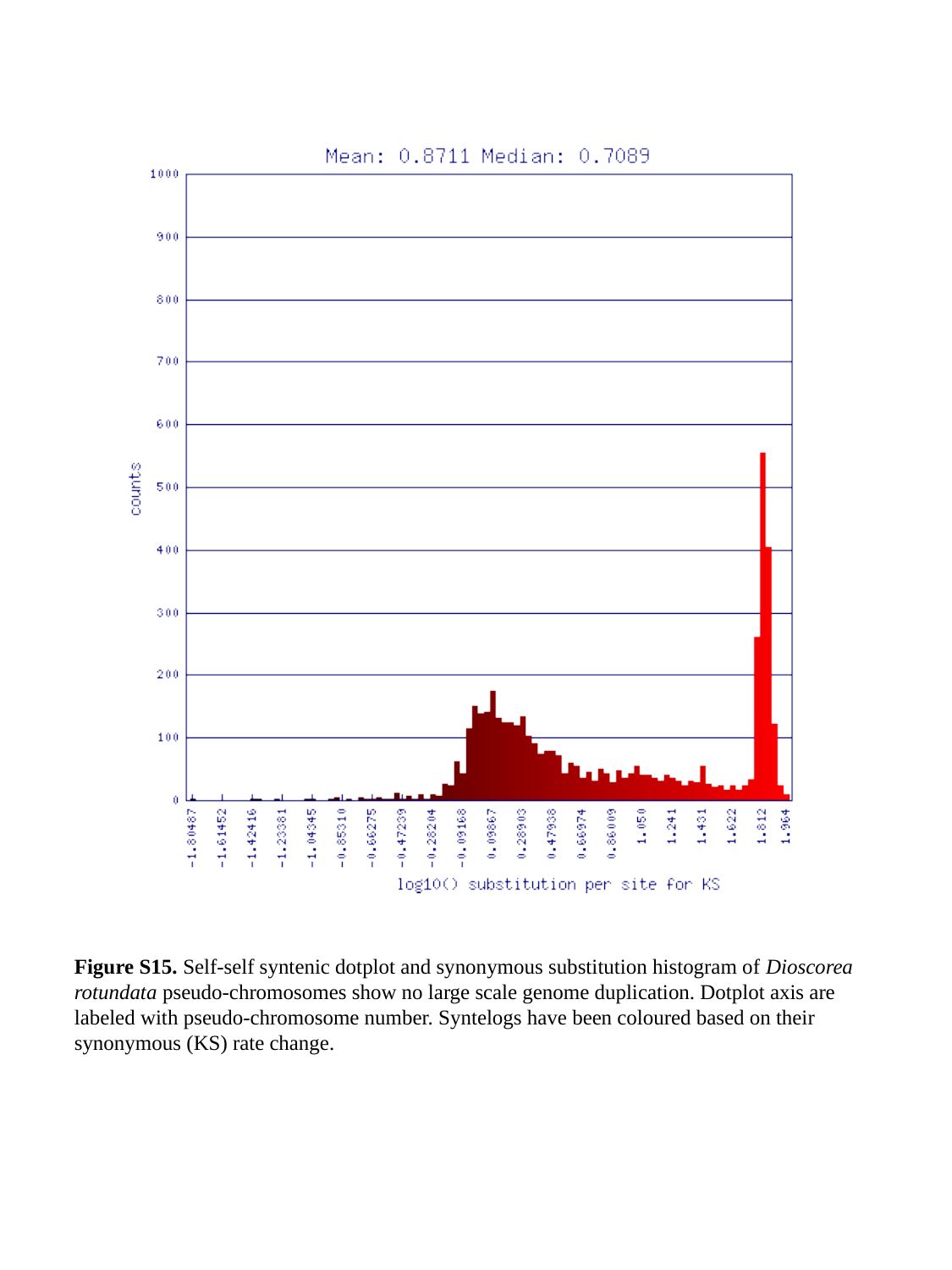

Figure S15. Self-self syntenic dotplot and synonymous substitution histogram of Dioscorea rotundata pseudo-chromosomes show no large scale genome duplication. Dotplot axis are labeled with pseudo-chromosome number. Syntelogs have been coloured based on their synonymous (KS) rate change.

## Slide 19
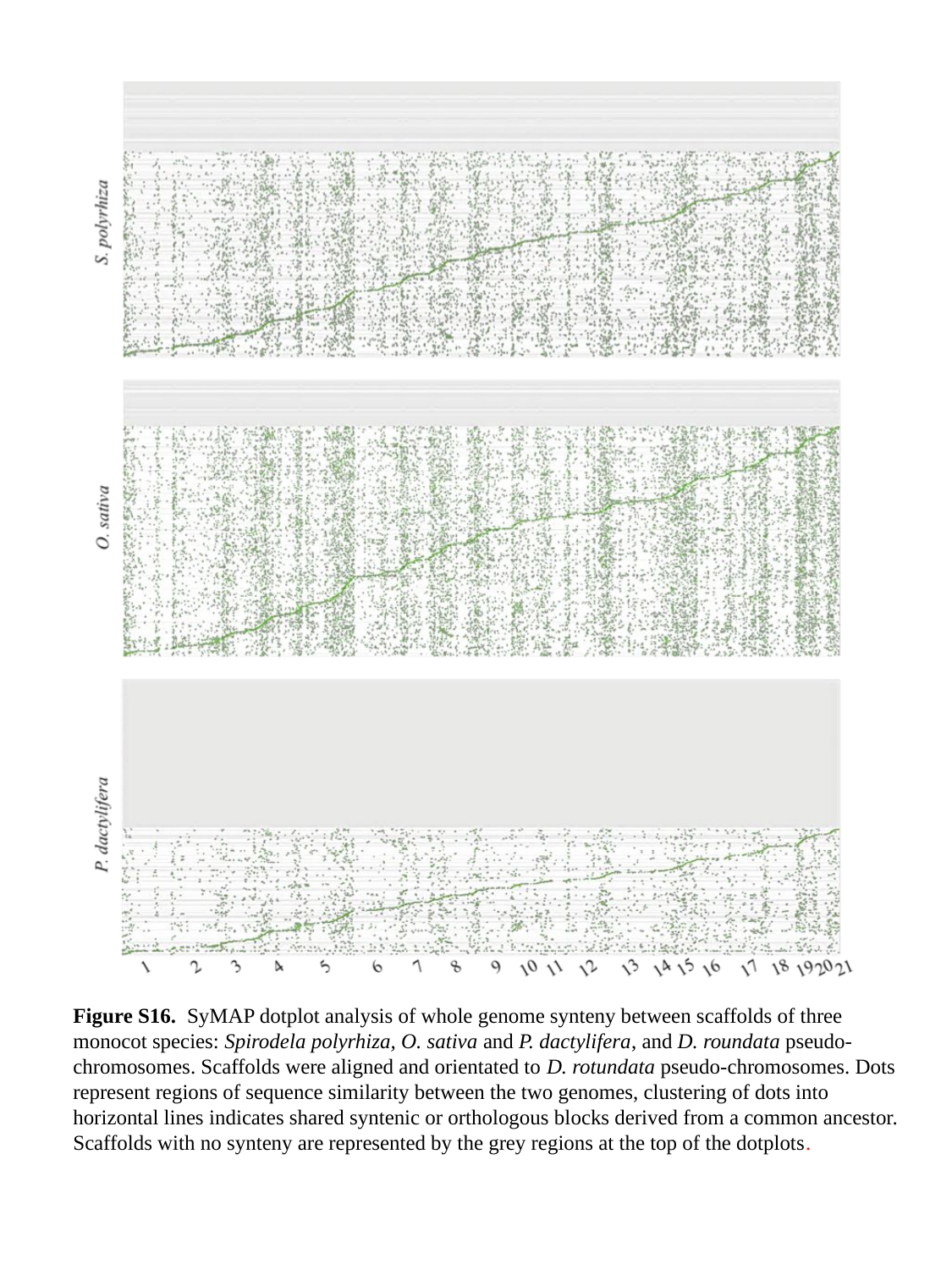

Figure S16. SyMAP dotplot analysis of whole genome synteny between scaffolds of three monocot species: Spirodela polyrhiza, O. sativa and P. dactylifera, and D. roundata pseudo-chromosomes. Scaffolds were aligned and orientated to D. rotundata pseudo-chromosomes. Dots represent regions of sequence similarity between the two genomes, clustering of dots into horizontal lines indicates shared syntenic or orthologous blocks derived from a common ancestor. Scaffolds with no synteny are represented by the grey regions at the top of the dotplots.

## Slide 20
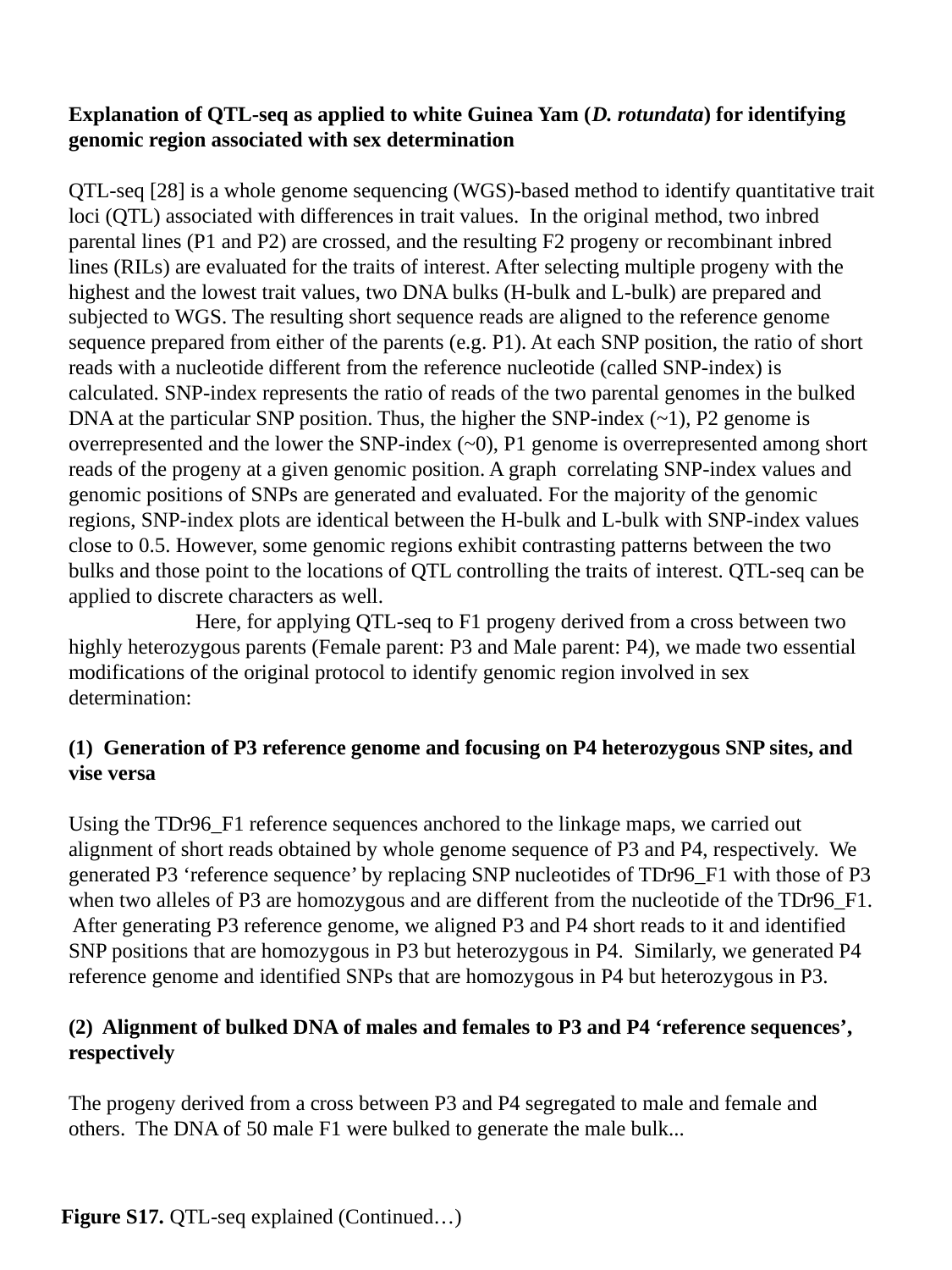

Explanation of QTL-seq as applied to white Guinea Yam (D. rotundata) for identifying genomic region associated with sex determination
QTL-seq [28] is a whole genome sequencing (WGS)-based method to identify quantitative trait loci (QTL) associated with differences in trait values. In the original method, two inbred parental lines (P1 and P2) are crossed, and the resulting F2 progeny or recombinant inbred lines (RILs) are evaluated for the traits of interest. After selecting multiple progeny with the highest and the lowest trait values, two DNA bulks (H-bulk and L-bulk) are prepared and subjected to WGS. The resulting short sequence reads are aligned to the reference genome sequence prepared from either of the parents (e.g. P1). At each SNP position, the ratio of short reads with a nucleotide different from the reference nucleotide (called SNP-index) is calculated. SNP-index represents the ratio of reads of the two parental genomes in the bulked DNA at the particular SNP position. Thus, the higher the SNP-index (~1), P2 genome is overrepresented and the lower the SNP-index (~0), P1 genome is overrepresented among short reads of the progeny at a given genomic position. A graph correlating SNP-index values and genomic positions of SNPs are generated and evaluated. For the majority of the genomic regions, SNP-index plots are identical between the H-bulk and L-bulk with SNP-index values close to 0.5. However, some genomic regions exhibit contrasting patterns between the two bulks and those point to the locations of QTL controlling the traits of interest. QTL-seq can be applied to discrete characters as well.
	Here, for applying QTL-seq to F1 progeny derived from a cross between two highly heterozygous parents (Female parent: P3 and Male parent: P4), we made two essential modifications of the original protocol to identify genomic region involved in sex determination:
(1) Generation of P3 reference genome and focusing on P4 heterozygous SNP sites, and vise versa
Using the TDr96_F1 reference sequences anchored to the linkage maps, we carried out alignment of short reads obtained by whole genome sequence of P3 and P4, respectively. We generated P3 ‘reference sequence’ by replacing SNP nucleotides of TDr96_F1 with those of P3 when two alleles of P3 are homozygous and are different from the nucleotide of the TDr96_F1. After generating P3 reference genome, we aligned P3 and P4 short reads to it and identified SNP positions that are homozygous in P3 but heterozygous in P4. Similarly, we generated P4 reference genome and identified SNPs that are homozygous in P4 but heterozygous in P3.
(2) Alignment of bulked DNA of males and females to P3 and P4 ‘reference sequences’, respectively
The progeny derived from a cross between P3 and P4 segregated to male and female and others. The DNA of 50 male F1 were bulked to generate the male bulk...
Figure S17. QTL-seq explained (Continued…)

## Slide 21
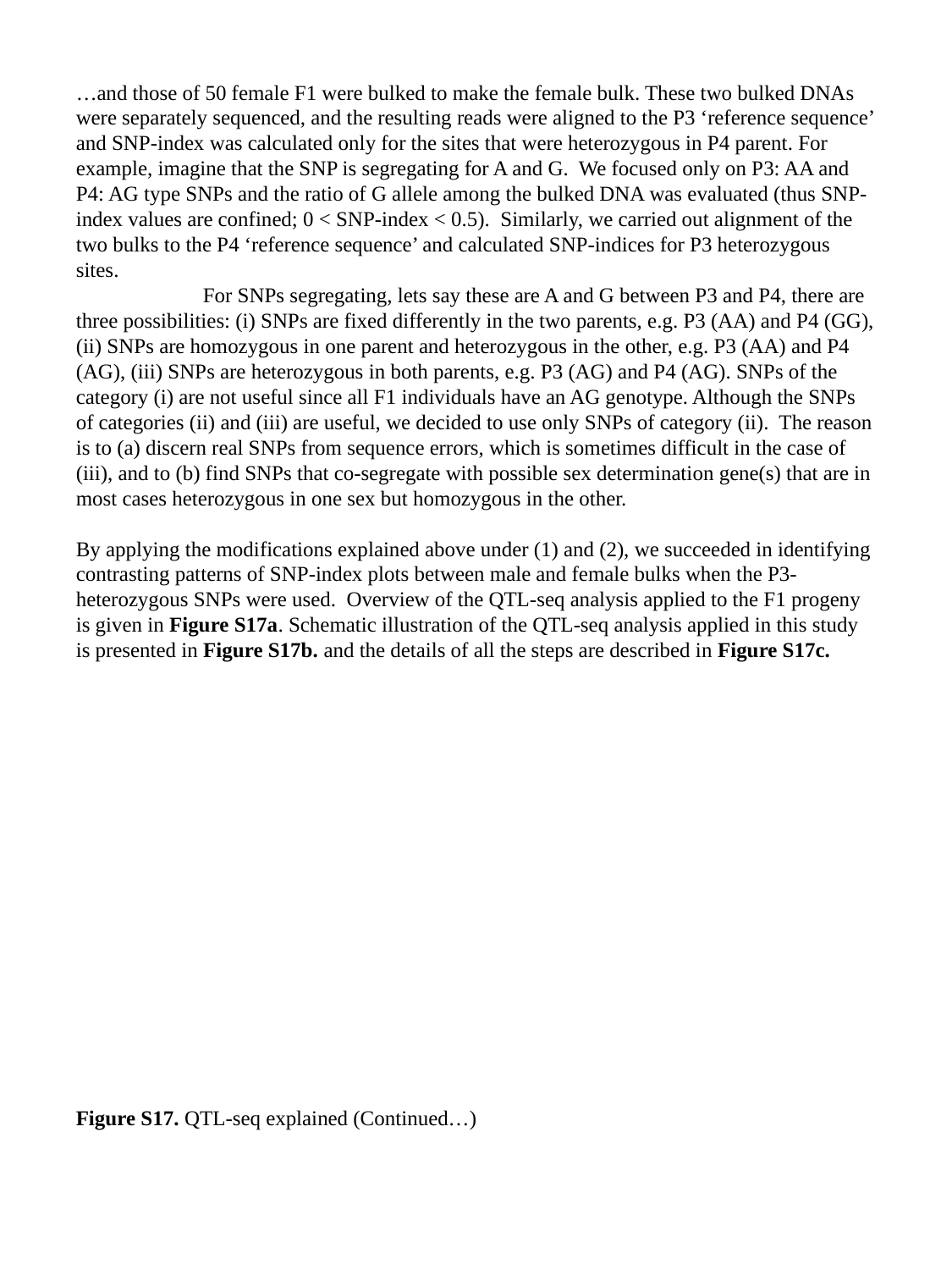

…and those of 50 female F1 were bulked to make the female bulk. These two bulked DNAs were separately sequenced, and the resulting reads were aligned to the P3 ‘reference sequence’ and SNP-index was calculated only for the sites that were heterozygous in P4 parent. For example, imagine that the SNP is segregating for A and G. We focused only on P3: AA and P4: AG type SNPs and the ratio of G allele among the bulked DNA was evaluated (thus SNP-index values are confined; 0 < SNP-index < 0.5). Similarly, we carried out alignment of the two bulks to the P4 ‘reference sequence’ and calculated SNP-indices for P3 heterozygous sites.
	For SNPs segregating, lets say these are A and G between P3 and P4, there are three possibilities: (i) SNPs are fixed differently in the two parents, e.g. P3 (AA) and P4 (GG), (ii) SNPs are homozygous in one parent and heterozygous in the other, e.g. P3 (AA) and P4 (AG), (iii) SNPs are heterozygous in both parents, e.g. P3 (AG) and P4 (AG). SNPs of the category (i) are not useful since all F1 individuals have an AG genotype. Although the SNPs of categories (ii) and (iii) are useful, we decided to use only SNPs of category (ii). The reason is to (a) discern real SNPs from sequence errors, which is sometimes difficult in the case of (iii), and to (b) find SNPs that co-segregate with possible sex determination gene(s) that are in most cases heterozygous in one sex but homozygous in the other.
By applying the modifications explained above under (1) and (2), we succeeded in identifying contrasting patterns of SNP-index plots between male and female bulks when the P3-heterozygous SNPs were used. Overview of the QTL-seq analysis applied to the F1 progeny is given in Figure S17a. Schematic illustration of the QTL-seq analysis applied in this study is presented in Figure S17b. and the details of all the steps are described in Figure S17c.
Figure S17. QTL-seq explained (Continued…)

## Slide 22
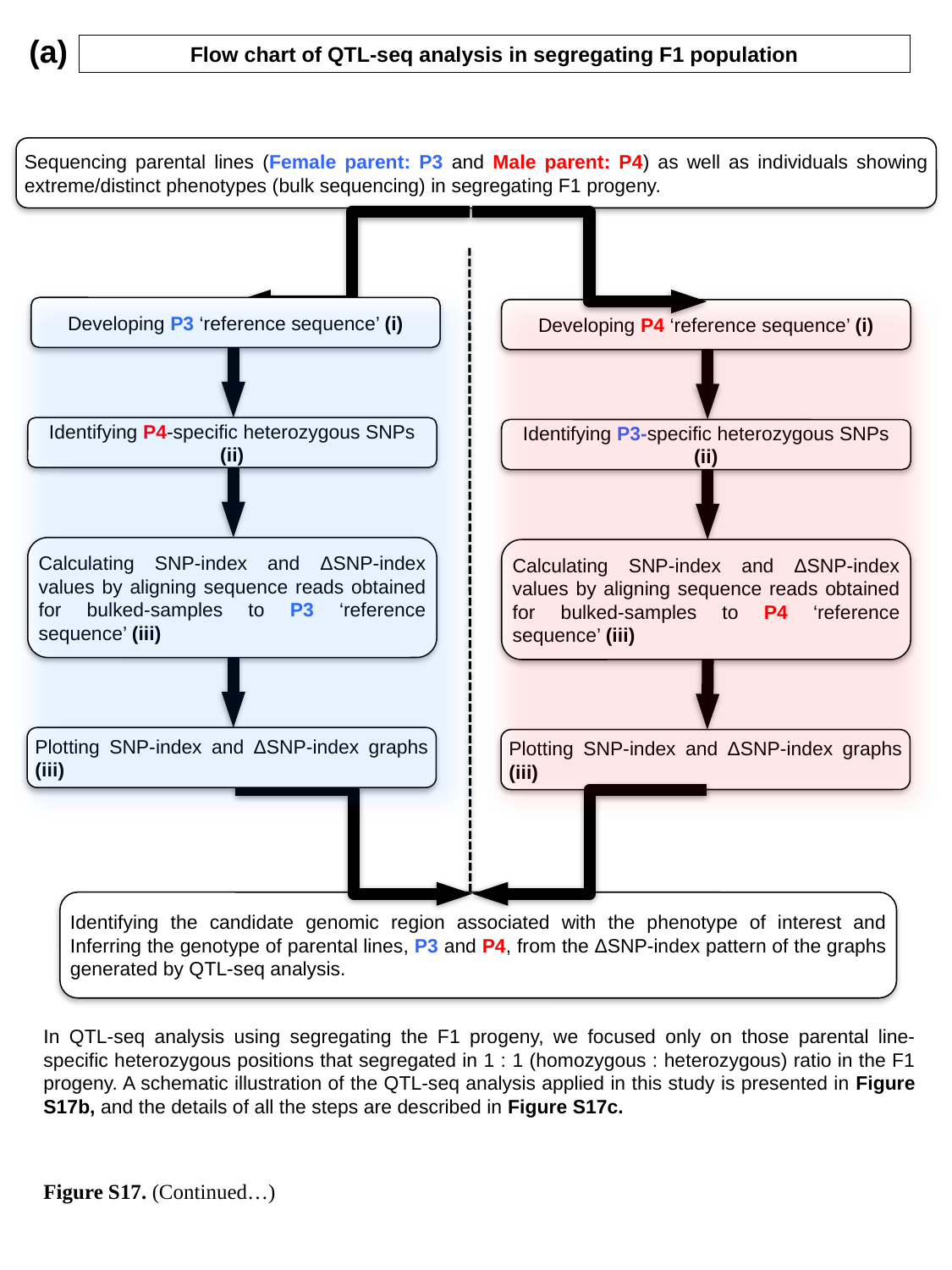

(a)
Flow chart of QTL-seq analysis in segregating F1 population
Sequencing parental lines (Female parent: P3 and Male parent: P4) as well as individuals showing extreme/distinct phenotypes (bulk sequencing) in segregating F1 progeny.
Developing P3 ‘reference sequence’ (i)
Developing P4 ‘reference sequence’ (i)
Identifying P4-specific heterozygous SNPs (ii)
Identifying P3-specific heterozygous SNPs (ii)
Calculating SNP-index and ΔSNP-index values by aligning sequence reads obtained for bulked-samples to P3 ‘reference sequence’ (iii)
Calculating SNP-index and ΔSNP-index values by aligning sequence reads obtained for bulked-samples to P4 ‘reference sequence’ (iii)
Plotting SNP-index and ΔSNP-index graphs (iii)
Plotting SNP-index and ΔSNP-index graphs (iii)
Identifying the candidate genomic region associated with the phenotype of interest and Inferring the genotype of parental lines, P3 and P4, from the ΔSNP-index pattern of the graphs generated by QTL-seq analysis.
In QTL-seq analysis using segregating the F1 progeny, we focused only on those parental line-specific heterozygous positions that segregated in 1 : 1 (homozygous : heterozygous) ratio in the F1 progeny. A schematic illustration of the QTL-seq analysis applied in this study is presented in Figure S17b, and the details of all the steps are described in Figure S17c.
Figure S17. (Continued…)

## Slide 23
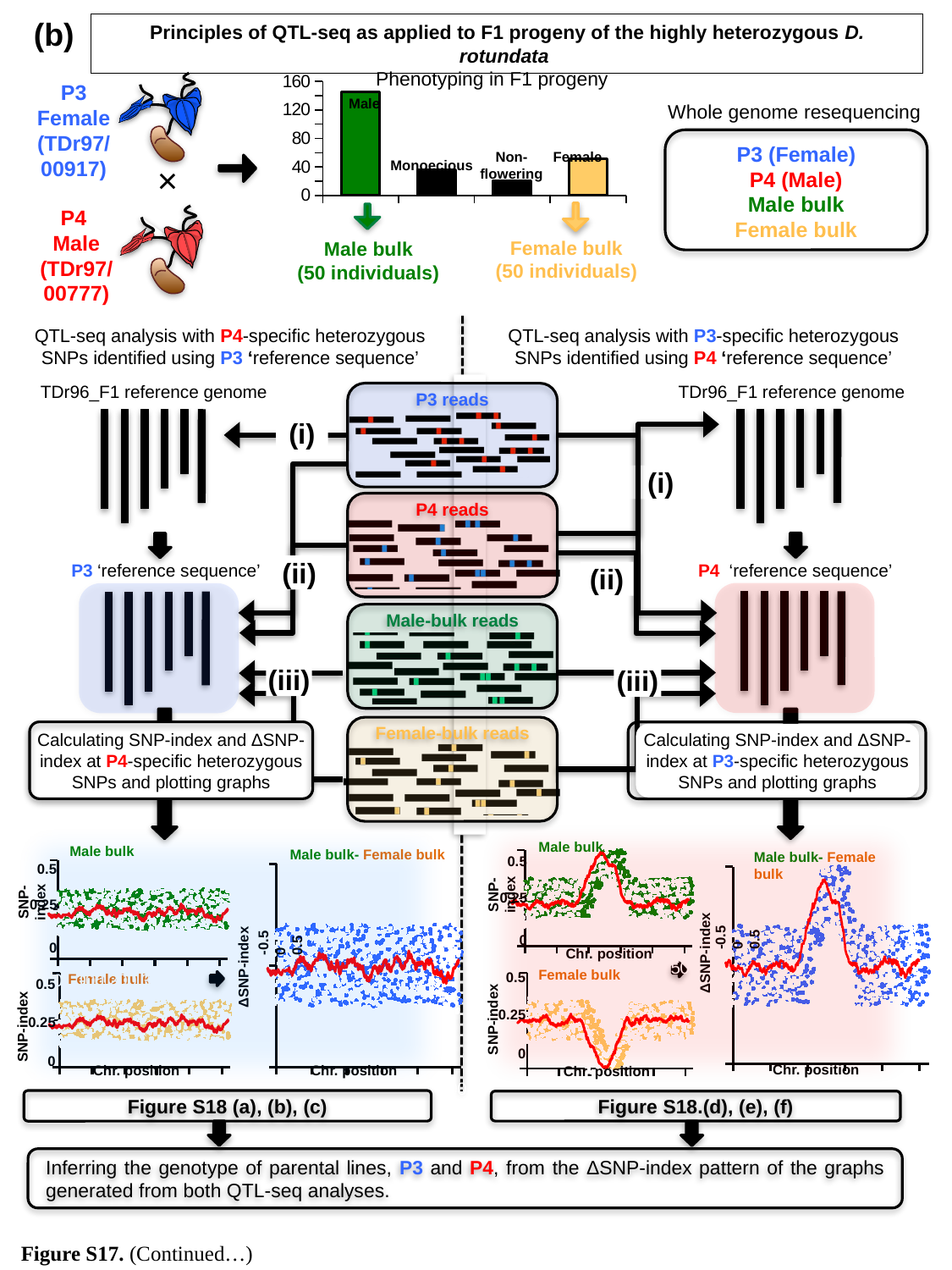

(b)
Principles of QTL-seq as applied to F1 progeny of the highly heterozygous D. rotundata
Phenotyping in F1 progeny
### Chart
| Category | |
|---|---|
| Male | 145.0 |
| Monoecious | 36.0 |
| Non-flowering | 21.0 |
| Female | 52.0 |P3
Female
(TDr97/00917)
Male
Whole genome resequencing
P3 (Female)
P4 (Male)
Male bulk
Female bulk
Non-flowering
Female
Monoecious
×
P4
Male
(TDr97/
00777)
Female bulk
(50 individuals)
Male bulk
(50 individuals)
QTL-seq analysis with P4-specific heterozygous SNPs identified using P3 ‘reference sequence’
QTL-seq analysis with P3-specific heterozygous SNPs identified using P4 ‘reference sequence’
TDr96_F1 reference genome
TDr96_F1 reference genome
P3 reads
(i)
(i)
P4 reads
P4 ‘reference sequence’
P3 ‘reference sequence’
(ii)
(ii)
Male-bulk reads
(iii)
(iii)
Female-bulk reads
Calculating SNP-index and ΔSNP-index at P4-specific heterozygous SNPs and plotting graphs
Calculating SNP-index and ΔSNP-index at P3-specific heterozygous SNPs and plotting graphs
Male bulk
Male bulk
### Chart
| Category | |
|---|---|
### Chart
| Category | |
|---|---|Male bulk- Female bulk
### Chart
| Category | |
|---|---|
### Chart
| Category | |
|---|---|
### Chart
| Category | |
|---|---|
### Chart
| Category | |
|---|---|Male bulk- Female bulk
0.5
### Chart
| Category | |
|---|---|
### Chart
| Category | |
|---|---|0.5
SNP-index
0.25
SNP-index
0.25
0
0
ΔSNP-index
Chr. position
	-0.5	0	0.5
	-0.5	0	0.5
ΔSNP-index
### Chart
| Category | |
|---|---|
### Chart
| Category | |
|---|---|Female bulk
### Chart
| Category | |
|---|---|
### Chart
| Category | |
|---|---|0.5
Female bulk
0.5
0.25
SNP-index
0.25
SNP-index
0
0
Chr. position
Chr. position
Chr. position
Chr. position
Figure S18 (a), (b), (c)
Figure S18.(d), (e), (f)
Inferring the genotype of parental lines, P3 and P4, from the ΔSNP-index pattern of the graphs generated from both QTL-seq analyses.
Figure S17. (Continued…)

## Slide 24
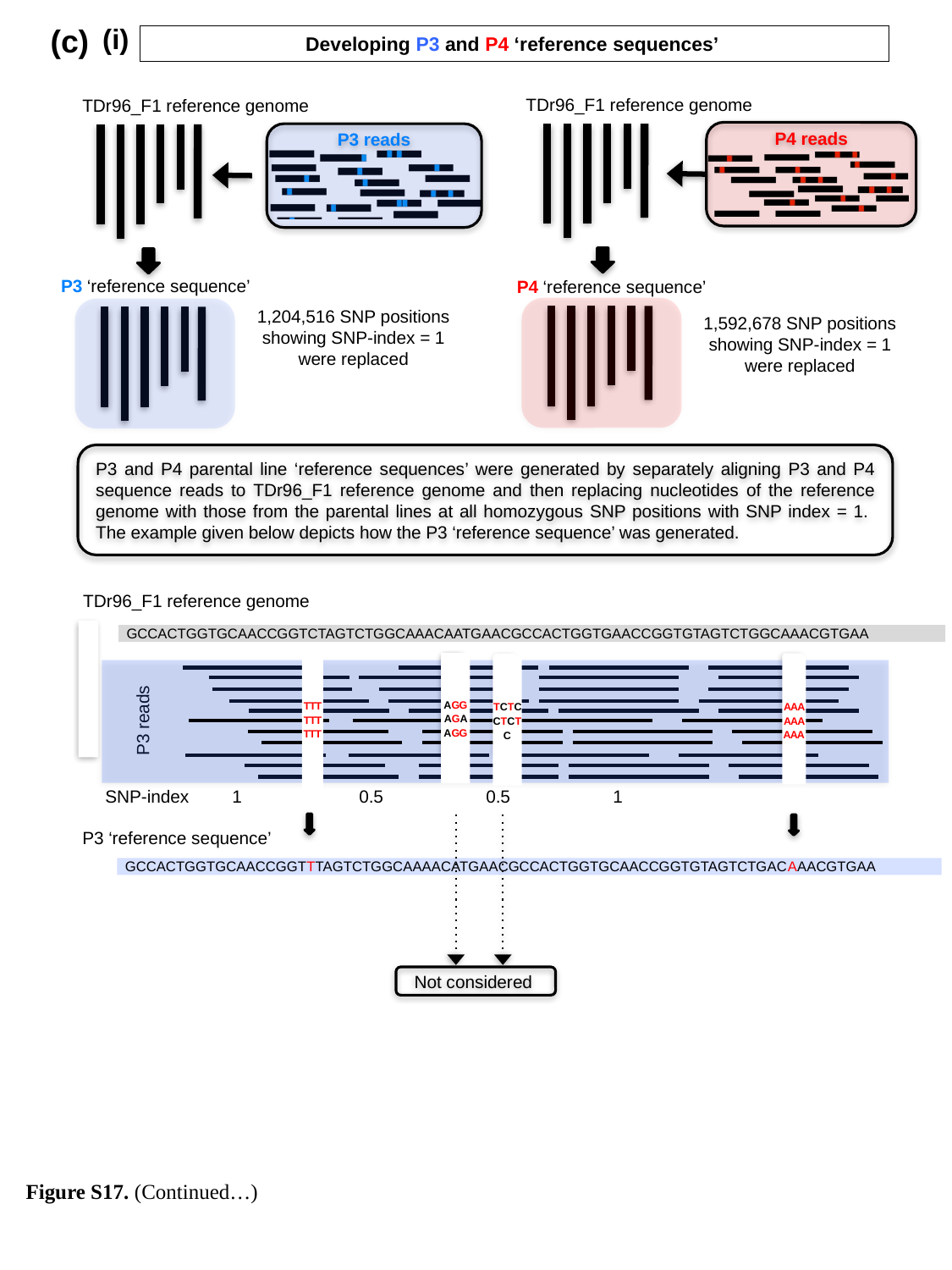

(c)
(i)
Developing P3 and P4 ‘reference sequences’
TDr96_F1 reference genome
TDr96_F1 reference genome
P4 reads
P3 reads
P3 ‘reference sequence’
P4 ‘reference sequence’
1,204,516 SNP positions showing SNP-index = 1 were replaced
1,592,678 SNP positions showing SNP-index = 1 were replaced
P3 and P4 parental line ‘reference sequences’ were generated by separately aligning P3 and P4 sequence reads to TDr96_F1 reference genome and then replacing nucleotides of the reference genome with those from the parental lines at all homozygous SNP positions with SNP index = 1. The example given below depicts how the P3 ‘reference sequence’ was generated.
TDr96_F1 reference genome
 GCCACTGGTGCAACCGGTCTAGTCTGGCAAACAATGAACGCCACTGGTGAACCGGTGTAGTCTGGCAAACGTGAA
TTTTTTTTT
AGGAGAAGG
TCTCCTCTC
AAAAAAAAA
P3 reads
SNP-index	1	0.5	0.5	1
P3 ‘reference sequence’
 GCCACTGGTGCAACCGGTTTAGTCTGGCAAAACATGAACGCCACTGGTGCAACCGGTGTAGTCTGACAAACGTGAA
Not considered
Figure S17. (Continued…)

## Slide 25
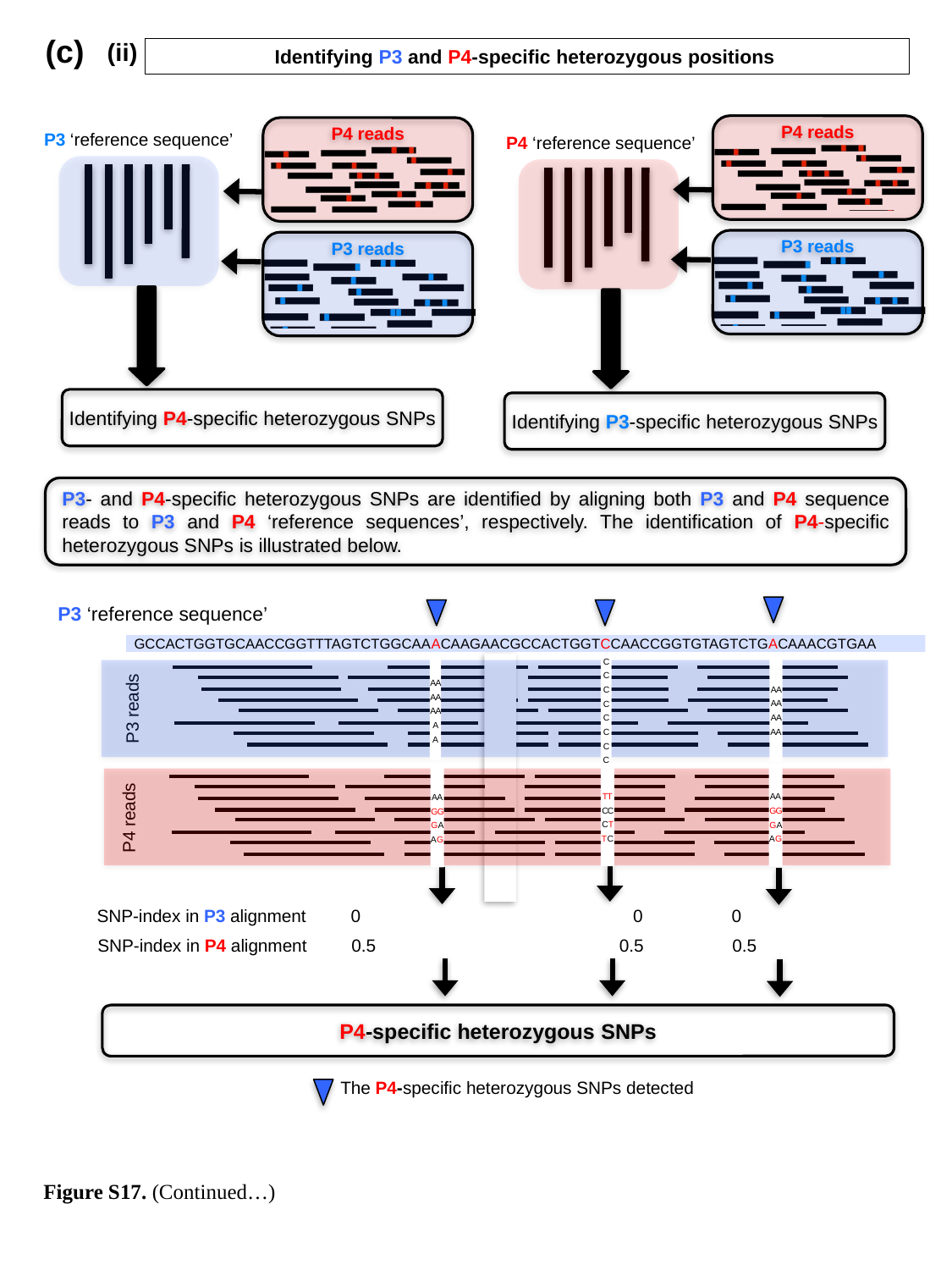

(c)
(ii)
Identifying P3 and P4-specific heterozygous positions
P4 reads
P4 reads
P3 ‘reference sequence’
P4 ‘reference sequence’
P3 reads
P3 reads
Identifying P4-specific heterozygous SNPs
Identifying P3-specific heterozygous SNPs
P3- and P4-specific heterozygous SNPs are identified by aligning both P3 and P4 sequence reads to P3 and P4 ‘reference sequences’, respectively. The identification of P4-specific heterozygous SNPs is illustrated below.
P3 ‘reference sequence’
 GCCACTGGTGCAACCGGTTTAGTCTGGCAAACAAGAACGCCACTGGTCCAACCGGTGTAGTCTGACAAACGTGAA
AAAAAAAA
CCCCCCCC
AAAAAAAA
P3 reads
TTCCCTTC
AAGGGAAG
AAGGGAAG
P4 reads
SNP-index in P3 alignment	0	 0	0
SNP-index in P4 alignment	0.5	 0.5	0.5
P4-specific heterozygous SNPs
The P4-specific heterozygous SNPs detected
Figure S17. (Continued…)

## Slide 26
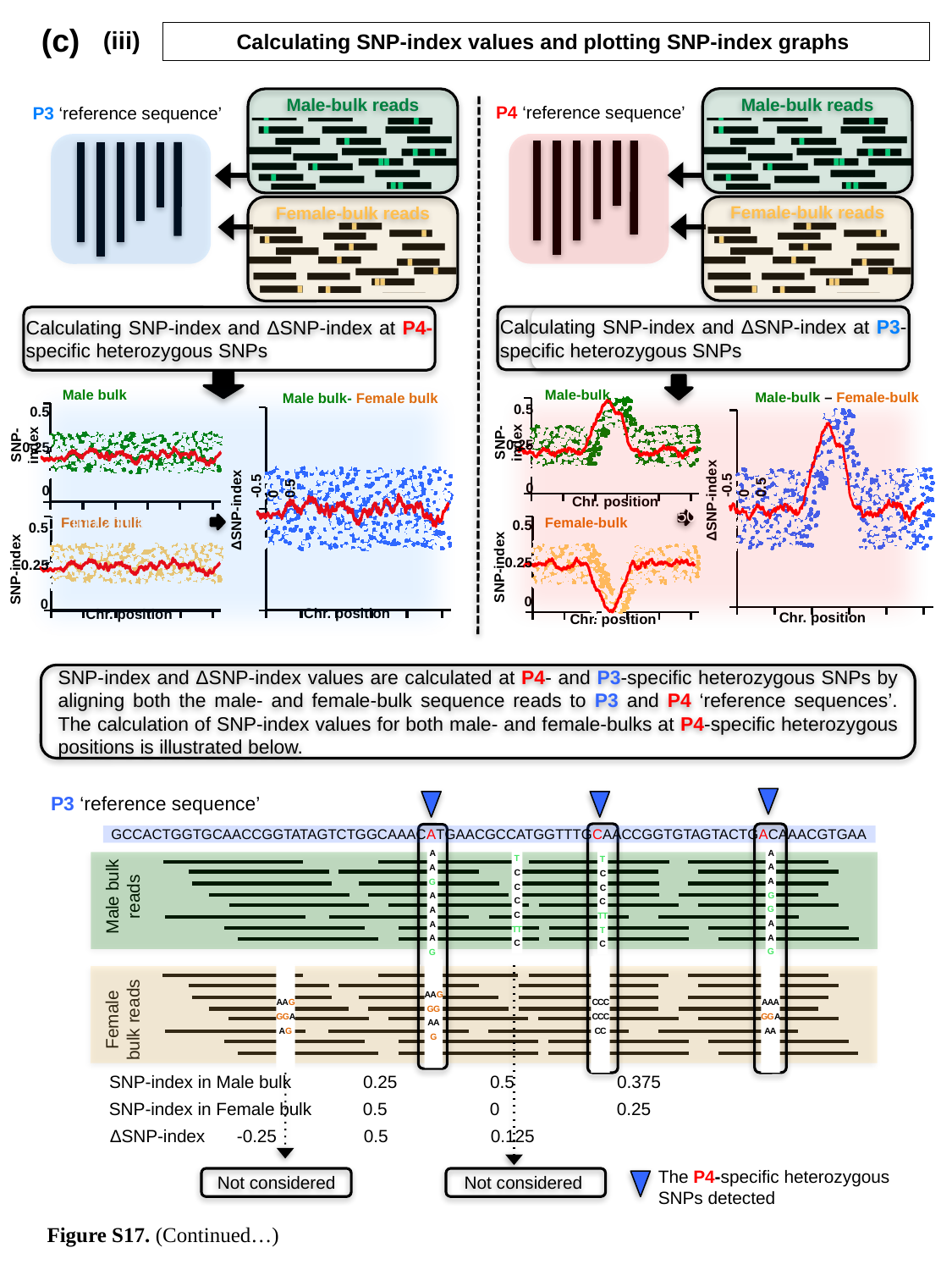

(c)
(iii)
Calculating SNP-index values and plotting SNP-index graphs
Male-bulk reads
Male-bulk reads
P4 ‘reference sequence’
P3 ‘reference sequence’
Female-bulk reads
Female-bulk reads
Calculating SNP-index and ΔSNP-index at P3-specific heterozygous SNPs
Calculating SNP-index and ΔSNP-index at P4-specific heterozygous SNPs
Male-bulk
Male bulk
Male-bulk – Female-bulk
Male bulk- Female bulk
### Chart
| Category | |
|---|---|
### Chart
| Category | |
|---|---|
### Chart
| Category | |
|---|---|
### Chart
| Category | |
|---|---|
### Chart
| Category | |
|---|---|
### Chart
| Category | |
|---|---|0.5
### Chart
| Category | |
|---|---|
### Chart
| Category | |
|---|---|0.5
SNP-index
0.25
SNP-index
0.25
0
0
ΔSNP-index
Chr. position
	-0.5	0	0.5
	-0.5	0	0.5
ΔSNP-index
### Chart
| Category | |
|---|---|
### Chart
| Category | |
|---|---|
### Chart
| Category | |
|---|---|
### Chart
| Category | |
|---|---|Female-bulk
Female bulk
0.5
0.5
0.25
0.25
SNP-index
SNP-index
0
0
Chr. position
Chr. position
Chr. position
Chr. position
SNP-index and ΔSNP-index values are calculated at P4- and P3-specific heterozygous SNPs by aligning both the male- and female-bulk sequence reads to P3 and P4 ‘reference sequences’. The calculation of SNP-index values for both male- and female-bulks at P4-specific heterozygous positions is illustrated below.
P3 ‘reference sequence’
 GCCACTGGTGCAACCGGTATAGTCTGGCAAACATGAACGCCATGGTTTGCAACCGGTGTAGTACTGACAAACGTGAA
TCCCCTTC
TCCCTTTC
AAAGGAAG
AAGAAAAG
Male bulk reads
AAGGGAAG
CCCCCCCC
AAAGGAAA
AAGGGAAG
Female bulk reads
SNP-index in Male bulk	0.25	0.5	0.375
SNP-index in Female bulk	0.5	0	0.25
ΔSNP-index	-0.25	0.5	0.125
The P4-specific heterozygous SNPs detected
Not considered
Not considered
Figure S17. (Continued…)

## Slide 27
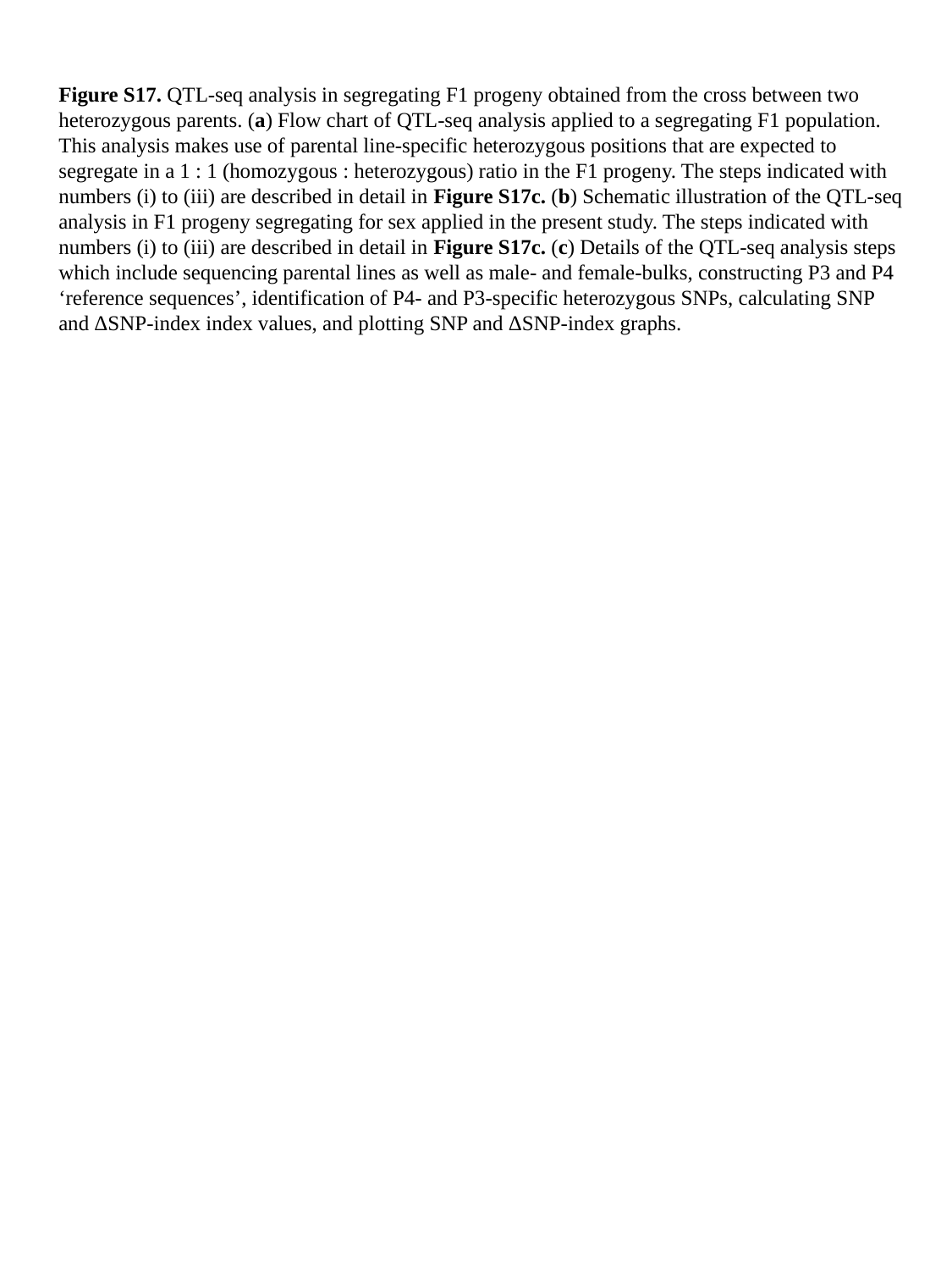

Figure S17. QTL-seq analysis in segregating F1 progeny obtained from the cross between two heterozygous parents. (a) Flow chart of QTL-seq analysis applied to a segregating F1 population. This analysis makes use of parental line-specific heterozygous positions that are expected to segregate in a 1 : 1 (homozygous : heterozygous) ratio in the F1 progeny. The steps indicated with numbers (i) to (iii) are described in detail in Figure S17c. (b) Schematic illustration of the QTL-seq analysis in F1 progeny segregating for sex applied in the present study. The steps indicated with numbers (i) to (iii) are described in detail in Figure S17c. (c) Details of the QTL-seq analysis steps which include sequencing parental lines as well as male- and female-bulks, constructing P3 and P4 ‘reference sequences’, identification of P4- and P3-specific heterozygous SNPs, calculating SNP and ΔSNP-index index values, and plotting SNP and ΔSNP-index graphs.

## Slide 28
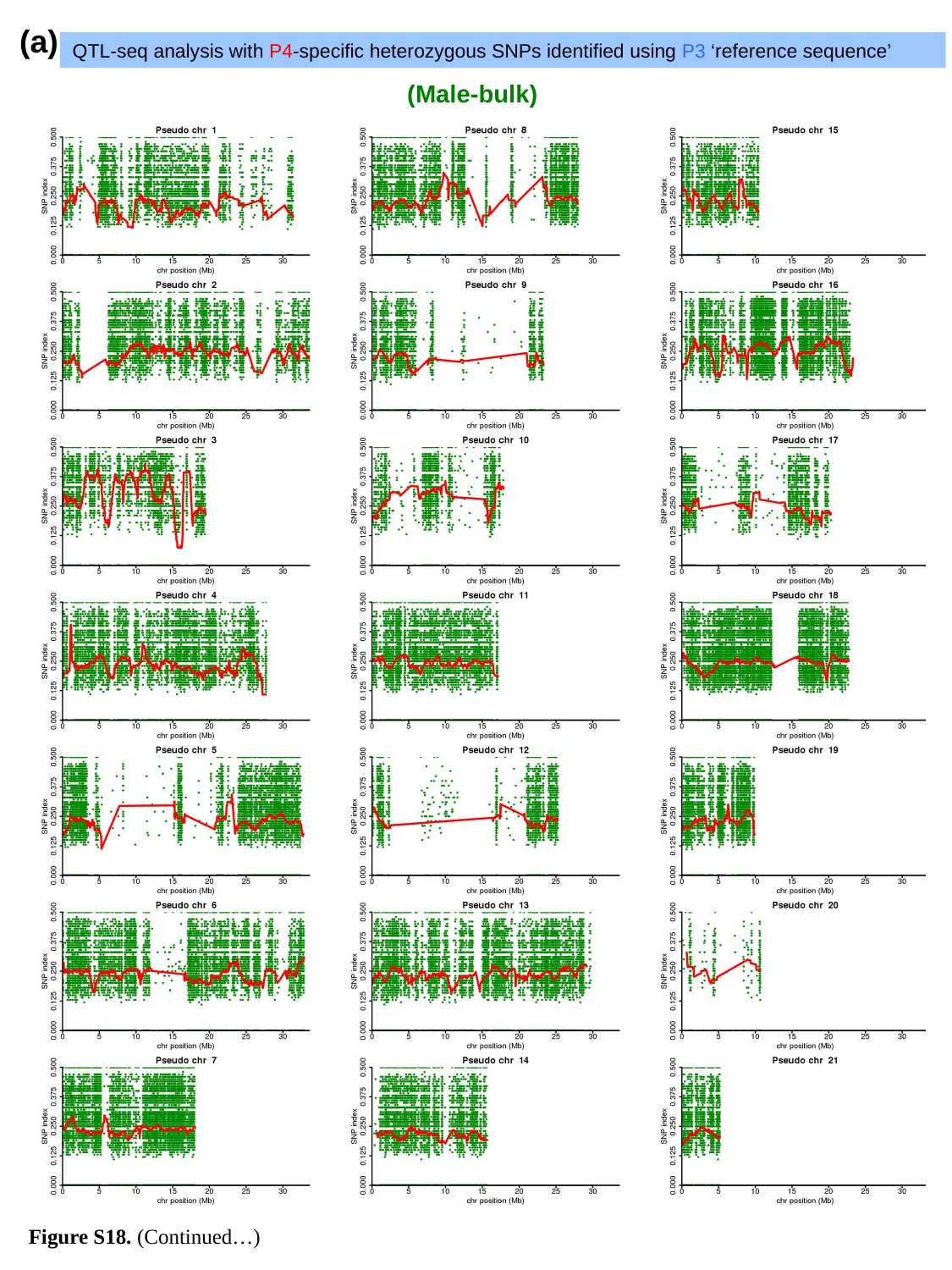

(a)
QTL-seq analysis with P4-specific heterozygous SNPs identified using P3 ‘reference sequence’
(Male-bulk)
Figure S18. (Continued…)

## Slide 29
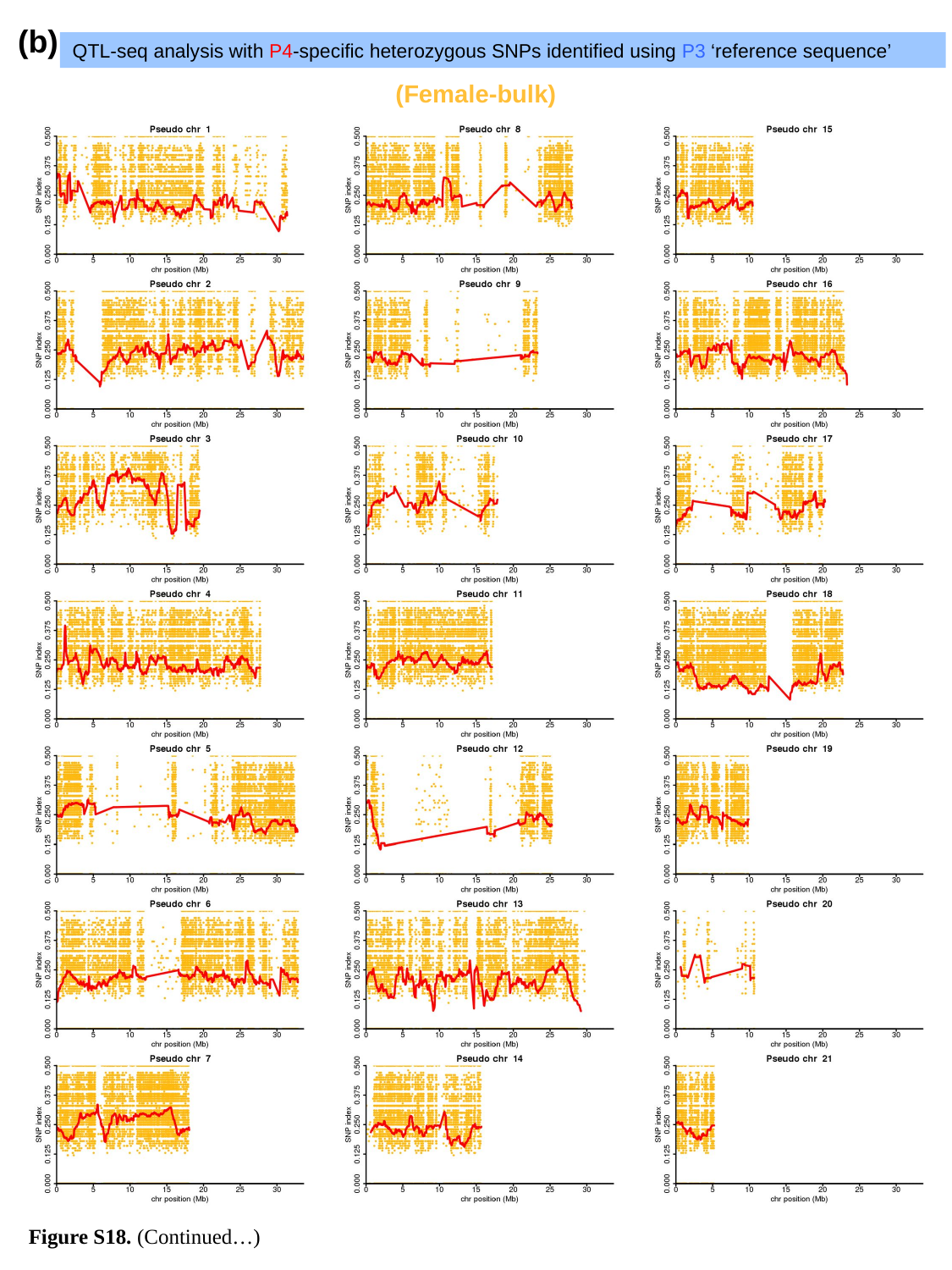

(b)
QTL-seq analysis with P4-specific heterozygous SNPs identified using P3 ‘reference sequence’
(Female-bulk)
Figure S18. (Continued…)

## Slide 30
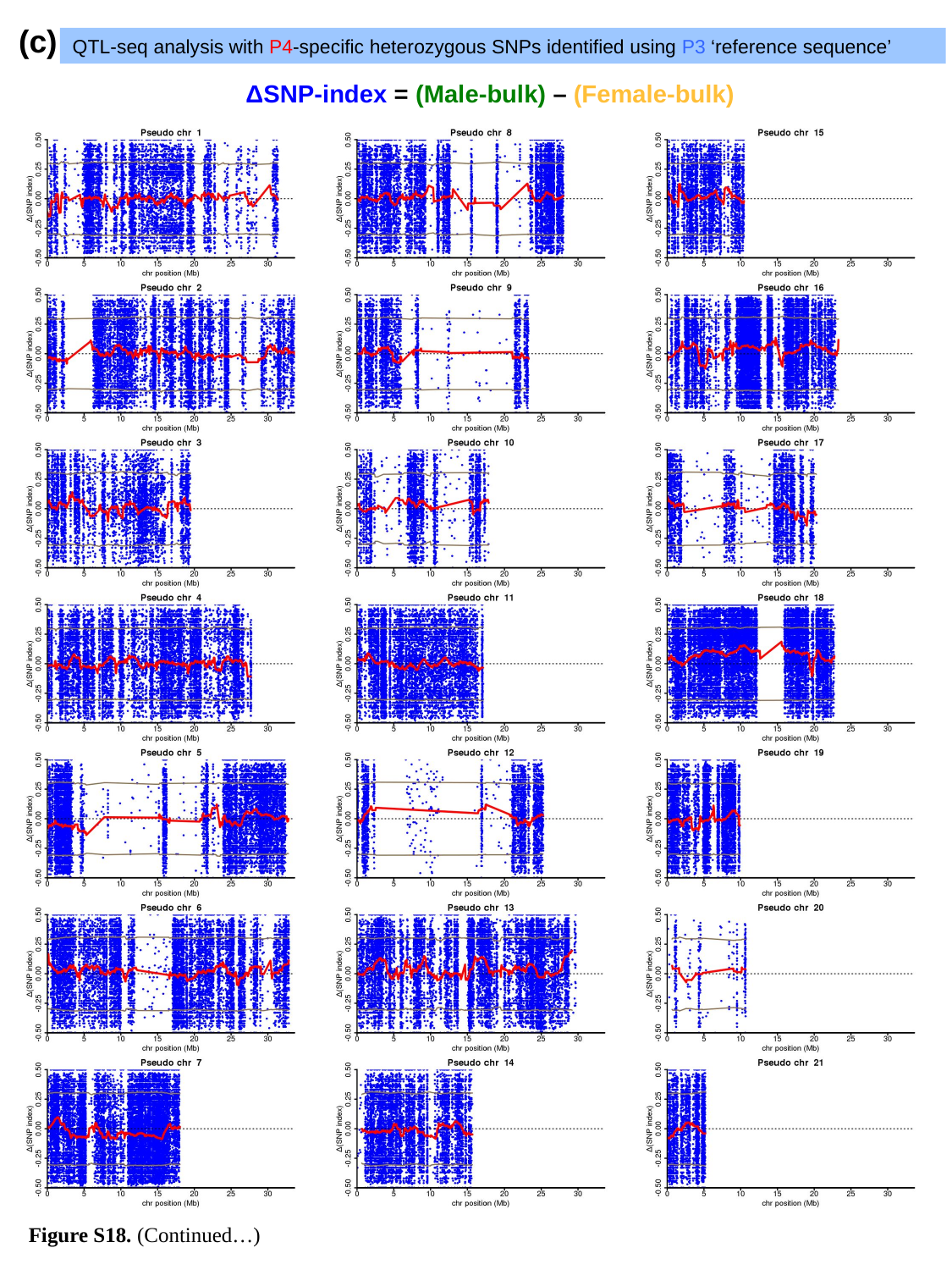

(c)
QTL-seq analysis with P4-specific heterozygous SNPs identified using P3 ‘reference sequence’
ΔSNP-index = (Male-bulk) – (Female-bulk)
Figure S18. (Continued…)

## Slide 31
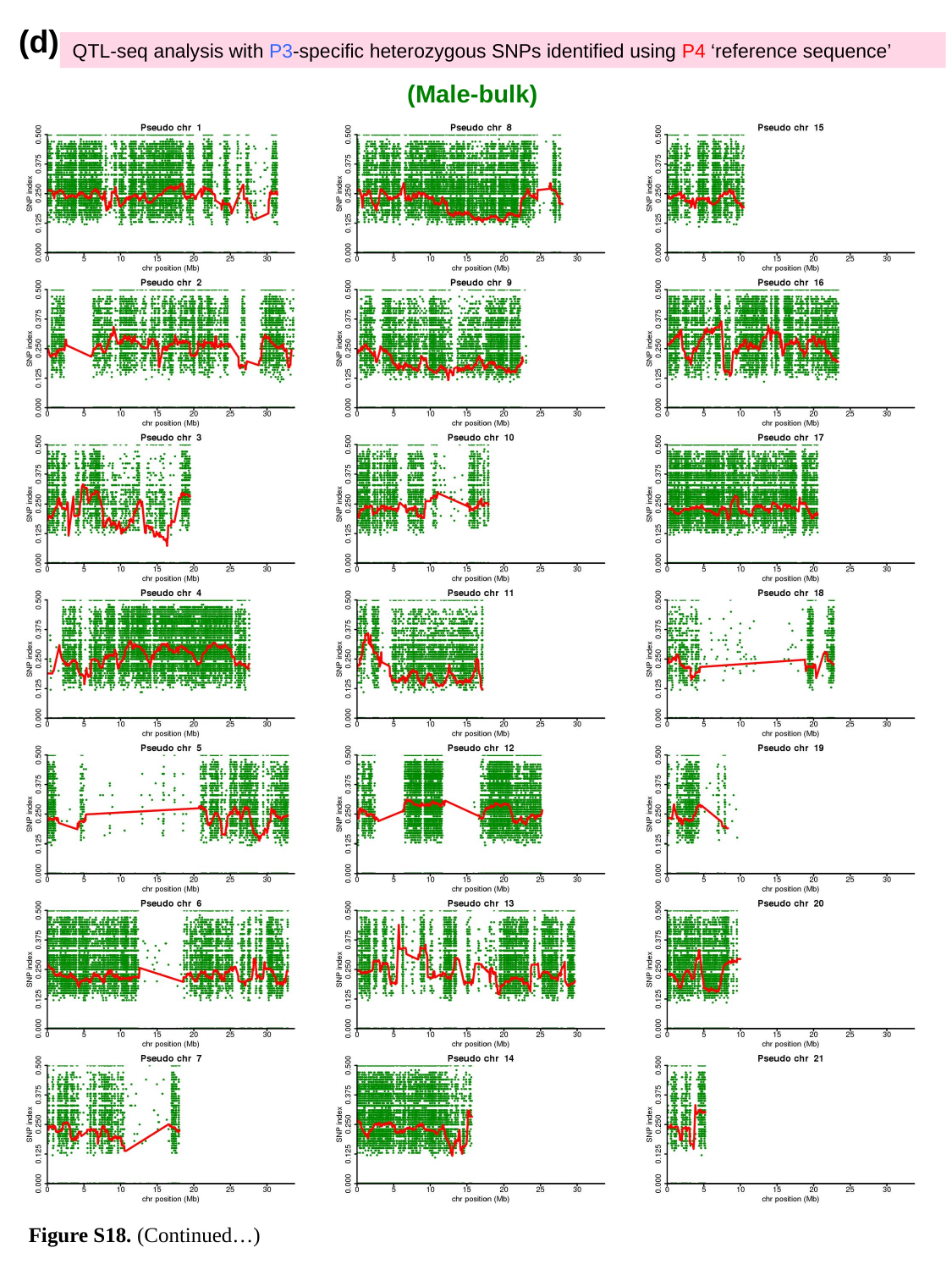

(d)
QTL-seq analysis with P3-specific heterozygous SNPs identified using P4 ‘reference sequence’
(Male-bulk)
Figure S18. (Continued…)

## Slide 32
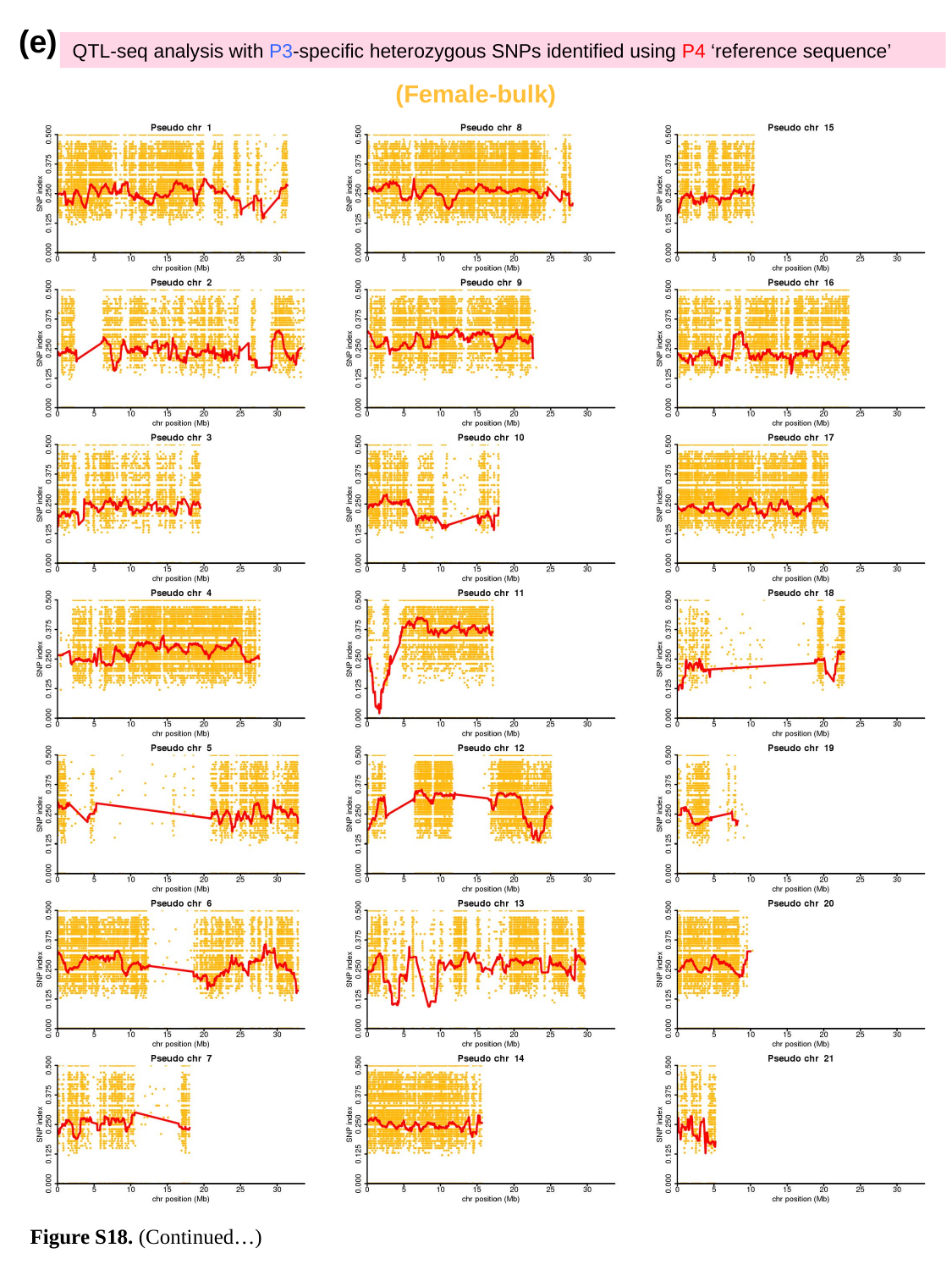

(e)
QTL-seq analysis with P3-specific heterozygous SNPs identified using P4 ‘reference sequence’
(Female-bulk)
Figure S18. (Continued…)

## Slide 33
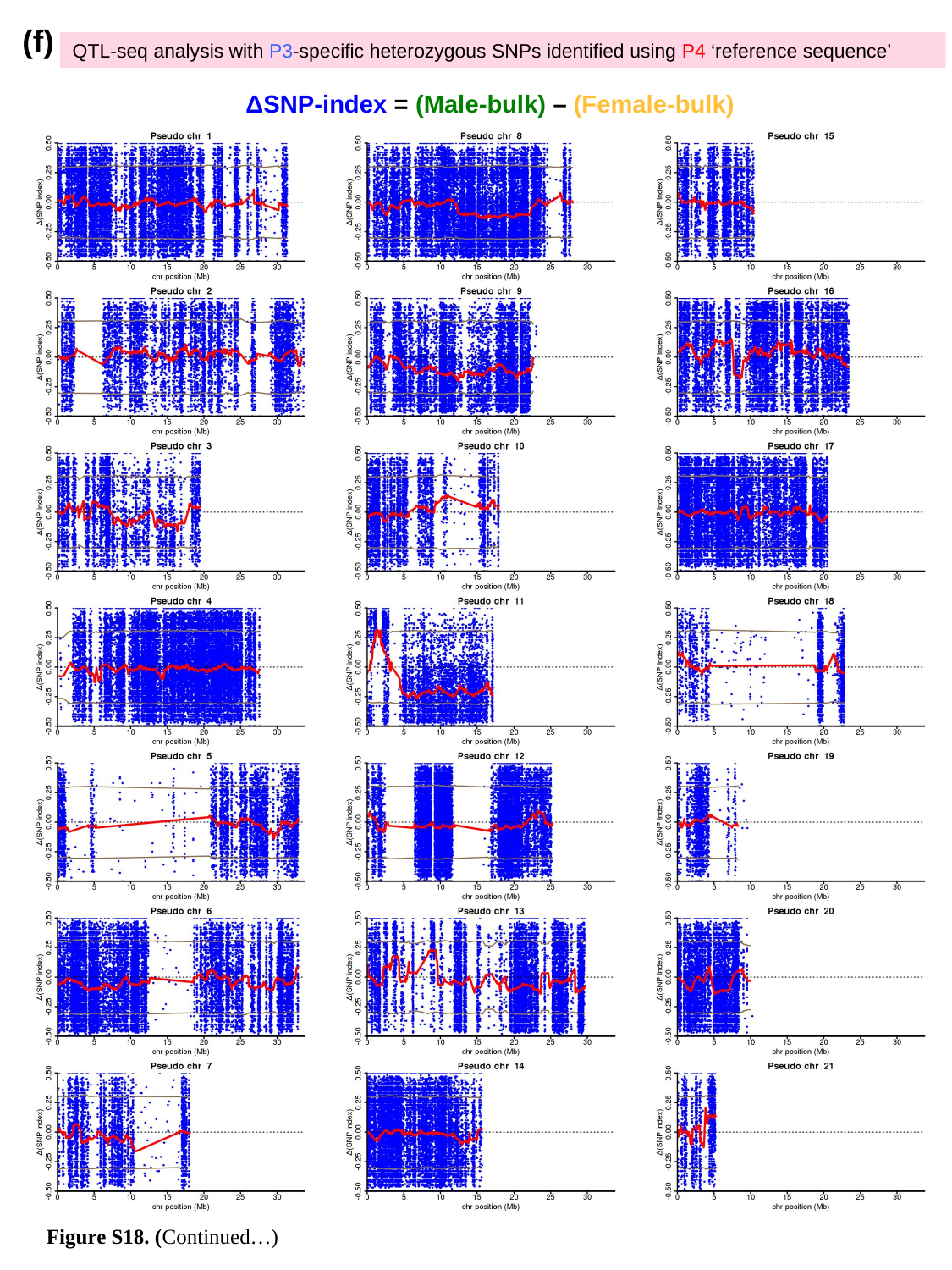

(f)
QTL-seq analysis with P3-specific heterozygous SNPs identified using P4 ‘reference sequence’
ΔSNP-index = (Male-bulk) – (Female-bulk)
Figure S18. (Continued…)

## Slide 34
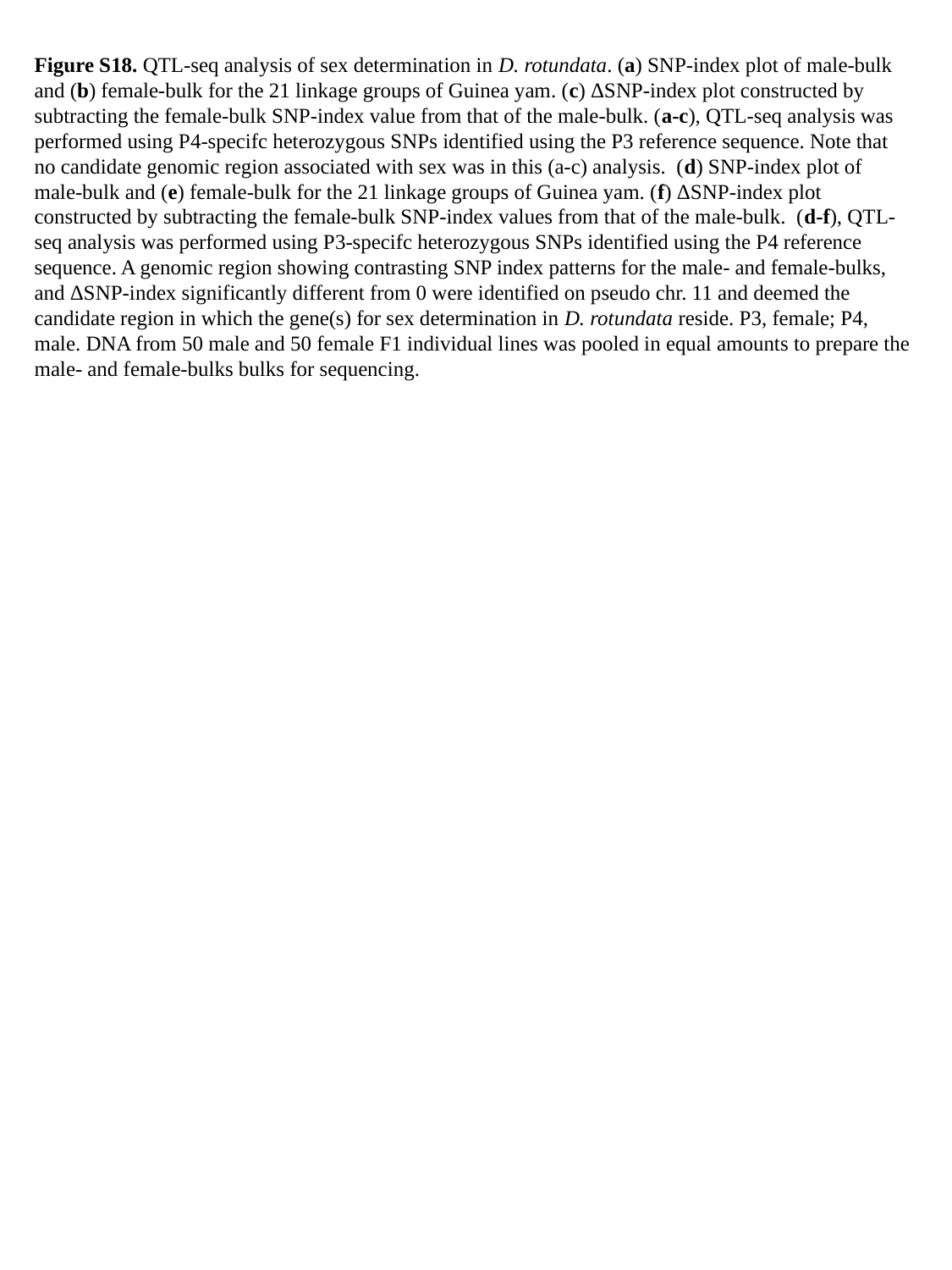

Figure S18. QTL-seq analysis of sex determination in D. rotundata. (a) SNP-index plot of male-bulk and (b) female-bulk for the 21 linkage groups of Guinea yam. (c) ΔSNP-index plot constructed by subtracting the female-bulk SNP-index value from that of the male-bulk. (a-c), QTL-seq analysis was performed using P4-specifc heterozygous SNPs identified using the P3 reference sequence. Note that no candidate genomic region associated with sex was in this (a-c) analysis. (d) SNP-index plot of male-bulk and (e) female-bulk for the 21 linkage groups of Guinea yam. (f) ΔSNP-index plot constructed by subtracting the female-bulk SNP-index values from that of the male-bulk. (d-f), QTL-seq analysis was performed using P3-specifc heterozygous SNPs identified using the P4 reference sequence. A genomic region showing contrasting SNP index patterns for the male- and female-bulks, and ΔSNP-index significantly different from 0 were identified on pseudo chr. 11 and deemed the candidate region in which the gene(s) for sex determination in D. rotundata reside. P3, female; P4, male. DNA from 50 male and 50 female F1 individual lines was pooled in equal amounts to prepare the male- and female-bulks bulks for sequencing.

## Slide 35
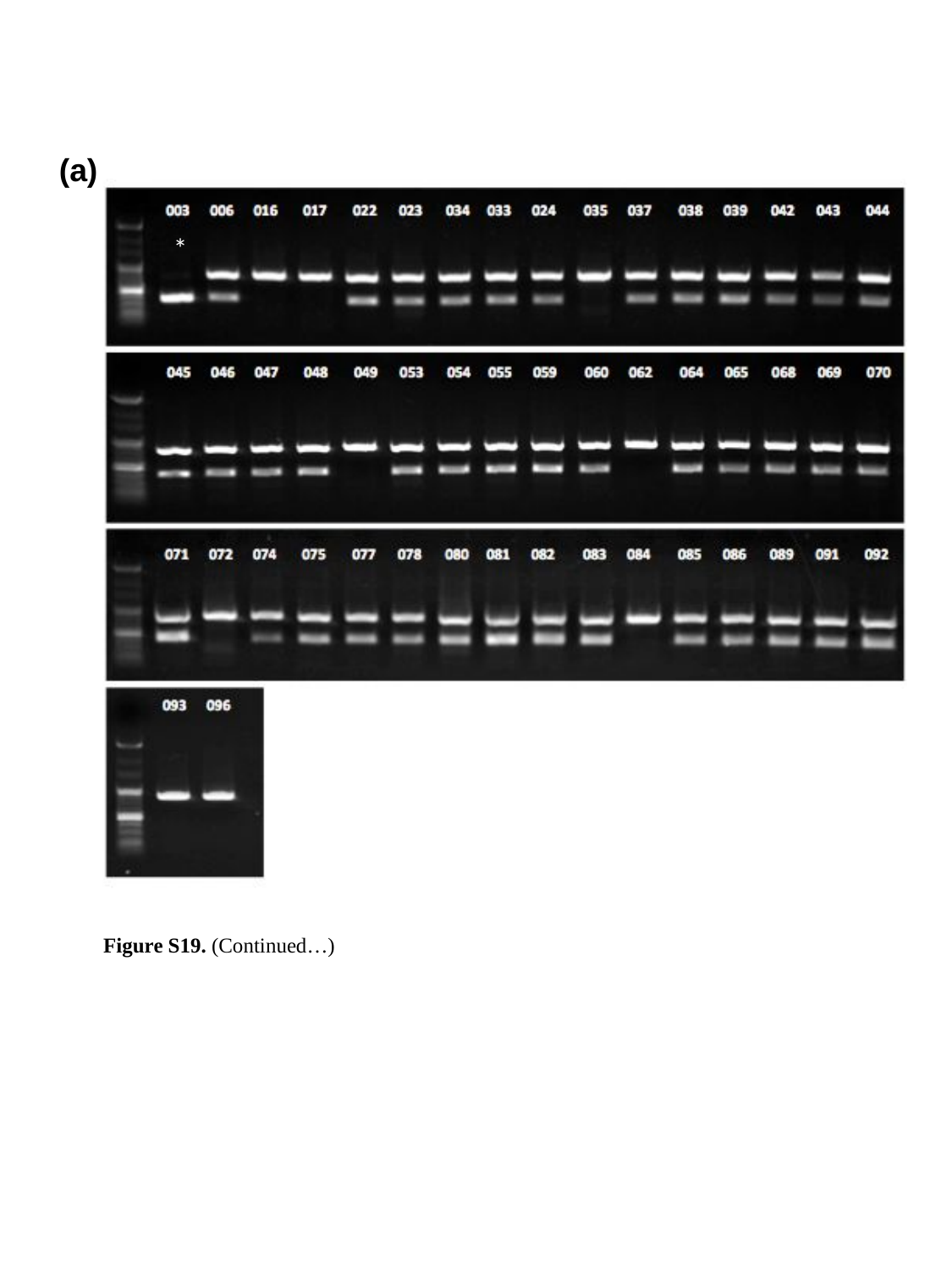

(a)
*
Figure S19. (Continued…)

## Slide 36
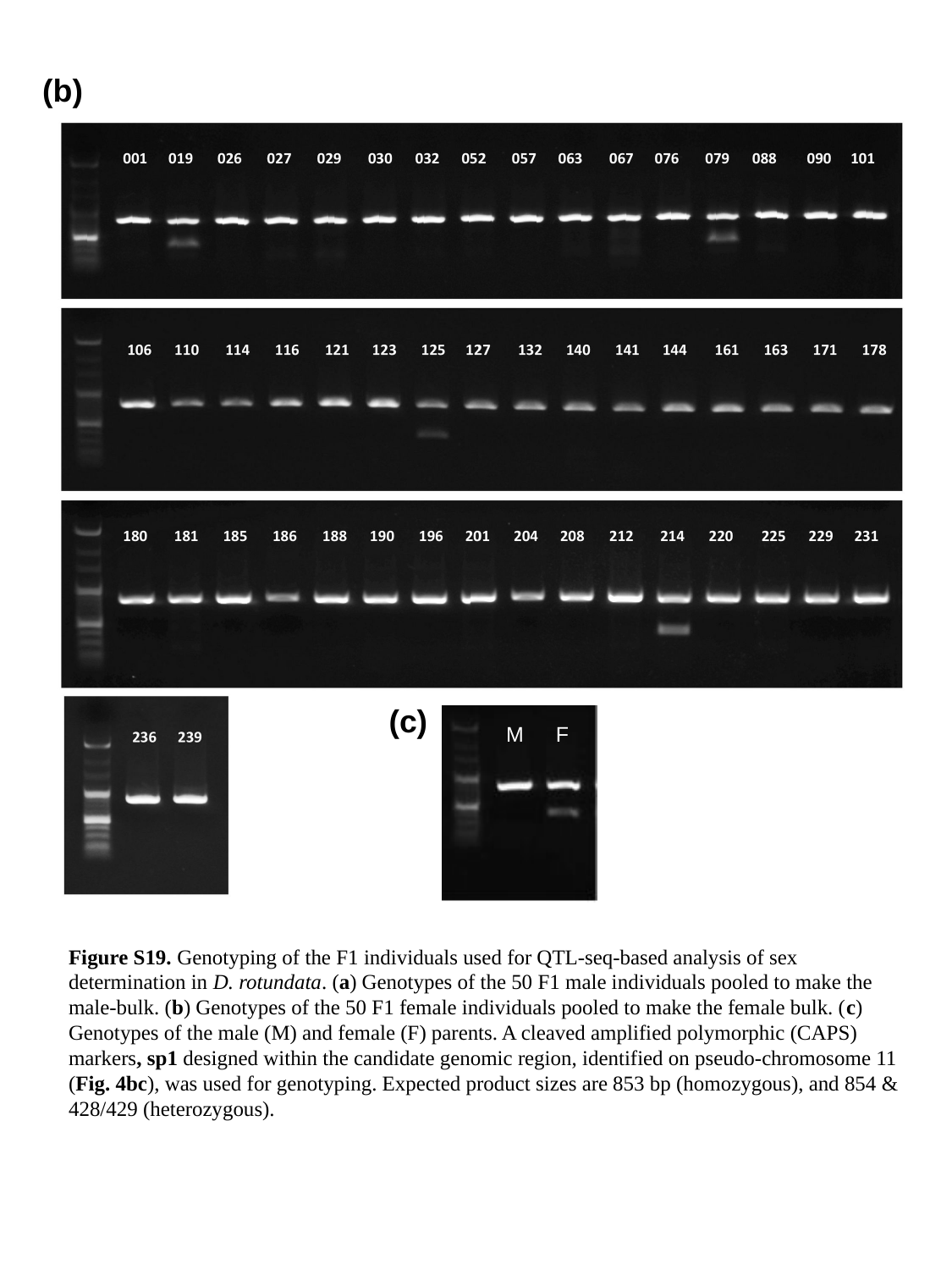

(b)
(c)
M
F
Figure S19. Genotyping of the F1 individuals used for QTL-seq-based analysis of sex determination in D. rotundata. (a) Genotypes of the 50 F1 male individuals pooled to make the male-bulk. (b) Genotypes of the 50 F1 female individuals pooled to make the female bulk. (c) Genotypes of the male (M) and female (F) parents. A cleaved amplified polymorphic (CAPS) markers, sp1 designed within the candidate genomic region, identified on pseudo-chromosome 11 (Fig. 4bc), was used for genotyping. Expected product sizes are 853 bp (homozygous), and 854 & 428/429 (heterozygous).

## Slide 37
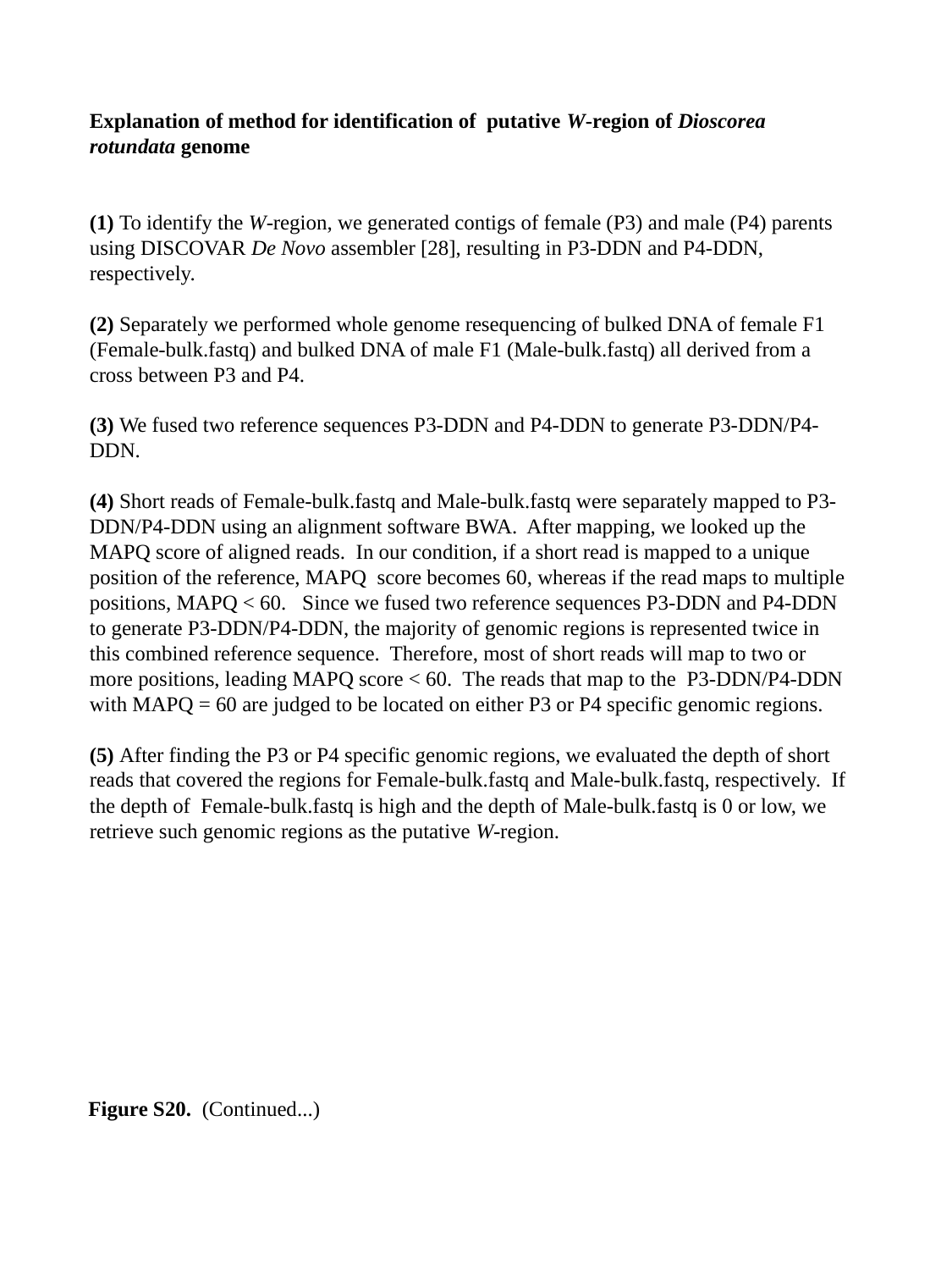

Explanation of method for identification of putative W-region of Dioscorea rotundata genome
(1) To identify the W-region, we generated contigs of female (P3) and male (P4) parents
using DISCOVAR De Novo assembler [28], resulting in P3-DDN and P4-DDN, respectively.
(2) Separately we performed whole genome resequencing of bulked DNA of female F1
(Female-bulk.fastq) and bulked DNA of male F1 (Male-bulk.fastq) all derived from a cross between P3 and P4.
(3) We fused two reference sequences P3-DDN and P4-DDN to generate P3-DDN/P4-DDN.
(4) Short reads of Female-bulk.fastq and Male-bulk.fastq were separately mapped to P3-DDN/P4-DDN using an alignment software BWA. After mapping, we looked up the MAPQ score of aligned reads. In our condition, if a short read is mapped to a unique position of the reference, MAPQ score becomes 60, whereas if the read maps to multiple positions, MAPQ < 60. Since we fused two reference sequences P3-DDN and P4-DDN to generate P3-DDN/P4-DDN, the majority of genomic regions is represented twice in this combined reference sequence. Therefore, most of short reads will map to two or more positions, leading MAPQ score < 60. The reads that map to the P3-DDN/P4-DDN with MAPQ = 60 are judged to be located on either P3 or P4 specific genomic regions.
(5) After finding the P3 or P4 specific genomic regions, we evaluated the depth of short reads that covered the regions for Female-bulk.fastq and Male-bulk.fastq, respectively. If the depth of Female-bulk.fastq is high and the depth of Male-bulk.fastq is 0 or low, we retrieve such genomic regions as the putative W-region.
Figure S20. (Continued...)

## Slide 38
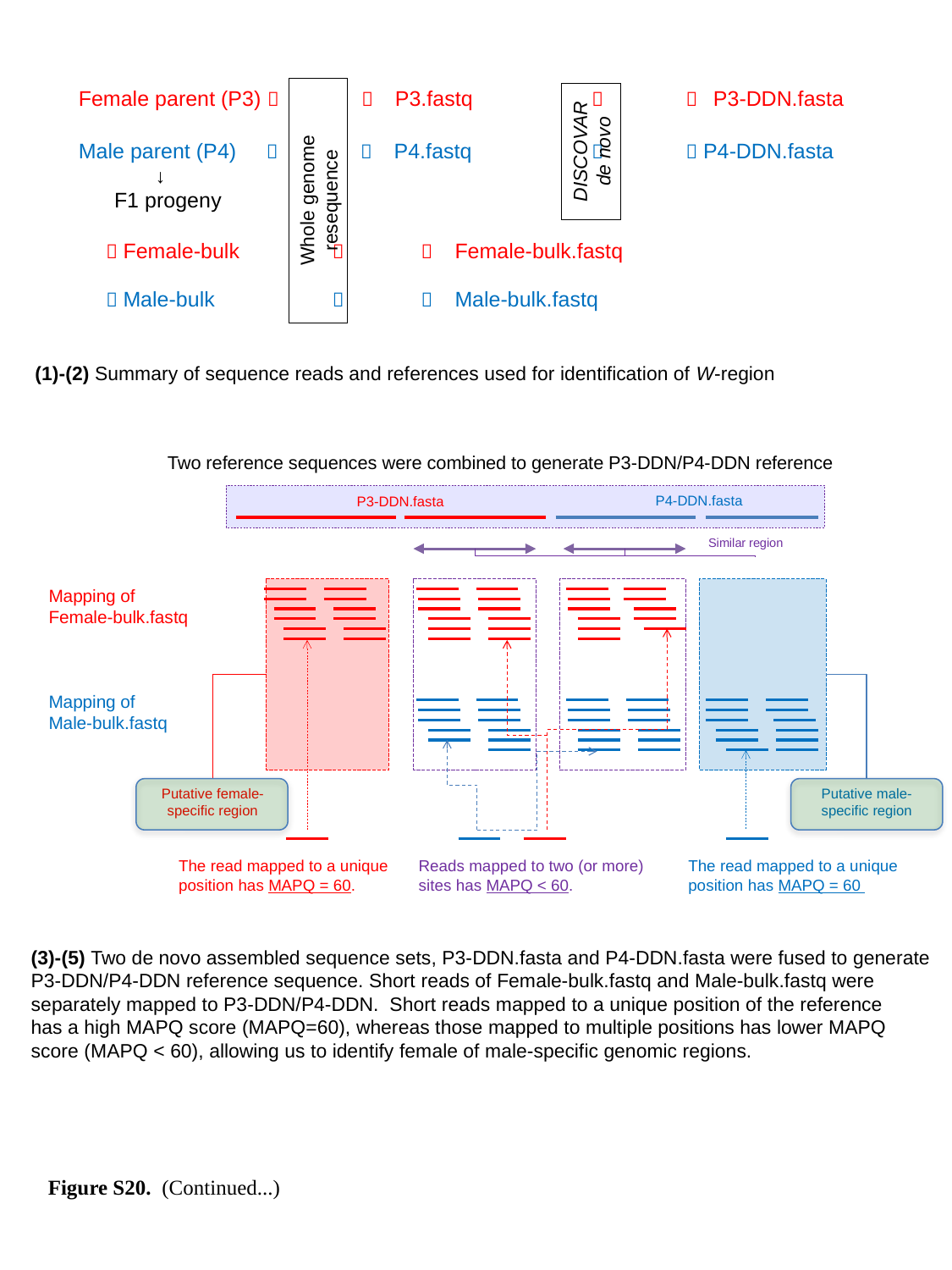

Female parent (P3)   P3.fastq	  	P3-DDN.fasta
Male parent (P4)   P4.fastq 	   P4-DDN.fasta
 ↓
 F1 progeny
  Female-bulk	  Female-bulk.fastq
  Male-bulk	  Male-bulk.fastq
DISCOVAR
de novo
Whole genome resequence
 (1)-(2) Summary of sequence reads and references used for identification of W-region
Two reference sequences were combined to generate P3-DDN/P4-DDN reference
P4-DDN.fasta
P3-DDN.fasta
Similar region
Mapping of
Female-bulk.fastq
Mapping of
Male-bulk.fastq
Putative female-specific region
Putative male-specific region
The read mapped to a unique position has MAPQ = 60.
Reads mapped to two (or more) sites has MAPQ < 60.
The read mapped to a unique position has MAPQ = 60
(3)-(5) Two de novo assembled sequence sets, P3-DDN.fasta and P4-DDN.fasta were fused to generate
P3-DDN/P4-DDN reference sequence. Short reads of Female-bulk.fastq and Male-bulk.fastq were
separately mapped to P3-DDN/P4-DDN. Short reads mapped to a unique position of the reference
has a high MAPQ score (MAPQ=60), whereas those mapped to multiple positions has lower MAPQ
score (MAPQ < 60), allowing us to identify female of male-specific genomic regions.
Figure S20. (Continued...)

## Slide 39
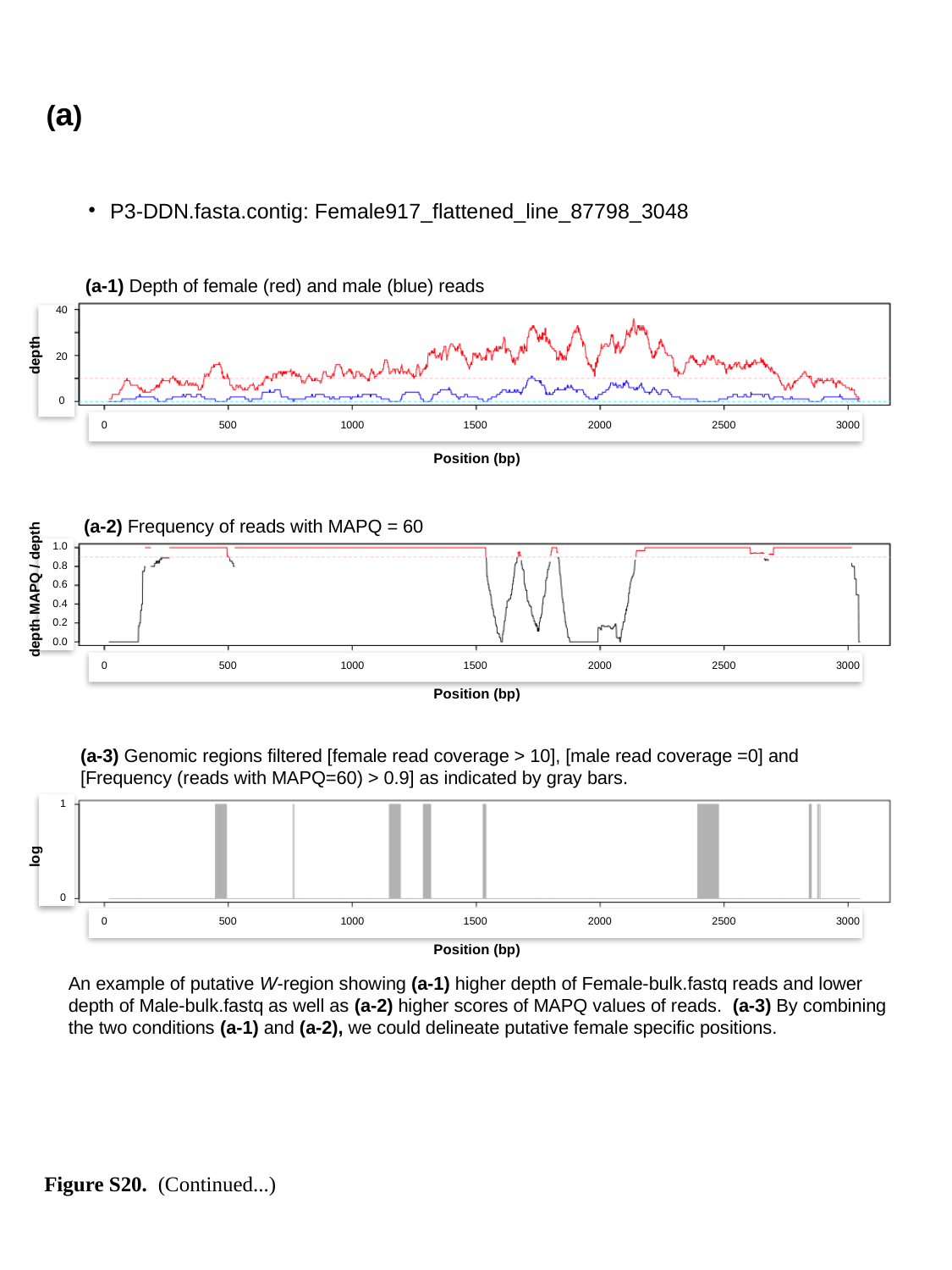

(a)
・P3-DDN.fasta.contig: Female917_flattened_line_87798_3048
(a-1) Depth of female (red) and male (blue) reads
40
20
0
depth
0
2000
2500
500
1000
1500
3000
Position (bp)
(a-2) Frequency of reads with MAPQ = 60
1.0
0.8
0.6
0.4
0.2
0.0
depth MAPQ / depth
0
2000
2500
500
1000
1500
3000
Position (bp)
(a-3) Genomic regions filtered [female read coverage > 10], [male read coverage =0] and [Frequency (reads with MAPQ=60) > 0.9] as indicated by gray bars.
1
0
log
0
2000
2500
500
1000
1500
3000
Position (bp)
An example of putative W-region showing (a-1) higher depth of Female-bulk.fastq reads and lower depth of Male-bulk.fastq as well as (a-2) higher scores of MAPQ values of reads. (a-3) By combining
the two conditions (a-1) and (a-2), we could delineate putative female specific positions.
Figure S20. (Continued...)

## Slide 40
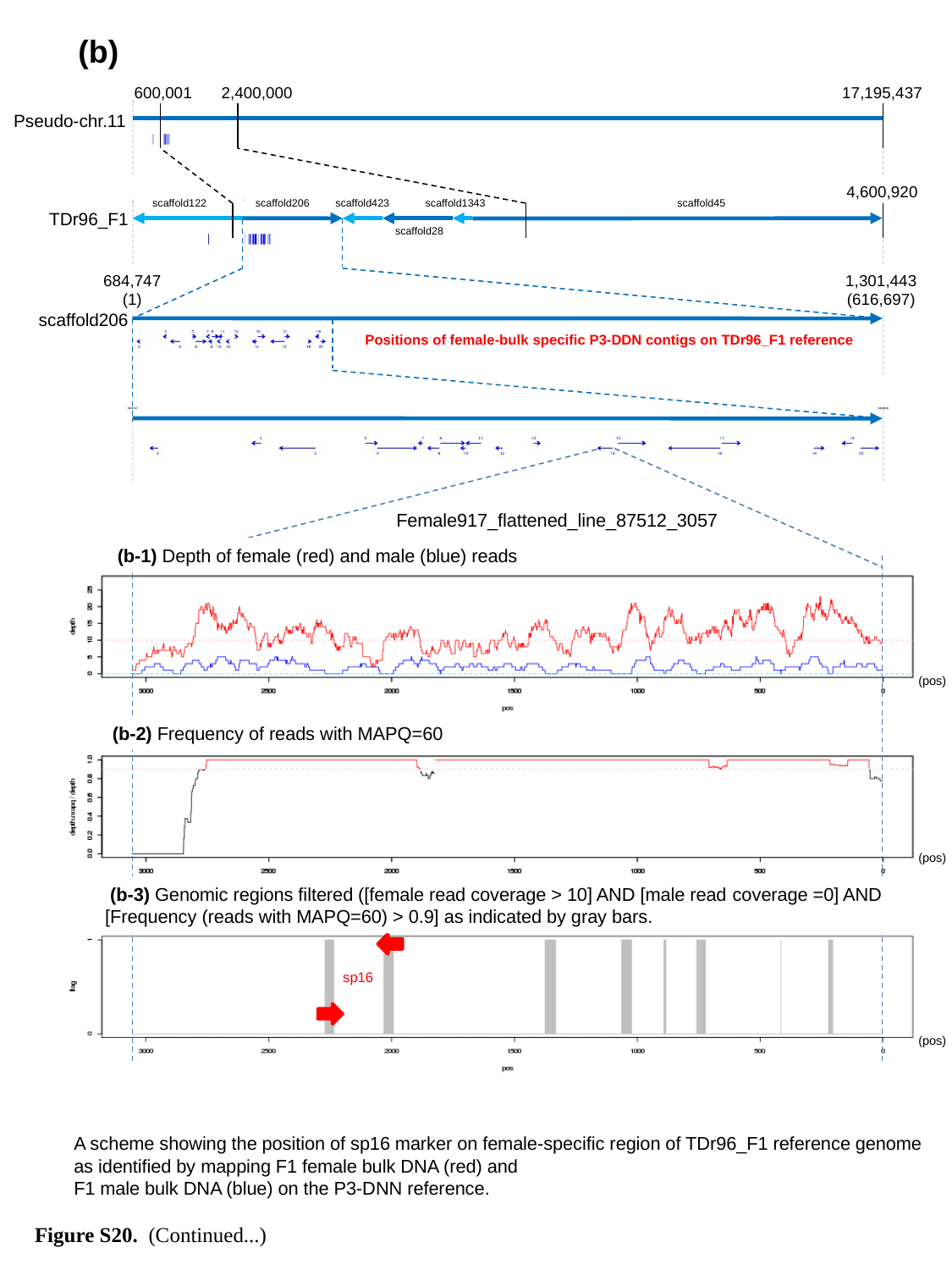

(b)
600,001
2,400,000
17,195,437
Pseudo-chr.11
4,600,920
scaffold122
scaffold206
scaffold423
scaffold1343
scaffold45
TDr96_F1
scaffold28
684,747
(1)
1,301,443
(616,697)
scaffold206
Positions of female-bulk specific P3-DDN contigs on TDr96_F1 reference
Female917_flattened_line_87512_3057
(b-1) Depth of female (red) and male (blue) reads
(pos)
(b-2) Frequency of reads with MAPQ=60
(pos)
 (b-3) Genomic regions filtered ([female read coverage > 10] AND [male read coverage =0] AND [Frequency (reads with MAPQ=60) > 0.9] as indicated by gray bars.
sp16
(pos)
A scheme showing the position of sp16 marker on female-specific region of TDr96_F1 reference genome as identified by mapping F1 female bulk DNA (red) and
F1 male bulk DNA (blue) on the P3-DNN reference.
Figure S20. (Continued...)

## Slide 41
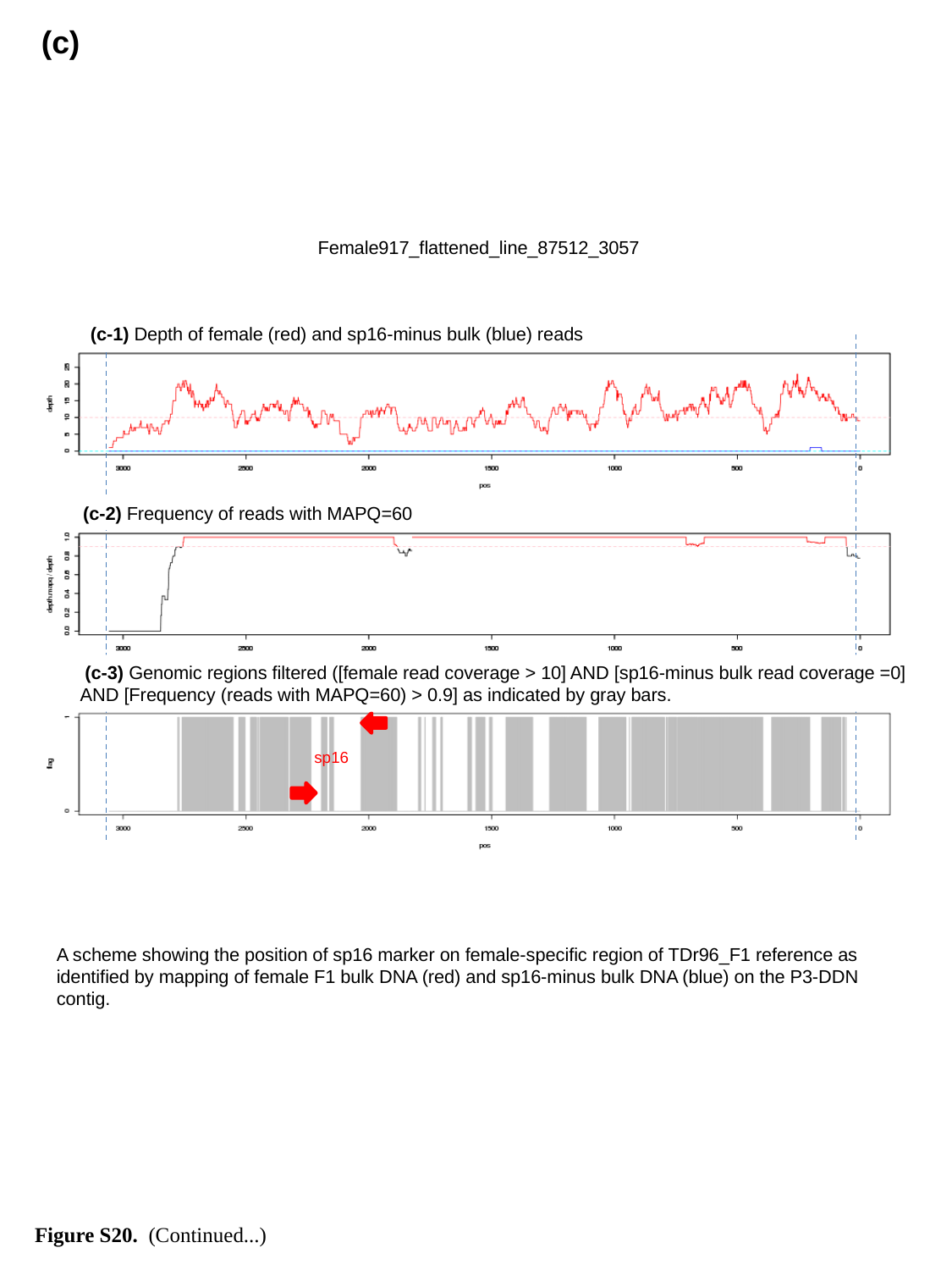

(c)
Female917_flattened_line_87512_3057
(c-1) Depth of female (red) and sp16-minus bulk (blue) reads
(c-2) Frequency of reads with MAPQ=60
 (c-3) Genomic regions filtered ([female read coverage > 10] AND [sp16-minus bulk read coverage =0] AND [Frequency (reads with MAPQ=60) > 0.9] as indicated by gray bars.
sp16
A scheme showing the position of sp16 marker on female-specific region of TDr96_F1 reference as identified by mapping of female F1 bulk DNA (red) and sp16-minus bulk DNA (blue) on the P3-DDN contig.
Figure S20. (Continued...)

## Slide 42
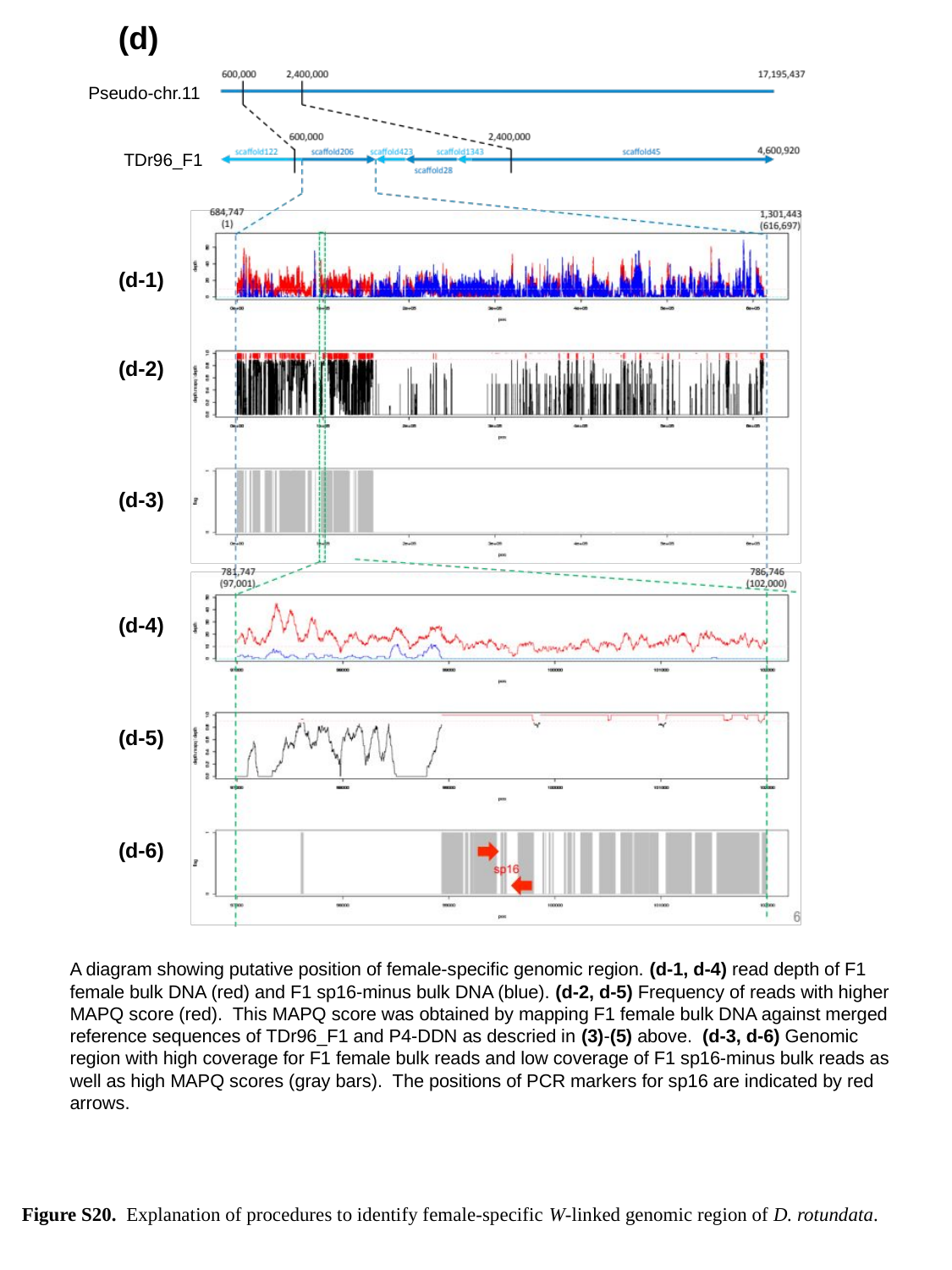

(d)
Pseudo-chr.11
TDr96_F1
(d-1)
(d-2)
(d-3)
(d-4)
(d-5)
(d-6)
A diagram showing putative position of female-specific genomic region. (d-1, d-4) read depth of F1 female bulk DNA (red) and F1 sp16-minus bulk DNA (blue). (d-2, d-5) Frequency of reads with higher MAPQ score (red). This MAPQ score was obtained by mapping F1 female bulk DNA against merged reference sequences of TDr96_F1 and P4-DDN as descried in (3)-(5) above. (d-3, d-6) Genomic region with high coverage for F1 female bulk reads and low coverage of F1 sp16-minus bulk reads as well as high MAPQ scores (gray bars). The positions of PCR markers for sp16 are indicated by red arrows.
Figure S20. Explanation of procedures to identify female-specific W-linked genomic region of D. rotundata.

## Slide 43
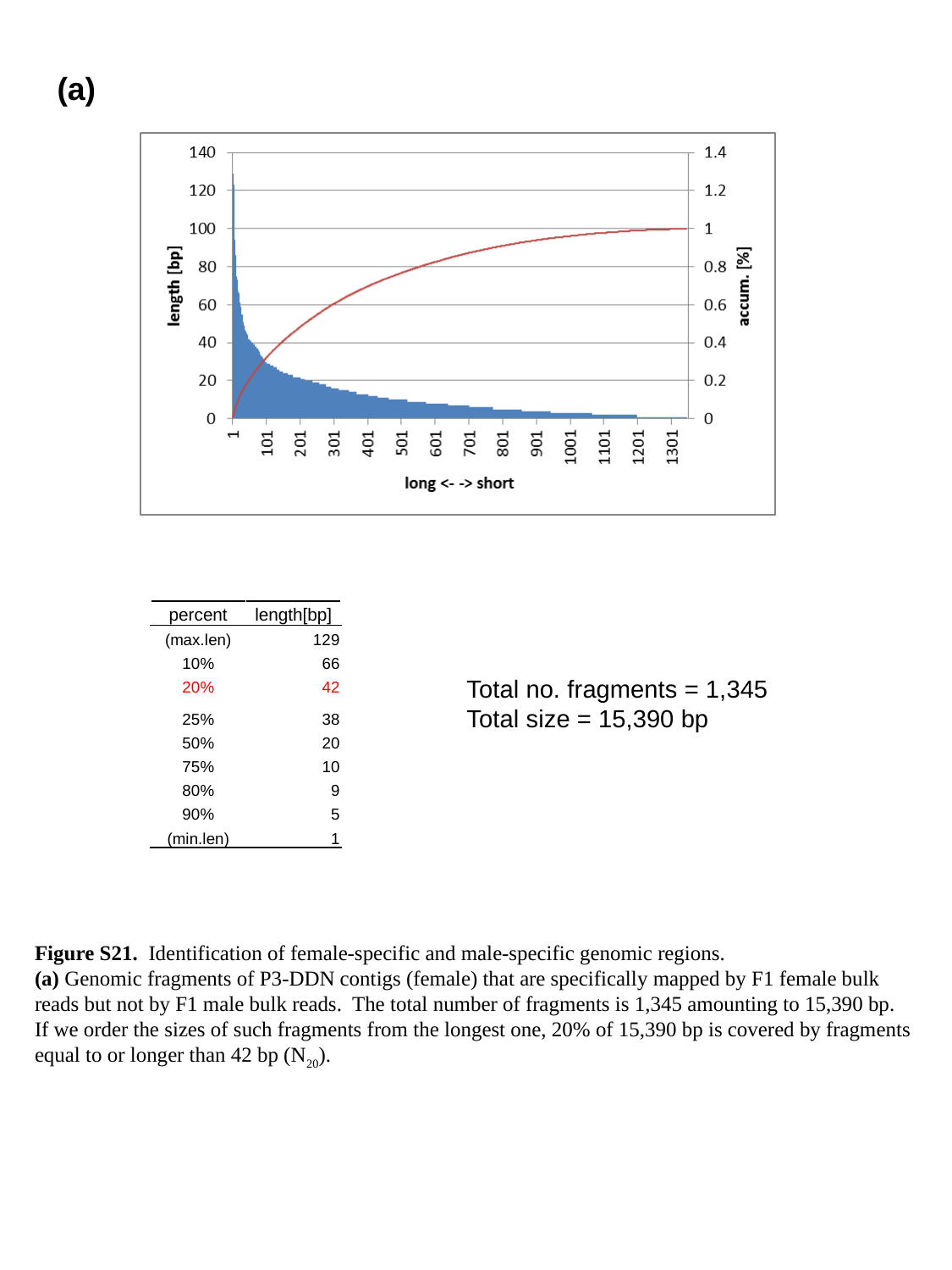

(a)
| percent | length[bp] |
| --- | --- |
| (max.len) | 129 |
| 10% | 66 |
| 20% | 42 |
| 25% | 38 |
| 50% | 20 |
| 75% | 10 |
| 80% | 9 |
| 90% | 5 |
| (min.len) | 1 |
Total no. fragments = 1,345
Total size = 15,390 bp
Figure S21. Identification of female-specific and male-specific genomic regions.
(a) Genomic fragments of P3-DDN contigs (female) that are specifically mapped by F1 female bulk reads but not by F1 male bulk reads. The total number of fragments is 1,345 amounting to 15,390 bp. If we order the sizes of such fragments from the longest one, 20% of 15,390 bp is covered by fragments equal to or longer than 42 bp (N20).

## Slide 44
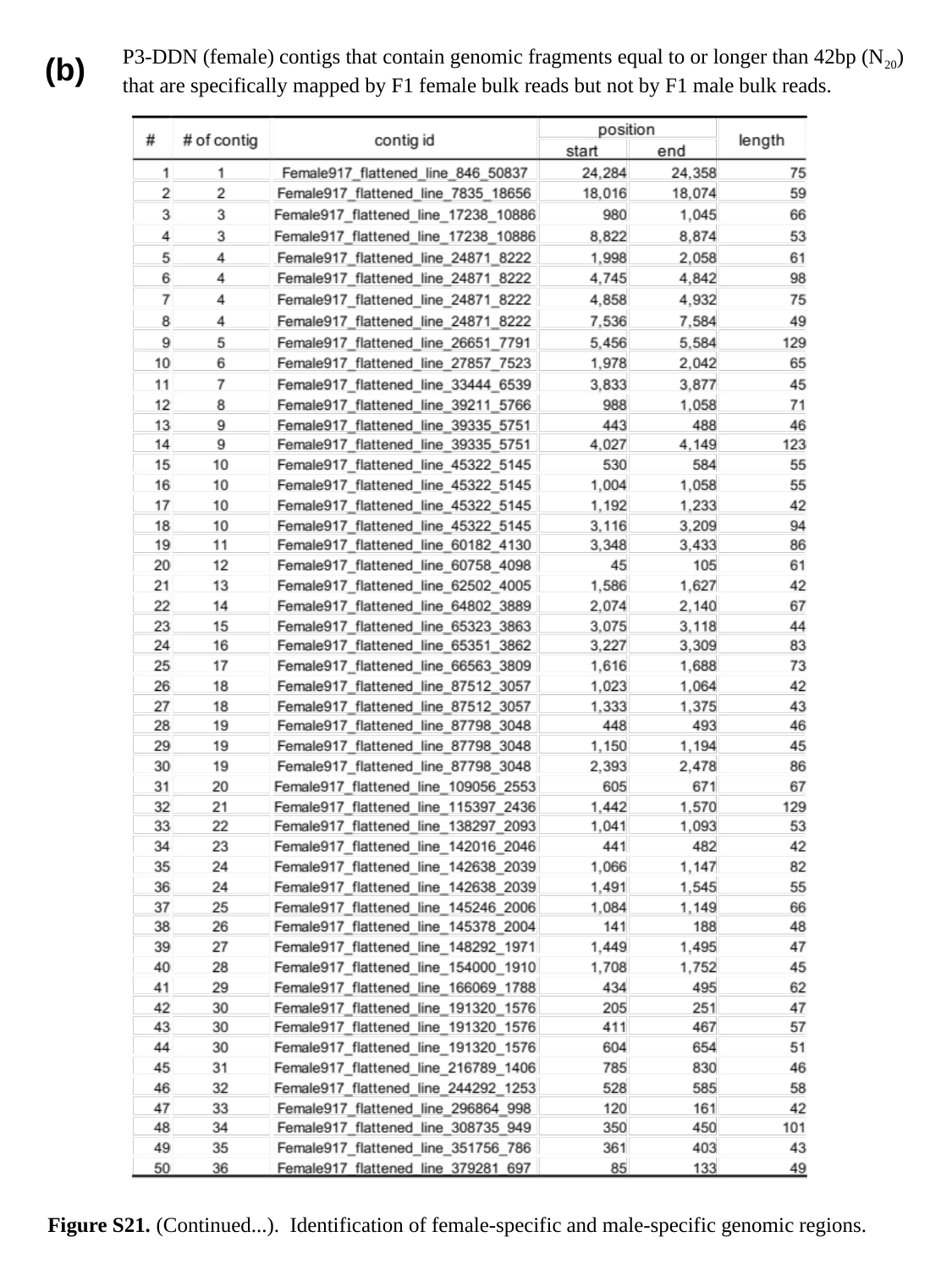

P3-DDN (female) contigs that contain genomic fragments equal to or longer than 42bp (N20)
 that are specifically mapped by F1 female bulk reads but not by F1 male bulk reads.
(b)
Figure S21. (Continued...). Identification of female-specific and male-specific genomic regions.

## Slide 45
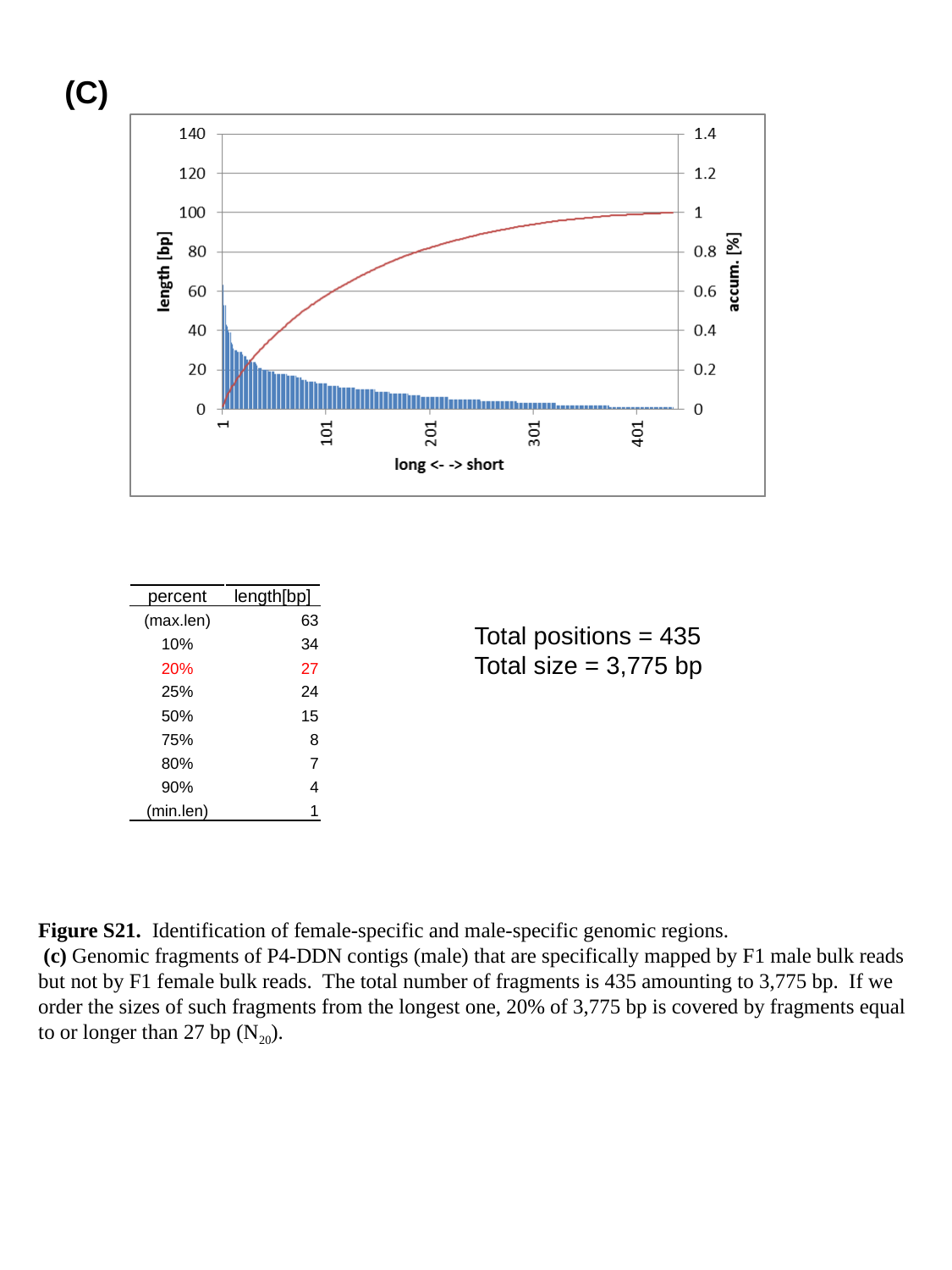

(C)
| percent | length[bp] |
| --- | --- |
| (max.len) | 63 |
| 10% | 34 |
| 20% | 27 |
| 25% | 24 |
| 50% | 15 |
| 75% | 8 |
| 80% | 7 |
| 90% | 4 |
| (min.len) | 1 |
Total positions = 435
Total size = 3,775 bp
Figure S21. Identification of female-specific and male-specific genomic regions.
 (c) Genomic fragments of P4-DDN contigs (male) that are specifically mapped by F1 male bulk reads but not by F1 female bulk reads. The total number of fragments is 435 amounting to 3,775 bp. If we order the sizes of such fragments from the longest one, 20% of 3,775 bp is covered by fragments equal to or longer than 27 bp (N20).

## Slide 46
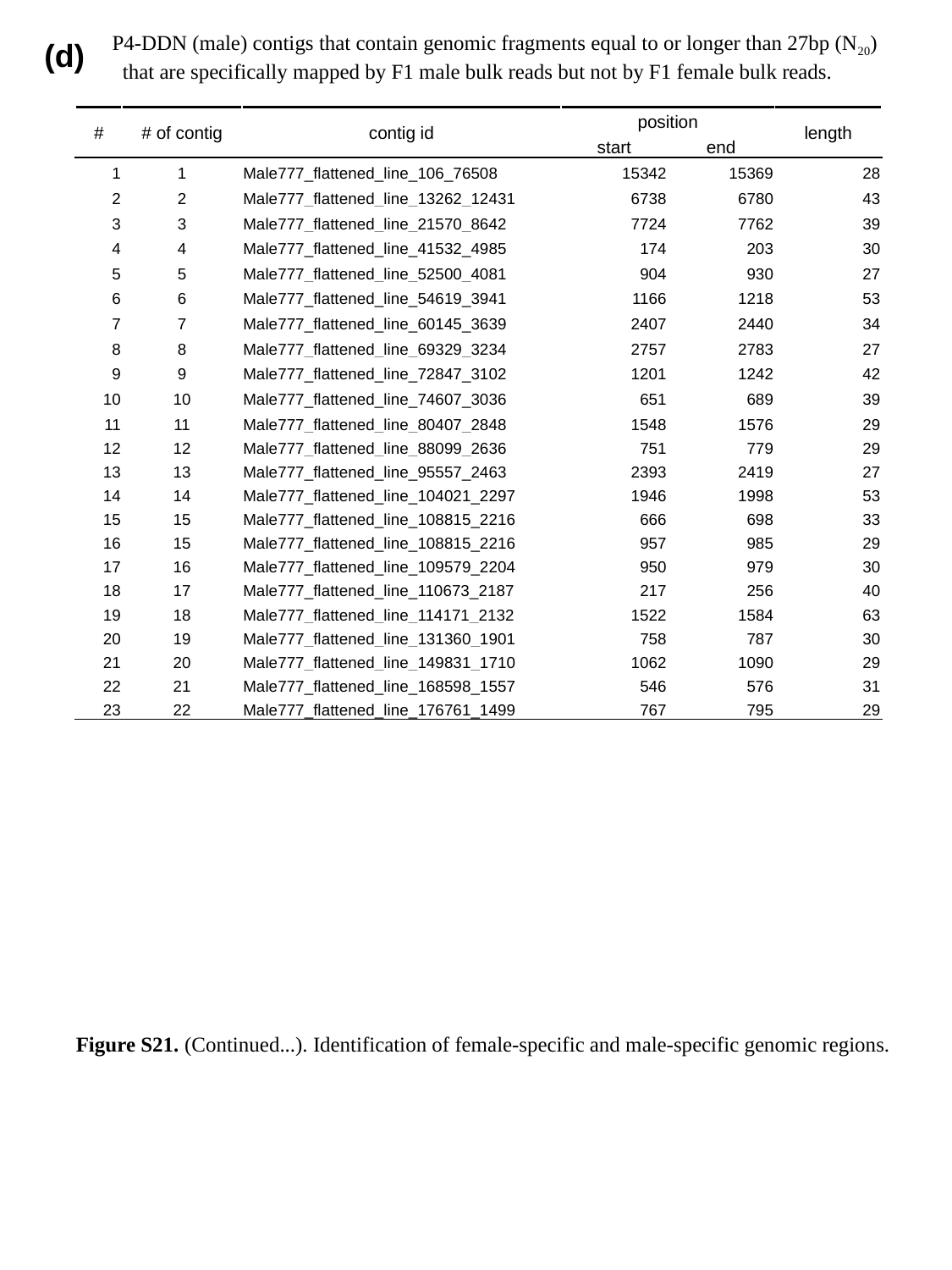

P4-DDN (male) contigs that contain genomic fragments equal to or longer than 27bp (N20)
 that are specifically mapped by F1 male bulk reads but not by F1 female bulk reads.
(d)
| # | # of contig | contig id | position | | length |
| --- | --- | --- | --- | --- | --- |
| | | | start | end | |
| 1 | 1 | Male777\_flattened\_line\_106\_76508 | 15342 | 15369 | 28 |
| 2 | 2 | Male777\_flattened\_line\_13262\_12431 | 6738 | 6780 | 43 |
| 3 | 3 | Male777\_flattened\_line\_21570\_8642 | 7724 | 7762 | 39 |
| 4 | 4 | Male777\_flattened\_line\_41532\_4985 | 174 | 203 | 30 |
| 5 | 5 | Male777\_flattened\_line\_52500\_4081 | 904 | 930 | 27 |
| 6 | 6 | Male777\_flattened\_line\_54619\_3941 | 1166 | 1218 | 53 |
| 7 | 7 | Male777\_flattened\_line\_60145\_3639 | 2407 | 2440 | 34 |
| 8 | 8 | Male777\_flattened\_line\_69329\_3234 | 2757 | 2783 | 27 |
| 9 | 9 | Male777\_flattened\_line\_72847\_3102 | 1201 | 1242 | 42 |
| 10 | 10 | Male777\_flattened\_line\_74607\_3036 | 651 | 689 | 39 |
| 11 | 11 | Male777\_flattened\_line\_80407\_2848 | 1548 | 1576 | 29 |
| 12 | 12 | Male777\_flattened\_line\_88099\_2636 | 751 | 779 | 29 |
| 13 | 13 | Male777\_flattened\_line\_95557\_2463 | 2393 | 2419 | 27 |
| 14 | 14 | Male777\_flattened\_line\_104021\_2297 | 1946 | 1998 | 53 |
| 15 | 15 | Male777\_flattened\_line\_108815\_2216 | 666 | 698 | 33 |
| 16 | 15 | Male777\_flattened\_line\_108815\_2216 | 957 | 985 | 29 |
| 17 | 16 | Male777\_flattened\_line\_109579\_2204 | 950 | 979 | 30 |
| 18 | 17 | Male777\_flattened\_line\_110673\_2187 | 217 | 256 | 40 |
| 19 | 18 | Male777\_flattened\_line\_114171\_2132 | 1522 | 1584 | 63 |
| 20 | 19 | Male777\_flattened\_line\_131360\_1901 | 758 | 787 | 30 |
| 21 | 20 | Male777\_flattened\_line\_149831\_1710 | 1062 | 1090 | 29 |
| 22 | 21 | Male777\_flattened\_line\_168598\_1557 | 546 | 576 | 31 |
| 23 | 22 | Male777\_flattened\_line\_176761\_1499 | 767 | 795 | 29 |
Figure S21. (Continued...). Identification of female-specific and male-specific genomic regions.

## Slide 47
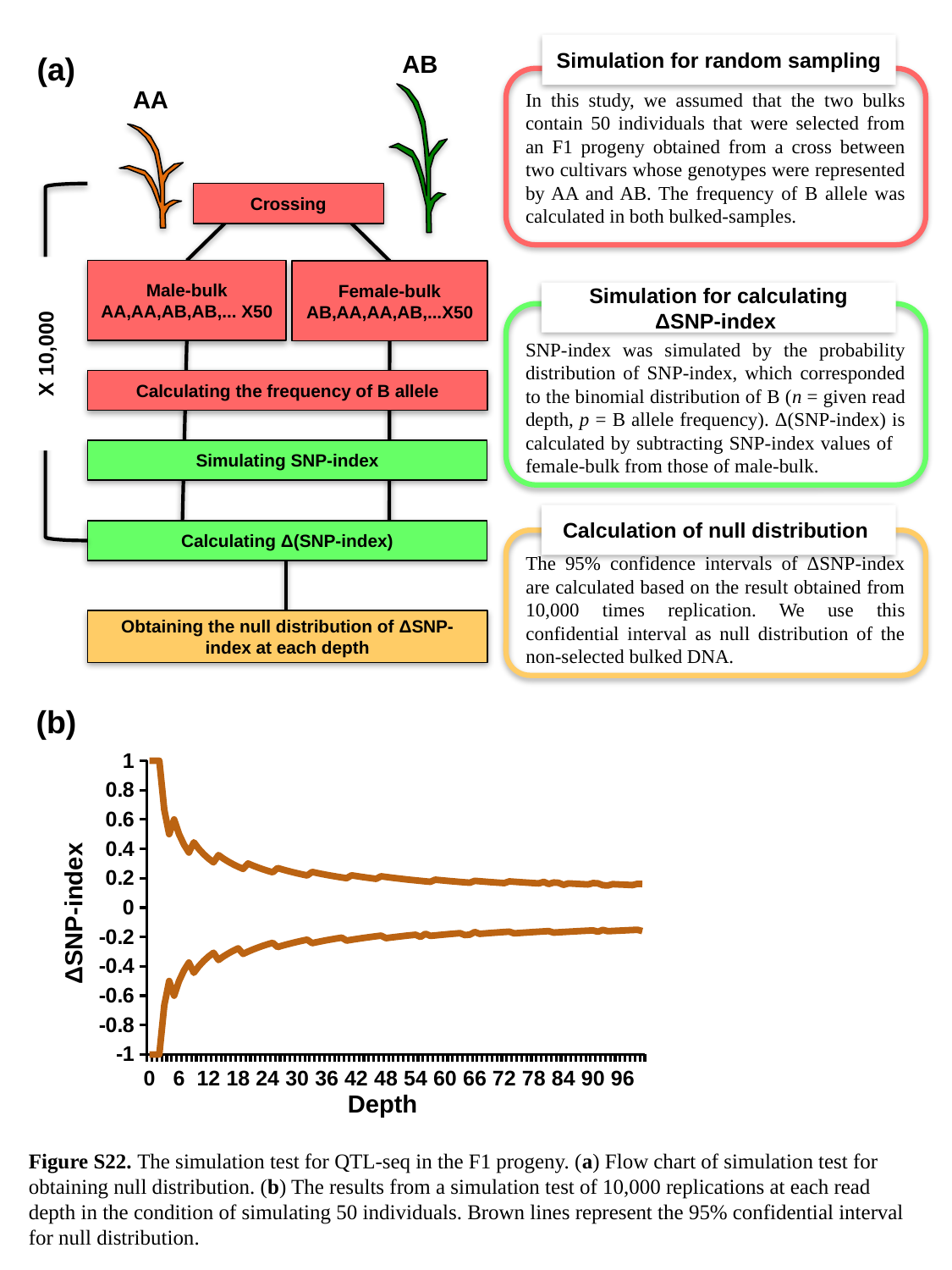

Simulation for random sampling
AB
(a)
AA
In this study, we assumed that the two bulks contain 50 individuals that were selected from an F1 progeny obtained from a cross between two cultivars whose genotypes were represented by AA and AB. The frequency of B allele was calculated in both bulked-samples.
Crossing
Male-bulk
AA,AA,AB,AB,... X50
Female-bulk
AB,AA,AA,AB,...X50
Simulation for calculating ΔSNP-index
SNP-index was simulated by the probability distribution of SNP-index, which corresponded to the binomial distribution of B (n = given read depth, p = B allele frequency). Δ(SNP-index) is calculated by subtracting SNP-index values of female-bulk from those of male-bulk.
X 10,000
Calculating the frequency of B allele
Simulating SNP-index
Calculation of null distribution
Calculating Δ(SNP-index)
The 95% confidence intervals of ΔSNP-index are calculated based on the result obtained from 10,000 times replication. We use this confidential interval as null distribution of the non-selected bulked DNA.
Obtaining the null distribution of ΔSNP-index at each depth
(b)
### Chart
| Category | | |
|---|---|---|
| 0.0 | 1.0 | -1.0 |
| 1.0 | 1.0 | -1.0 |
| 2.0 | 1.0 | -1.0 |
| 3.0 | 0.666666666666667 | -0.666666666666667 |
| 4.0 | 0.5 | -0.5 |
| 5.0 | 0.6 | -0.6 |
| 6.0 | 0.5 | -0.5 |
| 7.0 | 0.428571428571429 | -0.428571428571429 |
| 8.0 | 0.375 | -0.375 |
| 9.0 | 0.444444444444444 | -0.444444444444444 |
| 10.0 | 0.4 | -0.4 |
| 11.0 | 0.363636363636364 | -0.363636363636364 |
| 12.0 | 0.333333333333333 | -0.333333333333333 |
| 13.0 | 0.307692307692308 | -0.307692307692308 |
| 14.0 | 0.357142857142857 | -0.357142857142857 |
| 15.0 | 0.333333333333333 | -0.333333333333333 |
| 16.0 | 0.3125 | -0.3125 |
| 17.0 | 0.294117647058824 | -0.294117647058824 |
| 18.0 | 0.277777777777778 | -0.277777777777778 |
| 19.0 | 0.263157894736842 | -0.315789473684211 |
| 20.0 | 0.3 | -0.3 |
| 21.0 | 0.285714285714286 | -0.285714285714286 |
| 22.0 | 0.272727272727273 | -0.272727272727273 |
| 23.0 | 0.260869565217391 | -0.260869565217391 |
| 24.0 | 0.25 | -0.25 |
| 25.0 | 0.24 | -0.24 |
| 26.0 | 0.269230769230769 | -0.269230769230769 |
| 27.0 | 0.259259259259259 | -0.259259259259259 |
| 28.0 | 0.25 | -0.25 |
| 29.0 | 0.241379310344828 | -0.241379310344828 |
| 30.0 | 0.233333333333333 | -0.233333333333333 |
| 31.0 | 0.225806451612903 | -0.225806451612903 |
| 32.0 | 0.21875 | -0.21875 |
| 33.0 | 0.242424242424242 | -0.242424242424242 |
| 34.0 | 0.235294117647059 | -0.235294117647059 |
| 35.0 | 0.228571428571429 | -0.228571428571429 |
| 36.0 | 0.222222222222222 | -0.222222222222222 |
| 37.0 | 0.216216216216216 | -0.216216216216216 |
| 38.0 | 0.210526315789474 | -0.210526315789474 |
| 39.0 | 0.205128205128205 | -0.205128205128205 |
| 40.0 | 0.2 | -0.225 |
| 41.0 | 0.219512195121951 | -0.219512195121951 |
| 42.0 | 0.214285714285714 | -0.214285714285714 |
| 43.0 | 0.209302325581395 | -0.209302325581395 |
| 44.0 | 0.204545454545455 | -0.204545454545455 |
| 45.0 | 0.2 | -0.2 |
| 46.0 | 0.195652173913043 | -0.195652173913043 |
| 47.0 | 0.212765957446808 | -0.191489361702128 |
| 48.0 | 0.208333333333333 | -0.208333333333333 |
| 49.0 | 0.204081632653061 | -0.204081632653061 |
| 50.0 | 0.2 | -0.2 |
| 51.0 | 0.196078431372549 | -0.196078431372549 |
| 52.0 | 0.192307692307692 | -0.192307692307692 |
| 53.0 | 0.188679245283019 | -0.188679245283019 |
| 54.0 | 0.185185185185185 | -0.185185185185185 |
| 55.0 | 0.181818181818182 | -0.2 |
| 56.0 | 0.178571428571429 | -0.178571428571429 |
| 57.0 | 0.175438596491228 | -0.192982456140351 |
| 58.0 | 0.189655172413793 | -0.189655172413793 |
| 59.0 | 0.186440677966102 | -0.186440677966102 |
| 60.0 | 0.183333333333333 | -0.183333333333333 |
| 61.0 | 0.180327868852459 | -0.180327868852459 |
| 62.0 | 0.17741935483871 | -0.17741935483871 |
| 63.0 | 0.174603174603175 | -0.174603174603175 |
| 64.0 | 0.171875 | -0.1875 |
| 65.0 | 0.169230769230769 | -0.184615384615385 |
| 66.0 | 0.181818181818182 | -0.166666666666667 |
| 67.0 | 0.17910447761194 | -0.17910447761194 |
| 68.0 | 0.176470588235294 | -0.176470588235294 |
| 69.0 | 0.173913043478261 | -0.173913043478261 |
| 70.0 | 0.171428571428571 | -0.171428571428571 |
| 71.0 | 0.169014084507042 | -0.169014084507042 |
| 72.0 | 0.166666666666667 | -0.166666666666667 |
| 73.0 | 0.178082191780822 | -0.164383561643836 |
| 74.0 | 0.175675675675676 | -0.175675675675676 |
| 75.0 | 0.173333333333333 | -0.173333333333333 |
| 76.0 | 0.171052631578947 | -0.171052631578947 |
| 77.0 | 0.168831168831169 | -0.168831168831169 |
| 78.0 | 0.166666666666667 | -0.166666666666667 |
| 79.0 | 0.164556962025316 | -0.164556962025316 |
| 80.0 | 0.175 | -0.1625 |
| 81.0 | 0.160493827160494 | -0.160493827160494 |
| 82.0 | 0.170731707317073 | -0.170731707317073 |
| 83.0 | 0.168674698795181 | -0.168674698795181 |
| 84.0 | 0.154761904761905 | -0.166666666666667 |
| 85.0 | 0.164705882352941 | -0.164705882352941 |
| 86.0 | 0.162790697674419 | -0.162790697674419 |
| 87.0 | 0.160919540229885 | -0.160919540229885 |
| 88.0 | 0.159090909090909 | -0.159090909090909 |
| 89.0 | 0.157303370786517 | -0.157303370786517 |
| 90.0 | 0.166666666666667 | -0.155555555555556 |
| 91.0 | 0.164835164835165 | -0.164835164835165 |
| 92.0 | 0.152173913043478 | -0.152173913043478 |
| 93.0 | 0.150537634408602 | -0.161290322580645 |
| 94.0 | 0.159574468085106 | -0.159574468085106 |
| 95.0 | 0.157894736842105 | -0.157894736842105 |
| 96.0 | 0.15625 | -0.15625 |
| 97.0 | 0.154639175257732 | -0.154639175257732 |
| 98.0 | 0.153061224489796 | -0.153061224489796 |
| 99.0 | 0.161616161616162 | -0.151515151515152 |
| 100.0 | 0.16 | -0.16 |ΔSNP-index
Depth
Figure S22. The simulation test for QTL-seq in the F1 progeny. (a) Flow chart of simulation test for obtaining null distribution. (b) The results from a simulation test of 10,000 replications at each read depth in the condition of simulating 50 individuals. Brown lines represent the 95% confidential interval for null distribution.
